# The Kanyakla study: Randomized controlled trial of a microclinic social network intervention for promoting engagement and retention in HIV care in rural western Kenya

**DOI:** 10.1371/journal.pone.0255945

**Published:** 2021-09-13

**Authors:** Matthew D. Hickey, Gor B. Ouma, Brian Mattah, Ben Pederson, Nicholas R. DesLauriers, Pamela Mohamed, Joyce Obanda, Abdi Odhiambo, Betty Njoroge, Linda Otieno, Daniel E. Zoughbie, Eric L. Ding, Kathryn J. Fiorella, Elizabeth A. Bukusi, Craig R. Cohen, Elvin H. Geng, Charles R. Salmen

**Affiliations:** 1 Division of HIV, Infectious Diseases & Global Medicine, University of California San Francisco, San Francisco, California, United States of America; 2 Organic Health Response Research Group, Mfangano Island, Kenya; 3 Ekialo Kiona Centre, Mfangano Island, Kenya; 4 Providence Oregon Family Medicine Residency, Portland, Oregon, United States of America; 5 Department of Medicine, University of Washington, Seattle, Washington, United States of America; 6 Centre for Microbiology Research, Kenya Medical Research Institute, Nairobi, Kenya; 7 Family AIDS Care and Education Services (FACES), Kisumu, Kenya; 8 Microclinic International, San Francisco, California, United States of America; 9 Department of Population Medicine and Diagnostic Sciences, Cornell University, Ithaca, New York, United States of America; 10 Department of Obstetrics, Gynecology, & Reproductive Medicine, University of California San Francisco, San Francisco, California, United States of America; 11 Division of Infectious Diseases, Washington University, St Louis, St Louis, Missouri, United States of America; 12 Department of Family Medicine and Community Health, University of Minnesota, Minneapolis, Minnesota, United States of America; Brown University, UNITED STATES

## Abstract

**Background:**

Existing social relationships are a potential source of “social capital” that can enhance support for sustained retention in HIV care. A previous pilot study of a social network-based ‘microclinic’ intervention, including group health education and facilitated HIV status disclosure, reduced disengagement from HIV care. We conducted a pragmatic randomized trial to evaluate microclinic effectiveness.

**Methods:**

In nine rural health facilities in western Kenya, we randomized HIV-positive adults with a recent missed clinic visit to either participation in a microclinic or usual care (NCT02474992). We collected visit data at all clinics where participants accessed care and evaluated intervention effect on disengagement from care (≥90-day absence from care after a missed visit) and the proportion of time patients were adherent to clinic visits (‘time-in-care’). We also evaluated changes in social support, HIV status disclosure, and HIV-associated stigma.

**Results:**

Of 350 eligible patients, 304 (87%) enrolled, with 154 randomized to intervention and 150 to control. Over one year of follow-up, disengagement from care was similar in intervention and control (18% vs 17%, hazard ratio 1.03, 95% CI 0.61–1.75), as was time-in-care (risk difference -2.8%, 95% CI -10.0% to +4.5%). The intervention improved social support for attending clinic appointments (+0.4 units on 5-point scale, 95% CI 0.08–0.63), HIV status disclosure to close social supports (+0.3 persons, 95% CI 0.2–0.5), and reduced stigma (-0.3 units on 5-point scale, 95% CI -0.40 to -0.17).

**Conclusions:**

The data from our pragmatic randomized trial in rural western Kenya are compatible with the null hypothesis of no difference in HIV care engagement between those who participated in a microclinic intervention and those who did not, despite improvements in proposed intervention mechanisms of action. However, some benefit or harm cannot be ruled out because the confidence intervals were wide. Results differ from a prior quasi-experimental pilot study, highlighting important implementation considerations when evaluating complex social interventions for HIV care.

**Trial registration:**

**Clinical trial number**: NCT02474992.

## Introduction

As of 2019, an estimated 25.4 million people living with HIV (PLHIV) were on life-saving antiretroviral therapy (ART) worldwide [1]. As a result of this global progress towards improving HIV/AIDS mortality, millions of PLHIV have transitioned from management of an acute infection to management of a complex chronic illness, requiring regular follow-up and lifelong adherence to medication. Sustaining a high level of lifelong treatment engagement is particularly challenging within resource-limited settings. For instance, up to one-third of PLHIV in sub-Saharan Africa discontinue treatment within the first three years after initiating therapy [2]. Reasons for disengagement from care are diverse, though psychosocial barriers, often attributed to effects of stigma, predominate among those who discontinue treatment altogether [3, 4]. Interventions to prevent disengagement and, when it occurs, promote re-engagement in care are urgently needed, particularly in the era of “test-and-treat”, as increasingly hard-to-reach individuals are finally being linked to care who may require additional support to maintain long-term retention in care [5].

Several interventions have been proposed to improve psychosocial support for maintaining medication adherence and care engagement [6]. Community-based treatment supporters have shown efficacy for improving retention in care [7, 8], though assistance from a single supporter may be limited and potentially less beneficial than support from a broader support network [9]. Differentiated service delivery models such as adherence clubs, where group members share responsibility for monthly ART pick-up, have been widely implemented to both decongest health facilities and to provide patients with additional social support [10–13]. Qualitative studies suggest that these models may impact adherence by improving social support [14, 15], however many patients prefer to access care on an individual basis in a healthcare facility, rather than participate in an adherence club [16]. Microclinics are an alternative model that provide social-network based support for HIV treatment while allowing patients to access care individually [17].

Microclinics are network-based groups consisting of self-selected close social contacts (e.g. friends, family, co-workers, neighbors, etc) that focus on collective support for broad health goals, including HIV [18]. Microclinic interventions have also shown efficacy for improving diabetes control and other chronic disease management in a diverse array of low- and high-resource settings [19, 20]. Our group has proposed that microclinics could lead to improved engagement in care by reducing HIV-associated stigma and thus increasing access to social capital to provide support for both HIV treatment and overall health [17, 21].

In a prior quasi-experimental pilot study among an island community with high HIV prevalence in rural western Kenya, we previously demonstrated that offer to join a community-based microclinic group led to a 50% reduction in disengagement from HIV care [17]. In the present study, we conducted a pragmatic randomized trial of a targeted microclinic intervention at nine public clinics in rural western Kenya to understand the real-world impact of microclinics on HIV treatment outcomes. This targeted intervention was similar in content to the prior microclinic intervention, yet differed by focusing on patients at highest risk for disengagement from care and streamlining the intervention curriculum to improve cost-effectiveness and implementation efficiency.

## Methods

### Participants and setting

This study was conducted on Mfangano, Takawiri, Ringiti, and Remba Islands in Homa Bay County, Kenya where HIV prevalence is estimated at 21% among adults aged 15–49 [22]. Adult patients (≥18 years of age) at any of nine rural government-run health centers in the study area were eligible to participate if they missed a clinic visit by >3 days during study enrollment. Enrollment was conducted from 8/2015-2/2016 with data collection continuing through 2/2018. Participants were excluded if they had previously participated in the microclinic pilot study conducted at one of the clinics [17], lived outside the study area, or planned to move outside the study area in the following six months. The study was approved by the Kenya Medical Research Institute Ethical Review Committee and the University of California, San Francisco Human Research Protection Program. The study protocol is registered at ClinicalTrials.gov (NCT02474992). Written informed consent was obtained prior to study enrollment.

### Design and randomization

The study was an individual randomized controlled trial. Study staff prospectively reviewed clinic missed visit logs to identify participants missing a clinic visit by >3 days. Eligible patients were traced by study staff in the community and invited to participate in the study. After obtaining consent, 1:1 randomization was conducted using sequentially numbered sealed envelopes that were stratified by clinic site. The randomization sequence was generated by the study data manager, who was not involved in participant enrollment. Participants randomized to intervention were invited to form a microclinic group. Participants randomized to control were told that they would be guaranteed an opportunity to participate in a microclinic group and training program following completion of the study period. Group formation consisted of recruitment of 5–10 members of the participant’s social network who they commonly rely upon for support to form a health-focused group. There was no restriction on multiple study participants joining the same group. According to the participant’s preferences, recruitment could be conducted by the participant themselves, or by “microclinic facilitators”, i.e. community health workers (CHWs) trained to facilitate microclinic recruitment and training. If the participant preferred that their CHW conduct microclinic group recruitment, care was taken to avoid framing the group as focused on any one individual participant, but rather recruitment efforts described a group that was forming to help support collective health within the community. All participants were encouraged to return to the clinic if they had not done so already. All participants received standard HIV clinical care unrelated to their randomization assignment. Due to the nature of the intervention, it was not possible to blind participants to randomization allocation. Clinic staff were not notified of randomization assignment, though this information may have been available to them through inquiry with study participants during normal care activities.

### Intervention

The microclinic intervention consisted of formation of a group of 5–10 close family, friends and other members of the participant’s social support system, irrespective of the HIV status of these individuals. Microclinic groups did not provide antiretroviral therapy, but rather supplemented clinic-based care to provide community-based support for people living with HIV. Locally, these microclinic groups were known as “kanyaklas”, meaning “team” or “together” in vernacular Dholuo language. At the time of group formation, all members underwent individual HIV counseling and testing. Once formed, microclinic groups were assigned a CHW group facilitator and were guided through eight sessions scheduled every two weeks at a location of the group’s choosing. The curriculum was printed on customized flip-charts that could be easily carried by facilitators to community-based settings and used to facilitate the group sessions. Prior to each session, CHWs underwent a 3–4 hour “train-the-trainer” workshop to learn how to teach that session. CHWs then arranged with their group a meeting time and location of the group’s choosing and led each session without input from study staff. CHWs were paid a modest stipend for coordinating and facilitating each session.

Microclinic curriculum topics for the eight sessions included 1) program overview and group confidentiality, 2) HIV local epidemiology and prevention, 3) HIV treatment basics, 4) group support for HIV medication adherence and engagement in care, 5) local beliefs about HIV, herbs and nutrition, 6) group support for combating stigma, 7) group HIV status disclosure, and 8) debriefing of group disclosure and group support moving forward. The full intervention curriculum is available in the supplemental materials (S1 Appendix). Session seven involved group HIV status disclosure, allowing all group members to voluntarily be tested for HIV together and learn one another’s HIV status. This session was scheduled with a certified Voluntary Counseling and Testing counselor and care was taken to emphasize the voluntary nature of group testing and the importance of confidentiality. Participants were not compensated for attending microclinic sessions, though tea and snacks were provided for group participants.

### Measurements

Study staff conducted surveys and chart review to measure baseline demographics and clinical characteristics of participants. At baseline and 12 months post-enrollment, we conducted a social network index, asking participants to name all people who “provide close personal support or who are important to you”. For each of these individuals, participants were asked about mutual HIV status disclosure and social support in each of four domains (material support, emotional support, support attending clinic, support taking medications) using a 5-point Likert scale. We also measured perceived HIV-associated stigma at baseline and 12-months post-enrollment [23]. In addition to surveys, we conducted chart review including dates of scheduled and attended appointments. We conducted tracing of participants who left or were lost to follow up from their original clinics and reviewed charts for scheduled and attended appointments at any other clinics where the participant received care. For purposes of analysis, we assumed that participants who could not be located and were not in care at any other clinic on Mfangano, Remba, Ringiti, or Takawiri islands, or large neighboring mainland clinics, were disengaged from care. Participants received a small amount of compensation for their time of 100 Kenyan Shillings (~1 US Dollar) for completing baseline and end-of study surveys.

### Outcomes

Pre-specified primary outcomes were disengagement from care and ‘time in care’. Our primary disengagement outcome was defined as the time to the first instance of a 90-day absence from any discernable clinical care during 12 months of follow-up. Time in care was defined as the proportion of follow-up time spent adhering to clinic visit schedules over 12 months of follow-up [17].

We calculated gaps in care by determining the number of days from a missed clinic visit until return to any clinic within Homa Bay County. Participants were censored on the date of death or transfer to a facility outside Homa Bay County. Thus, 90-day disengagement indicates missing an appointment by at least 90 days and not known to have first transferred to another facility or died. Time in care is the proportion of follow up time that a participant adhered to clinic appointments and was calculated as [(total follow up time)–(sum of gaps in care)]/(total follow up time).

Pre-specified secondary outcomes focused on proposed mechanisms for the microclinic intervention. Based on end of study survey data, we evaluated mean scores on the HIV-associated stigma scale developed by Earnshaw et al to assess overall stigma, as well as internalized, anticipated, and enacted stigma [23]. We assessed HIV status disclosure based on the number of persons in each participant’s close social network who knew their HIV status and whose HIV status was known to the participant. We calculated mean Likert scale ratings of social support received from close social network members for material, emotional, clinic attendance, and medication management.

### Statistical analysis

Initial sample size calculations determined that we needed to enroll a minimum of 156 participants in each study arm, assuming complete follow-up, to detect a 50% reduction in disengagement in care in the intervention arm, accounting for participant clustering in microclinic groups with an average of two participants per group and a coefficient of variation of 0.25. This effect size was based on the effect size seen in a prior quasi-experimental pilot study [17]. We aimed for a larger sample size of 180 participants to account for attrition and errors in our calculations, though our primary outcomes are assessed in all enrolled participants regardless of whether they complete the end-of-study survey. The study completed planned follow-up and was not stopped early.

Using intention-to-treat analysis, we compared the rate of 90-day disengagement between study arms using Cox proportional hazards over the first 12 months of follow-up for our primary outcome. We conducted several sensitivity analysis, including evaluation of time to disengagement from care over extended follow-up to 24 months and comparison of time to modified composite primary outcome of 90-day disengagement from care or death. We evaluated the proportional hazards assumption both graphically and using Schoenfeld residuals. We used linear regression to compare differences in time in care between study arms, with boostrapping using 10,000 replications to address potential non-normality of residuals. For secondary outcomes, we used linear regression with bootstrapping using 10,000 replications to account for potential non-normality to evaluate intervention effect on end of study mean HIV-associated stigma and social support, adjusting for baseline values. We used Poisson regression to compare the number of close network supports to whom participants had disclosed HIV status to and whose HIV status was known between trial arms, adjusting for baseline values. We used robust standard errors that accounted for clustering by microclinic group in all comparisons.

## Results

**Fig 1:**
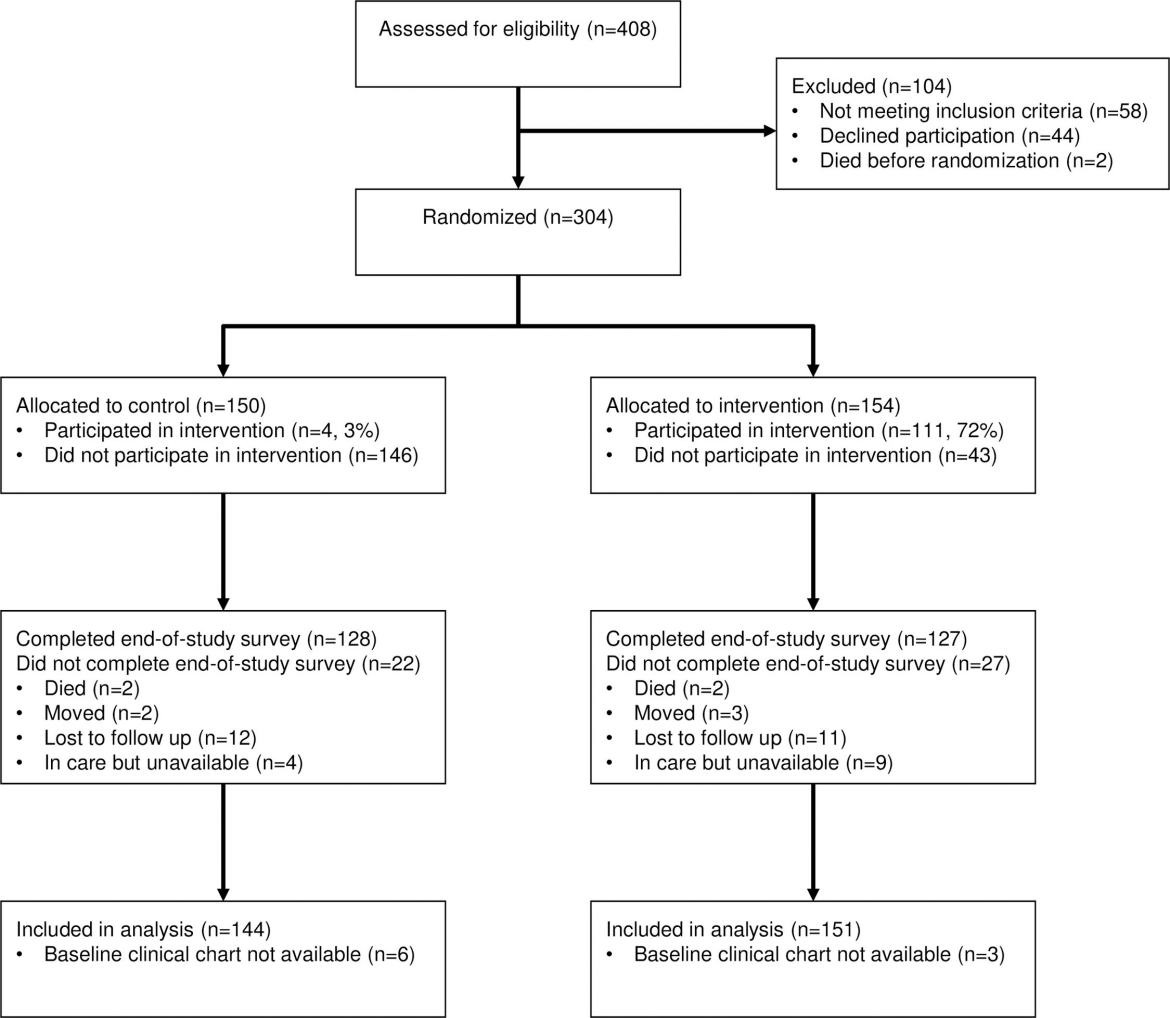
Consort diagram.

Of 350 eligible patients in nine rural HIV care facilities, 304 (87%) enrolled in the study (Fig 1), 45 declined participation, and 1 died before study staff could offer enrollment (S1 Table). Among the 46 who did not enroll, clinic records indicate that 41 (89%) had initiated ART, however only three participants (7%) self-reported taking ART; only three (7%) reported previously disclosing their HIV status to anyone (S2 Table). Baseline characteristics were similar between intervention and control (Table 1). Overall, approximately two-thirds of participants were women and the median age was 34 years (interquartile range, IQR 29–43). Most were ART-experienced (90%) with a median time on ART of 3.0 years (IQR 1.1–4.7). Most (94%) had disclosed their HIV status to at least one person other than clinic staff.

**Table 1:** Baseline characteristics of enrolled participants.

Characteristic	Control (n = 150)		Intervention (n = 154)	
Men, n (%)	44	29%	53	34%
Women, n (%)	106	71%	101	66%
Age, median (IQR)	34	29–44	35	29–42
Age category, n (%)				
18–25 years	18	12%	14	9%
26–49 years	111	74%	121	79%
≥50 years	21	14%	19	12%
Level of education completed, n (%)				
None/partial primary	84	56%	77	50%
Primary	56	37%	68	44%
Secondary	5	3%	7	5%
Post-secondary	5	3%	2	1%
Marital Status, n (%)				
Single/Never married	2	1%	5	3%
Separated/Divorced	10	7%	12	8%
Widowed	28	19%	19	12%
Married	110	73%	118	77%
Occupation, n (%)				
Fishing/Fish seller	64	43%	67	44%
Farming	17	11%	27	18%
Unemployed	15	10%	14	9%
Student	1	1%	1	1%
Other	53	35%	45	29%
HIV Status Disclosure				
Disclosed HIV status to anyone else, n (%)	137	91%	148	96%
Number of people disclosed HIV status, median (IQR)	4	2–7	4	2–8
Proportion of named close social supports disclosed to, mean (SD)	0.76	0.36	0.77	0.34
Clinical Characteristics				
Time since HIV diagnosis (yrs), median (IQR)	4.4	2.2–7.6	4.7	2.4–8.2
Time since clinic enrollment (yrs), median (IQR)	3.7	1.5–6.5	4.2	2.2–7.0
Proportion on ART at baseline, n (%)	131	87%	144	93%
Time since ART initiation (yrs), median (IQR)	2.8	1.0–4.8	2.9	1.1–4.6

IQR = interquartile range.

Among those in the intervention arm, 111 (72%) participated in a microclinic group (70% participation among men, 73% among women). Four of those in the control arm (3%) were recruited to join microclinics of other participants, and thus also participated in a microclinic group; all analyses were conducted by intention-to-treat. Fifty groups were formed in total, comprised of 485 total participants and ranging in size from 5–10 group members. Among 374 non-index participant microclinic group members, 134 (36%) reported known HIV-positive status at baseline and 7 reported a negative prior HIV test but were seropositive on baseline testing; all individuals living with HIV who were not in care or newly diagnosed were promptly referred to the nearest clinic for HIV care. Mean attendance for the eight sessions was 74%, and 89% of participants attended more than half of the sessions; 89% participated in group HIV testing and status disclosure. All study participants were asked about coercion or other harms associated with microclinic participation at study completion; no harms were reported.

### Primary outcomes

Over 12 months of follow-up, 27 (18%) intervention participants and 26 (17%) control participants disengaged from care for ≥90 days (Table 2). The incidence rate of 90-day disengagement was 20.3 per 100 person-years (95% confidence interval (CI) 13.9–29.7) in the intervention group and 19.5 per 100 person-years (95% CI 13.2–28.6) in the control group. Using an unadjusted Cox proportional hazards model, rates of 90-day disengagement were similar between intervention and control (hazard ratio (HR) 1.03, 95% CI 0.61–1.75). The intervention effect was similar across gender strata (men HR 1.01, 95% CI 0.52–1.96; women HR 1.03, 95% CI 0.44–2.43). Sensitivity analysis using a composite failure outcome of disengagement from care for 90 days or death, whichever occurred first, did not change outcomes (HR 1.03, 95% CI 0.63–1.69).

**Table 2:** Care engagement outcomes.

	Control						Intervention					
	Men (n = 44)		Women (n = 106)		Overall (n = 150)		Men (n = 53)		Women (n = 101)		Overall (n = 154)	
	n	%	n	%	n	%	n	%	n	%	n	%
Care outcomes over 1 year												
Consistently in care	30	68%	86	81%	116	77%	39	74%	77	76%	116	75%
Any 90-day disengagement	9	20%	17	16%	26	17%	11	21%	16	16%	27	18%
Death while in care	3	7%	0	0%	3	2%	2	4%	1	1%	3	2%
Transfer to facility out of study area	2	5%	3	3%	5	3%	1	2%	7	7%	8	5%
End of Study Outcomes at 2 years												
In care at end of study	27	61%	81	76%	108	72%	38	72%	77	76%	115	75%
Disengaged from care at end of study	12	28%	22	21%	34	23%	12	23%	16	16%	28	18%
Death while in care	3	7%	0	0%	3	2%	2	4%	1	1%	3	2%
Transfer to facility out of study area	2	5%	3	3%	5	3%	1	2%	7	7%	8	5%

Extended follow up to 24 months did not change these results, with 41 intervention participants (27%) and 40 control participants (27%) experiencing a 90-day absence from care at any time during the study (HR 1.02, 95% CI 0.67–1.56). At the end of 24 months, 28 intervention participants (18%) and 34 control participants (23%) had missed a visit by ≥90 days and not returned to any known care setting (HR 0.80, 95% CI 0.49–1.31). In additional exploratory analysis, incidence rates of 90-day disengagement from care over 24 months of follow-up differed between clinics on Mfangano and Takawiri Islands (10.1 per 100 person years, 95% CI 7.4–13.8) and for the more mobile populations on Remba and Ringiti Islands (42.4 per 100 person years, 95% CI 28.8–62.2). Stratified hazard of 90-day disengagement from care suggested possible differential effect by location (HR 1.32 for Mfangano/Takawiri, 95% CI 0.71–2.46; HR 0.73 for Remba/Ringiti, 94% CI 0.40–1.34) though the interaction term between trial arm and location was not significant (p = 0.18).

To further characterize engagement in care, we also evaluated the proportion of time spent adherent to clinic appointment schedules, termed *time in care*. The mean proportion of time spent in care over the first 12 months of follow up was 76.9% in intervention and 79.7% in control arms and did not differ significantly by treatment arm (absolute difference -2.8%, 95% CI -10.0% to +4.5%).

### Secondary outcomes

Table 3 describes differences in social network support, HIV status disclosure, and HIV-associated stigma at the end of the study by trial arm. Participants named a median of 3 close social supports at baseline (IQR 2–4) and 2 (IQR 1–3) after 12 months of follow up. Social network support was greater in intervention than control at the end of the study for material, emotional and support for attending clinic appointments. Participants randomized to intervention reported an average of 0.3 additional close social network supporters who knew their HIV status (0.3 persons, 95% CI 0.16 to 0.47), and 0.3 close network supporters whose status they knew by the end of the study (0.3 persons, 95% CI 0.11 to 0.43) compared to control. Stigma was also lower in the intervention arm compared to control (-0.28 units on 5-point Likert scale, 95% CI -0.40 to -0.17), largely driven by reductions in internalized stigma (-0.43, 95% CI -0.62 to -0.23) and anticipated stigma (-0.38, 95% CI -0.53 to -0.23). There were no reported harms or unintended effects.

**Table 3:** Intervention mechanisms: Social support, stigma, and HIV status disclosure.

	Control		Intervention		Intervention effect^d^
	Baseline	End	Baseline	End	*Beta (95% CI; p-value)*
	(n = 150)	(n = 128)	(n = 154)	(n = 127)	
Social Network Support (mean, 5-point scale)^a^					
Material Support	2.6	2.6	2.6	2.9	0.33 (95% CI 0.07 to 0.59; p = 0.01)
Emotional Support	2.7	2.7	2.8	3.1	0.36 (95% CI 0.12 to 0.60; p = 0.003)
Clinic Support	1.5	2.0	1.5	2.4	0.36 (95% CI 0.08 to 0.63; p = 0.01)
Medication Support	1.3	1.8	1.3	2.1	0.28 (95% CI -0.03 to 0.58; p = 0.08)
HIV-Associated Stigma (mean, 5-point scale)^b^					
Internalized	2.1	2.2	2.1	1.7	-0.43 (95% CI -0.62 to -0.23; p<0.001)
Anticipated	1.9	1.9	1.9	1.6	-0.38 (95% CI -0.53 to -0.23; p<0.001)
Enacted	1.1	1.1	1.2	1.1	-0.04 (95% CI -0.10 to 0.03; p = 0.3)
Mean overall	1.7	1.7	1.7	1.5	-0.28 (95% CI -0.40 to -0.17; p<0.001)
HIV Status Disclosure (mean)^c^					
Number of network members who know my status	2.2	1.9	2.3	2.5	0.3 (95% CI 0.16 to 0.47; p<0.001)
Number of network members for whom I know their status	1.9	1.6	2.0	2.2	0.3 (95% CI 0.11 to 0.43; p = 0.001)

^a^ Network index assessing different types of social support. 5-point scale (0 = no support; 4 = great deal of support).

^b^ HIV Stigma Framework (Earnshaw 2013). 5-point scale (1 = no stigma; 5 = severe stigma).

^c^ Social Network Index assessing bidirectional HIV disclosure status.

^d^ Comparing end of study values by trial arm adjusting for baseline values using logistic regression with bootstrapping for social network support and stigma outcomes and Poisson regression for HIV status disclosure outcomes. All analyses adjusted for clustering by microclinic group.

## Discussion

Despite improving social support, increasing HIV status disclosure, and reducing HIV-associated stigma, the invitation to participate in a targeted social network-based support intervention among patients missing routine HIV clinic visits did not significantly effect engagement in care. The confidence intervals around our effect size estimate were wide and compatible with both benefit and harm of the intervention. Lack of clear effectiveness of this intervention stands in contrast to improvements in care engagement in a prior quasi-experimental study of a similar community-wide social network microclinic intervention [17, 21]. Efforts to understand reasons for these discrepancies may shed light both on the behavioral mechanisms targeted by microclinics to improve engagement in HIV care, as well as on the broader context of discrepant results from implementation studies.

One potential interpretation of our finding that microclinics improved proposed mechanisms of effect without impacting care engagement is that interventions to improve social support, though important, may be insufficient to change clinical outcomes. There is a robust literature linking stigma, social support, and HIV status disclosure to care engagement [4, 24–27], though our findings suggest that social support interventions may be more effective if coupled with interventions to address residual structural or clinic-based barriers to care.

In the island communities where this study took place, mobility constituted a particularly notable structural barrier. We noted substantially greater disengagement from HIV care at locations further from the mainland where mobility is even more prevalent (i.e. Remba and Ringiti islands). Greater mobility among participants in locations with a larger proportion of migratory fisherfolk may present particular challenges for an intervention that relies on the strength of pre-existing social networks for efficacy. Surprisingly, when stratifying our intervention effect by location, we saw a nonsignificant reduction in disengagement from care associated with the intervention in more highly mobile islands and a nonsignificant increase in disengagement from care associated with the intervention on islands with relatively less mobility. Confidence intervals were wide and these differences were not statistically significant, so it is difficult to draw definitive conclusions from these findings. Nonetheless, our finding that disengagement from care is substantially more common among those accessing care in regions where mobility is more common is notable. Others have also noted the challenges of consistent access to HIV care among mobile fisherfolk [28], highlighting the need for interventions that both account for mobility and address structural barriers to care engagement specifically among mobile populations [29, 30]. Beyond mobility, other structural and clinic-level barriers not addressed by the microclinic intervention may have contributed to lack of intervention effect on care engagement [3].

Differences in intervention implementation and content may have also contributed to lack of intervention effect on care engagement and the discrepancy of this result with the effectiveness of our prior community-wide microclinic intervention. In the present study, we shortened the previous microclinic intervention into eight sessions instead of twelve. In attempt to focus the intervention on those most likely to disengage from care and to facilitate randomization, we also offered the intervention only to patients missing visits in the present study, as opposed to the entire clinic population from a particular community as we did in our prior study. At the same time, we focused on recruitment of microclinic groups based on preferences of these at-risk patients; in the previous study we simultaneously recruited both patient-centered microclinics as well as microclinics formed from among existing community groups in an effort to circumvent stigma associated with prior HIV-specific interventions [21]. In adapting our recruitment strategy for this randomized format, and to protect individual patient confidentiality, there was less community-wide mobilization and less community participation in our present study [21]. This focused engagement among randomized participants and their directly recruited networks alone may have considerably reduced unrecognized modifiers of impact of the intervention that relate to community-wide transitions in norms and practices, suggesting that microclinic groups may be an effective component of community-based retention in care strategies, but may not be sufficient alone to affect target outcomes as a siloed intervention [21].

Participation in the microclinic intervention was high, with 72% of those in the intervention arm participating in a group. However, incomplete participation may have attenuated the intervention effect, particularly if those most in need of additional support were less likely to join a microclinic group. Nonetheless, microclinic group participation was similar in our prior quasi-experimental pilot study, thus intervention participation is unlikely to explain discrepant results between the present trial and our prior pilot study [17].

Further, in the present randomized trial, our recruitment strategy may have failed to include those most in need of intervention, namely those who have not previously disclosed their HIV status and those who have not started ART despite their clinic initiating therapy. Among the 46 eligible participants who did not enroll in the study, 93% had not previously disclosed their HIV status to anyone, compared to only 6% baseline non-disclosure among individuals who enrolled in the study. In contrast to a community-wide recruitment strategy in our prior study [17], the present study’s attempt to target patients more likely to disengage from care may have inadvertently contributed to missed opportunities to engage those most likely to benefit from the increased social support, status disclosure, and stigma reductions that the intervention sought to effect.

Finally, study design features such as measurement error or lack of blinding could have also played a role in our lack of observed intervention effect. Though gaps in care have been associated with adverse HIV-associated outcomes [31], it is also possible that gaps observed in our study are not well correlated with medication adherence or care engagement. Travel is common and patients frequently obtain medications from other sources [32]. Though we sought to identify all other clinics where participants accessed care through extensive tracing, it is possible that we were not able to capture all sources of HIV care, particularly in this multi-site study where patients accessed care at a broad number of sites. Due to cost constraints, we were unable to measure HIV viral load; this measurement may have improved interpretation of our findings and further elucidated possible effects of the proposed intervention mediators of stigma, social support, and disclosure on ART adherence. Lack of blinding could have attenuated the intervention effect if clinicians more carefully monitored participants not known to participate in a microclinic to ensure they were receiving adequate support.

## Conclusion

The data from our pragmatic randomized trial in rural western Kenya are compatible with the null hypothesis of no difference in HIV care engagement between those who participated in a microclinic intervention and those who did not, despite improvements in proposed intervention mechanisms of action, namely social support, HIV-associated stigma, and HIV status disclosure. However, some benefit or harm cannot be ruled out because the confidence intervals were wide. One key implication is that interventions focused solely on improving social network support may fall short of improving HIV clinical outcomes if not coupled with interventions to simultaneously address structural and clinic-level barriers. At the same time, discrepant results observed between the prior community-wide and current more targeted microclinic interventions also highlight important considerations regarding the ways that study design, and particularly recruitment strategies for more vulnerable groups, may reduce intervention effectiveness. Additional strategies are needed to engage individuals experiencing greater stigma and lower rates of HIV status disclosure in social network interventions. Furthermore, given ongoing interest in social support approaches to harness social capital and network resilience across contexts and diseases, more research is needed to evaluate potential synergies between these complex social interventions and other structural and clinic-based interventions.

## Supporting information

S1 Appendix Kanyakla programme curriculum. (PDF)

S1 Table Reasons for non-enrollment. (DOCX)

S2 Table Baseline characteristics of enrolled vs non-enrolled participants. (DOCX)

S1 File CONSORT checklist. (DOC)

S2 File Study protocol. (DOC)
