# Supplementary material for: The Kanyakla study: Randomized controlled trial of a microclinic social network intervention for promoting engagement and retention in HIV care in rural western Kenya
Source: PLoS One. 2021 Sep 13;16(9):e0255945. doi: 10.1371/journal.pone.0255945 (PMC8437299; doi:10.1371/journal.pone.0255945)
Supplement: S1 Appendix — (PDF) [file pone.0255945.s001.pdf]

Kanyakla Programme

Mfangano Division, Kenya

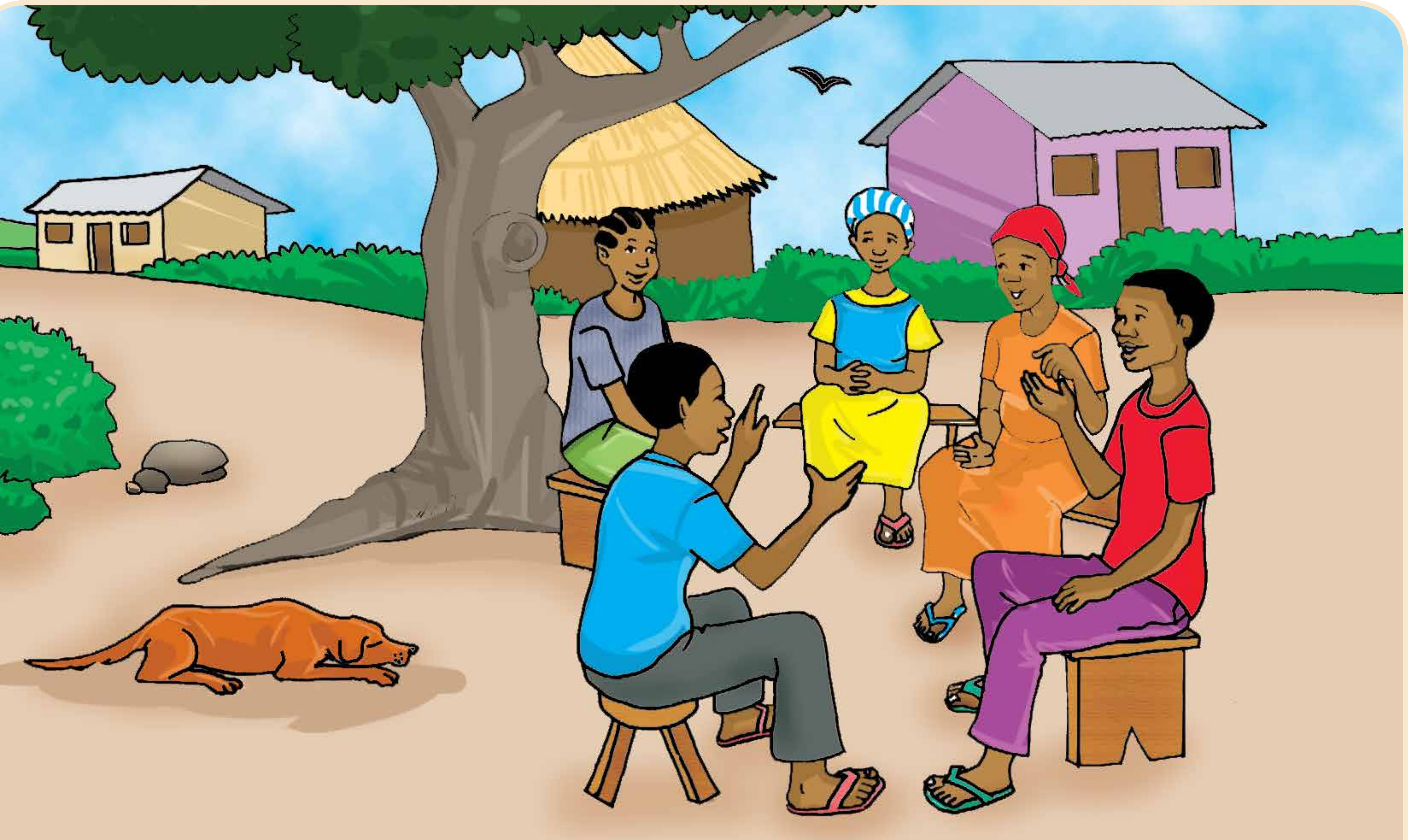

Mfangano Health Net

organic health response

MICROCLINIC INTERNATIONAL
Making good health contagious

SEGAL Family Foundation

Facilitator's Guide

Before You Start:

1. Revise this flipbook before you begin training to make sure you are familiar with the material.
2. Make sure you understand the objectives, activities, and key message for each session.
3. Prepare your materials before the discussion.
4. Prepare the venue early. Organise seats in a way that encourages discussion, such as in a circle.
5. The side that reads Facilitator's Guide should always face you. Images and visual aids should face out to the discussion group to help them understand the topics.

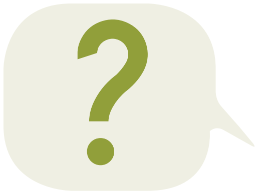

This symbol represents important discussion questions or topics for the Kanyakla.

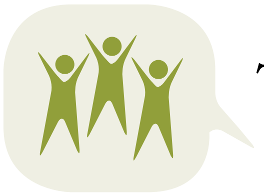

This symbol means there is a group activity, game, or role play for the Kanyakla.

Each session ends with a task or 'challenge' for the members to do on their own before the next session.

Tips:

1. Try to involve every group member. Ask them to take turns answering questions and giving their opinion.
2. If possible, involve different people each time in the role play sessions.
3. Get to know each group member by name. Use name tags at the beginning if necessary.

Table of contents

Session One: Pulling the Net Together 1

Life in Our Community 2
What is a Kanyakla? 3
Why Are the First Kanyakla Sessions about HIV? 4
What is Confidentiality? 5
Team Building Exercises 6
Let's Role Play! 7
The Way Forward 8

Session Two: HIV on Lake Victoria 9

HIV in Africa: The Scope of the Problem 10
What is HIV? 11
How HIV Works in the Body 12
HIV Fluids 13
Review Questions for Games 14
HIV Transmission and Safe Practices 15
The Way Forward 16

Session Three: Combination Antiretroviral Therapy 17

How HIV Works 18
How ARVs Work 19
Side Effects of ARVs 20
How Does HIV Become Resistant to ARVs? 21
Medication Adherence 22
The Way Forward 23

Session Four: Engaging with Care 24

5 Questions 25
What Steps Can I Take to Promote My Own Health? 26
As a Kanyakla, What Can We Do to Promote the Health of Others? 27
Let's Role Play! 28
Organising Medications 29
The Way Forward 30

Session Five: ARVs and You 31

Let's Role Play! 32
Local Beliefs Around HIV vs "Chira" 33
Herbs and Medications 34
Let's Role Play! 35
Nutrition for People Living with HIV/AIDS 36
The Way Forward 37

Session Six: Chasing Stigma 38

Let's Role Play! 39
Talking to Kids About Sex 40
Let's Role Play! 41
Stigma 42
5 Pillars of Kanyakla Support 43
The Way Forward 44

Session Seven: Group Disclosure 45

Let's Role Play! 46
HIV Status Disclosure 47
Team Building Exercises 48
Confidentiality 49
What to Expect 50
The Way Forward 51

Session Eight: The Way Forward 52

HIV Status Disclosure 53
The Way Forward: Staying Healthy 54
Moving Forward 55
The Way Forward 56

Facilitator's Guide

Objectives:

- Understand the Kanyakla Programme.
- Recognise the importance of confidentiality in relation to HIV/AIDS.
- Understand practice and enforcement of confidentiality in Kanyakla groups.

Session overview (110 minutes):

Activity	Time (minutes)	Objectives
Prayer and mindfulness	20	• Gather the group together and pray for a good session.<br>• Have the group reflect on the meditations listed below.
Class - Teach from the flipbook	15	• Help the group understand the goals of the Kanyakla programme.<br>• Identify Kanyakla as 'health groups', not just HIV groups.
Group Discussion	20	• Discuss confidentiality.
Trust games	25	• Play the human knot game and the ankle line game to build teamwork through trust.
Role play	20	• Through role play, illustrate why confidentiality is important.<br>• Use the script provided in the flipbook.
Wisdom circle	10	• Review the objectives of the session. What was learnt?<br>• Distribute a handout for the Kanyakla if it is provided.<br>• Plan the next session.

Meditations:

"Our fears arise from things we don't confront. Once we are willing to look fully and deeply at the source of a fear, it loses its power." – *Unknown*

"To heal is to touch with love, that which we previously touched with fear." – *Stephen Levine*

Session One: Pulling the Net Together

1 2 3 4 5 6 7 8

Objectives:

- Understand the Kanyakla Programme.
- Recognise the importance of confidentiality when discussing, treating, or dealing with HIV/AIDS.
- Understand the practice and enforcement of confidentiality in Kanyakla groups.

I know something about HIV, I can do something about it, and I can do something for someone else affected by HIV and AIDS!

Facilitator's Guide

Life in Our Community:

Use the questions below to lead a discussion that helps the group identify what is easy and what is difficult to get support for from their community.

1. What can we count on community support for?
2. When is it difficult to get community support?

   Suggest the following prompts: School fees? Food? Transportation? Funerals?
3. Could you get help from your community to pay for medications or go to the clinic?

Life in Our Community

?

1. In what ways are you supported by your community?
2. What things are difficult to get support for?

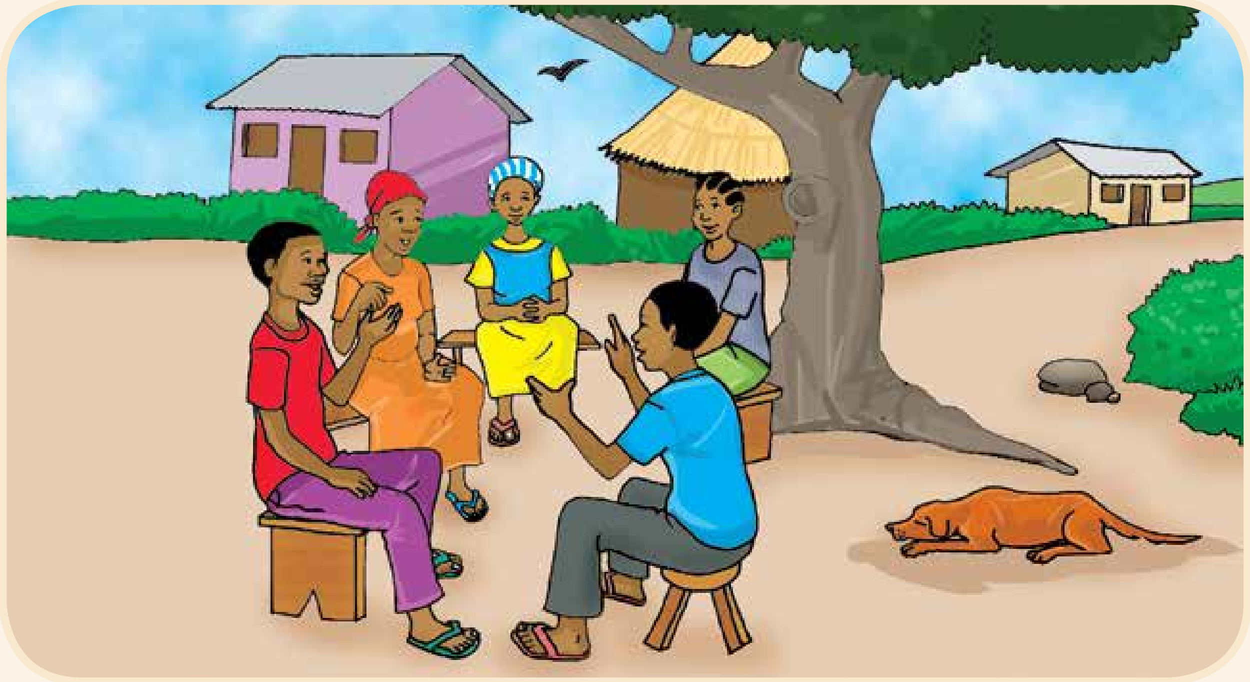

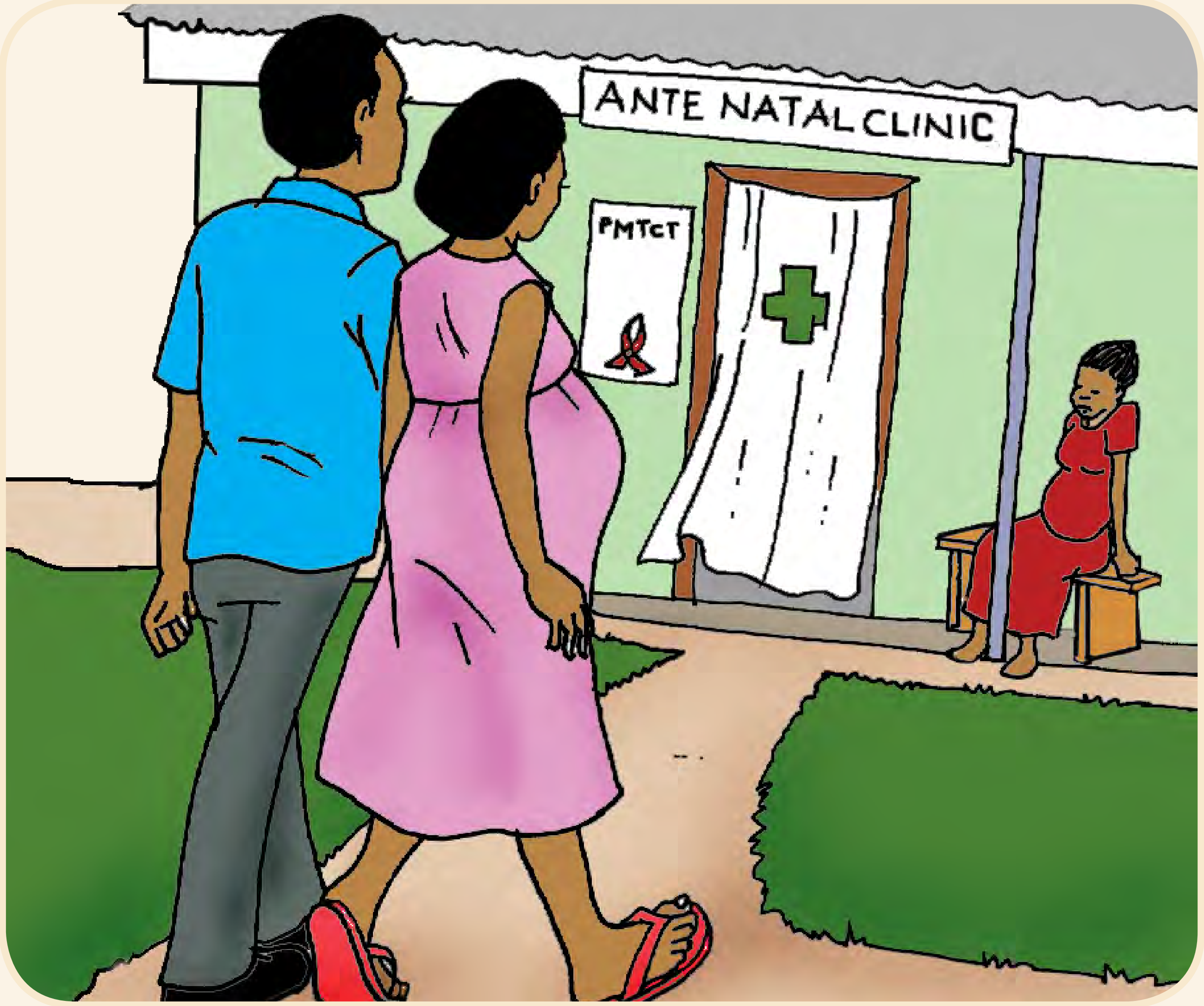

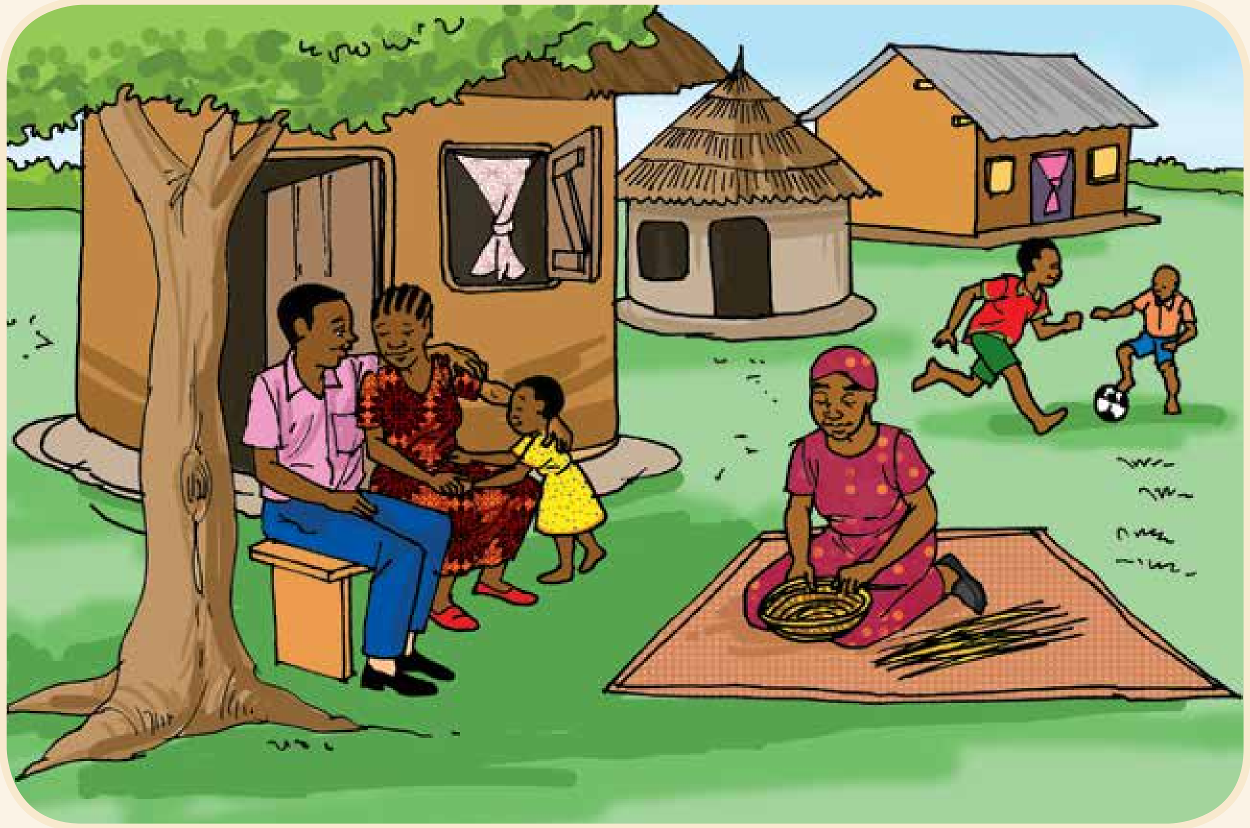

Facilitator's Guide

What is a Kanyakla?

Begin by asking the group what they think a Kanyakla is. Give them time to discuss their ideas before you explain what a Kanyakla is.

A Kanyankla is a health team.

Goals of a Kanyakla:

1. To form a health team that can be a source of support for maintaining health.
2. To provide community support. Kanyaklas can especially provide support for the things that the group identified as being difficult to access support for.

The 3 roles of a Kanyakla:

1. To listen to the challenges experienced by other Kanyakla members, provide support, and maintain confidentiality.
2. To promote healthy behaviours among Kanyakla members.
3. To promote healthy behaviours within the community.

What is a Kanyakla?

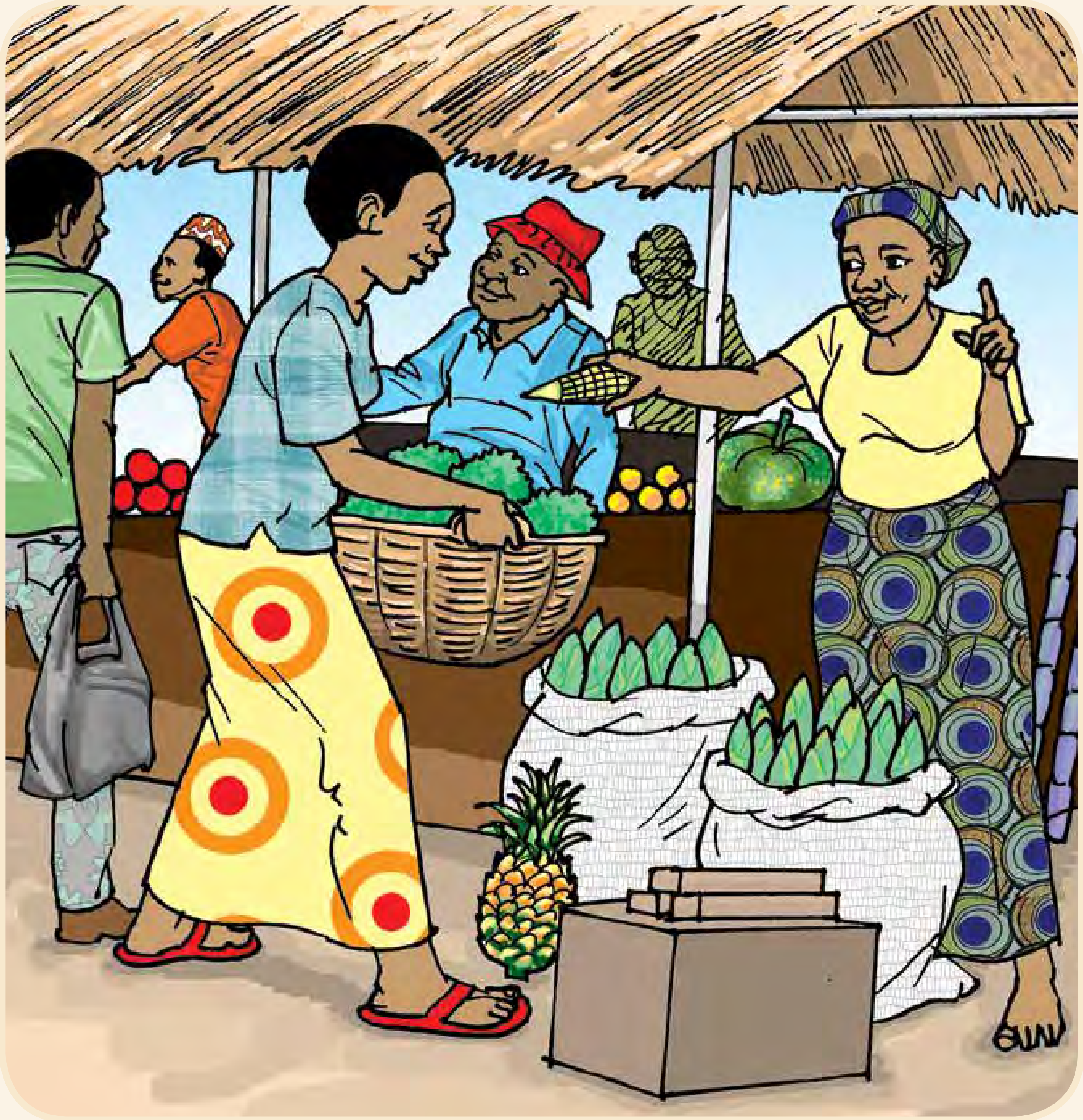

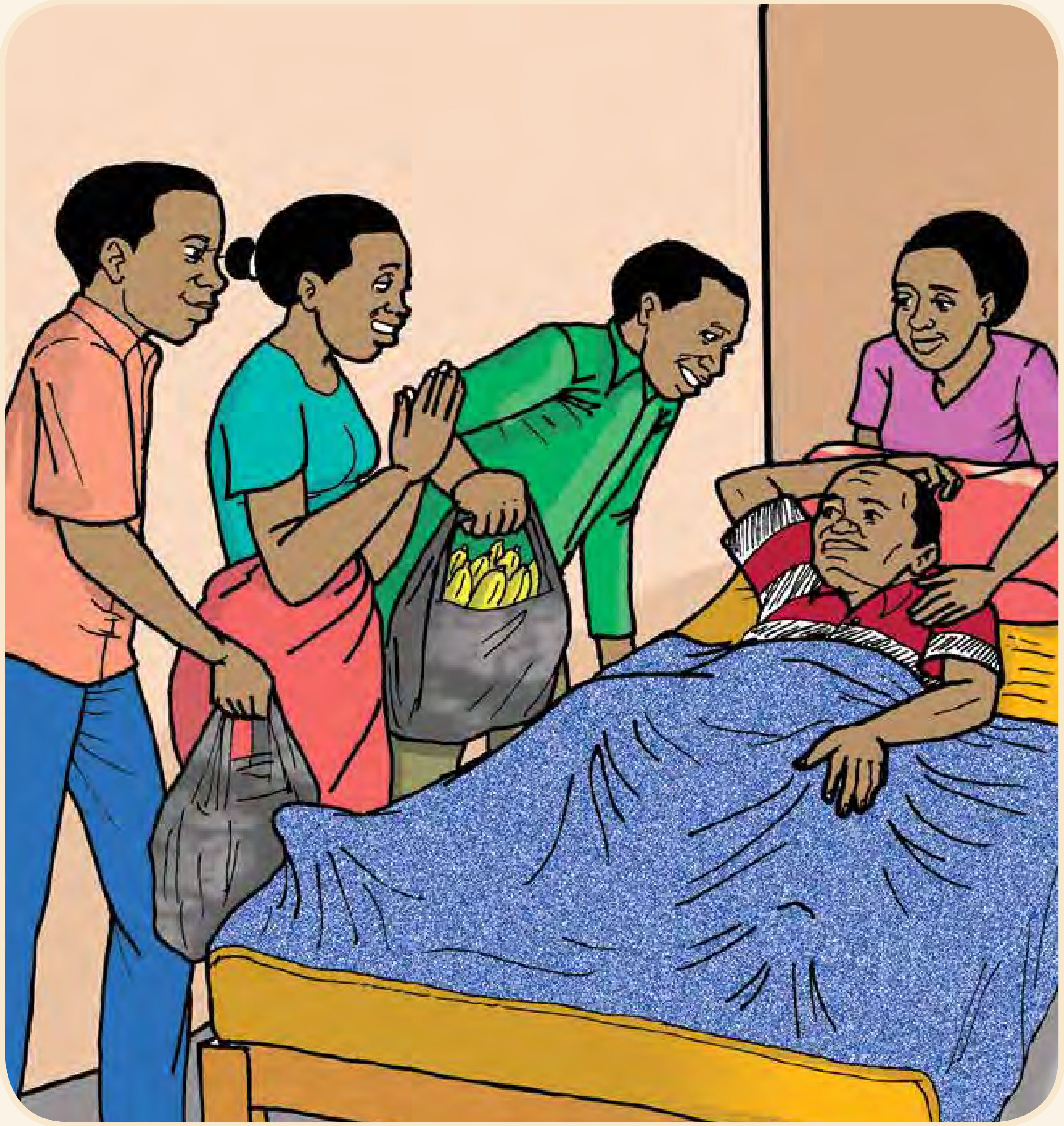

Facilitator’s Guide

Why Are the First Kanyakla Sessions About HIV?

Kanyaklas are intended to be health teams – focused on general health and well-being. So, why are the first sessions only about HIV?

Ask the group the following question:

- Raise your hand if you know someone with HIV.

The 3 roles of a Kanyakla:

We are talking about HIV first because it is a big health problem in our community.

5 in 20 people in Homa Bay County are infected with HIV. Kanyakla members may be HIV infected or uninfected, but we are all **AFFECTED** by HIV.

Key message:

- Kanyaklas are not only about HIV!
- We will start by discussing HIV because it affects us all, but we want these Kanyaklas to focus on all health issues that are important to the group.

Why Are the First Kanyakla Sessions About HIV?

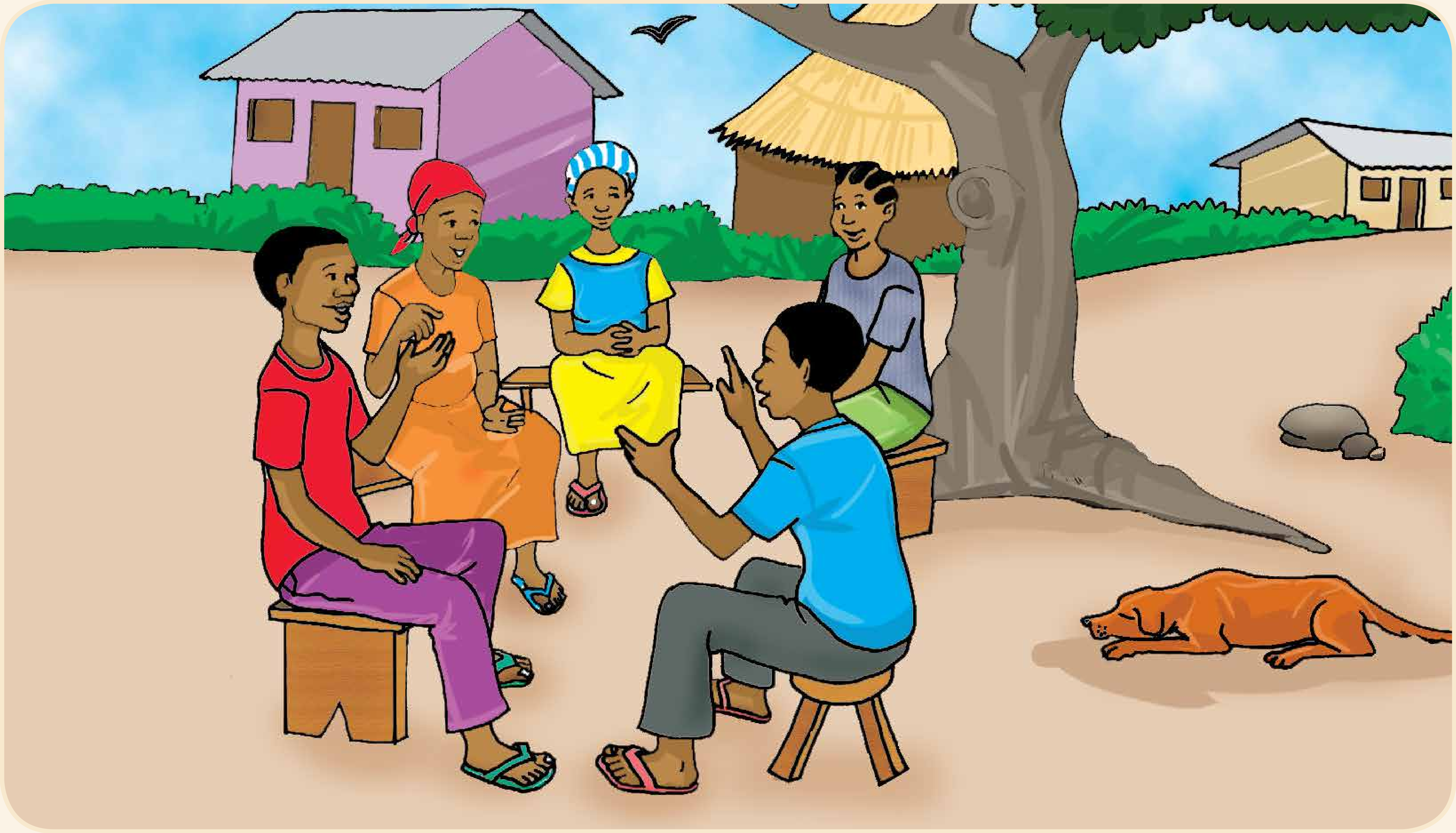

Facilitator's Guide

What is confidentiality?

Use the following questions to guide a discussion about the importance of confidentiality. As the group discusses, emphasise that a key part of the Kanyakla relationship is confidentiality.

1. What is confidentiality?
2. Why is confidentiality important?
3. How do we maintain confidentiality?
4. What happens if confidentiality is broken?

Key message:

- Confidentiality is about trust.

What is Confidentiality?

?

1. What is confidentiality?
2. Why is it important?
3. How do we maintain confidentiality?
4. What happens if confidentiality is broken?

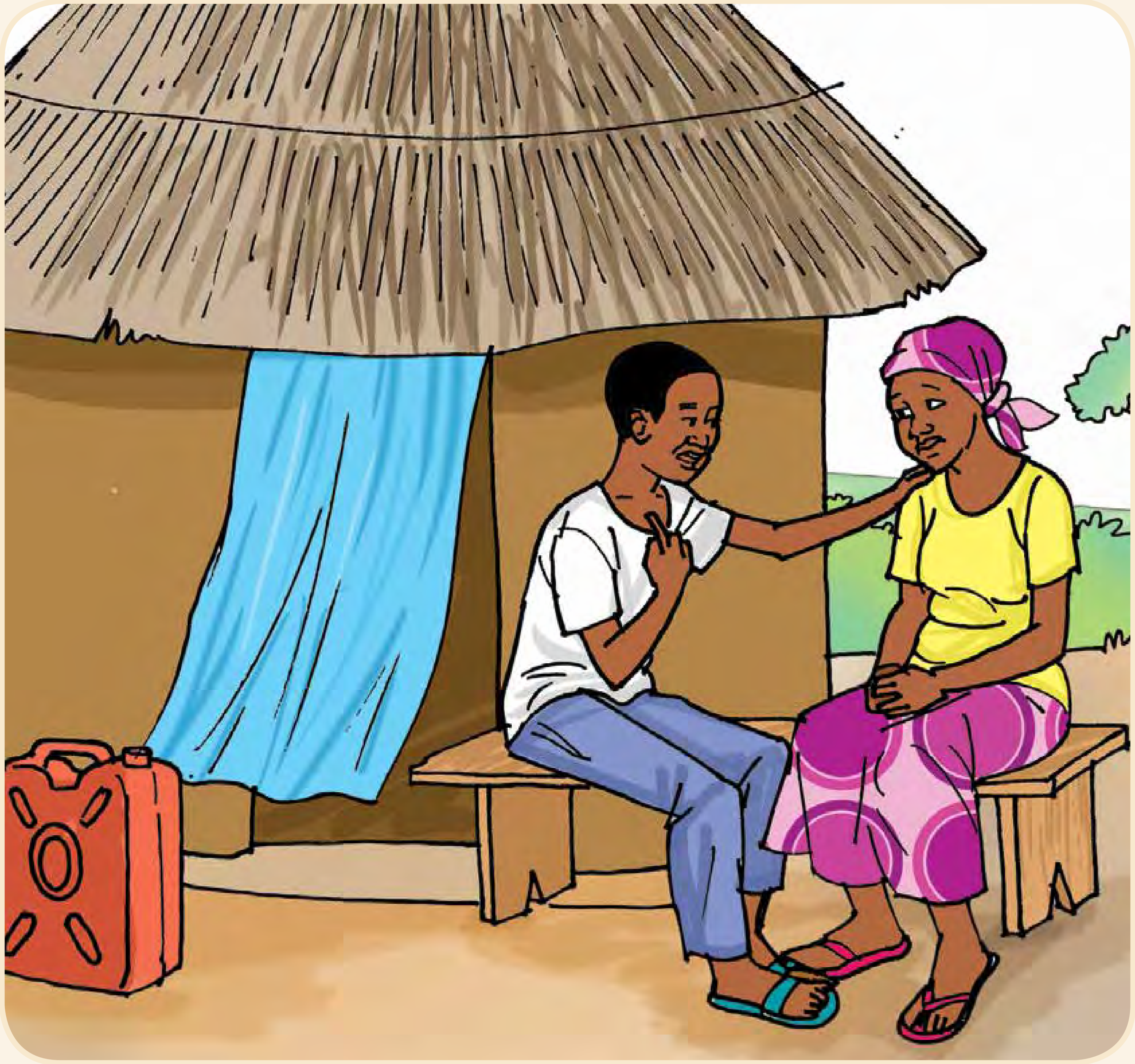

Team Building Exercises

Human Knot

Instructions:

1. Members of the Kanyakla form a circle.
2. Each person holds hands with 2 different people. Group members should not hold hands with someone who is standing right next to them.
3. The group works together to untangle themselves and form a perfect circle again.
4. This means group members might have to twist themselves around, crawl under or climb over each other's arms.
5. The group must achieve this while still holding each other's hands.

Ankle Line Walk

Instructions:

1. The group stands next to each other in a straight line on one side of the room.
2. Members touch ankles together with the people standing next to them on both sides.
3. The whole group must walk as a team across the room without their ankles coming apart.

Time to Play a Game!

Team Building Exercises

1. Human Knot
2. Ankle Line Walk

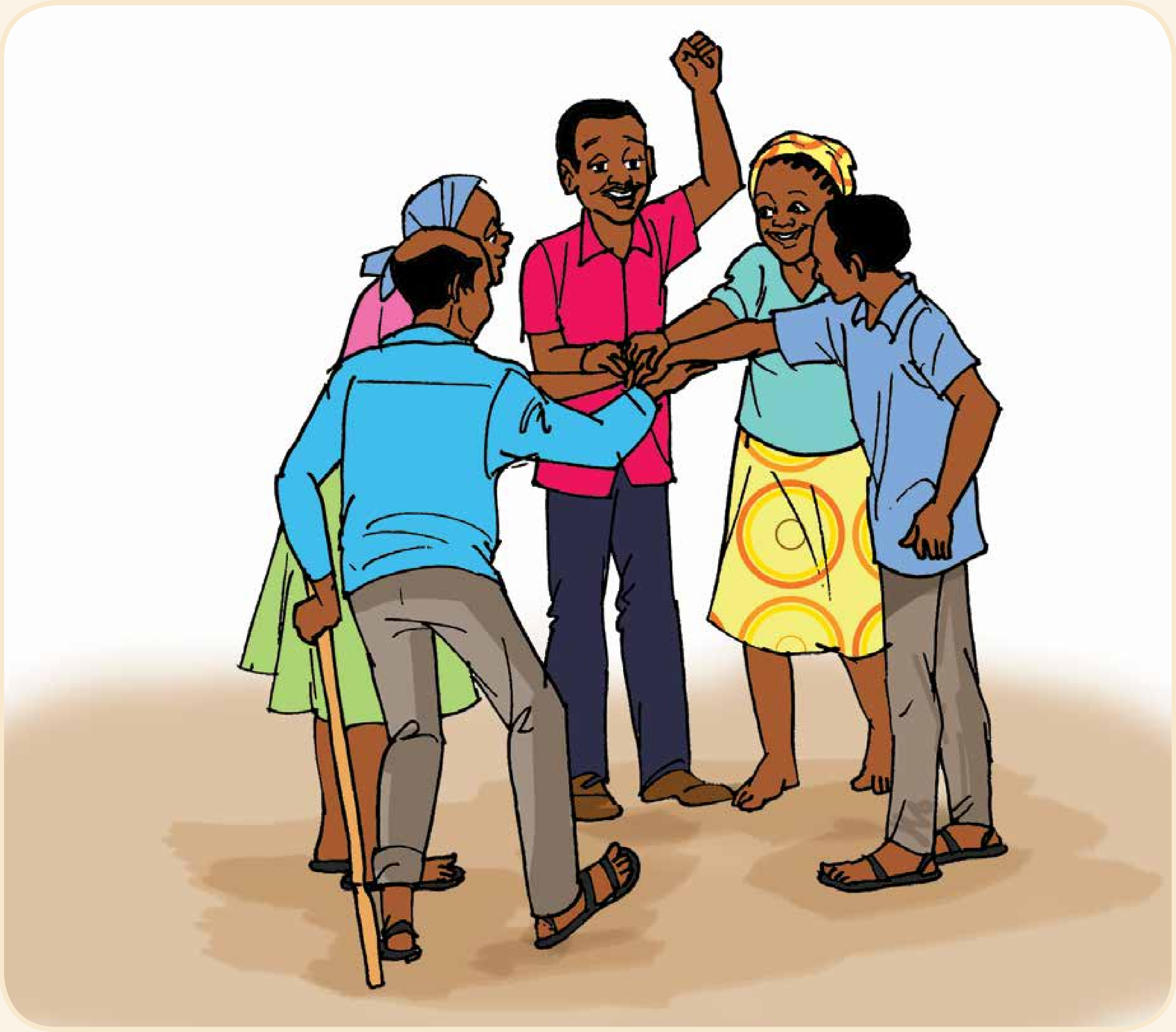

Facilitator's Guide

Let's Role Play!

Breach of Confidentiality at the Market

This exercise requires 3 volunteers to play the following roles:

Woman 1 – A vegetable seller

Woman 2 – A customer

Woman 3 – A person living positively with HIV in the same community

Woman 1, a vegetable seller, is arranging her vegetables and looks up as a customer approaches. They greet each other. They talk about the dry, hot weather and how the price of vegetables is higher this year. They notice a third woman who is buying vegetables from a different vendor nearby.

Woman 1 (seller): *"Wow, I haven't seen Mary in several months. I heard she was sick. She is looking very fat and healthy all of the sudden."*

Woman 2, a customer, whispers back to Woman #1:

Woman 2 (customer): *"Our children go to the same school. I heard from one of the teachers that she has HIV. I wonder how she got infected. She must be taking the drugs now."*

Woman 1 (seller): *"Wow. She's in my church but I didn't know that at all. My friends in church will be so surprised to hear about that."*

A third woman, woman 3, is a person living positively with HIV. She overhears the conversation and her eyes get big, but she says nothing and pretends not to be listening. She tries to hide the tears in her eyes.

After the role play, ask the group the following questions:

- Can anyone identify the 3 different breaches of confidentiality described in this role play?
  - A teacher told Woman 2 that Mary has HIV
  - Woman 2 told Woman 1 that Mary has HIV
  - Woman 1 plans to tell her friends at church
- What might be the consequences of these breaches?
- How can this be prevented?

Let's Role Play!

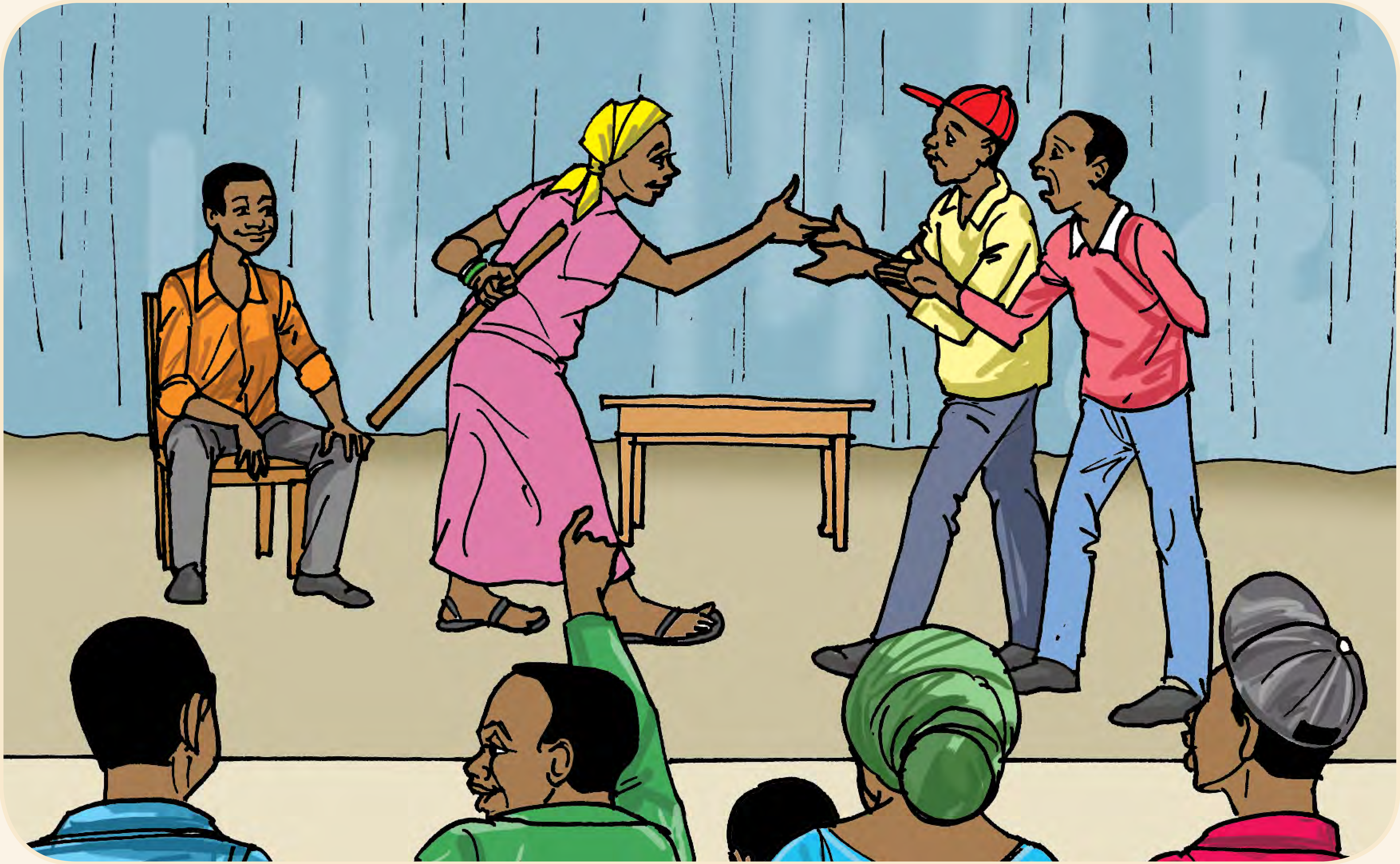

Facilitator's Guide

The Way Forward

Key message:

- Kanyaklas are health groups where people work together to support each other and maintain health in the community.
- 5 in 20 people in Homa Bay County are infected with HIV but we are all AFFECTED by HIV.
- Confidentiality is a key part of relationships and must be maintained.

Challenge: Find one Kanyakla member and discuss with him or her how you can help one another stay healthy. Also discuss how to help each other keep confidentiality.

Next Session: In the next session, we will discuss HIV biology and HIV around Lake Victoria.

Note: Remember to tell the group the time and place of the next session.

Remember:

I know something about HIV, I can do something about it, and I can do something for someone else affected by HIV and AIDS!

The Way Forward

Key message:

- Kanyaklas are health groups where people work together to support each other and maintain health in the community.
- 5 in 20 people in Homa Bay County are infected with HIV but we are all AFFECTED by HIV.
- Confidentiality is a key part of relationships and must be maintained.

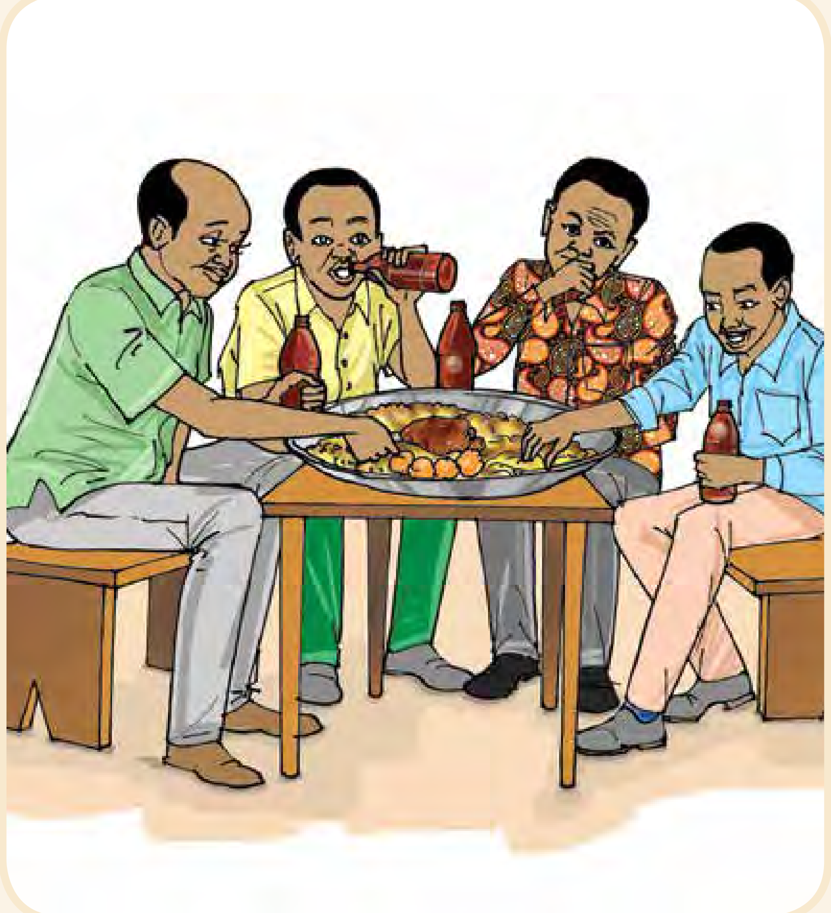

Challenge

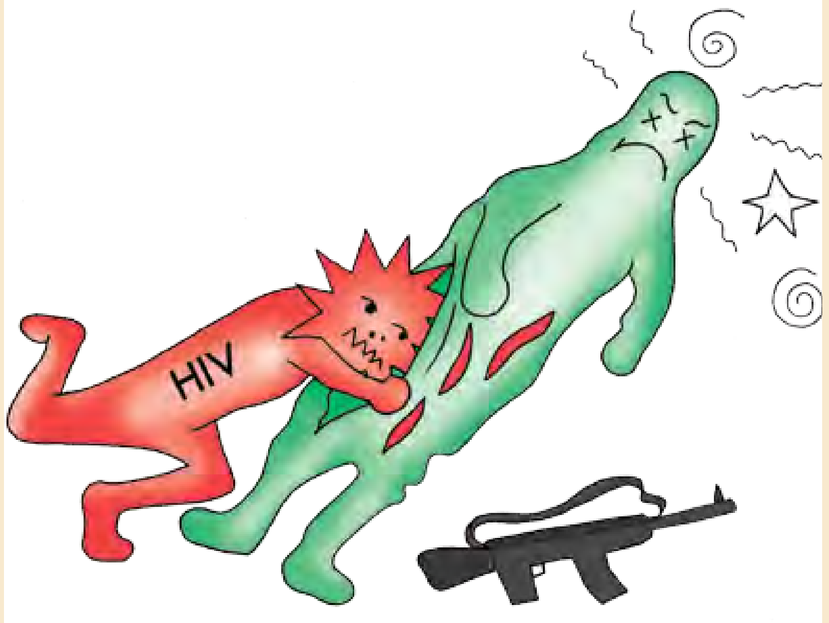

Next Session

I know something about HIV, I can do something about it, and I can do something for someone else affected by HIV and AIDS!

Facilitator's Guide

HIV on Lake Victoria

Objectives:

- Know the HIV statistics in Sub-Saharan Africa, Kenya, and Homa Bay County.
- Understand what HIV is and how it is spread.
- Understand ways to protect yourself from HIV and keep yourself healthy if you have HIV.

Session overview (100 minutes)

Activity	Time (minutes)	Objectives
Prayer and meditation	20	• Gather the group together and pray for a good session.<br>• Have the group reflect on the meditations listed below.
Review previous session	15	• Review the material from the previous session.<br>• Use questions and the key messages.
Class - Teach from the flipbook	20	• Give the group the statistics about HIV in Sub-Saharan Africa, Kenya, and Homa Bay County.<br>• Discuss HIV biology.
HIV games	30	• Use the games to demonstrate what HIV is, how it is transmitted, and how it hides in the body.<br>• You will need paper and markers to make posters.
Group discussion	5	• Discuss how HIV can be prevented through safe practices.
Wisdom circle	10	• Review the objectives of the session. What was learnt?<br>• Distribute a handout for the Kanyakla if it is provided.<br>• Schedule the next session.

Meditations:

"Accepting the possibility of death—honestly and completely—frees up a whole lot of energy to live." – ***Keith Gann***

"I am living with AIDS, not dying with AIDS." – ***Justice Edwin Cameron***

"It is a common experience that a problem difficult at night is resolved in the morning after the committee of sleep has worked on it."– ***John Steinbeck***

Session Two: HIV on Lake Victoria

1 2 3 4 5 6 7 8

Objectives:

- Learn the HIV statistics in Sub-Saharan Africa, Kenya, and Homa Bay County.
- Understand what HIV is and how it is spread.
- Understand ways to protect yourself from HIV and keep yourself healthy if you have HIV.

I know something about HIV, I can do something about it, and I can do something for someone else affected by HIV and AIDS!

Facilitator's Guide

HIV in Africa: The Scope of the Problem

Discuss HIV statistics in Sub-Saharan Africa, Kenya, and Homa Bay County.

- In Sub-Saharan Africa, 3% of adults are HIV-positive (about 3 out of 100)
- In Kenya, 6% of adults are HIV-positive (about 6 out of 100)
- In Homa Bay County, 27% of adults are HIV-positive (27 out of 100)

Ask the group: Raise your hand if you know someone living with HIV.

We are all **AFFECTED** by HIV.

HIV in Africa: The Scope of the Problem

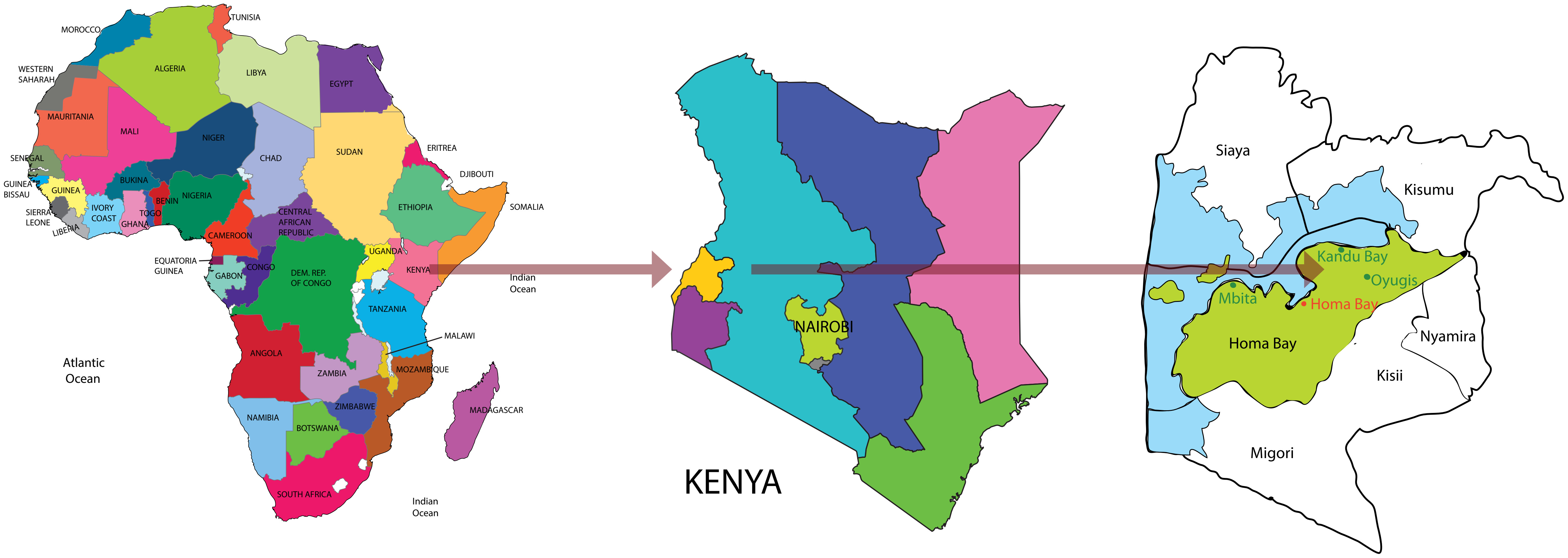

Africa:

25 million people living with HIV/AIDS

3 of 100 adults HIV+

Kenya:

1.6 million people living with HIV/AIDS

6 of 100 adults HIV+

Homa Bay County:

146,700 people living with HIV/AIDS

27 of 100 adults HIV+

Facilitator's Guide

What is HIV?

Ask someone in the group to explain the picture. If the participants still need clarity, explain the image to the group using the following description.

Think of your body as a house. Your house needs to have strong supports. The CD4 cells are your body's supports. They protect your body against illnesses. If your house's supports are strong, sickness will come but it will not knock your house down.

However, when a person has HIV, it is like termites are eating the house's supports. Eventually, the termites eat so much of the supports that the house becomes weak. Illness can now knock the house over.

HIV: How it spreads and how we can stay safe

Discuss each underlined word below.

- Human – HIV can spread from human to human. HIV can attack anyone of any race, any sex, any age, or any profession.
- Immunodeficiency – HIV attacks the immune system, leaving us vulnerable to infections and diseases.
- Virus – HIV is a virus, meaning it is very small. It cannot be seen with the naked eye.
- Treatable – It cannot be cured, but it can be treated with medications.
- Chronic disease – Much like diabetes or asthma, HIV is a chronic (lifelong) disease that can be managed with help from the doctor and the community.

If time allows, bring up some myths about HIV, and compare them to what we know is true about HIV.

What is HIV?

Human Immunodeficiency Virus

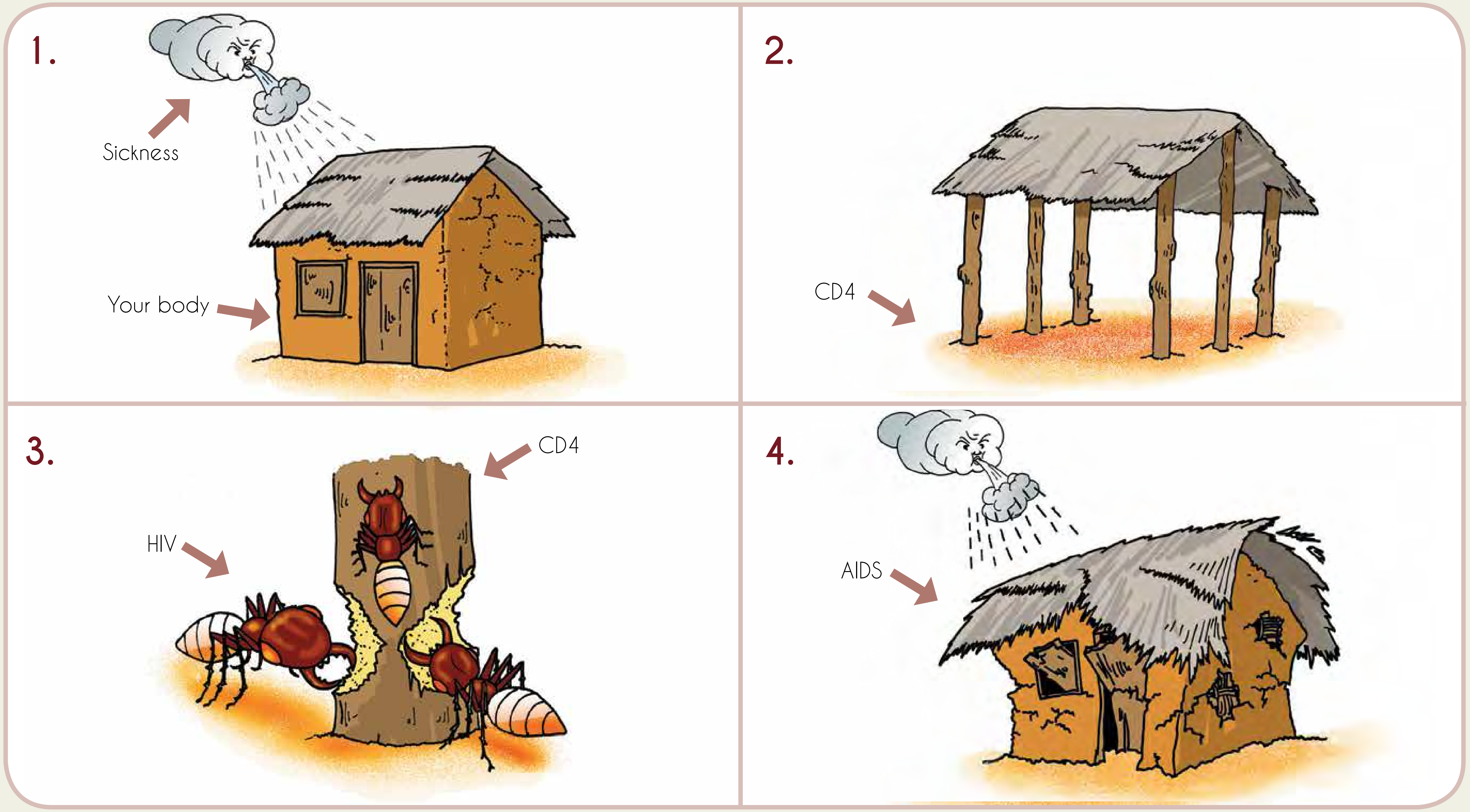

Facilitator's Guide

How HIV Works in the Body

Tell the group that you are going to play a game where everyone learns how HIV works in the body. For this game you need 2 presenters and 4 volunteers from the group.

Instructions:

1. Ask for 4 volunteers from the audience and give each a poster for the BODY, CD4 CELLS, CANCER, and TB.
2. Line the volunteers up in front of the audience. Present the BODY person
   and the CD4 CELLS person.
   - **Say:** The BODY is what we are trying to protect.
   - **Say:** CD4 CELLS are the main "askaris" or guards of the immune system. They give orders to other
     cells in the immune system. They protect the BODY.
3. Have the CD4 volunteer stand in front of the BODY volunteer and look menacing.
4. Have theTB and CANCER persons stand away from the BODY and CD4 volunteers.
5. Have the TB and CANCER volunteers attack the body. The CD4 CELLS person fights them off. TB and CANCER run back to their part of the stage.
   - **Say:** The body's defenses are strong.
6. Hang the HIV poster around your own neck.
   - Say: HIV is a virus that attacks CD4 cells and destroys them.
7. Beat on the shoulders of the CD4 CELLS person and have them get down on their knees to show they are dying/weak. As HIV, you raise your arms in a sign of strength.
   - **Say:** now the CD4 cells are destroyed, diseases like TB and CANCER can enter the body easily.
8. Have TB and CANCER attack the BODY. They run over and pound on the BODY, driving him down to his knees. The BODY is weak, CD4 is weak. Have TB, CANCER, and HIV dance around in triumph.
   - **Ask:** What is this condition called?
   - **Say:** This is called AIDS. AIDS is not a separate disease but a condition where other diseases like TB and cancer can attack the body. Without help, this person will die.
9. Now have the second presenter place the ARVs poster on his neck.
   - **Say:** ARVs are medications that put HIV in prison and keep it from killing CD4 CELLS.

10.Have the ARV person pound on HIV until they are on their knees.
   - ARVs cannot destroy HIV but can slow them down and can keep them in prison.

11. As HIV goes down, tell the CD4 volunteer to stand up.
    - **Ask:** why is CD4 getting stronger again?
12. As CD4 stands up, it fights off TB and CANCER who run to the other side of the room.
13. Then BODY stands up.
    - **Ask:** As CD4 gets strong the body gets strong. Why is that?
    - **Say:** This shows a person who has HIV but is living positively. They are strong. They can work and have a family. They are a Person Living With HIV (PLWH).

14 Collect the posters and thank the volunteers.

Bonus (If time allows)

- **Say:** Now that the body is feeling strong, he decides to start selling his medicine to a friend.
- **Ask:** What do you think will happen?

15. The ARV person backs away from the BODY, and HIV rises up and starts attacking CD4 again. The BODY person starts getting sick again, and begins to take his medicines. This time he does not start feeling well as quickly.
    - **Ask:** Why is it taking him longer to feel well?

Time to Play a Game!

Team Building Exercises

- How HIV Works in the Body

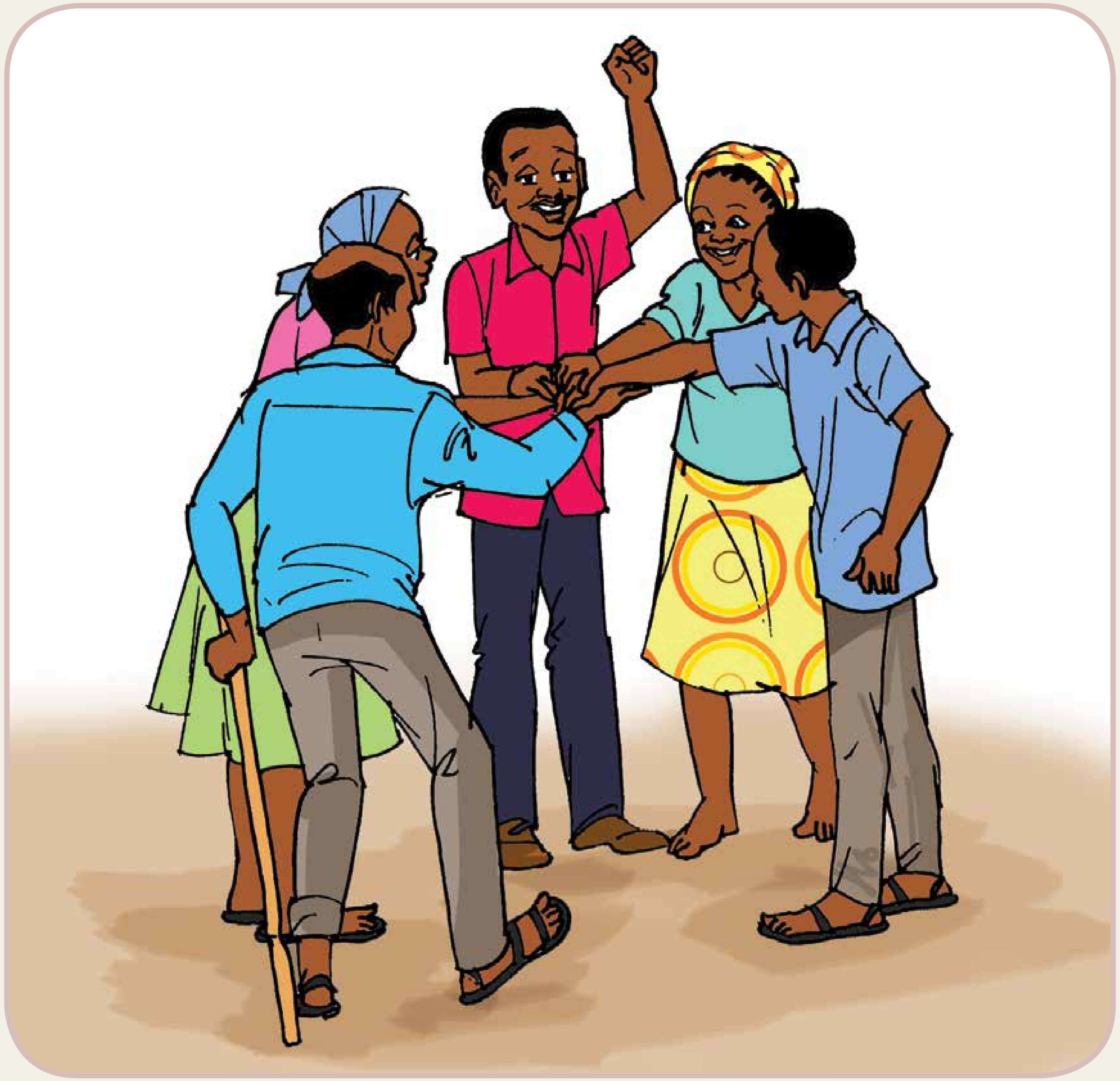

Facilitator's Guide

HIV Fluids

This game teaches the group how HIV is passed from person to person.

Instructions:

1. Ask for 8 volunteers (at least 2 women, if possible) from the audience. Line them up in front of the group.
2. Place 4 chairs at intervals behind the 8 volunteers. Give one of the following body fluid posters to each person and have them put it around their neck: SWEAT, SALIVA, SEMEN, BLOOD, VAGINAL FLUID, BREAST MILK, URINE
   - **Ask:** What do all these things have in common? **Answer:** They are all body fluids.
   - **Ask:** What body fluids can transmit HIV? Give the group a chance to answer.
3. Stand behind each volunteer and ask the audience, "Can this body fluid transmit HIV?" Discuss each fluid as needed.
4. If the body fluid CANNOT transmit HIV, have the person sit down in their chair. If the fluid CAN pass HIV, have the volunteer keep standing.

   The ones that CAN transmit HIV are: SEMEN, BLOOD, VAGINAL FLUID and BREAST MILK.
5. Have the audience repeat the 4 body fluids that can pass HIV (BLOOD, SEMEN, VAGINAL FLUID, and BREAST MILK.).
6. Ask for questions from the group.
7. Ask the group: Which of the following situations can result in you getting HIV?
   a. You kiss an HIV-positive person. [Answer: NO, you cannot get HIV from saliva.]
   b. You hug a sweaty HIV-positive person. [Answer: NO, you cannot get HIV from sweat.]
   c. You get a needle puncture when taking blood from a person whose HIV status is unknown. [Answer: YES, you can get HIV from blood.]
   d. You have unprotected sex with an HIV-positive person. [Answer: YES, you can get HIV from vaginal fluid/semen.]
   e. A boy is circumcised using a common knife. [Answer: YES, you can get HIV from blood.]
   f. You sit on a toilet seat that has urine on it. [Answer: NO, you cannot get HIV from urine.]
   g. A baby is born and breastfed by an HIV-positive mother [Answer: YES, you can get HIV from blood, vaginal fluid, or breast milk.]
9. Ask for questions. Thank the volunteers.

How HIV Hides (Ball Game)

1. Ask for 6 volunteers from the audience
2. Give each volunteer one of 6 posters: YOUR CHIEF, YOUR GIRLFRIEND, YOUR BOYFRIEND, YOUR SISTER, YOUR BROTHER, A COMMERCIAL SEX WORKER.
3. Have the 6 volunteers line up, shoulder-to-shoulder, facing the audience.
   - **Say:** The volunteers are going to pass a small ball behind their backs, hand to hand, and try not to let the audience know who has the ball.
4. Give the ball to the person at the end of the line.
   - Say: Watch closely and try to determine who has the ball.
5. Give the volunteers a minute to pass the ball, then ask them to stop.
6. Ask the audience who has the ball. If they cannot guess it, have the person with the ball hold it up.
   - **Ask:** What does this game have to do with HIV? [The answers are: (1) You cannot tell by looking at a person who has the virus and who does not. (2) Anyone can have the virus. (3) The virus can pass easily from person to person.]
   - **Ask:** What is the only way to tell who has the virus? [Answer: Get tested]
7. Discuss the importance of getting tested to know your own status.

Time to Play a Game!

Team Building Exercises

- HIV Fluids
- How HIV Hides (Ball Game)

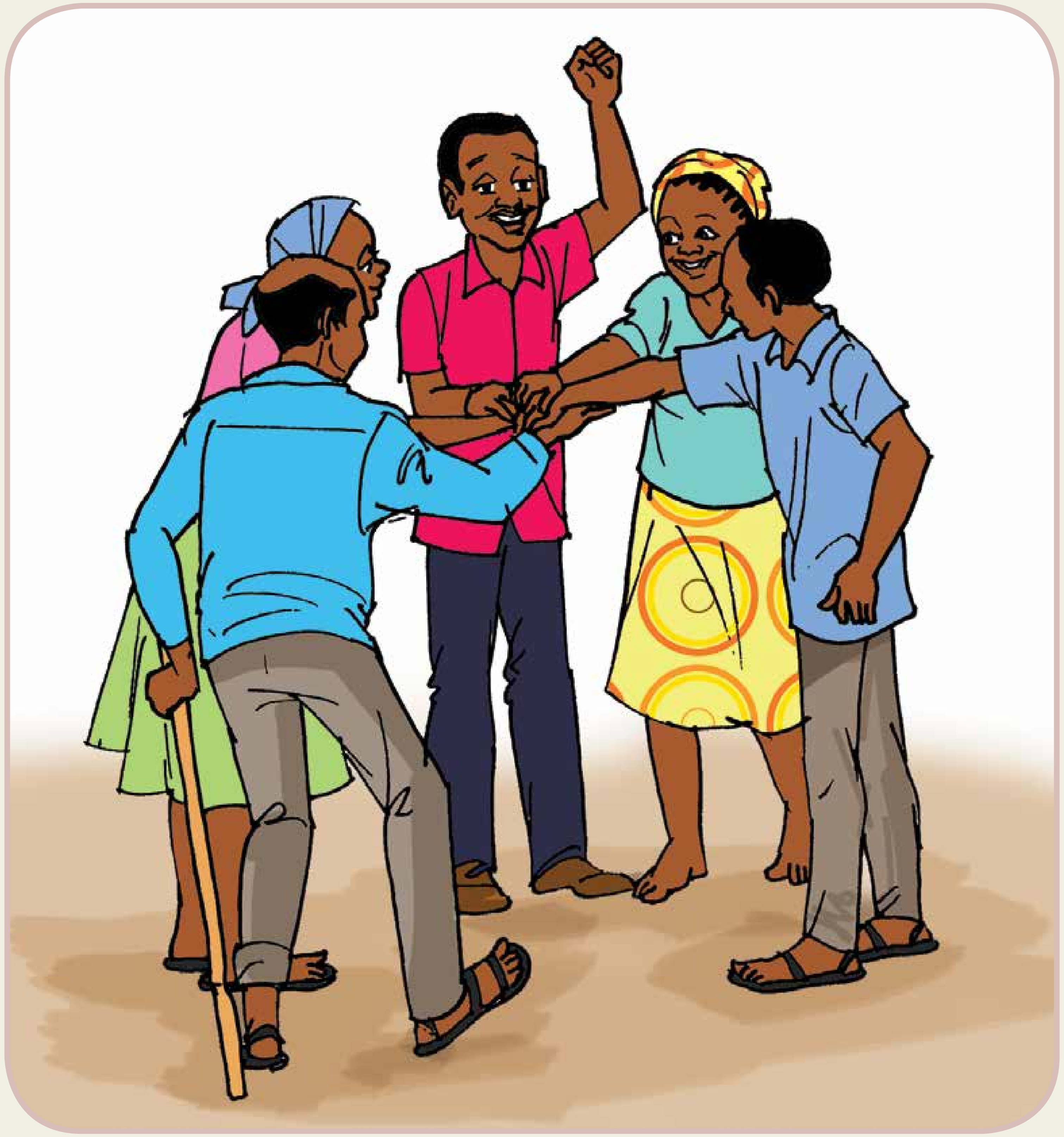

Review Questions for Games:

1. Who can explain how HIV weakens the body?
   - **Answer:** HIV attacks CD4 cells and weakens the immune system. Then other infections can come in to make a person sick.
2. Who can explain how ARVs work to keep a person healthy?
   - **Answer:** ARVs prevent HIV from growing within the body, though they cannot stop HIV completely.
3. Which fluids transfer HIV from person to person?
   - **Answer:** Blood, semen, vaginal fluid, breast milk
4. How can you tell if someone has HIV?
   - **Answer:** You can't tell by looking at them! The only way to know if someone has HIV is to see their test results.

Team Building Exercises

- Let's review!

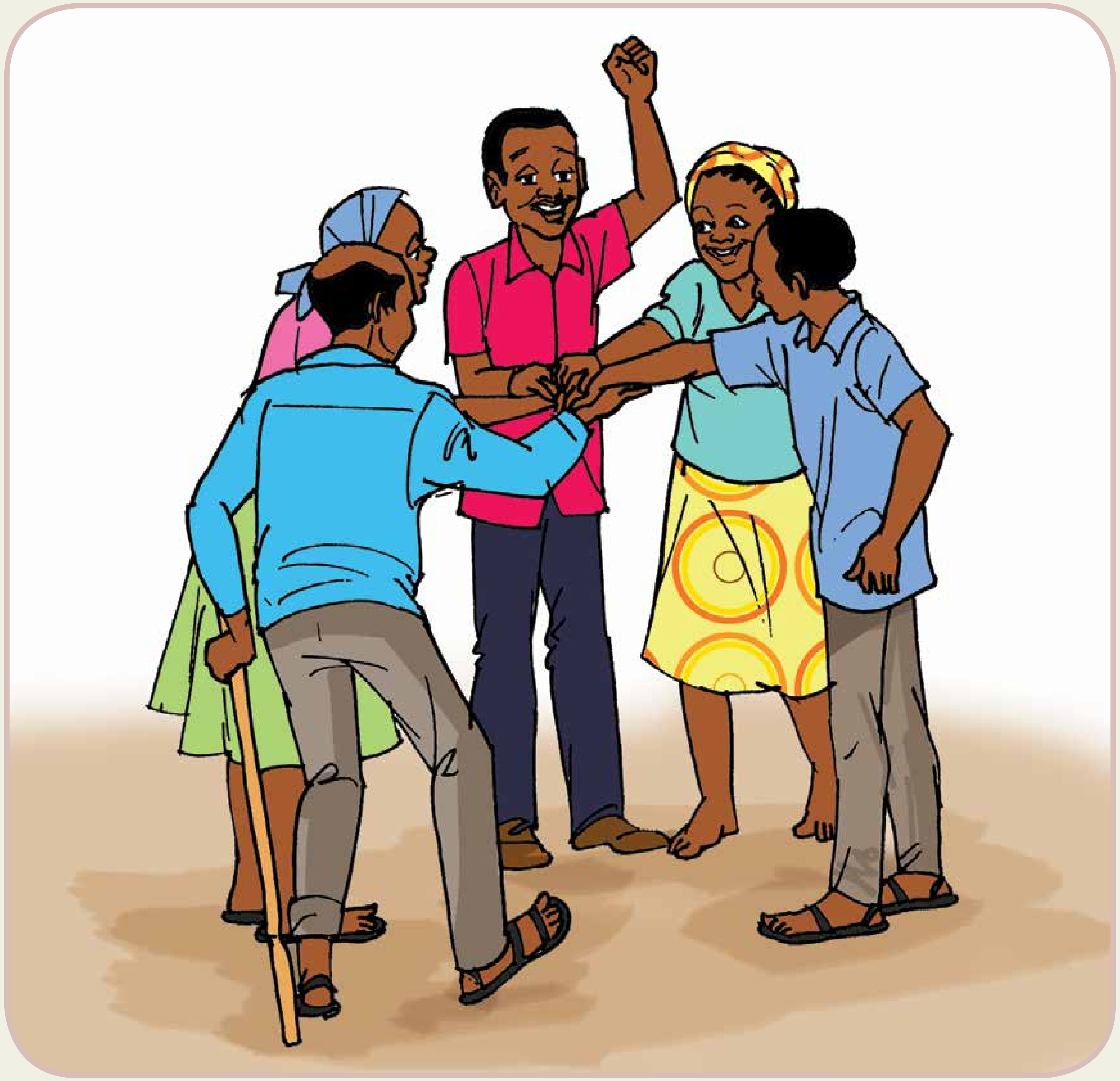

HIV Transmission and Safe Practices

This session calls for group participation.

- Get the group to name 3 ways HIV can be transmitted.
- Write the answers on the left of each of the boxes.
- Next, ask about ways to prevent infection or stay healthy from each of these possible means of transmission.
- Write the answers on the right side of the blue boxes.

HIV Transmission and Safe Practices

HIV is a virus spread from person to person through unprotected sex, Below are the suggested answers. Use them to fill in answers not mentioned by the group.

Mode of Transmission:

- Unprotected sex

How it can be prevented:

- Consistent condom use
- Get tested
- Have partners get tested
- Take ARVs to keep viral count low
- Get educated about the risks of unprotected sex

Mode of Transmission:

- Contaminated blood

How it can be prevented:

- Wear gloves if you have a cut
- Use clean knives for circumcisions/other surgical procedures
- If you think you may have been exposed, seek medical care

Mode of Transmission:

- Mother-to-child

How it can be prevented:

- Know your HIV status
- Take ARVs during pregnancy and breastfeeding (and always)

HIV Transmission and Safe Practices

Facilitator's Guide

The Way Forward

Key message:

- 5 in 20 people in Homa Bay County are HIV-infected. We are all AFFECTED by HIV.
- HIV is spread from person to person through unprotected sex, contaminated blood and mother-to-child TRANSMISSION.
- There are many ways to stay healthy: know your status, seek treatment, reduce the risk, get proper nutrition.

Challenge: Discuss with your partner or another member of the group one way you and your partner can reduce your risk of transmitting HIV and report back to the group.

Next Session: The next session will cover HIV medications: How they work and why they should be taken regularly.

Note: Remember to tell the group the time and place of the next session.

Remember:

I know something about HIV, I can do something about it, and I can do something for someone else affected by HIV and AIDS!

The Way Forward

Key message:

- 5 in 20 people in Homa Bay County are HIV-infected. We are all AFFECTED by HIV.
- HIV is spread from person to person through unprotected sex, contaminated blood and mother-to-child TRANSMISSION.
- There are many ways to stay healthy: know your status, seek treatment, reduce the risk, get proper nutrition.

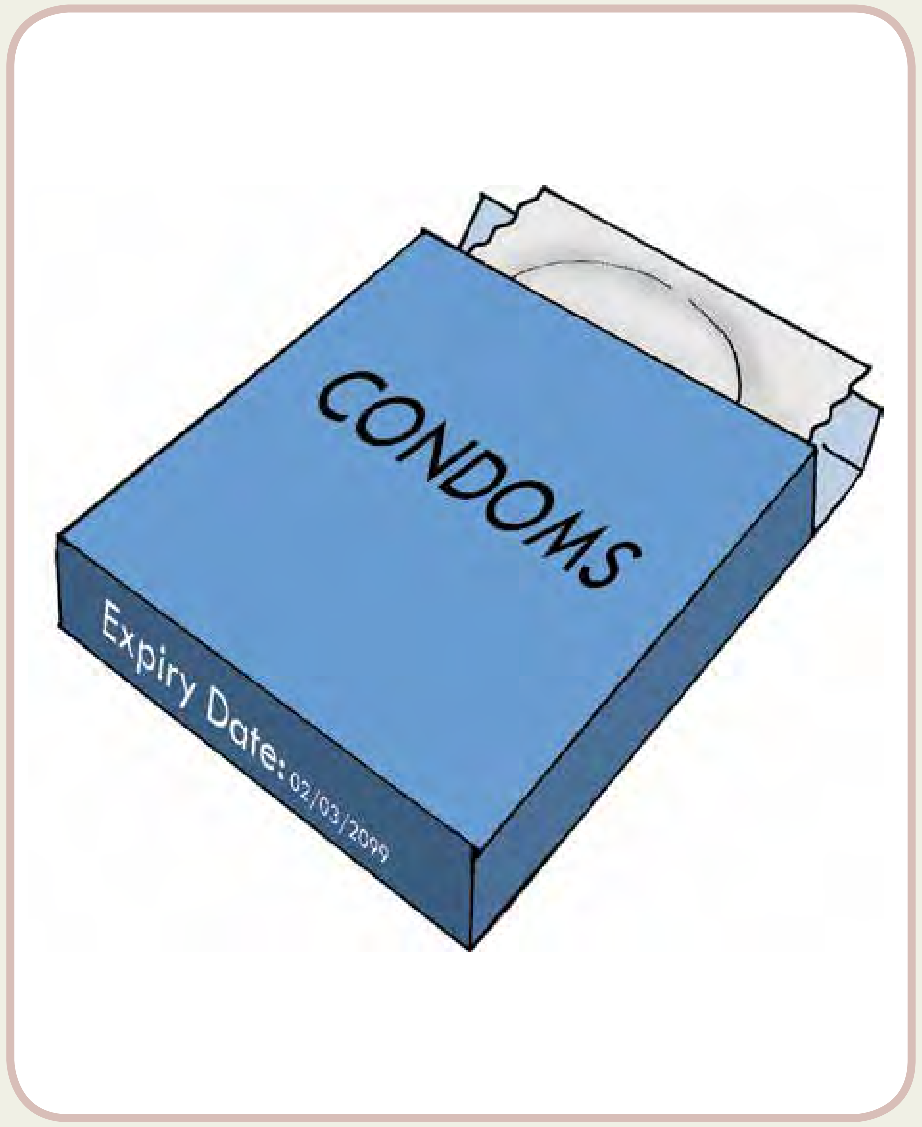

Challenge

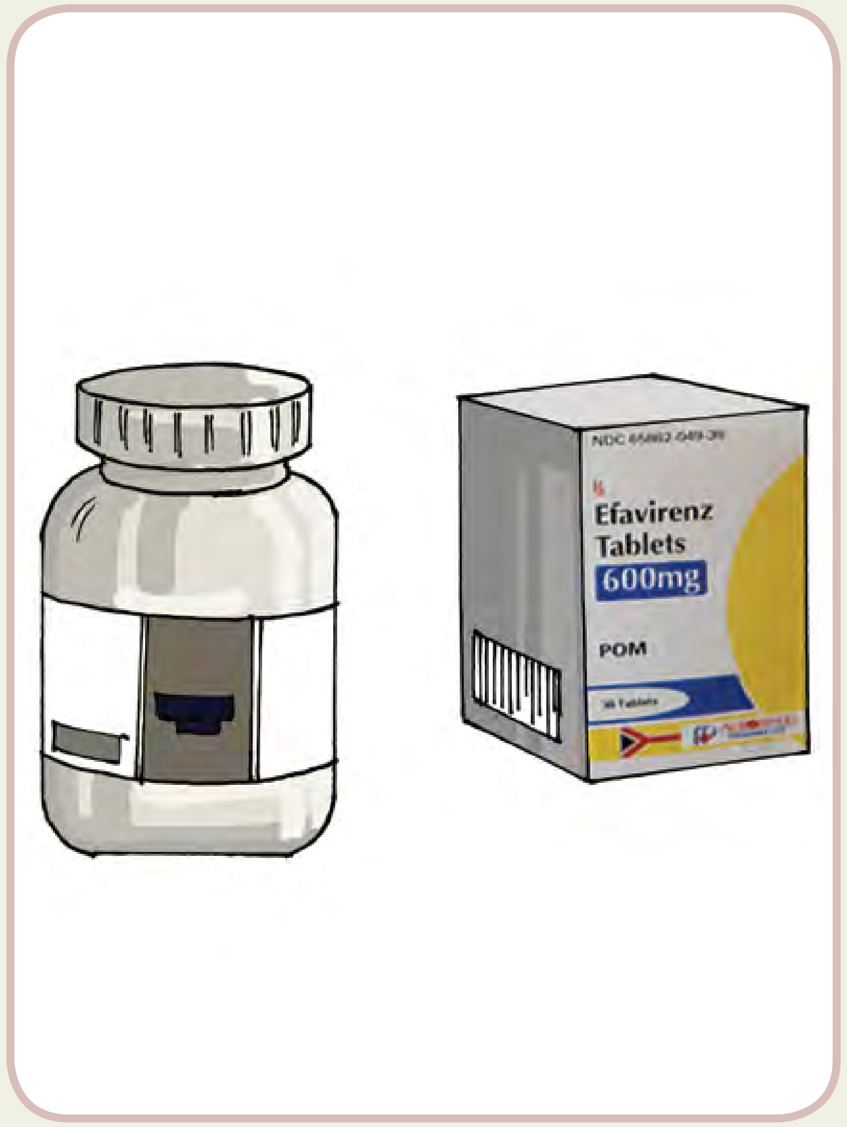

Next Session

I know something about HIV, I can do something about it, and I can do something for someone else affected by HIV and AIDS!

Facilitator's Guide

Combination Antiretroviral Therapy

Objectives:

- Understand how HIV takes over a cell.
- Learn how ARVs work to fight the HIV virus.
- Understand the importance of taking medication regularly.

Session overview (125 minutes)		
**Activity**	**Time (minutes)**	**Objectives**
Prayer and meditation	20	• Gather the group together and pray for a good session.<br>• Have the group reflect on the meditations listed below.
Review previous session	15	• Review the material from the previous session.<br>• Use the questions and key messages to guide the review.
Class - Teach from the flipbook	20	• Explain how HIV takes over a cell.<br>• Explain how ARVs work.<br>• Discuss the importance of taking medicine regularly.
HIV games	25	• Review how HIV attacks the cell and how ARVs work.
Group discussion	10	• Discuss the side effects of ARVs.
Class - Teach from the flipbook	20	• Learn how HIV can become resistant to ARVs if they are not taken properly.<br>• Learn the importance of adhering to ARVs.
Wisdom circle	15	• Review the objectives of the session. What was learnt?<br>• Distribute a handout for the Kanyakla if it is provided.<br>• Schedule the next session.

Meditations:

"If the total integrated system of mind, body and emotions, which constitutes the whole person, is not working in the direction of health, then purely physical interventions may not succeed." – *Carol and Stephanie Simonton*

"The immune system is affected positively and negatively by thoughts and feelings." – *Mark Friedlander and Terry Phillips*

Session Three: Combination Antiretroviral Therapy

1 2 3 4 5 6 7 8

Objectives:

- Understand how HIV takes over a cell.
- Learn how ARVs work to fight the HIV virus.
- Understand importance of taking medication regularly.

I know something about HIV, I can do something about it, and I can do something for someone else affected by HIV and AIDS!

Facilitator's Guide

How HIV Works

This illustration demonstrates the steps HIV takes to enter a cell, take control of it, and produce more HIV viruses using three viral enzymes called Reverse Transcriptase, Integrase, and Protease.

Note: **These ideas will be reinforced through the HIV in the Body and the ARV game.**

1. HIV enters the CD4 cell.
2. Once inside the cell, HIV uses "Reverse Transcriptase" to make a copy of itself and trick the cell into thinking it belongs inside.
   - **Defense:** This is where the first type of ART drug works (called Reverse Transcriptase Inhibitor) to stop this from happening.
3. HIV then makes itself a permanent part of the CD4 cell's DNA using Integrase
   - **Defense:** This is where the next type of ART drug works (called Integrase Inhibitor).
4. Next, HIV tricks the cell into making many copies of the HIV virus using Protease. In the process, the cell makes a special coat to protect the virus.
   - Defense: This is where the third type of drug works (called Protease Inhibitor.)
5. Finally, the HIV virus, wrapped in its protein coat, leaves the cell and attacks other CD4 cells.
6. This process of producing more HIV viruses kills the CD4 cells.

Medication:

- It is important to take several different types of drugs to block HIV at multiple places.
- Sometimes ARVs come in pills with more than one type of drug in it, other times separate pills have to be taken.
- This is why people may have different numbers of pills they need to take.

Key message:

- There are several different ART drugs that can block HIV from multiplying.

How HIV Works

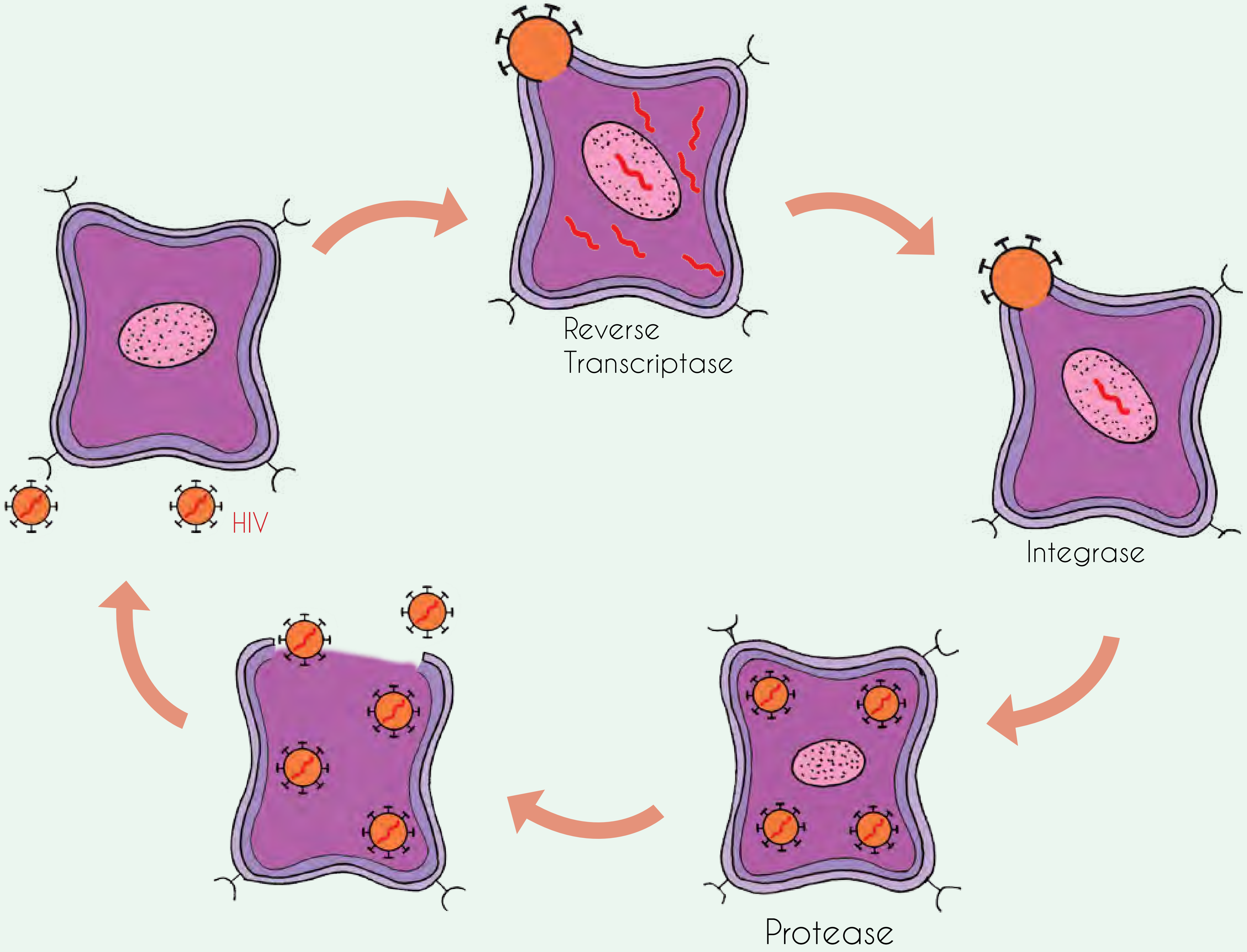

How ARVs Work:

This game will teach the group how ARVs work to protect the body from HIV. You will need:

- Placards: 1 CD4 DNA, 4 HIV VIRUS, 4 PROTEIN COAT, 1 HIV DNA, 3 ARVs.
- 1 mask
- 6-9 volunteers

Instructions:

1. Mark out a circle on the floor to represent the 'CD4 cell'.
2. Have 1 volunteer (CD4 DNA) stand in the cell.
3. 4 volunteers will stand outside cell, wearing both the 'HIV VIRUS' placard and the 'PROTEIN COAT' placard. Then 1 volunteer will take off his 'protein coat' placard and step into the cell.
4. The first thing HIV does after arriving into a cell is to disguise itself so that the cell thinks it's supposed to be there. The HIV VIRUS volunteer will put on a mask at this time to represent tricking the cell.
5. Next, the HIV virus incorporates some of its DNA into the cell's DNA – permanently taking up residence in the cell. At this time, the HIV VIRUS volunteer hands the CD4 DNA volunteer a HIV DNA sign to hold.
6. Next, the HIV virus makes many copies of itself. 3 other HIV VIRUS volunteers step into the cell and the original HIV VIRUS volunteer hands each of them a PROTEIN COAT sign.

ARVs can block each of these steps!

7. Repeat the steps above with 3 ARV volunteers making it difficult (but not impossible) for HIV to multiply. The ARV volunteers should:

    - Prevent HIV VIRUS from putting on the mask
    - Block HIV VIRUS from incorporating HIV DNA into the cell
    - Make it difficult for HIV VIRUS to make copies of itself
    - Keep HIV VIRUS from giving a PROTEIN COAT to the other HIV VIRUSES that are made

Time to Play a Game!

Team Building Exercises

- How ARVs Work

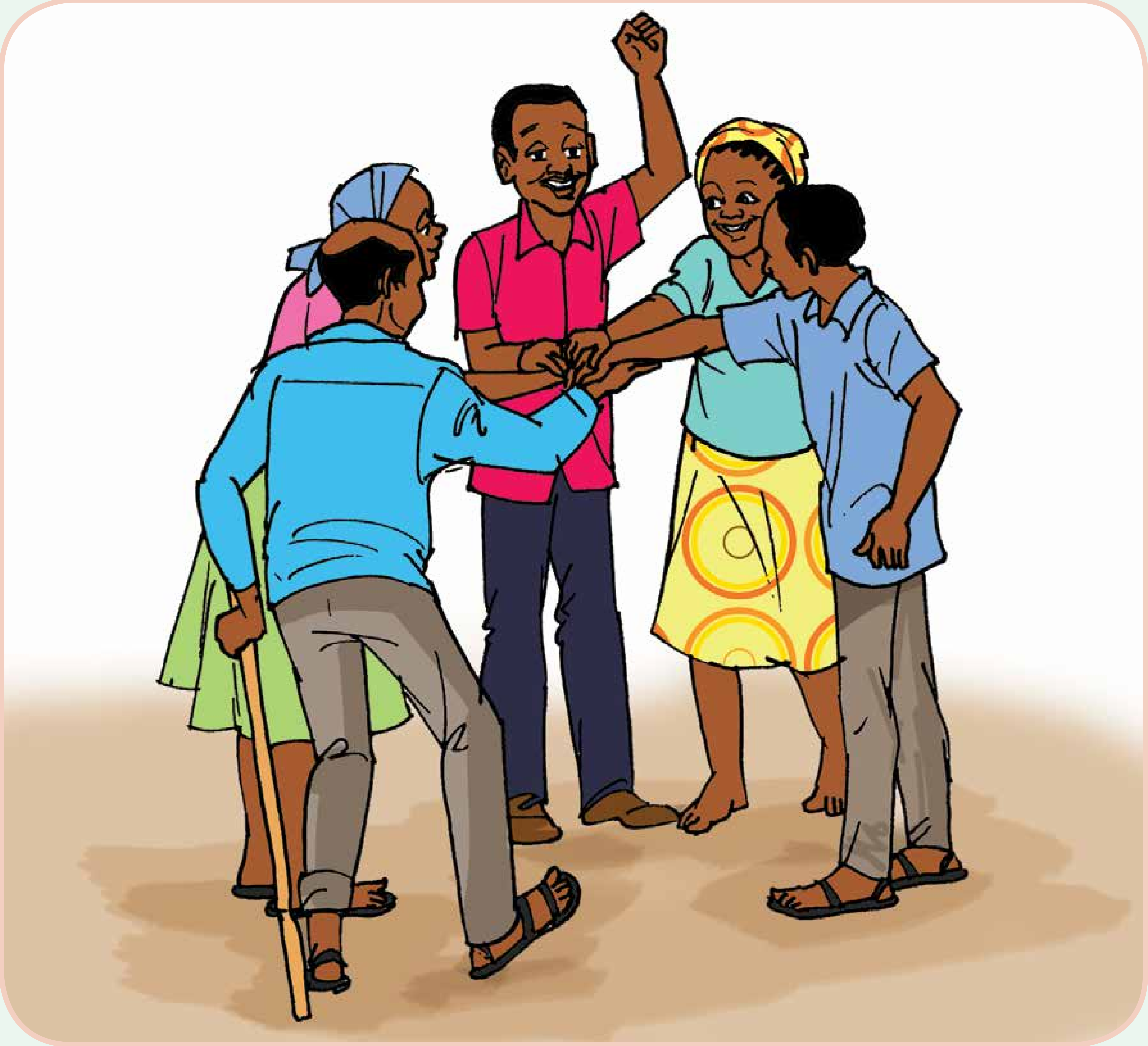

Facilitator's Guide

Side Effects of ARVs:

Use the following questions to guide a discussion on the side effects of taking ARVs. Discuss the real side effects and the imagined side effects learnt from rumours or unrealistic fears people have.

1. What are the side effects of ARVs?
2. What should I do if I'm having side effects?
    - Should you stop taking ARVs?
    - Should you stop just one drug?
    - What can happen if you go to the health centre?

Key message:

If someone is having side effects from ARV's it is important for them to do the following:

- Seek medical care from the clinic or hospital as soon as possible
- Do NOT stop taking your ARV's unless directed by a doctor or clinical officer
- Talk with friends and family who are living with HIV to learn more about their ideas for helping to manage side effects
- Take hope, these side effects often improve with time

Side Effects of ARVS

?

1. What are the side effects of ARVs?
2. What should I do if I'm having side effects?

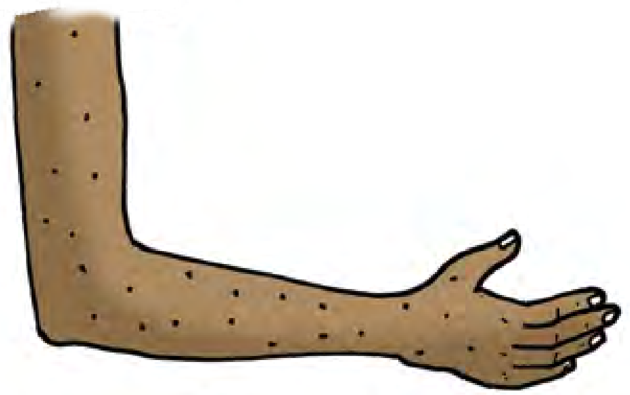

Skin rash

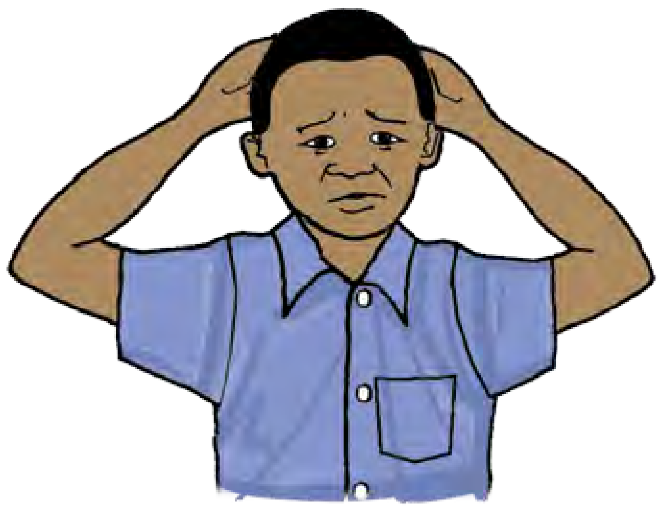

Headache

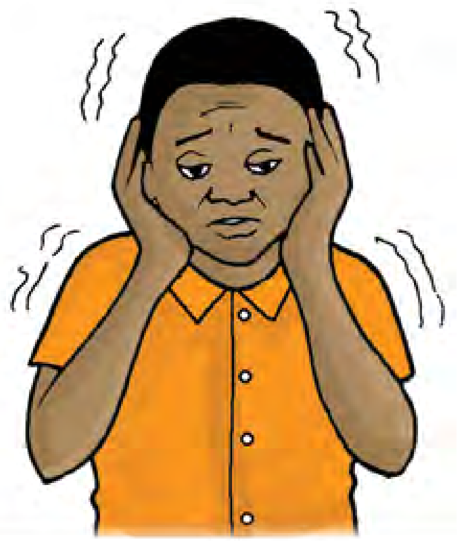

Fever

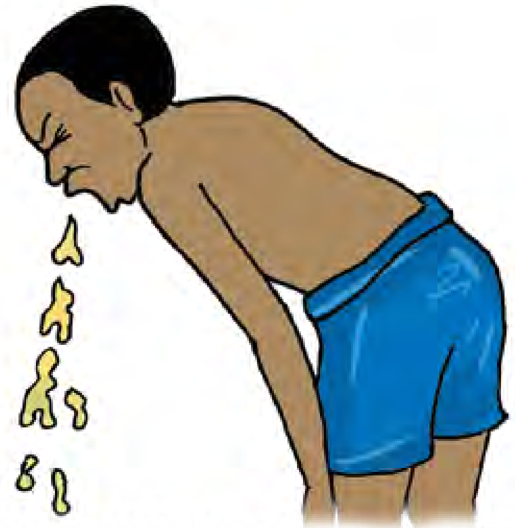

Vomiting

Facilitator's Guide

How Does HIV Become Resistant to ARVs?

The illustrations shows what can happen if someone stops taking their ARVs for a while. The HIV virus is able to mutate and then when the person starts ARVs again, the mutated HIV viruses will be able to get past the ARVs and attack cells. Taking 2 or 3 drugs at the same time makes it difficult for HIV to mutate and prevents resistance.

Explain the diagrams to the group.

1. (Top left) When you take all of your ARVs (usually 3 different drugs, though sometimes multiple drugs are put in a single pill), HIV is weakened and forced to stay asleep in your body.
2. When you miss some of your doses (top right), or you share some of your pills with others (bottom centre), HIV wakes up and begins attacking your body.
3. When you take only some of your doses, it is not enough to keep HIV asleep. This allows HIV to learn about how to fight back against your ARVs. Sometimes when you start taking the ARVs again, they don't work as well because HIV has learnt how to fight back. This is called 'drug resistance.'

Key message:

- Taking 3 or more different ARVs (sometimes multiple drugs are in one pill, but there are still 3 or more drugs) and never missing doses is the best way to prevent resistance and keep HIV from waking up within your body.

How Does HIV Become Resistant to ARVs?

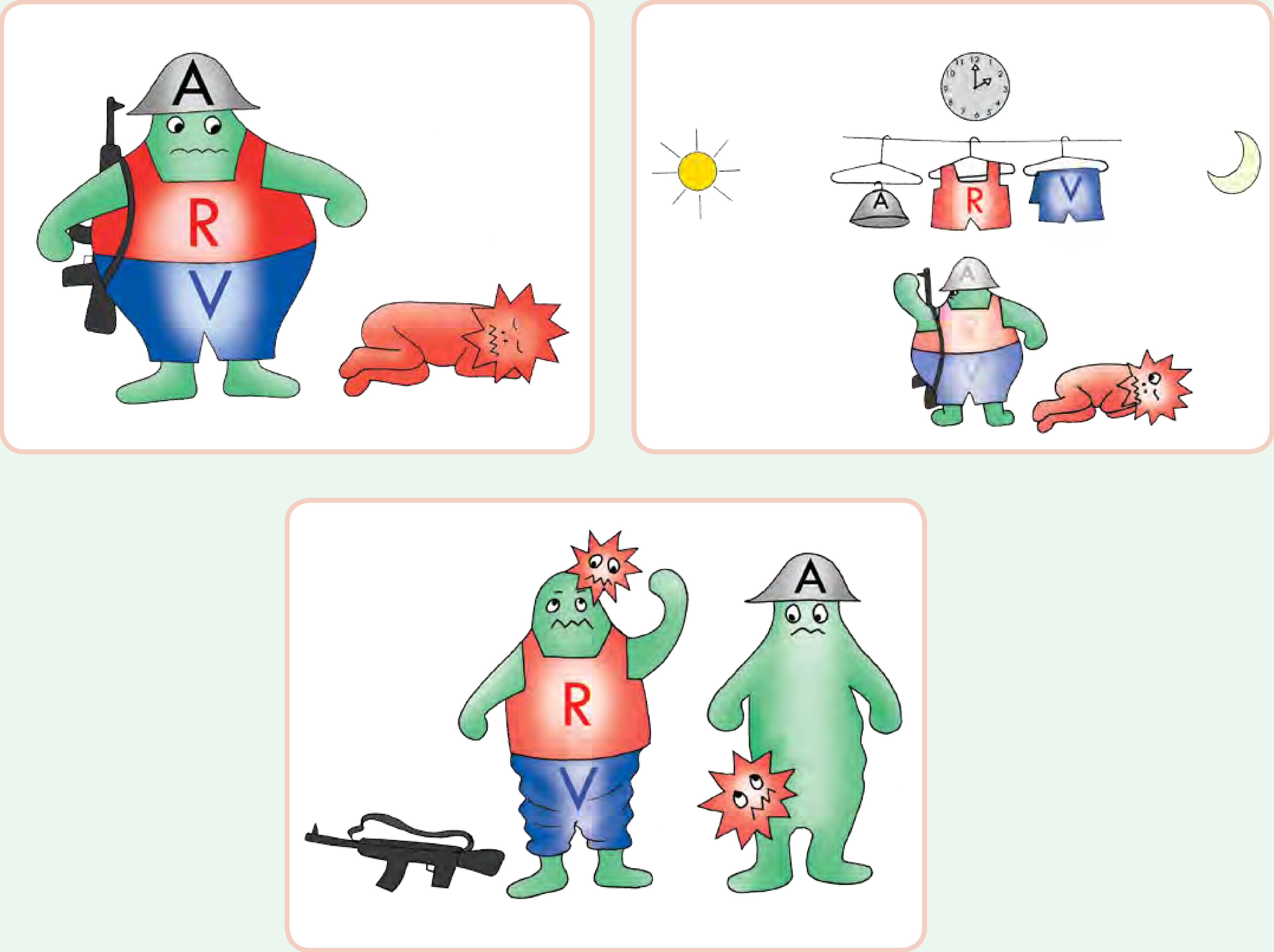

Facilitator's Guide

Medication Adherence

Using the questions below, guide a discussion with the group about why it can be difficult to take medications every day and strategies for remembering to take medications.

Brainstorm how Kanyakla members can help each other remember to take their medications.

Questions for the group:

1. Why do people find it difficult to take their medications every day?
2. Why is it important to take medications every day?
3. As a Kanyakla, what can we do to help each other take medications?

Medication Adherence

?

1. Why do people find it difficult to take their medications every day?
2. Why is it important to take medications every day?
3. As a Kanyakla, what can we do to help each other take medications?

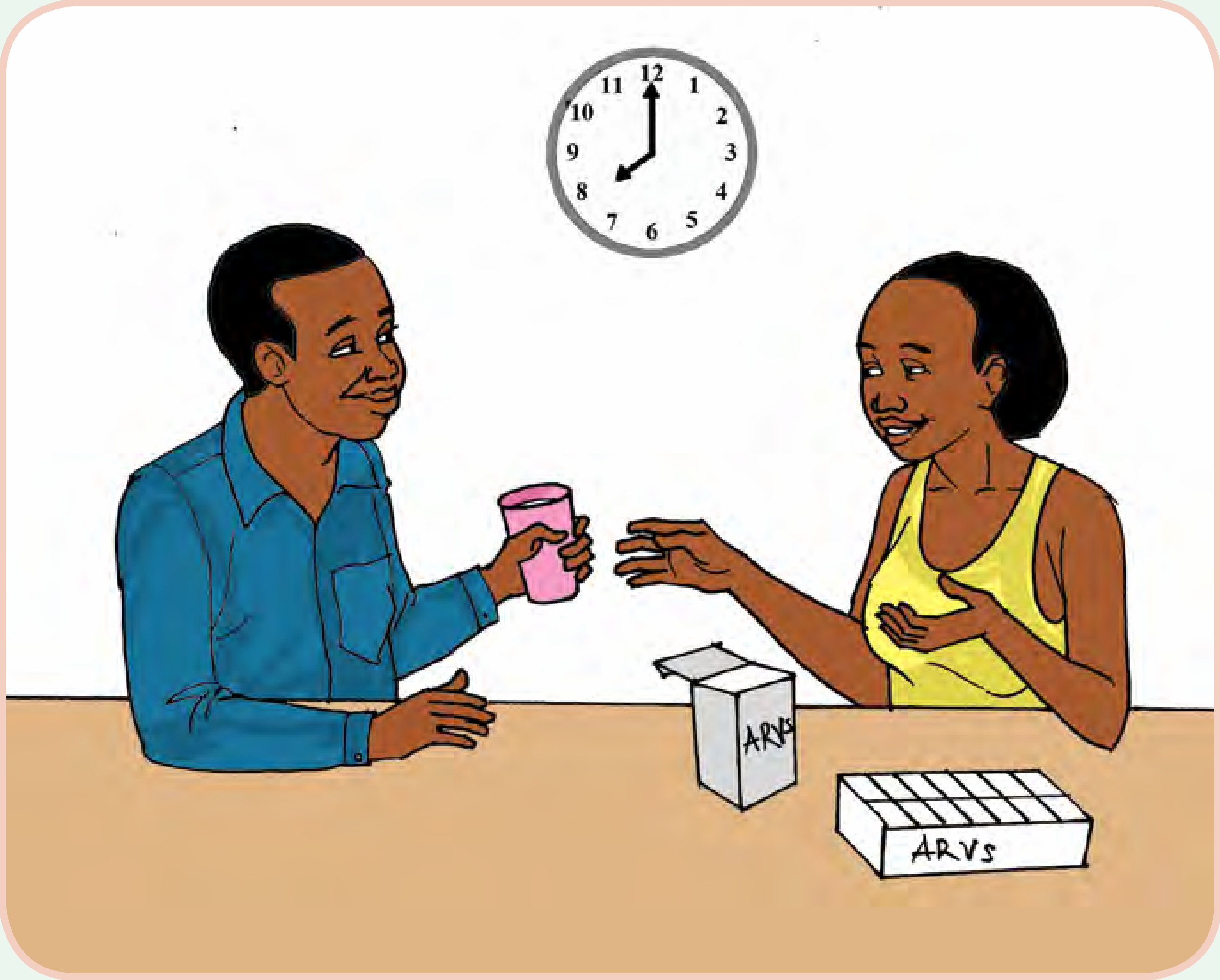

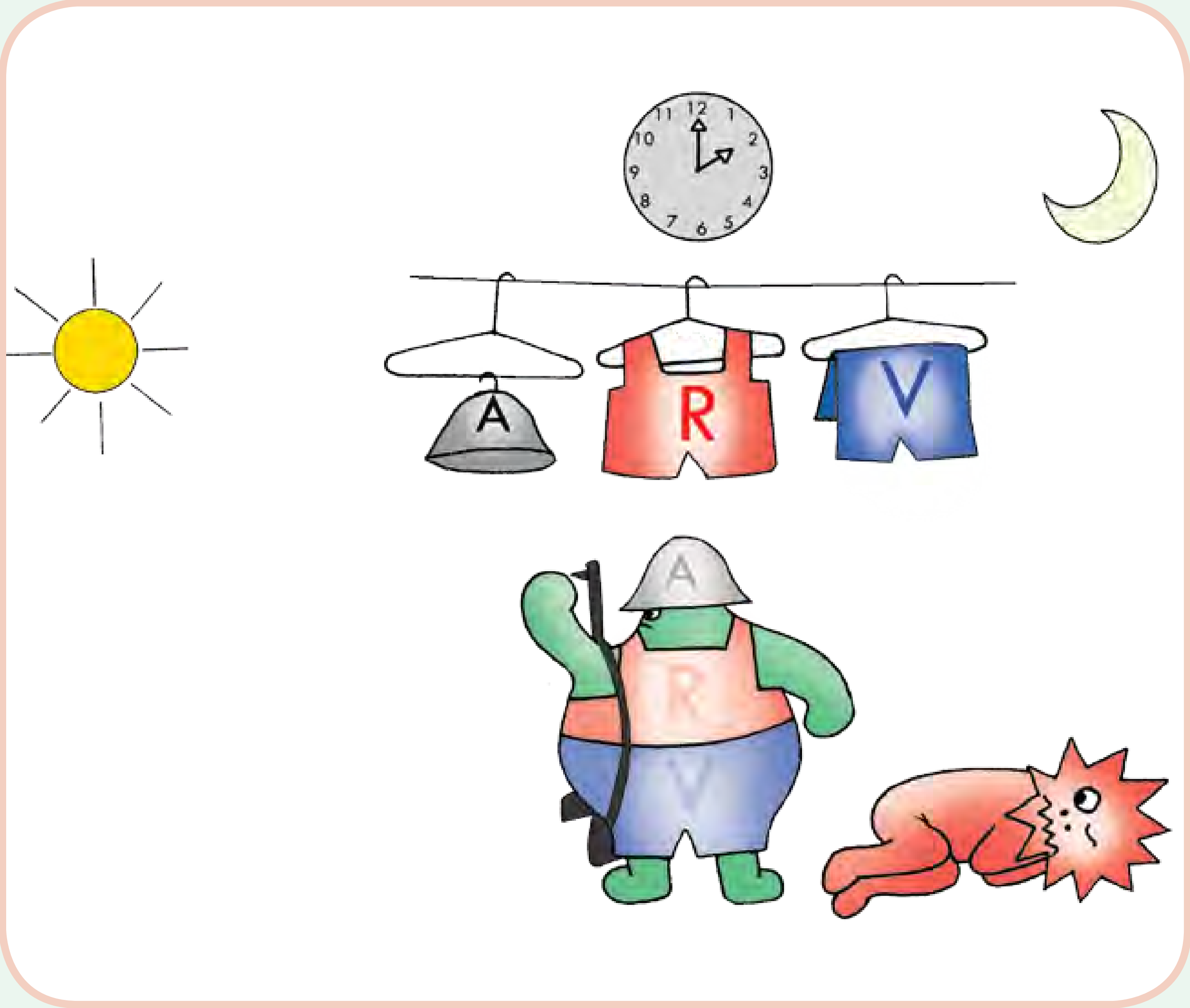

Facilitator's Guide

The Way Forward

Key message:

- ARVs work in multiple ways to prevent HIV from multiplying in your body
- People are often given multiple drugs to block HIV in different ways
- If you are having side effects, go to the clinic and they can change your medications
- Stopping medications can lead to the virus no longer responding to drugs (resistance)

Challenge: Talk to someone who is on medication about the importance of taking medicine as prescribed.

Next Session: The next session will be about Engaging with Care – being an active patient.

Note: Remember to communicate the time and place for the next meeting.

Remember:

I know something about HIV, I can do something about it, and I can do something for someone else affected by HIV and AIDS!

The Way Forward

Key message:

- ARVs work in multiple ways to prevent HIV from multiplying in your body
- People need to take multiple drugs to block HIV in different ways
- If you are having side effects, go to the clinic and they can change your medications
- Stopping medications can lead to the virus no longer responding to drugs (resistance)

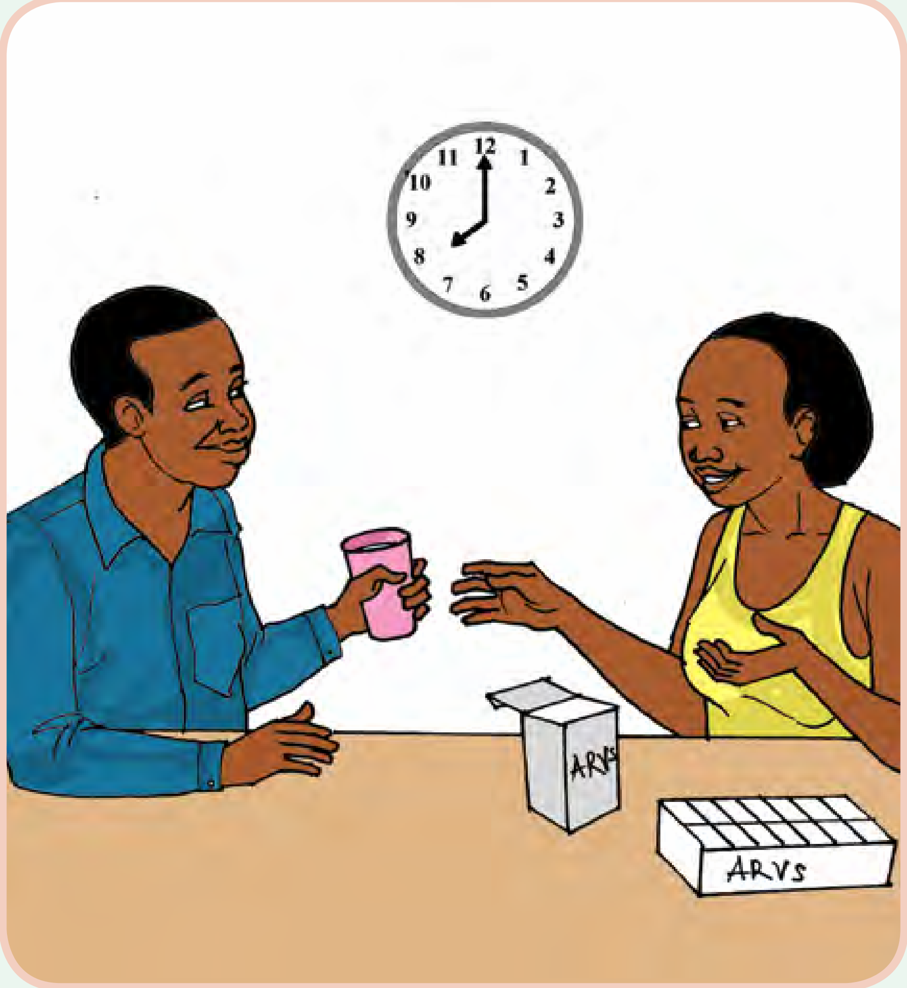

Challenge

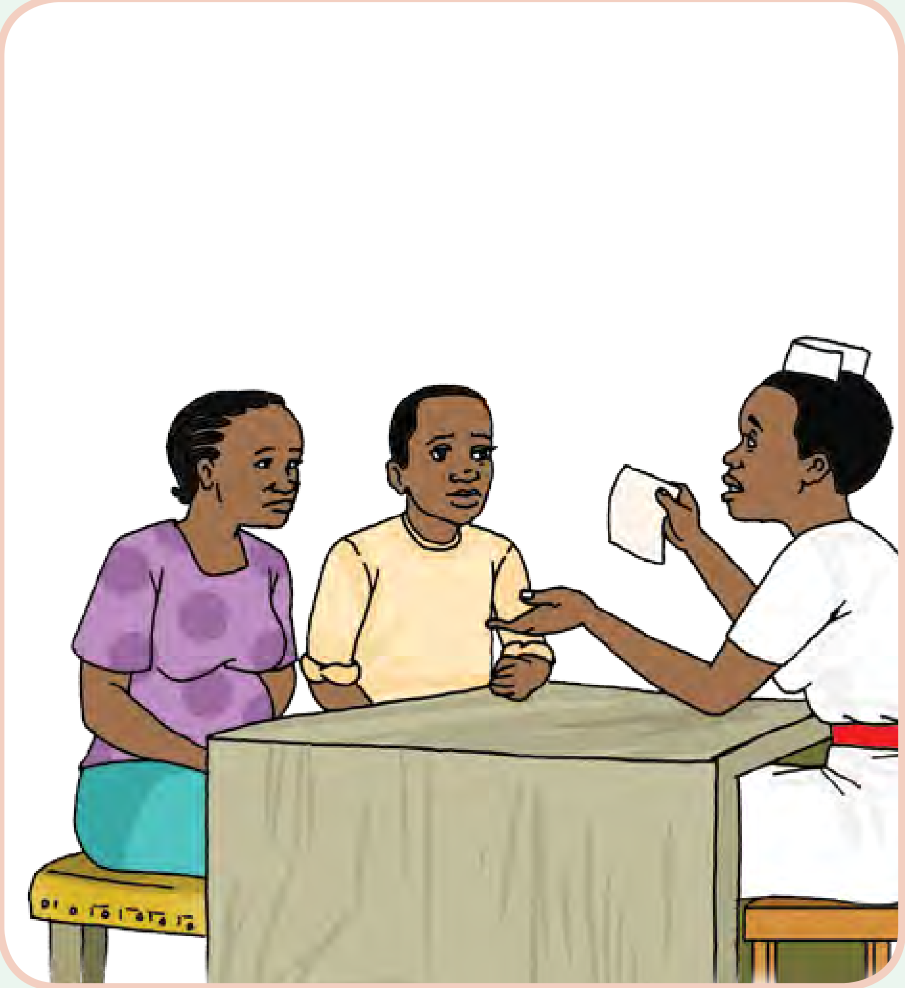
Next Session

I know something about HIV, I can do something about it, and I can do something for someone else affected by HIV and AIDS!

Facilitator's Guide

Engaging with Care

Objectives:

- Learn what it means to be an active and organised patient.
- Discuss the challenges of adherence to medication and strategies to stay adherent.
- Develop strategies for how Kanyakla members can help one another with adherence to medication.

Session overview (130 minutes)		
**Activity**	**Time (minutes)**	**Objectives**
Prayer and mindfulness	20	• Gather the group together and pray for a good session.<br>• Have the group reflect on the meditations below.
Wheel of Hope	15	• Review the material from the previous session.<br>• Use the questions and key messages to guide the review.
Class - Teach from the flipbook	50	• Teach about how ARV's work.<br>• Use role play to illustrate the importance of being organised and engaged patients.
5 Questions activity	30	• Demonstrate how to be an active, engaged patient.
Wisdom circle	15	• Review the objectives of the session. What was learnt?<br>• Schedule the next session.<br>• Review the handout.

Meditations:

"A man too busy to take care of his health is like a mechanic too busy to take care of his tools." – ***Spanish Proverb***

Session Four: Engaging with Care

1 2 3 **4** 5 6 7 8

Objectives:

- Learn what it means to be an active and organised patient.
- Discuss challenges to adherence and strategies to stay adherent.
- Develop strategies for how Kanyakla members can help one another with adherence.

I know something about HIV, I can do something about it, and I can do something for someone else affected by HIV and AIDS!

5 Questions

There are 5 questions that everyone should ask their doctor about their medications.

It is important to understand these things about your medications so you know why you are taking them and you know how to take them properly.

It is a good idea to ask your doctor questions if you are confused about anything or don't understand part of your treatment or care.

Take time to discuss and practice these questions with the group.

1. What is my **DIAGNOSIS**? (What is causing me to be sick?)
2. What is the **NAME** of the medication prescribed? (Try to write it down so you remember)
3. How does the medicine **HELP** me?
4. **HOW MUCH** should I take and **HOW OFTEN** should I take it? (Dosing instructions)
5. Are there any **SPECIAL INSTRUCTIONS** for taking the medication? (For example: Should I take it with food, as soon as I wake up, or before I sleep?)

5 Questions

1. What is my DIAGNOSIS (What is causing me to be sick?)
2. What is the NAME of the medication prescribed?
3. How does the medicine HELP me?
4. HOW MUCH should I take and HOW OFTEN should I take it (Dosing instructions)?
5. Are there any SPECIAL INSTRUCTIONS for taking the medication?

What Steps Can I Take to Promote My Own Health?

Ask the group what steps they can take to promote their health in the settings shown in the boxes. Below are some possible answers. Use these to prompt discussion if the group is unsure how to answer.

Seeking Healthcare

- If a health problem arises that cannot be dealt with at home, I can go to the hospital.
- If I am not sure whether my sickness requires medical attention, I can ask a friend or my community health worker for advice.
- I can go to all medical appointments on the scheduled date.

Things I can do when at the hospital

- I can be honest about my symptoms with the health workers at the clinic.
- I can ask questions if there is something I do not understand.
- I can make sure I know the name, purpose, and dose of all medications I am taking.

Social Support

- When I have a problem, I can share it with my family, friends and/or Kanyakla members.
- I should not feel the need to hide medications and can be open about my HIV status.

Prevention

- I can take steps to prevent illnesses before they occur by using the following precautions: mosquito net, clean water, clean latrine, safe sex, family planning.
- I can do things that promote health for myself (exercise, good nutrition, adequate sleep, socialise with friends and family, attend to spiritual needs).

What Steps Can I Take to Promote My Own Health?

Seeking Healthcare

At the Hospital/Clinic

Social Support

Prevention

As a Kanyakla, What Can We Do to Promote the Health of Others?

Brainstorm what kind of behaviours empowered Kanyakla members would demonstrate.

(Note: It is also a good way to set up a contract for Kanyakla empowerment with the group.)

Below are some of the behaviors an empowered Kanyakla would have:

- Get the group to name 3 ways HIV can be transmitted.
- Write the answers on the left of each of the blue boxes.
- Next, ask about ways to prevent infection or stay healthy from each of these possible means of transmission.
- Write the answers on the right side of the blue boxes.

Maintaining confidentiality

- We can maintain confidentiality for one another. What is discussed in Kanyakla stays in Kanyakla.

Support

- We can visit one another and, in the process, identify any problems that arise.
- If a member has a problem or becomes sick, we can support him or her.
- We can be an advocate for one another in addressing health or other problems.

Empowerment

- We can encourage others in our Kanyakla and our community to take charge of their own health.

Community

- We can share what we have learnt in Kanyakla with others in our community.
- We can be open about our own health challenges to reduce stigma in the community.

As a Kanyakla, What Can We Do to Promote the Health of Others?

Maintaining Confidentiality

Support

Empowerment

Community

Facilitator's Guide

Let's Role Play!

Situation one:

Three fishermen go out fishing for a few days. Two of them are HIV+, the third is not.

Fisherman 1 is HIV+. He puts his medications in his pocket. He is embarrassed and stigmatised and does not share his status or inform anyone he is on medications.

Fisherman 2 is also HIV + but has his medications in a plastic bag. He shows them to the other fishermen.

Fisherman 2: "These are my medications. Please help me look after them and remind me to take them if I forget."

As they are out on the lake a wave comes into the boat. The medications get washed out of the first fisherman's pocket. The plastic bag full of medications for the other fisherman is saved by the third because he knows they are important.

Fisherman 1: *"Oh no!"*

Fisherman 2 and 3: *"What's wrong?"*

Fisherman 1 now cannot explain that he lost his medication.

Situation two:

Woman 1 is walking home and sees woman 2 picking up pills off the ground.

Woman 1: *"What are you doing?"*

Woman 2: *"My medications fell out of my purse and now I'm trying to pick them up. But I'm not sure which is which and if these are even all of them!"*

The first woman helps her pick her medications up.

Woman 1: *"Come with me. I will show you how I keep my medication."*

They go to her house and the first woman shows the second how she keeps her medications in a special, safe spot. She has them labelled and has told others where to find them.

Let's Role Play!

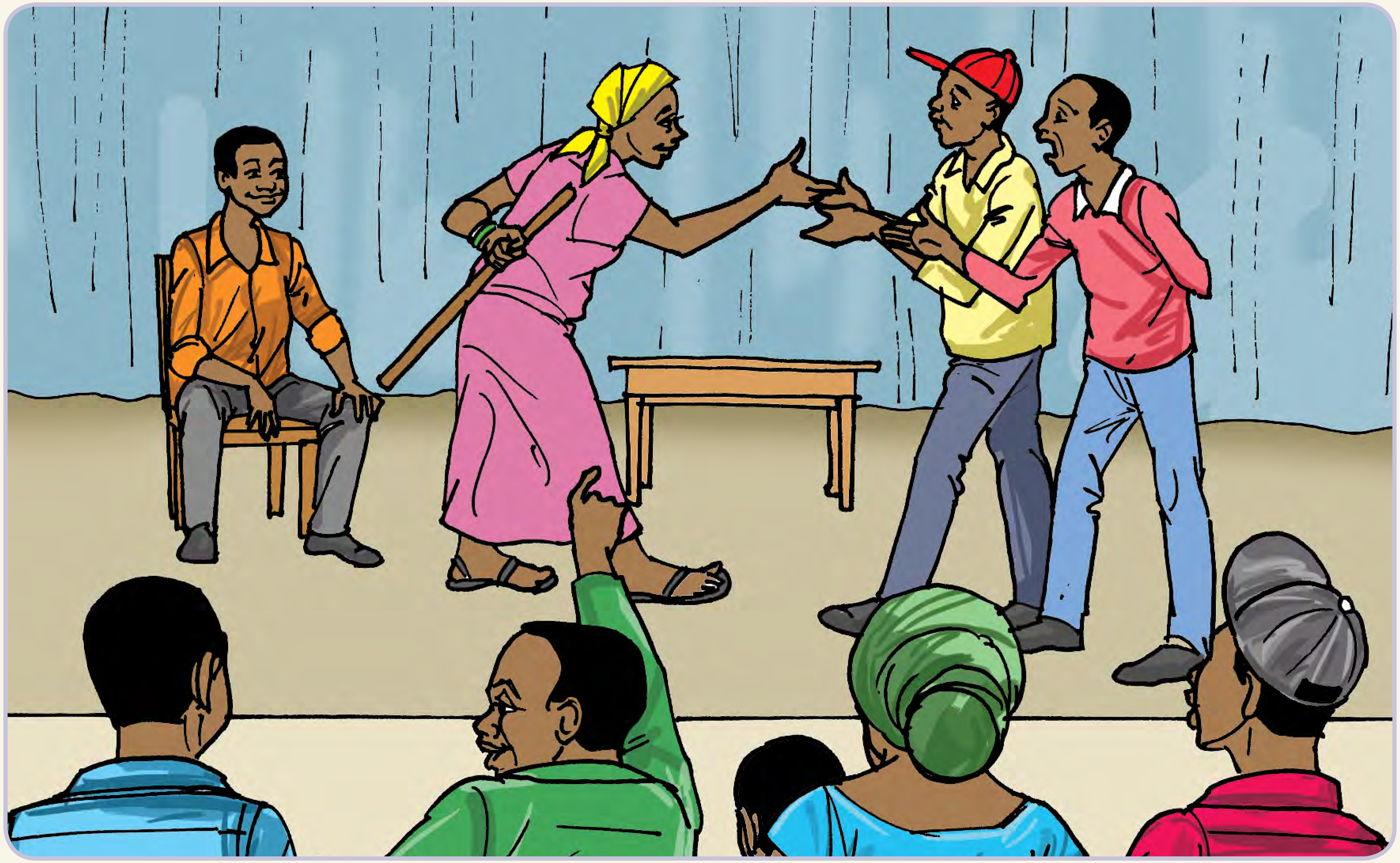

Facilitator's Guide

Organising Medications

Using the questions below, guide a discussion on the importance of organising medications so you can take the right ones at the right time, bringing medications to appointments, and keeping them in a safe place where you can find them but that is away from children.

?

Questions for the group:

1. How do you organise your medications?
2. Have you ever lost medicines?
3. What are good ways to organise medicines?
4. Would other people be able to find your medications if you are too sick to get them?

Organising Medications

?

1. How do you organise your medications?
2. Have you ever lost your medications?
3. What are some good ways to organise medicines?

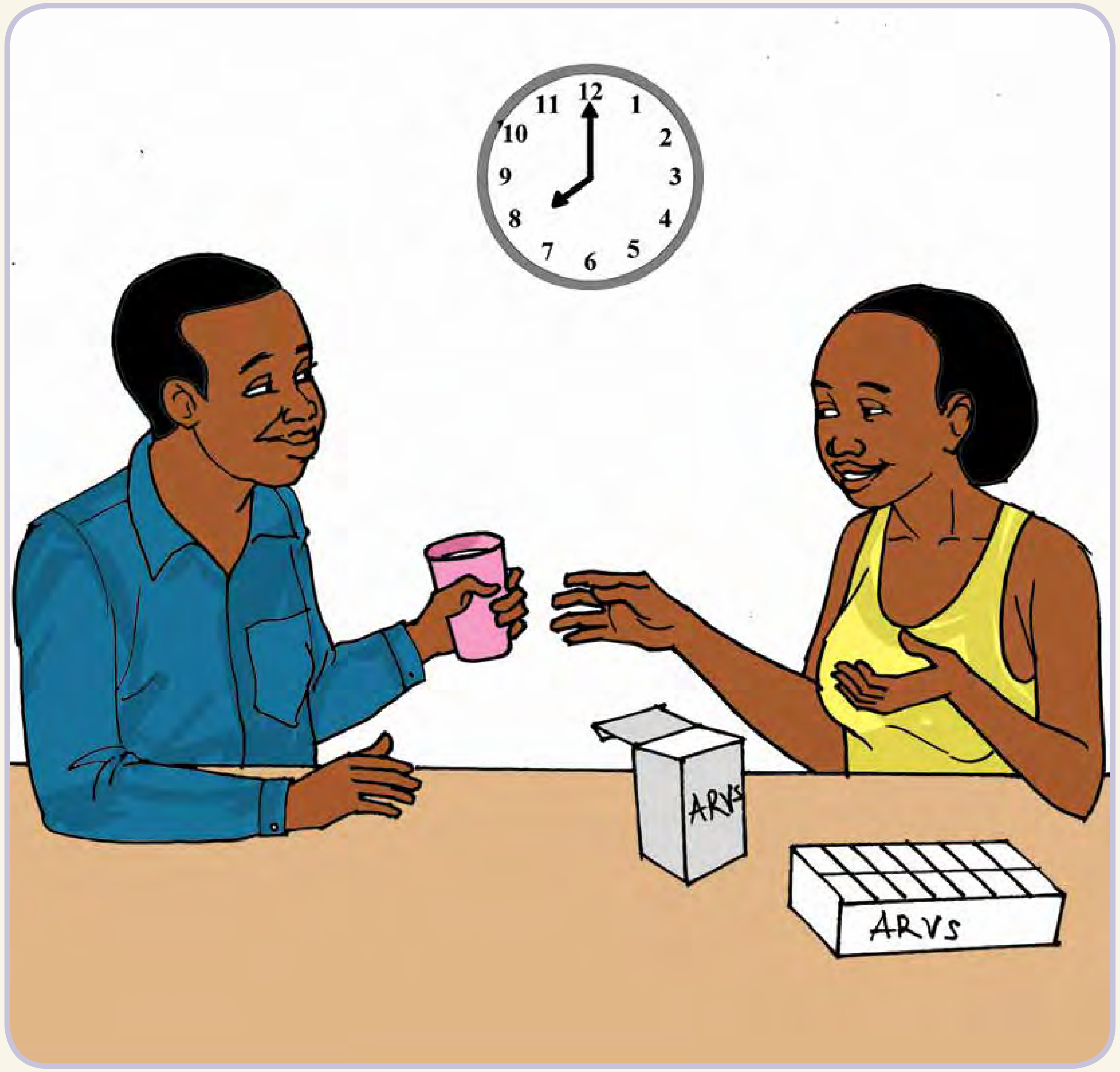

Facilitator's Guide

The Way Forward

Key message:

- Take an active role in your care: Ask questions, seek care when you need it, get support, and prevent health problems through healthy living.
- As a Kanyakla, we can help keep each other and our community healthy.
- Be able to answer the '5 Questions' about your medications.
- Keep medications organised and in a safe place.

Challenge: Go home and organise your medications or help a family member or friend organise their medications.

Next Session: The next session is HIV medications and you. It also covers nutrition.

Note: Remember to communicate the time and place of the next session.

Remember:

I know something about HIV, I can do something about it, and I can do something for someone else affected by HIV and AIDS!

The Way Forward

Key message:

- Take an active role in your care: Ask questions, seek care when you need it, get support, and prevent health problems through healthy living.
- As a Kanyakla, we can help keep each other and our community healthy.
- Be able to answer the '5 Questions' about your medications.
- Keep medications organised and in a safe place.

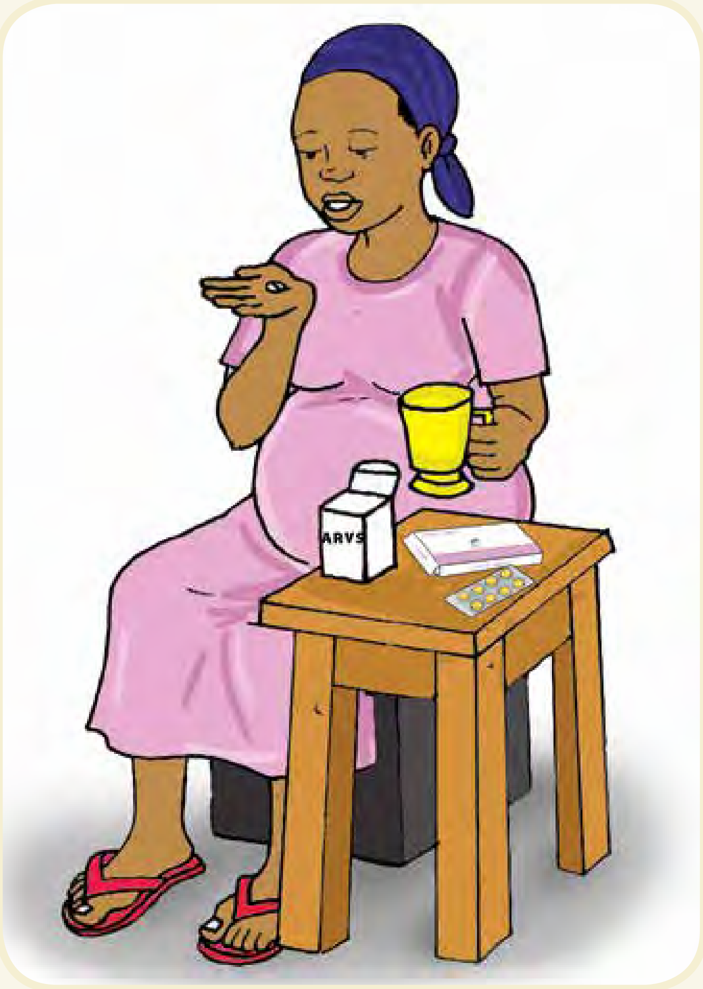

Challenge

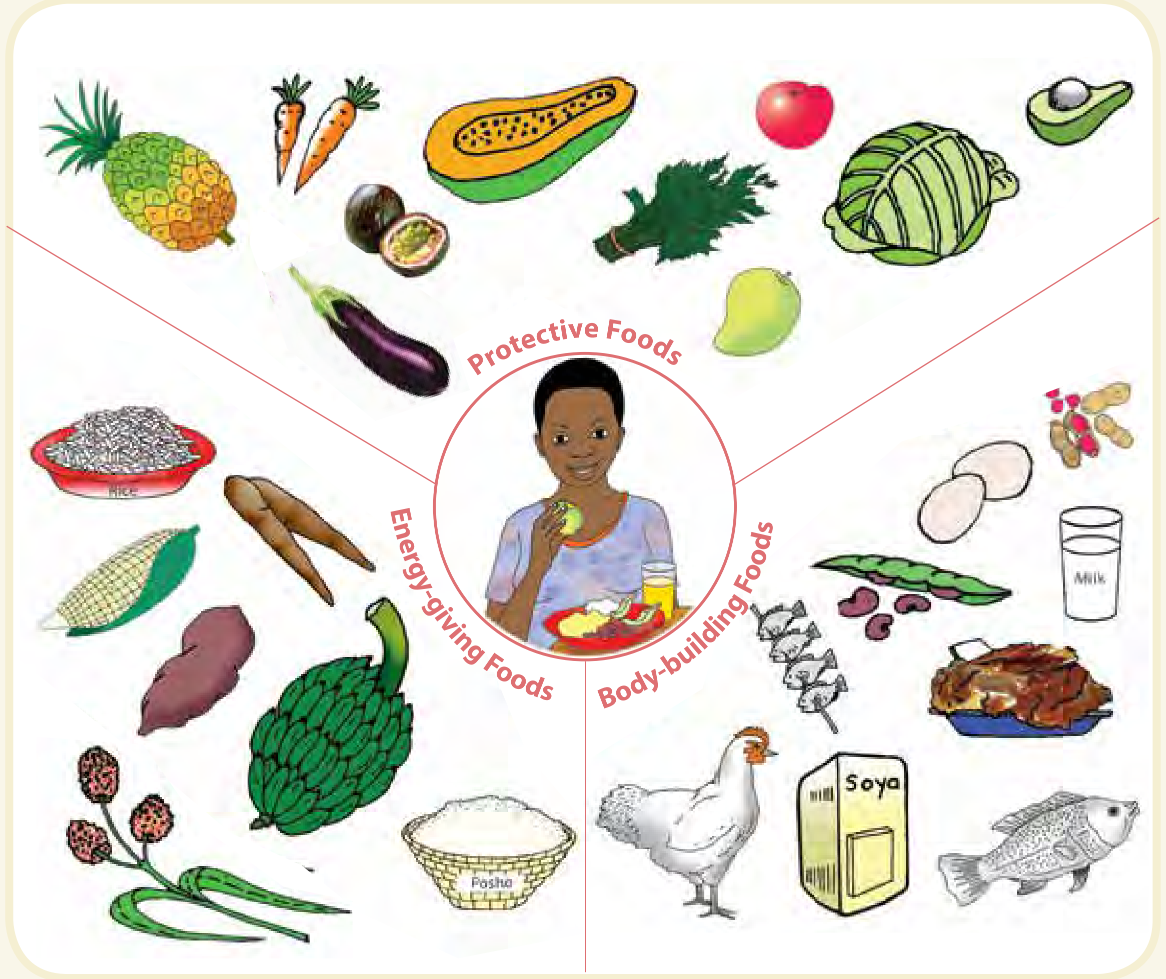

Next Session

I know something about HIV, I can do something about it, and I can do something for someone else affected by HIV and AIDS!

Facilitator's Guide

ARVs and You

Objectives:

- Discuss local beliefs around HIV
- Understand the appropriate use of herbs and medications
- Discuss nutrition and balanced diet for a healthy life

Session overview (140 minutes)

Activity	Time (minutes)	Objectives
Prayer and meditation	20	• Gather the group together and pray for a good session.<br>• Have the group reflect on the meditations below.
Wheel of Hope	15	• Review the material from the previous session.<br>• Use the questions and key messages to guide the review.
Class - Teach from the flipbook	60	• Discuss local beliefs about HIV and the use of herbs and medication.<br>• Discuss nutrition.
Role play	30	• Demonstrate the importance of taking ARVs and visiting herbalists.
Wisdom circle	15	• Review the objectives of the session. What was learnt?<br>• Schedule the next session.<br>• Review the handout.

Meditations:

"Your body is a temple, but only if you treat it as one." – *Astrid Alauda*

"Our bodies are our gardens – our wills are our gardeners." – *William Shakespeare*

Session Five: ARVs and You

1 2 3 4 **5** 6 7 8

Objectives:

- Discuss local beliefs around HIV
- Understand the appropriate use of herbs and medications
- Discuss nutrition and balanced diet for a healthy life

I know something about HIV, I can do something about it, and I can do something for someone else affected by HIV and AIDS!

Let’s Role Play!

“Chira”

A woman of unknown HIV status has a child. She begins feeling sick, loses weight, is very tired and has a cough.

At first she is told that this is “chira”, and that the family is suffering from a curse for having broken a taboo.

The woman seeks out an herbalist, and is given herbs and instructions for breaking the “chira”. The child continues to get worse.

A Kanyakla member meets the woman.

Kanyakla member: *“How are you doing?”*

Woman: *“My child is not doing well.”*

Kanyakla member: *“You should go to the health facility to get him tested.”*

The woman and her child go to the clinic, and the child is found to be HIV/TB+. The mother is also tested and is positive. They are put on ARVs and medications and the child begins to get healthier.

Let's Role Play!

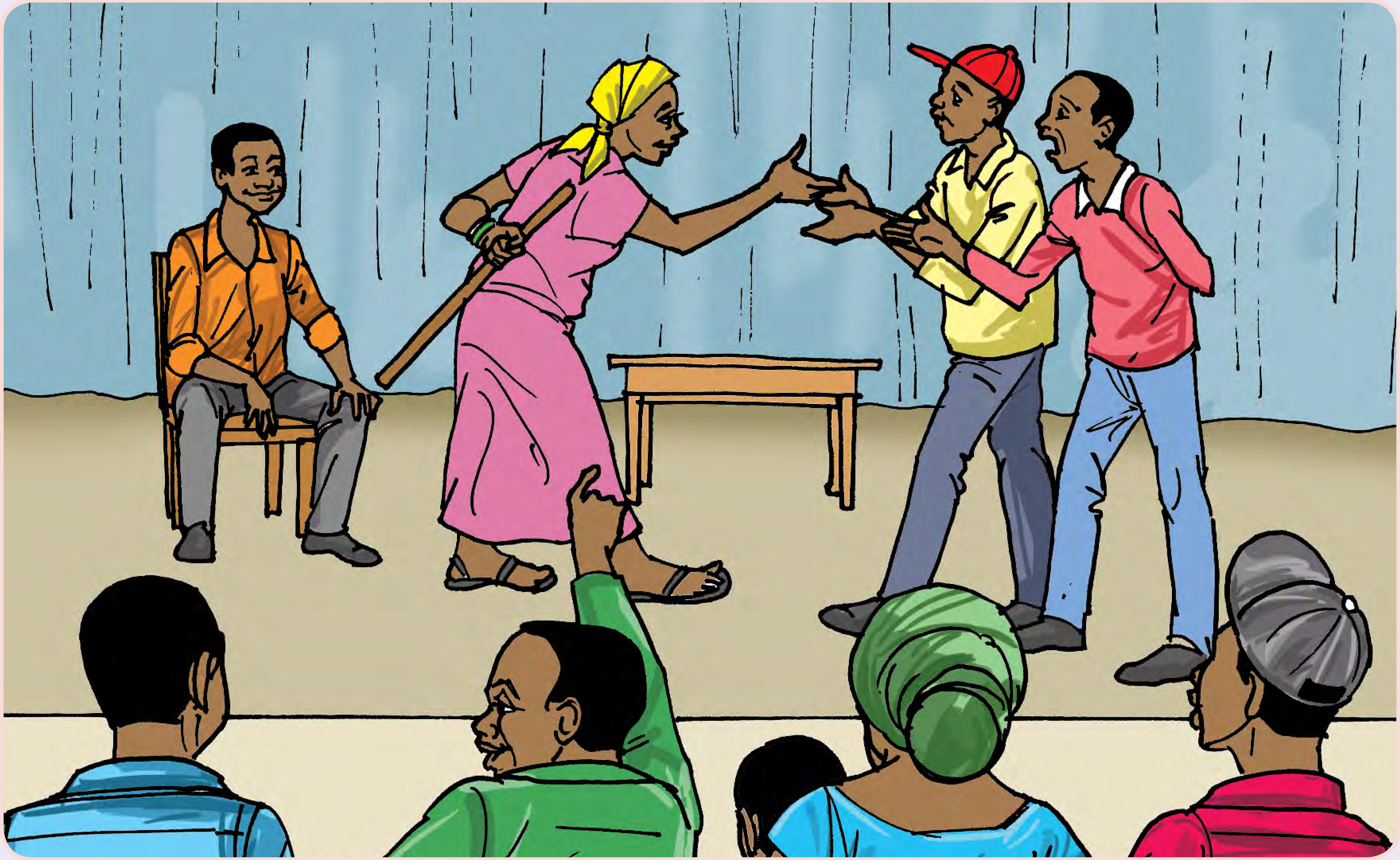

Facilitator's Guide

Local Beliefs Around HIV vs "Chira"

Ask the group the question below. Allow them time to answer before giving them the recommendations.

- If you are sick, when should you go to the hospital and when should you visit an herbalist?

We recommend that if you are sick, you should first go to the hospital. If you have not been tested for HIV recently you should get tested.

Go see an herbalist if the hospital does not have treatment that helps or if you are still having symptoms after you have started medicines given to you by the hospital.

Chira vs HIV:

Ask the group:

What is the difference between Chira and HIV?

If someone tells you that you have Chira, what should you do?

Key message:

Encourage people to go to a hospital or clinic and be tested for HIV/TB or other illnesses before going to an herbalist or blaming ill health on Chira.

Local Beliefs Around HIV vs "Chira"

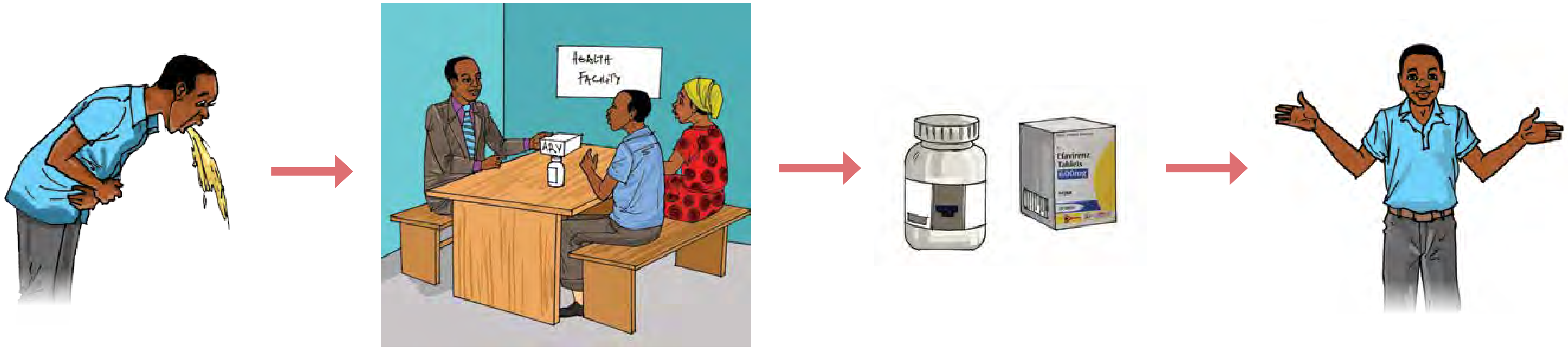

vs

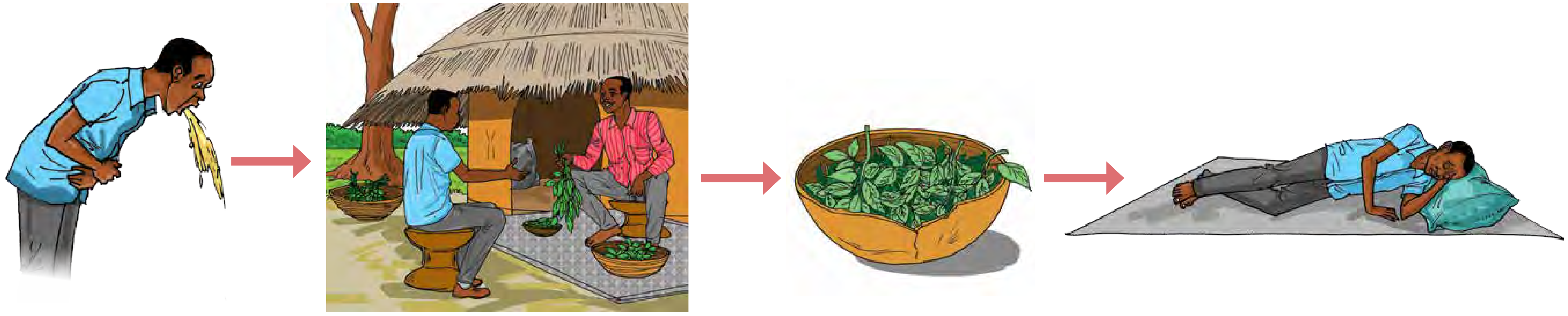

Facilitator's Guide

Herbs and Medications

Use the questions below to lead a discussion about the use of doctor-prescribed medicines and herbs from herbalists.

?

1. Can HIV medications and herbs be taken together?
2. What have you heard from other people?
3. What have you heard from doctors?
4. What have you heard from herbalists?

Key message:

- Do not stop taking HIV medicines to take herbal medications. It is better to take both than to stop HIV medications to take herbs instead.
- Be honest with your healthcare provider about the herbs you may be taking.
- If you have side effects from a drug, go to the health clinic first. It could be an infection and not a side effect. Also, they may be able to change the drug you are taking.
- If there is no solution at the health facility then you can go to an herbalist to see if they have anything to help the side effects.
- Be honest with doctors about the side effects of the medicines. They may be able to change the medication or tell you how long the effects will last.
- Prayer is also helpful. But no one should make you choose between prayer and HIV medications. It is ok to do both at the same time.

Herbs and Medications

?

1. Can these be taken together?
2. What have you heard others say?
3. What do doctors say?
4. What do herbalists say?

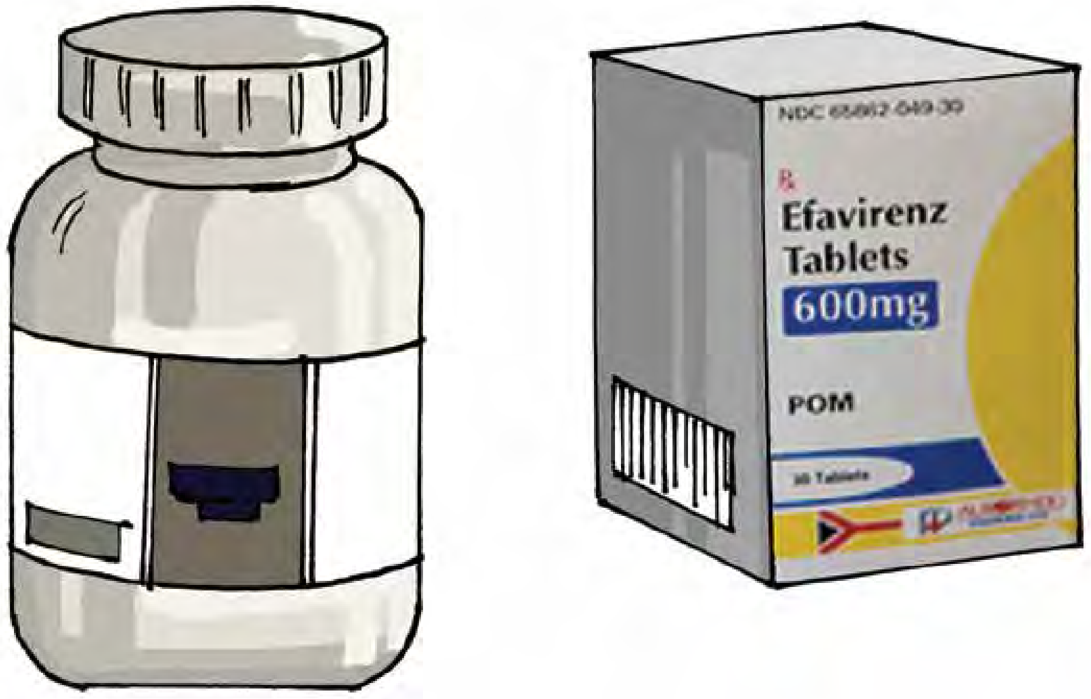

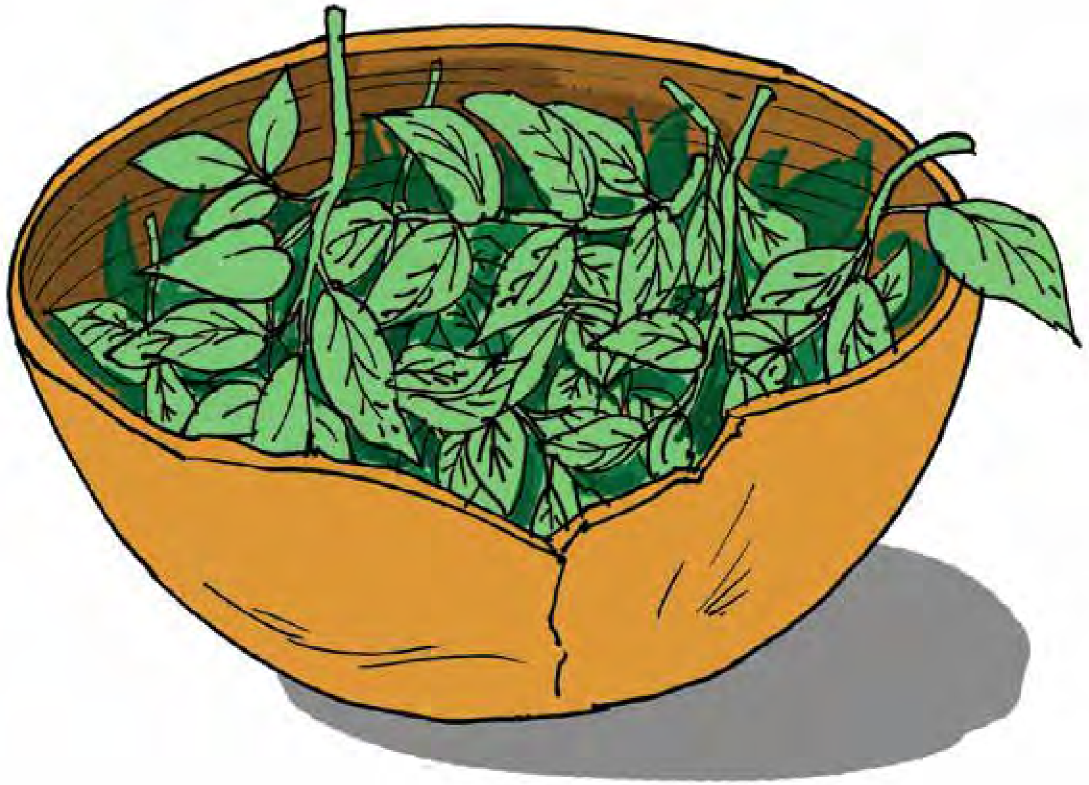

Let's Role Play!

HIV clinic

Patient is prescribed ARVs and the doctor instructs her on how to take them.

Doctor: *"Do you ever use any herbs?"* (He speaks in a tone that shows he does not approve the use of herbs.)

(Patient starts to say yes then decides to say no.)

Patient: *"Ye- No, I don't."*

Doctor: *"Well, don't take any herbs with the ARVs." The doctor instructed her not to take any herbs with the ARVs.*

Patient: *"Why?" The patient asks the doctor why?*

Doctor: *"We don't know how the herbs work and there might be interactions that cause problems."*

The patient goes home and asks the grandmother who is an herbalist whether she should stop taking her ARVs or the herbs she has been using for improving fertility.

Patient: *"Should I stop taking the ARVs or the herbs for fertility? The doctor says I cannot take them together."*

Grandma: *"If you can't take both, you have to choose. I can't make up your mind for you."*

2 people with HIV

Man 1 visits his friend, Man 2, and notices that his friend keeps on dashing out after every few minutes.

Man 1: *"Is there a problem? You keep moving out."*

Man 2: *"I have had persistent diarrhoea for more than 3 months now and, despite seeking treatment, there is no improvement."*

Man 1: *"Have you tried using some local herbs?"*

Man 2: *"No, because I have been using some drugs from the hospital and the clinician told me not to take both ARVs and herbs."*

Man 1: *"I'm on ARVs. I had the same problem but I still used the herbs and it helped me a lot."*

Man 2: *"Aren't you worried about interactions between herbs and ARVs?"*

Man 1: *"I was really suffering with the diarrhoea and was almost considering stopping the ARVs that he thought were causing it. I went to an herbalist who said she had herbs to help diarrhoea, but the herbalist was also in the Kanyakla and told me that I should not stop the ARVs no matter what. She told me to tell my doctor about the diarrhoea and the herbs and to be open. Since then I have been using both and I'm free with my doctor and the herbalist. The doctor was even impressed that the herbs actually work for diarrhoea and my CD4 is improving. I can take you to see the same herbalist."*

Let's Role Play!

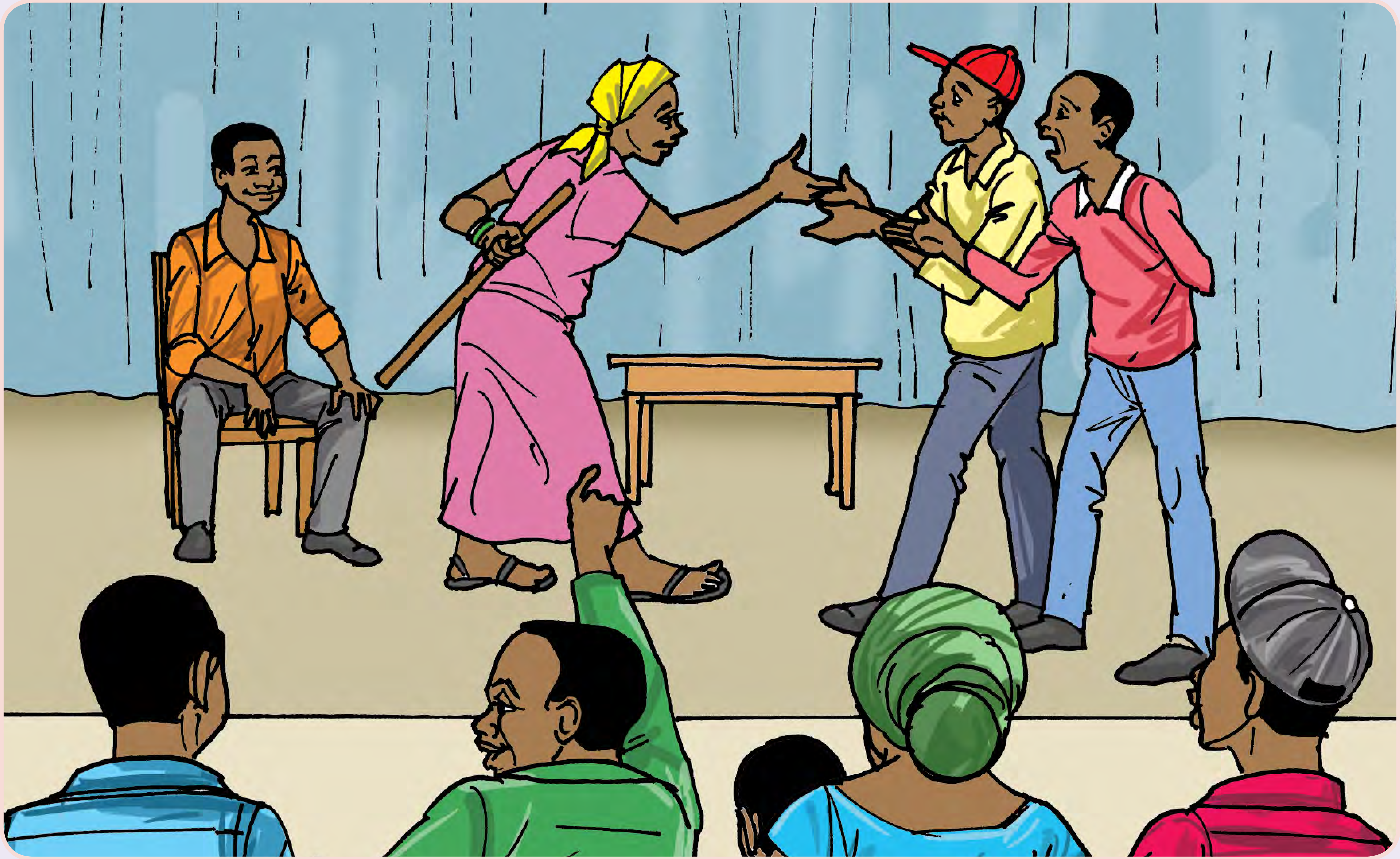

Facilitator's Guide

Nutrition for People Living with HIV/AIDS

Hold a group discussion about what good nutrition is, focusing on the foods available locally (and cheaply) that are healthy and make a complete diet. Also, focus on the 3 'food groups': carbohydrates (energy-giving foods), vitamins (protective foods), and protein (body-building foods). Complete nutrition involves foods from all the different groups.

Carbohydrates

These are the starches like ugali and chapatti. Whole grains are better for you. Brown ugali is better than white.

Vitamins

You can get vitamins in vegetables and fruits. Eat local fruits and vegetables like papaya, mango, tomatoes, and kale.

Protein

There are lots of local options here as well. You can eat fish, omena, eggs, groundnuts, chicken, and meat.

Nutrition for People Living with HIV/AIDS

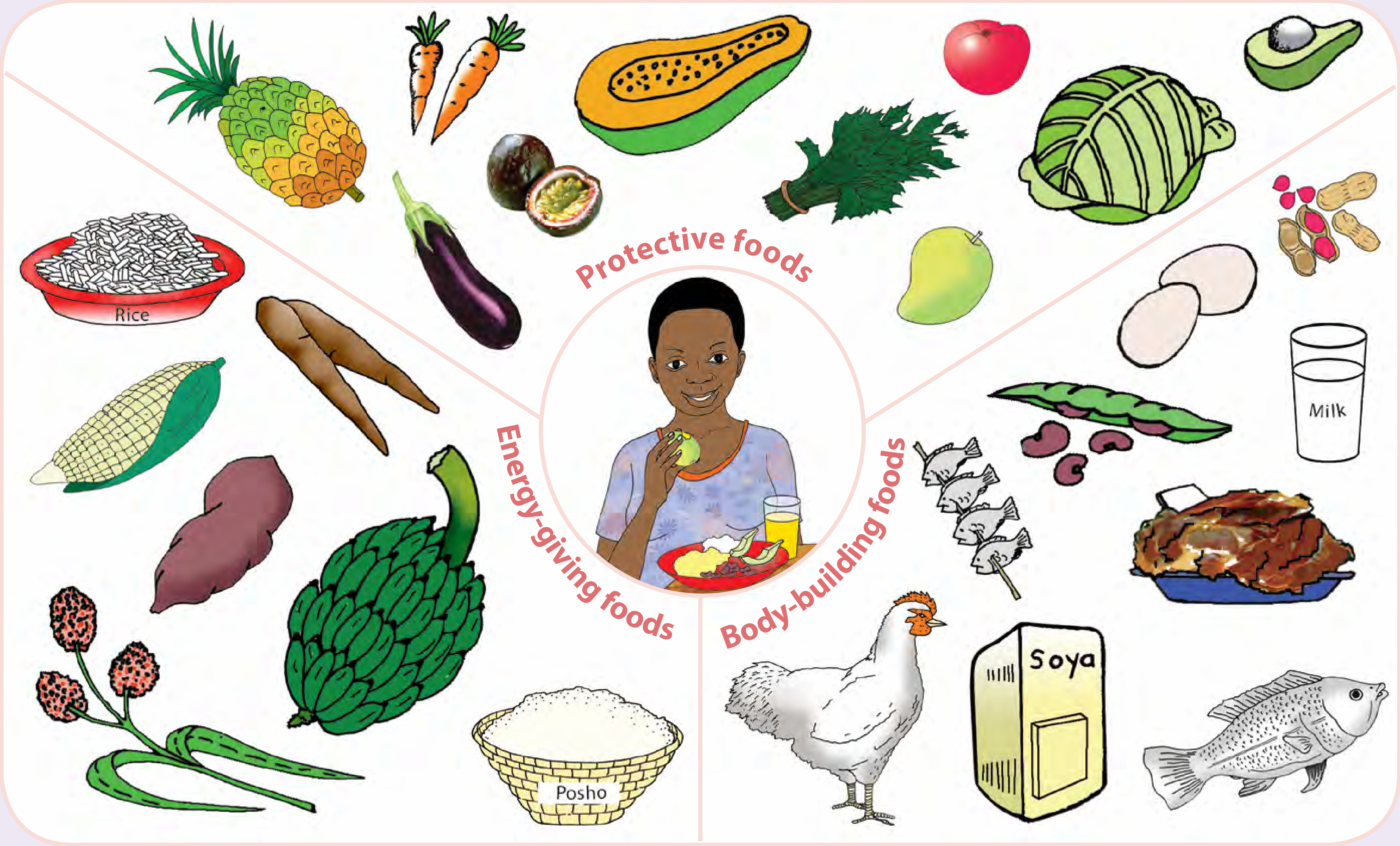

Facilitator's Guide

The Way Forward

Key message:

- If you have a health problem, go to the health centre before using any other treatment
- Don't stop taking ARVs because you are using herbs
- Be honest with your doctor about the herbal medicines you may be taking
- Good nutrition means eating from all the food groups: protein, carbohydrates, and vitamins

Challenge: Cook a healthy meal using all 3 of the food groups. Discuss with your partners what herbs they are using or what they have used.

Next Session: The next session is "Chasing Stigma". We will discuss how we can help reduce stigma in our community.

Note: Remember to tell members the time and place of the next session.

Remember:

I know something about HIV, I can do something about it, and I can do something for someone else affected by HIV and AIDS!

The Way Forward

Key message:

- If you have a health problem, go to the health centre before using any other treatment
- Don't stop taking ARVs because you are using herbs
- Be honest with your doctor about the herbal medicines you may be taking
- Good nutrition means eating from all the food groups: protein, carbohydrates, and vitamins

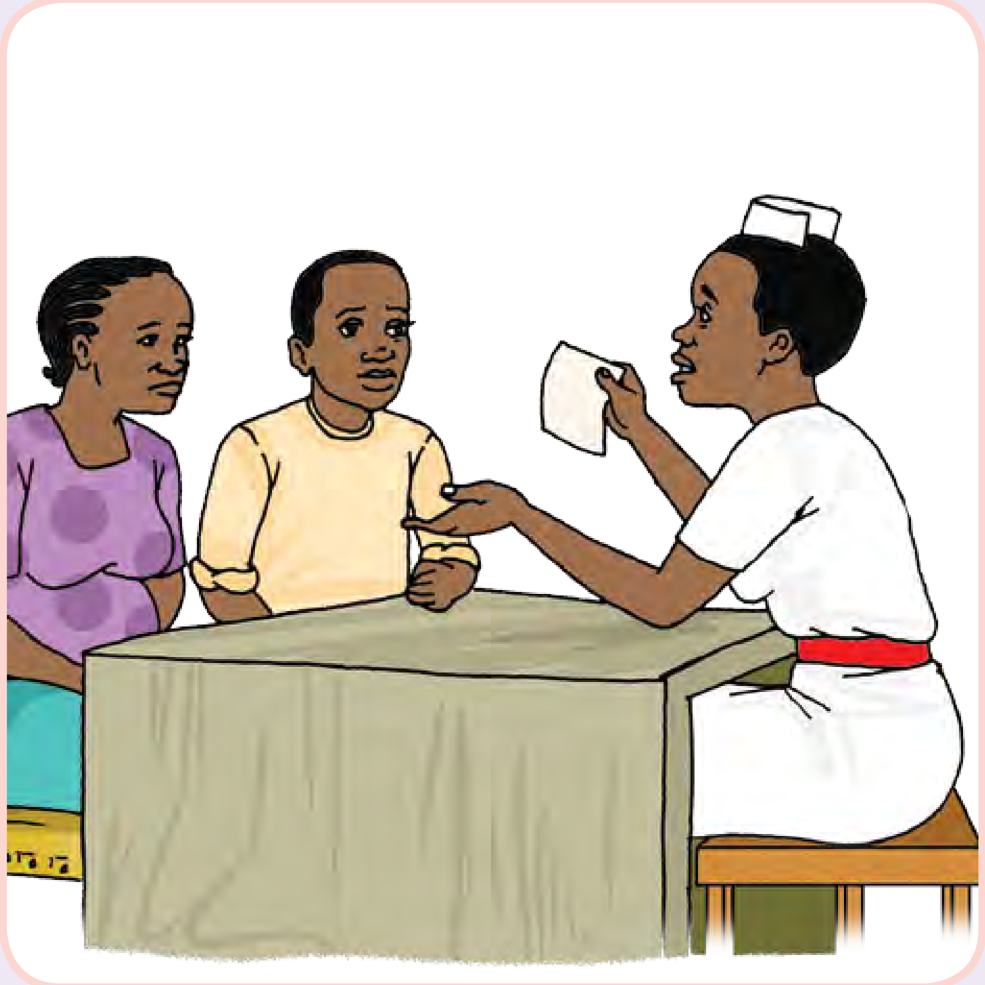

Challenge

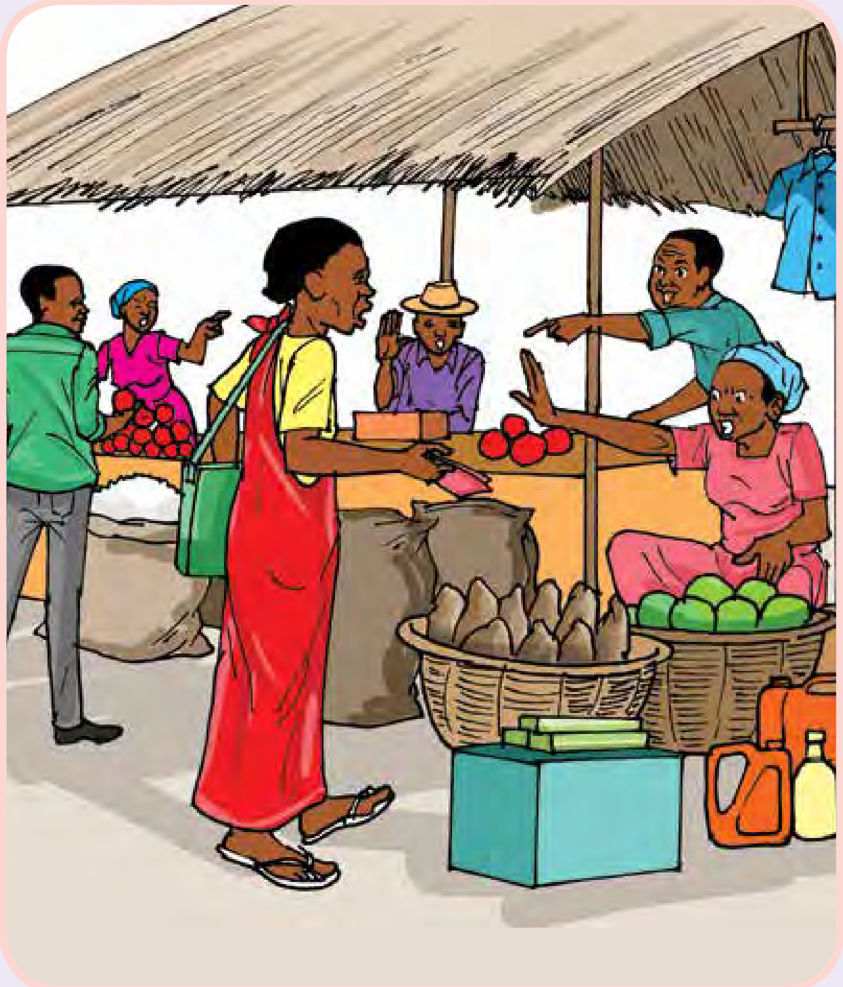

Next Session

I know something about HIV, I can do something about it, and I can do something for someone else affected by HIV and AIDS!

Facilitator's Guide

Chasing Stigma

Objectives:

- Discuss talking to kids about sex.
- Discover how stigma is started and what we can do to fight stigma.
- Discuss the 5 pillars of Kanyakla support.

Session overview (120 minutes)		
**Activity**	**Time (minutes)**	**Objectives**
Prayer and mindfulness	20	• Gather the group together and pray for a good session.<br>• Have the group reflect on the meditations below.
Wheel of Hope	15	• Review the material from the previous session.<br>• Use the questions and key messages to guide the review.
Class - Teach from the flipbook	55	• Discuss talking to kids about sex.<br>• Discuss the 5 pillars of Kanyakla support.
Role play	15	• Demonstrate the effects of stigma.
Wisdom circle	15	• Review the objectives of the session. What was learnt?<br>• Schedule the next session.<br>• Review the handout.

Meditations:

"You are not your illness. You have an individual story to tell. You have a name, a history, a personality. Staying yourself is part of the battle." – *Julian Seifter*

"Let food be thy medicine and medicine be thy food" – ***Hippocrates***

Session Six: Chasing Stigma

1 2 3 4 5 **6** 7 8

Objectives:

- Discuss talking to kids about sex.
- Discover how stigma is started and what we can do to fight stigma.
- Discuss the 5 pillars of Kanyakla support.

I know something about HIV, I can do something about it, and I can do something for someone else affected by HIV and AIDS.

Facilitator's Guide

Let's Role Play!

Talking to kids about sex:

Scenario One

Neighbour: You meet the aunt at the market. You tell the aunt that you have heard that her niece has been sexually active with a boy at school. You ask if it is true and what the aunt and her family will do about it.

Aunt: You are at the market when you learn from a neighbour that your niece has been sexually active. You rush home to confront your niece. You tell her that you are very angry about her choices and demand more information about her sexual activity. When she refuses you are visibly upset.

Niece: At home, you are confronted by your aunt. You are upset that she would talk to you about your sex life because you think it isn't any of her business. She is so angry at you that you refuse to answer her questions and get angry yourself.

Scenario Two

Mother: You talk with your husband (the father) and decide that now is a good time for him to talk to your son about sex. You want to make sure that the father doesn't go into too much detail, that he stresses the risks involved in having sex, and that the son should not be having sex until he is much older.

Father: You speak with your wife and decide that the time is right to talk to your son about sex. He is 10 years old, so you want to give him basic information about what sex is, what it is used for, and that it is a responsibility that should not be taken until he is ready. You also stress that your son can ask you questions any time and that you want to be a resource. You want him to get the facts from him and not learn wrong details from someone else.

Son: You are 10 years old. At first, you are hesitant about talking with your father about sex. You are embarrassed and feel ashamed. However, as the talk goes on, you warm up and ask a few questions. You ask where babies come from and what happens to sperms when they are deposited in the vagina. At the end, you agree to be responsible and ask if you have more questions

Let's Role Play!

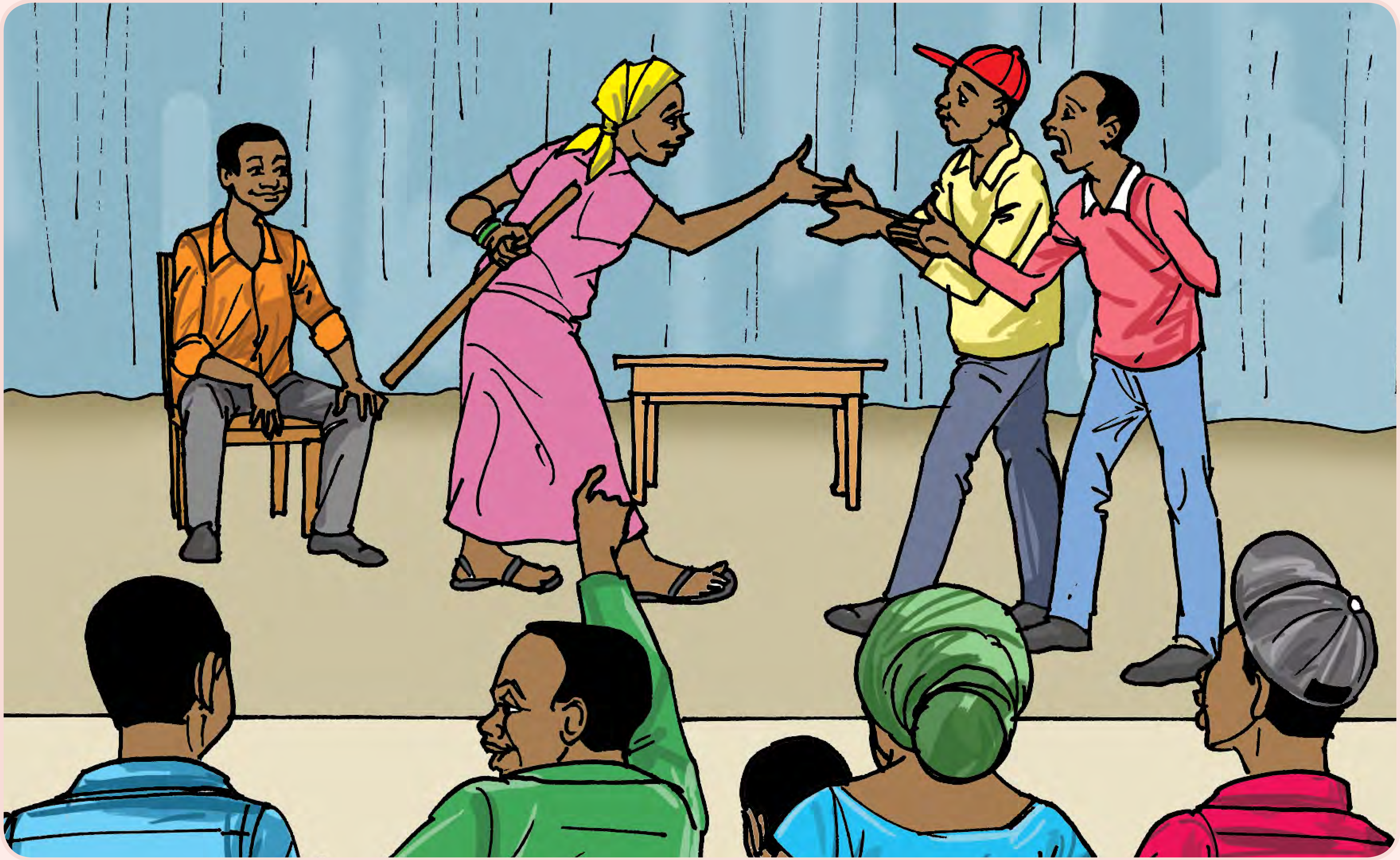

Facilitator's Guide

Talking to Kids About Sex

Use the following questions to lead a discussion about the importance of talking to kids about sex and challenges that may come up when trying to start talking about sex.

1. What are the challenges of talking to kids about sex?
2. Why is it important to talk to kids about sex?
3. What age should your child be before you start talking to them about sex and HIV/AIDS?
4. Whose responsibility is it to talk to children about sex?

Suggested responses

You can point out some of these responses if they are not covered by the group.

1. Challenges when talking to kids about sex:
    - Myths and misconceptions, culture, taboos and norms, the effects of peer pressure and its influence on sexual knowledge
2. Important reasons to talk to kids about sex:
    - Knowledge of sexual/reproductive health, sexual abuse and exploitation, STIs, family planning, and pregnancy prevention

Talking to Kids About Sex

?

1. What are the challenges of talking to kids about sex?
2. Why is it important to talk to kids about sex?
3. What age should your child be before you start talking to them about sex and HIV/AIDS?
4. Whose responsibility is it to talk to children about sex?

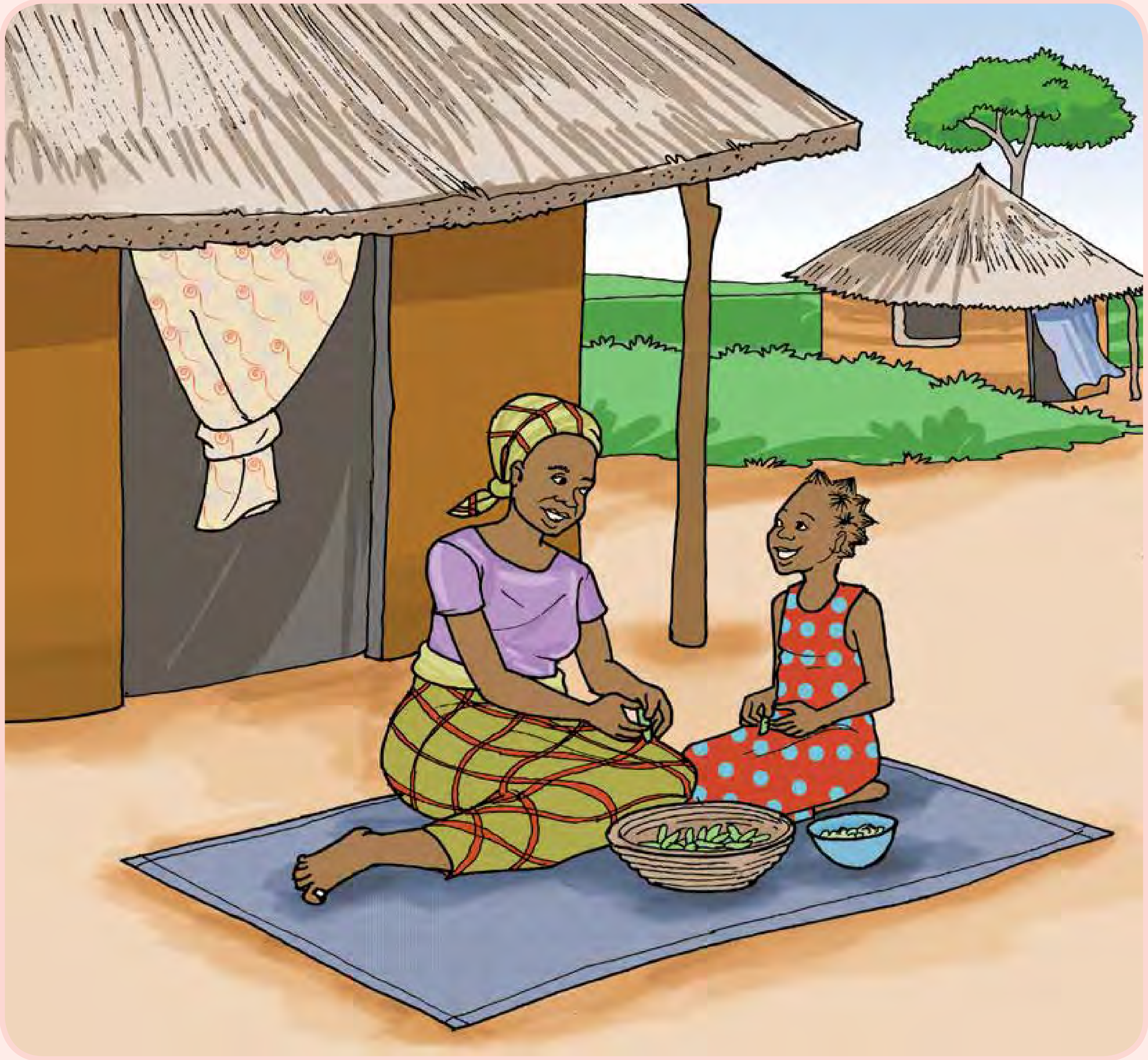

Facilitator's Guide

Let's Role Play!

Stigma

Three patients are waiting to see the doctor and they begin talking about why they are there. Later another patient joins them.

Patient 1: *"I have a bad headache and need to get some medicine. Why are you at the clinic?" (Patient 1 asks Patient 2 and Patient 3, but is really wondering if anyone has HIV.*

Patient 2: *"I have an eye infection."*

Patient 3: *"I have a cough."*

Patient 4 arrives and the others ask why he is there.

Patient 4: *"I have HIV and I've come for a check-up. Is that why you are all here?"*

Patient 3: *"Well actually, I also have HIV and that's really why I'm here.*

Patient 2: *"That's also why I am here."*

Patient 1: *"No, I'm here to just get treatment for my headache." Patient 1 moves away from the others.*

The other patients (2, 3, and 4) discuss when they found out about their status, that they have been healthy as long as they take their ARVs, and how challenging it can be to hide HIV status.

Patient 3: *"I didn't want to say why I was really here. I feel as though I may be thought of as an outcast to the rest of the community if I am open about my status."*

Patient 4: *"You should be open if you have HIV. It is better to educate family members and friends rather than trying to hide the disease.*

Patient 3 and 4 discuss how they feel much better about their health.

Let's Role Play!

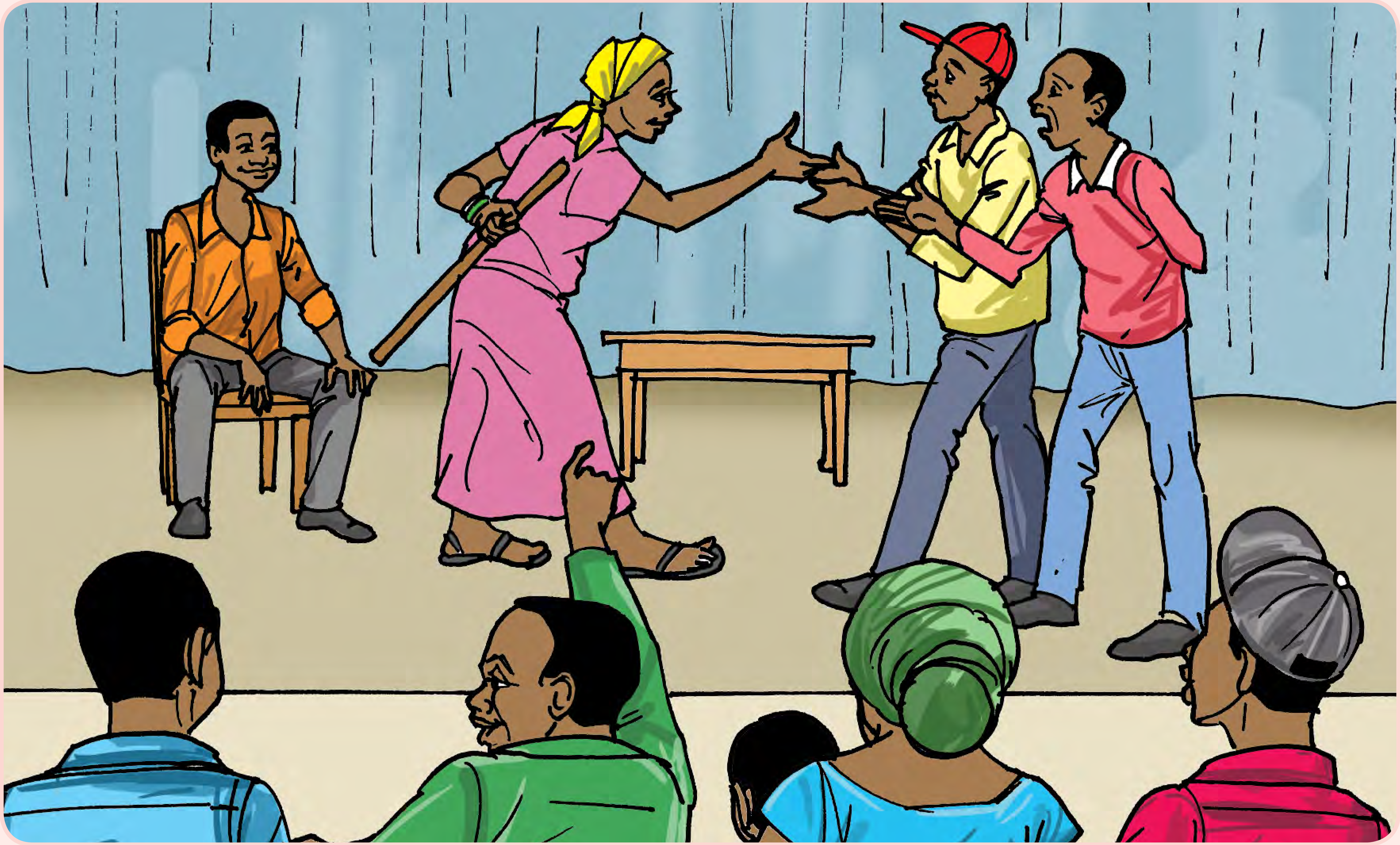

Facilitator's Guide

Stigma

Use the questions below to guide a discussion on how stigma is experienced, and the effects it has on health and sense of community.

There are no right answers. The goal is for the group to discuss how stigma has impacted them and how HIV disclosure may help.

?

Discussion questions for the group:

1. What is stigma?
2. In what ways have you experienced stigma?
3. What barriers are there to disclosing your HIV status?
4. What can be gained by sharing your HIV status?

Stigma

?

1. What is stigma?
2. In what ways have you experienced stigma?

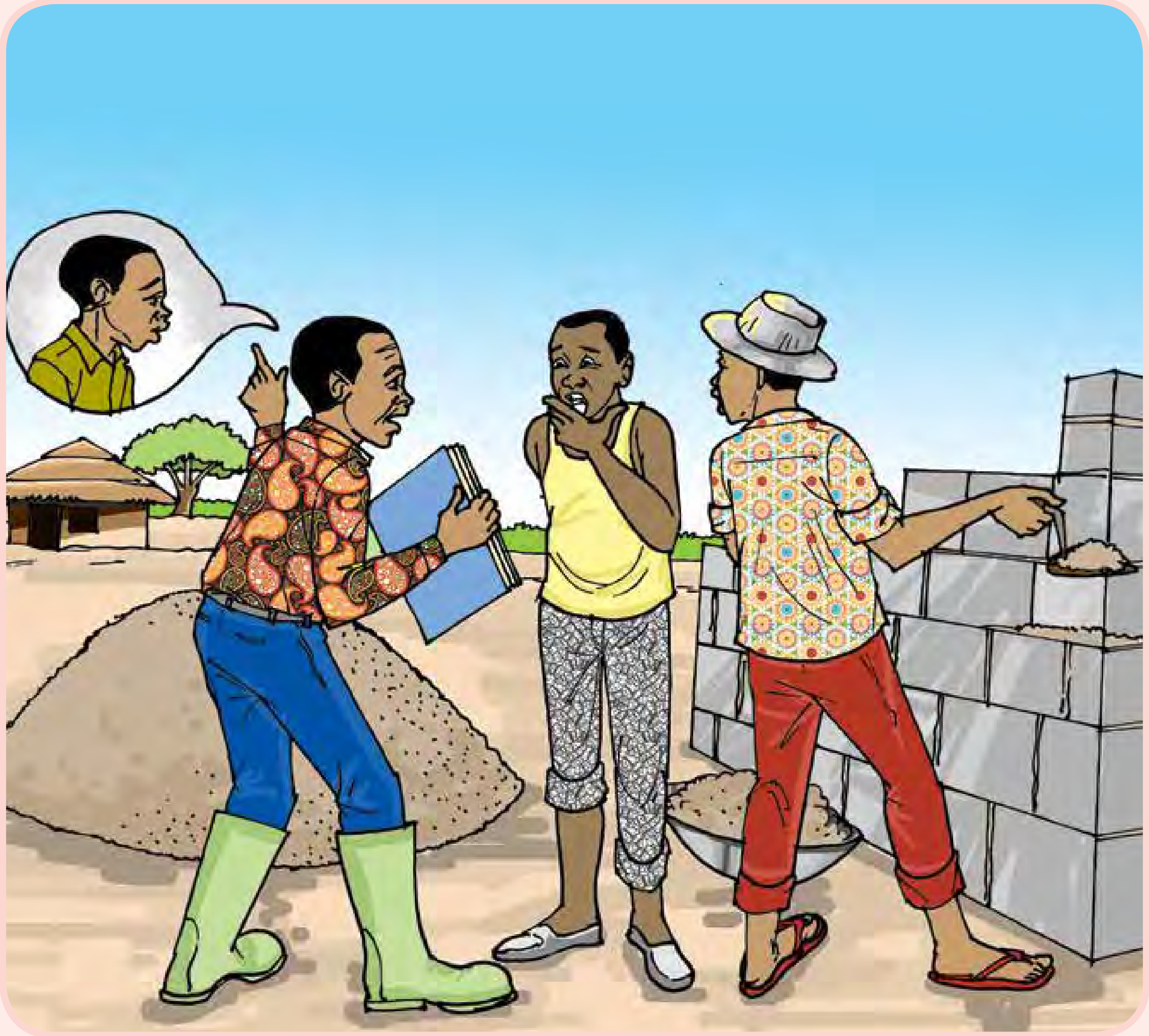

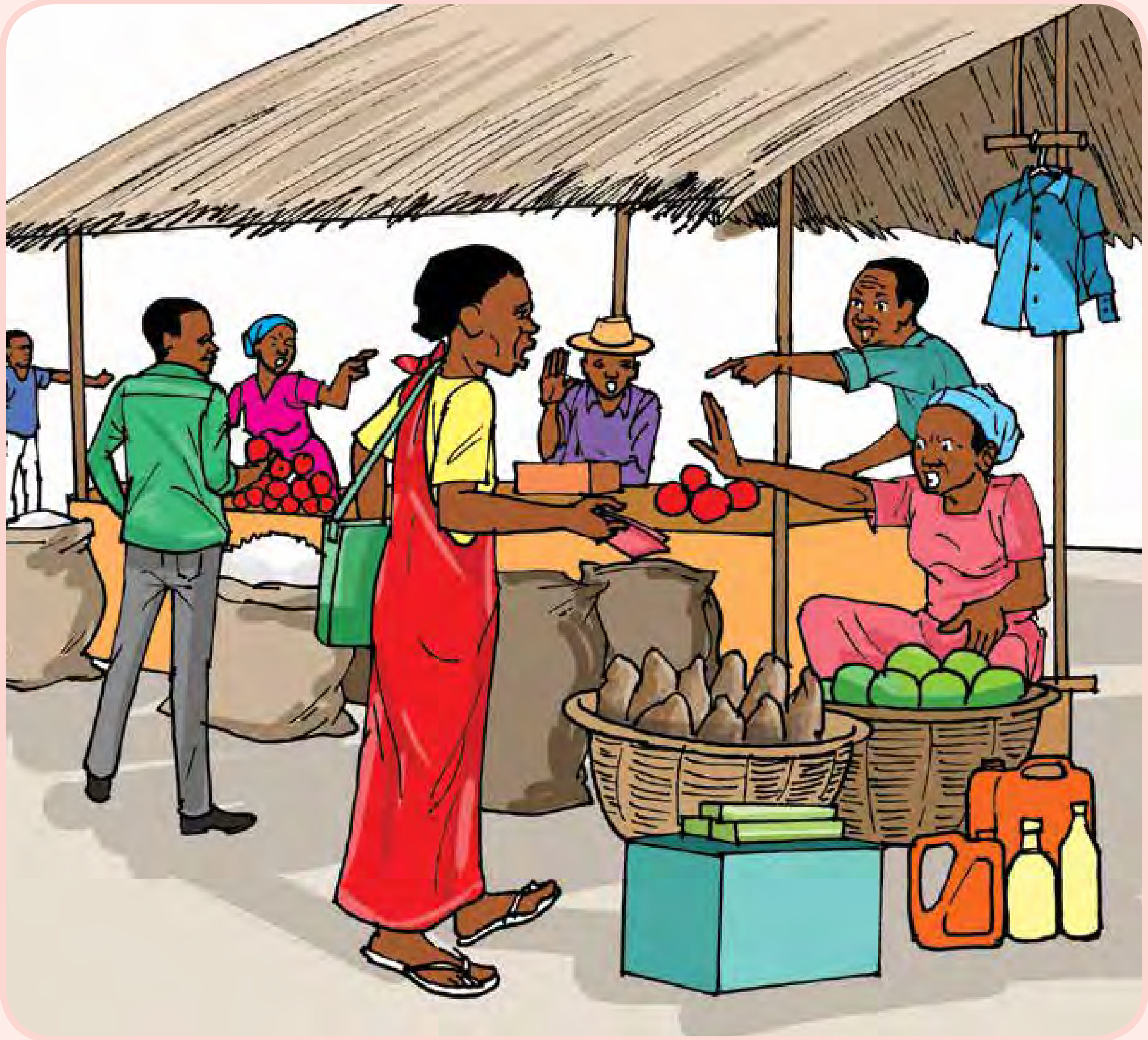

Facilitator's Guide

5 Pillars of Kanyakla Support

Use these illustrations to discuss the role of the Kanyakla and how it can support its members.

Ask the group: How can we support each other in each of the following areas:

1. **Emotional support** (supporting one another's feelings)
2. **Material support** (helping with material things like food, money or labour)
3. **Confidential support** (being able to keep a secret)
4. **Organisational support** (helping to organise medications)
5. **Spiritual support** (supporting one another's spiritual needs)

5 Pillars of Kanyakla Support

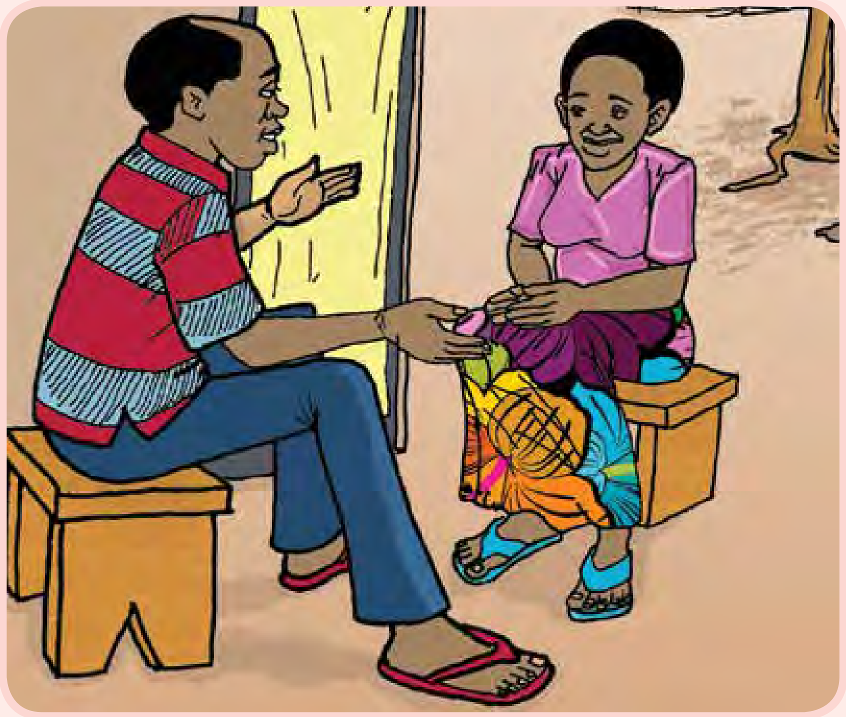
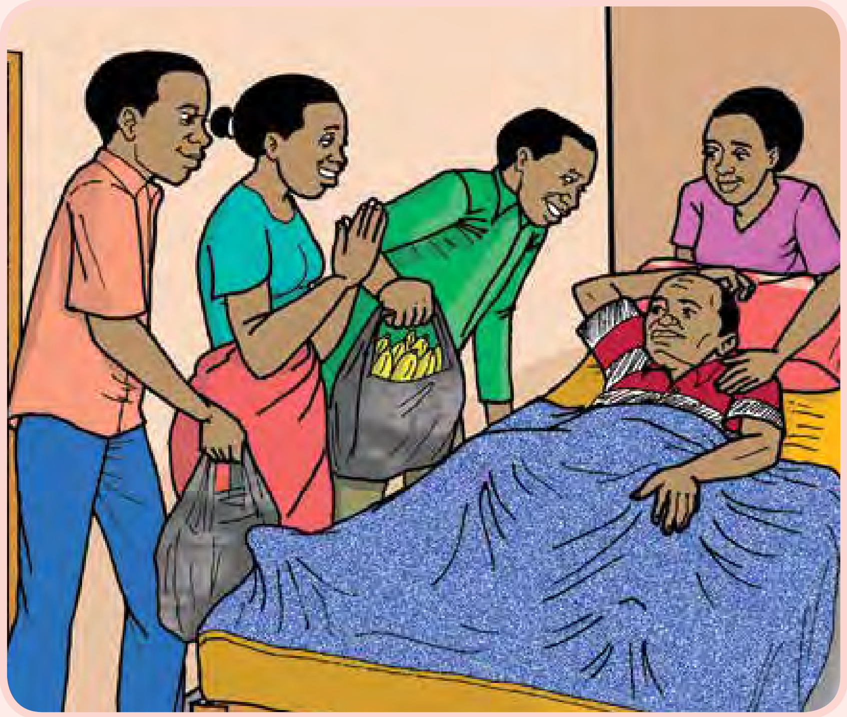
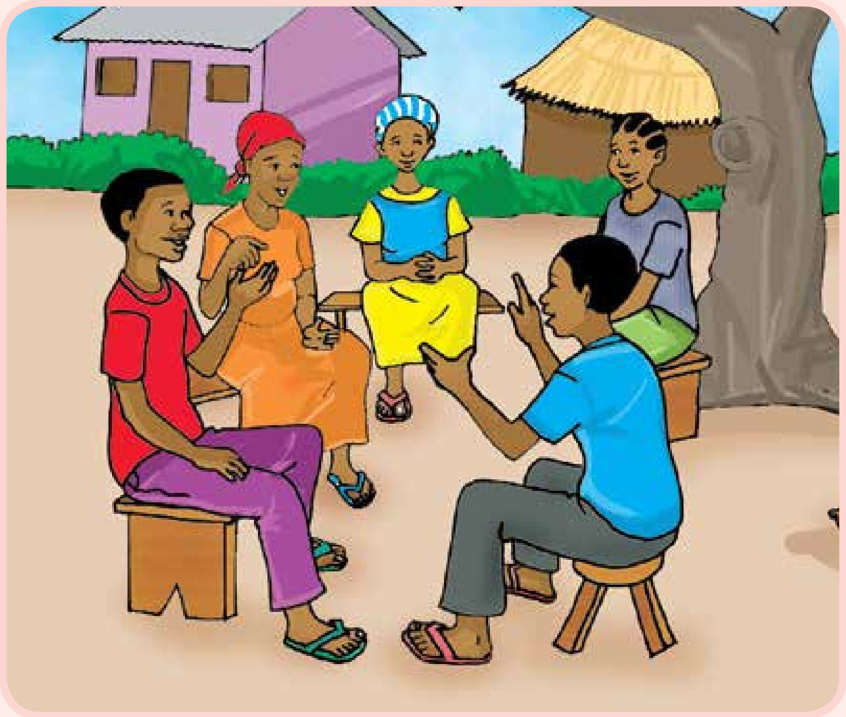
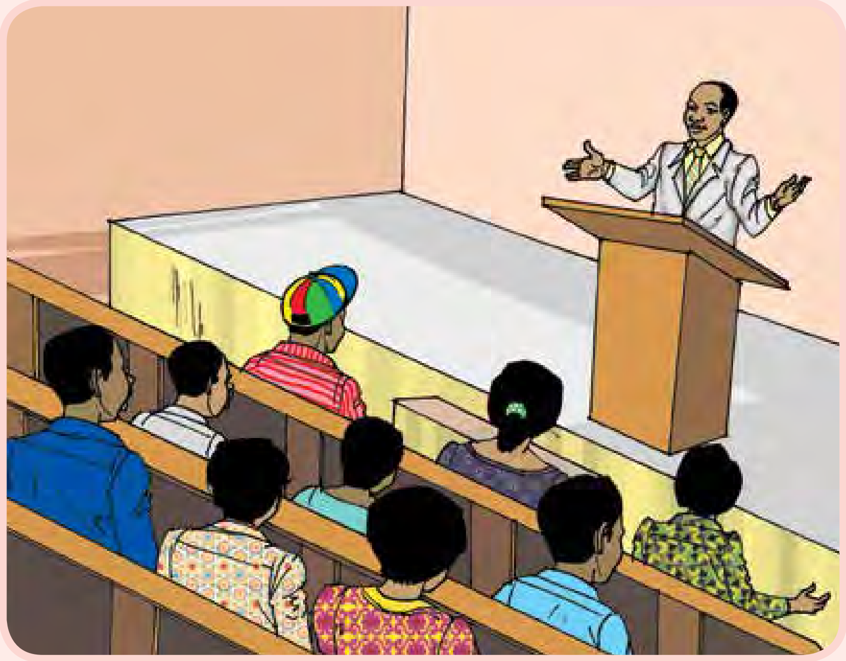
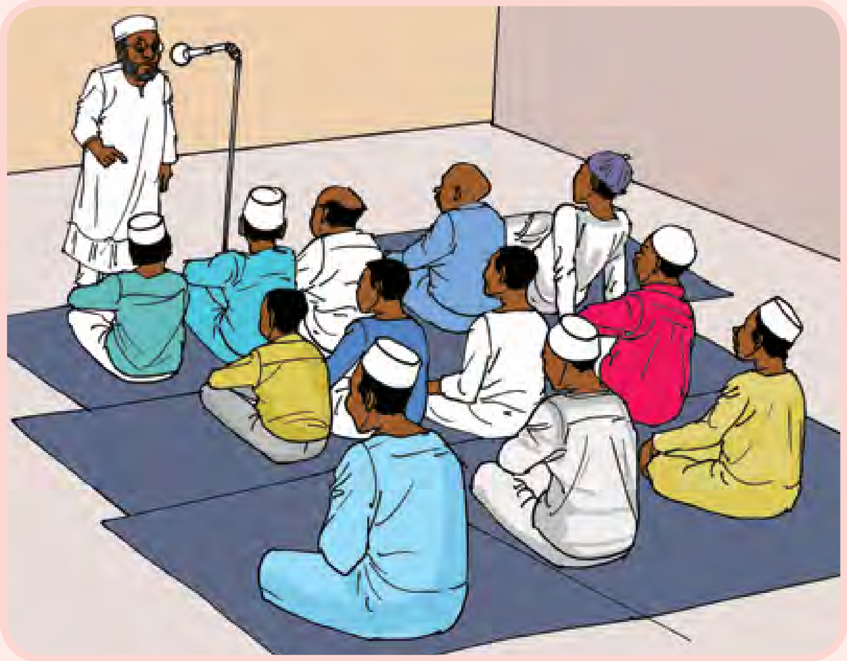
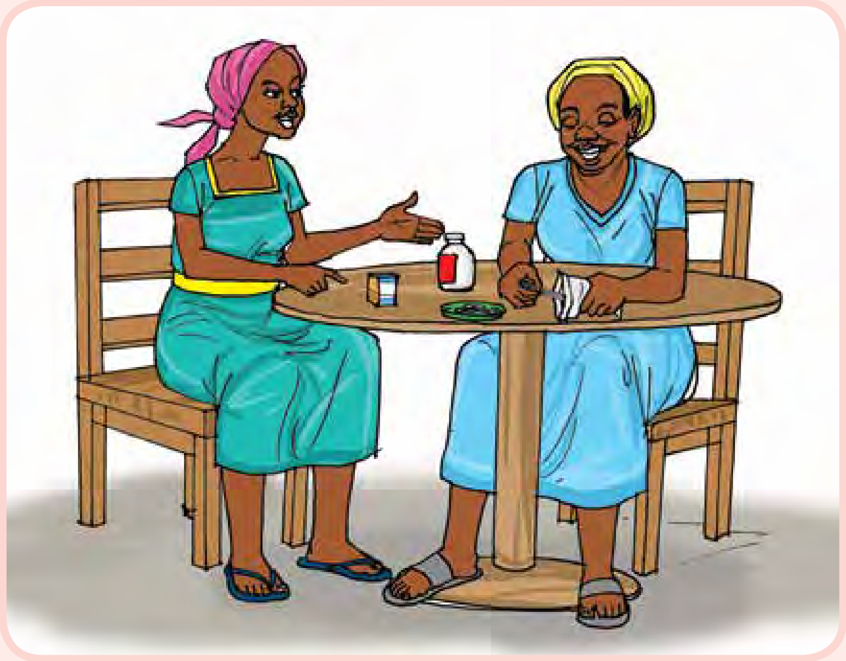

Facilitator's Guide

The Way Forward

Key message:

- Open lines of communication about sex early in your child's life. This is part of HIV prevention. It is not giving them permission to have sex.
- Open and honest discussion is the best way to combat stigma, which will improve individual and community health.
- Be open about your HIV status. It is not your fault you have HIV. It is your responsibility to take your ARVs and educate others about HIV.

Challenge: Go home and begin having a conversation with your children about sex and how to stay healthy. Discuss with a partner types of stigma you have experienced and how it made you feel.

Next Session: The next session is "Voluntary Group Disclosure".

Note: Remember to tell members the time and place of the next session.

Remember:

I know something about HIV, I can do something about it, and I can do something for someone else affected by HIV and AIDS!

The Way Forward

Key message:

- Open lines of communication about sex early in your child's life. This is part of HIV prevention. It is not giving them permission to have sex.
- Open and honest discussion is the best way to combat stigma, which will improve individual and community health.
- Be open about your HIV status. It is not your fault you have HIV. It is your responsibility to take your ARVs and educate others about HIV.

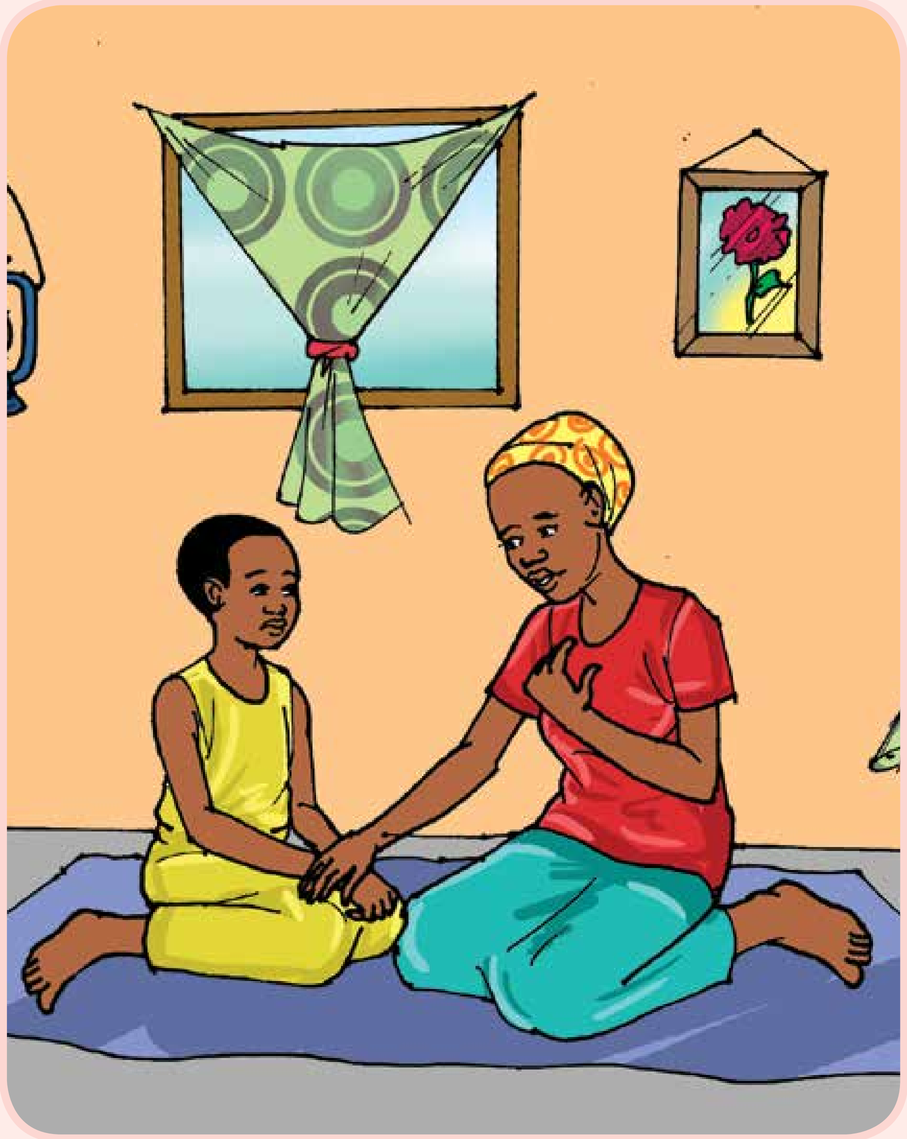

Challenge

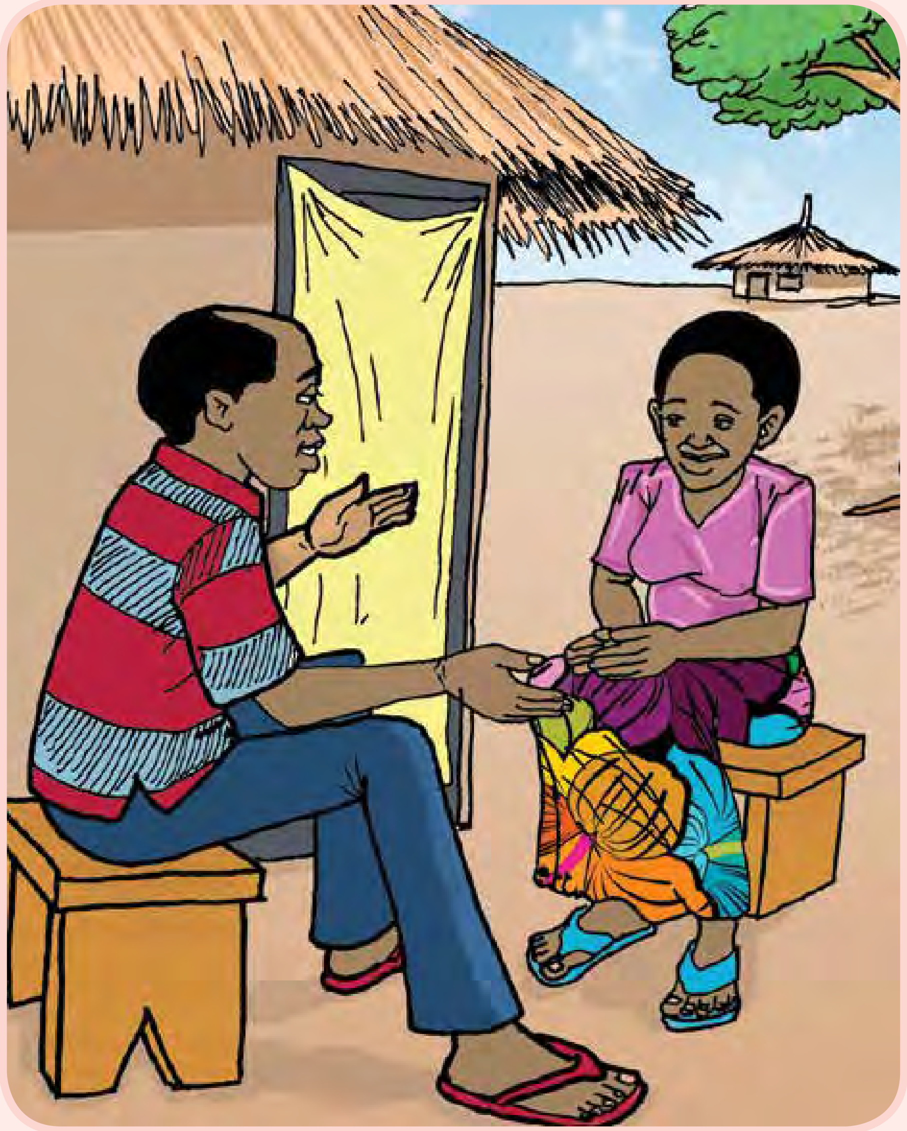

Next Session

I know something about HIV, I can do something about it, and I can do something for someone else affected by HIV and AIDS!

Facilitator's Guide

Group Disclosure

Objectives:

- Discuss confidentiality and how we maintain it
- Discuss why HIV status disclosure is important and strategies for disclosure

Session overview (120 minutes)

Activity	Time (minutes)	Objectives
Prayer and mindfulness	20	• Gather the group together and pray for a good session.<br>• Have the group reflect on the meditations below.
Wheel of Hope	15	• Review the material from the previous session.<br>• Use the questions and key messages to guide the review.
Class - Teach from the flipbook	45	• Review VCT (voluntary counselling and testing) and the importance of confidentiality<br>• Review group support.
Role play	20	• Demonstrate the importance of confidentiality.
Wisdom circle	15	• Review the objectives of the session. What was learnt?<br>• Schedule the next session.<br>• Review the handout.

Meditations:

"I enjoy being the messenger for God in terms of letting people know about HIV and AIDS." – ***Magic Johnson***

"No one is useless in this world who lightens the burdens of another." – ***Charles Dickens***

Session Seven: Group Disclosure

1 2 3 4 5 6 7 8

Objectives:

- Discuss confidentiality and how we maintain it
- Discuss why HIV status disclosure is important and strategies for disclosure

I know something about HIV, I can do something about it, and I can do something for someone else affected by HIV and AIDS!

Let's Role Play!

Importance of disclosure

Husband: You are HIV positive and taking ARVs, and you have told your wife. You have also disclosed to your neighbour. Your wife leaves for a few days, and you become ill due to the side effects of your ARVs. You consider stopping the ARVs.

Wife: You are HIV negative, and you know that your husband is HIV positive. You must leave him to travel for a few days.

Neighbour: You are HIV positive and taking ARVs. You know that your friend (the husband) is HIV positive and that his wife must leave for a few days. You go to check on the husband because you know his wife is away and his status.

Neighbour: "My friend, how are you doing?"

Husband: "I feel terrible. I think this medication is making me throw up. Maybe I should stop taking it."

Neighbour: "No. Keep taking it. I had the same reaction. It will go away after some time."

After the role play, encourage the group to discuss. Prompt discussion by asking: Has anyone had a similar experience?

Let's Role Play!

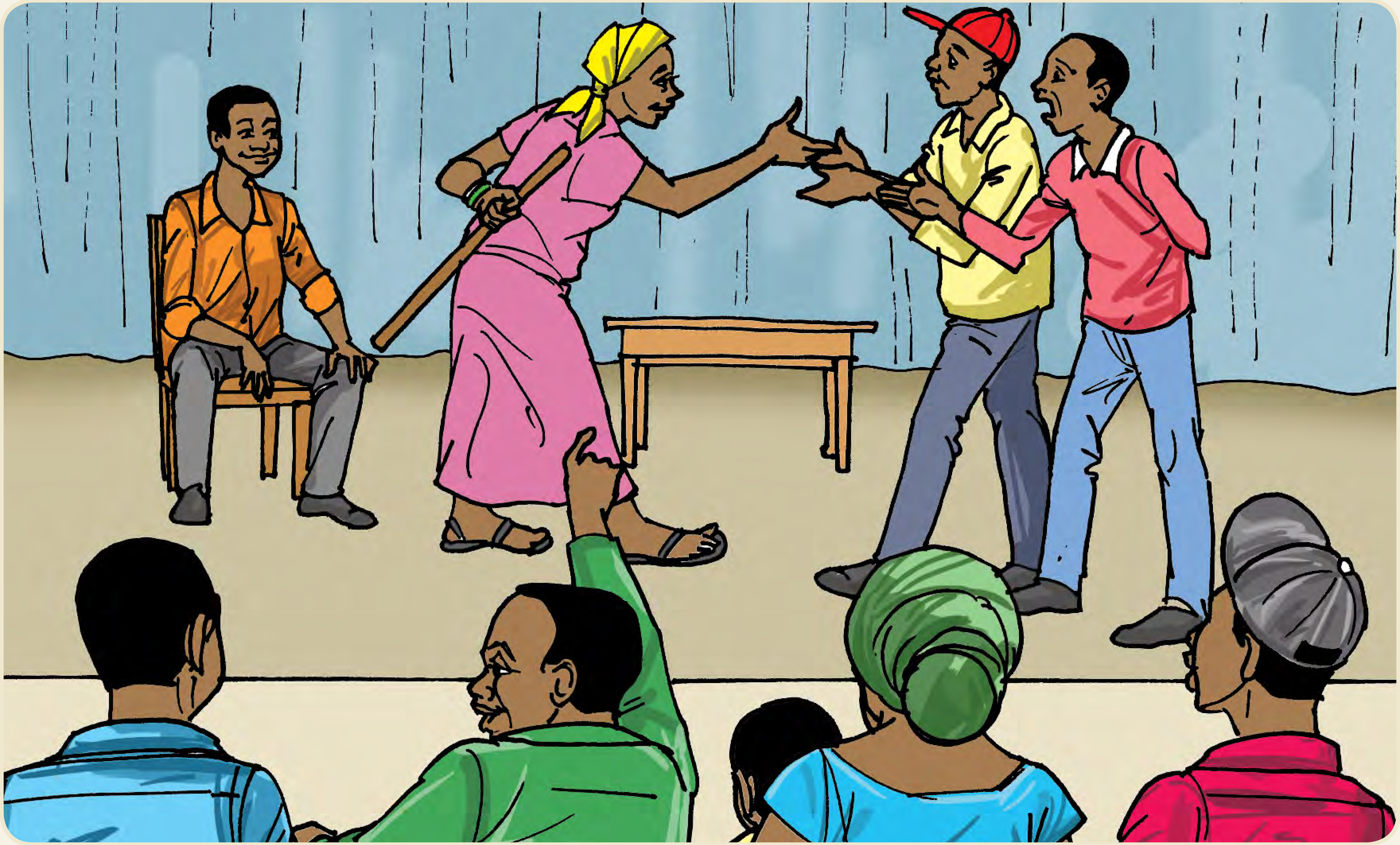

Facilitator's Guide

HIV Status Disclosure

Use the questions below to guide a discussion about the challenges to disclosing your HIV status as well as the potential benefits to understand why it is important.

Group Discussion

1. What barriers are there to disclosing your HIV status?
2. What can be gained by sharing your HIV status?
3. What is stigma?
4. In what ways have you experienced stigma?

HIV Status Disclosure

?

1. What barriers are there to disclosing your HIV status?
2. What can be gained by sharing your HIV status?

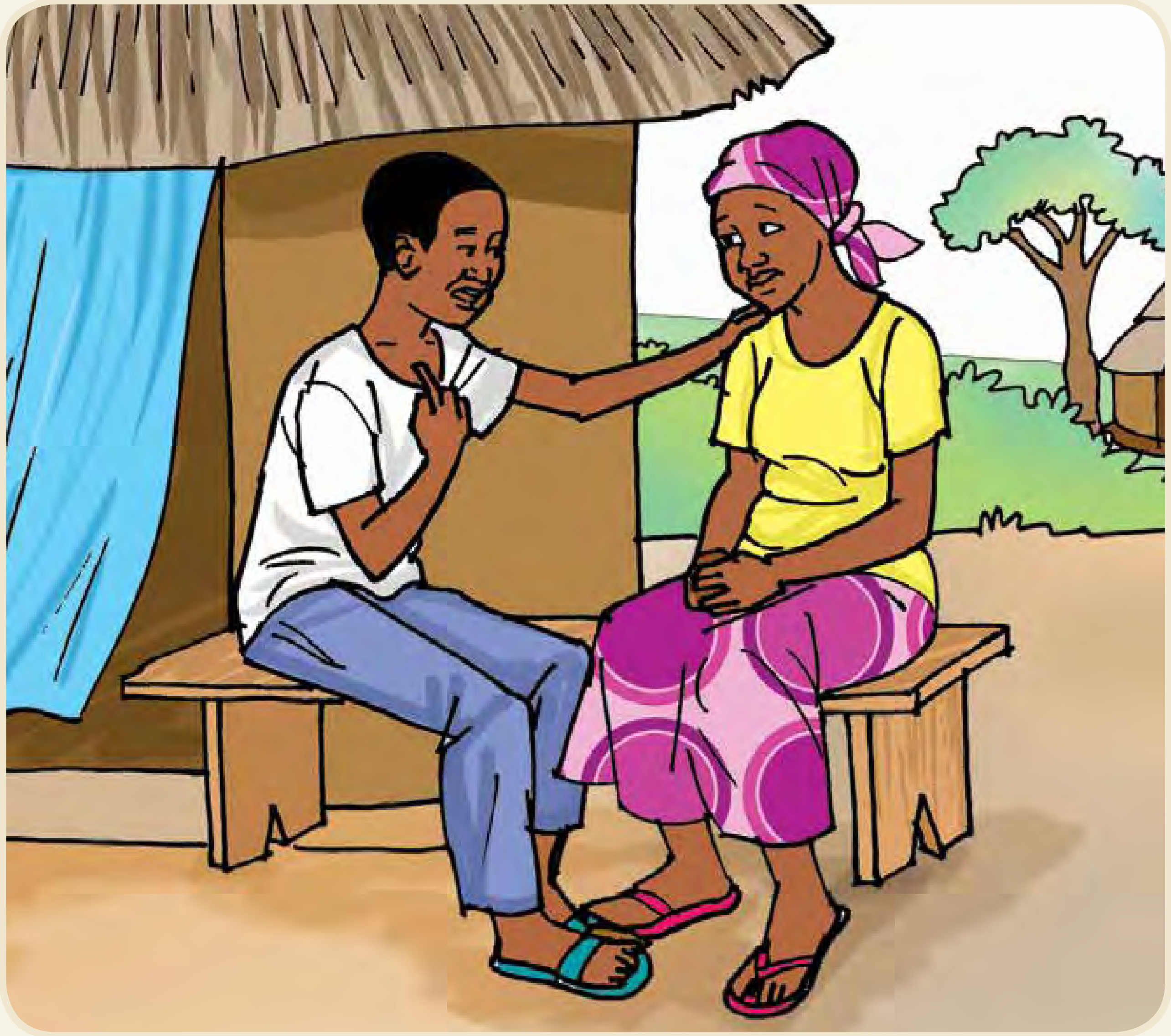

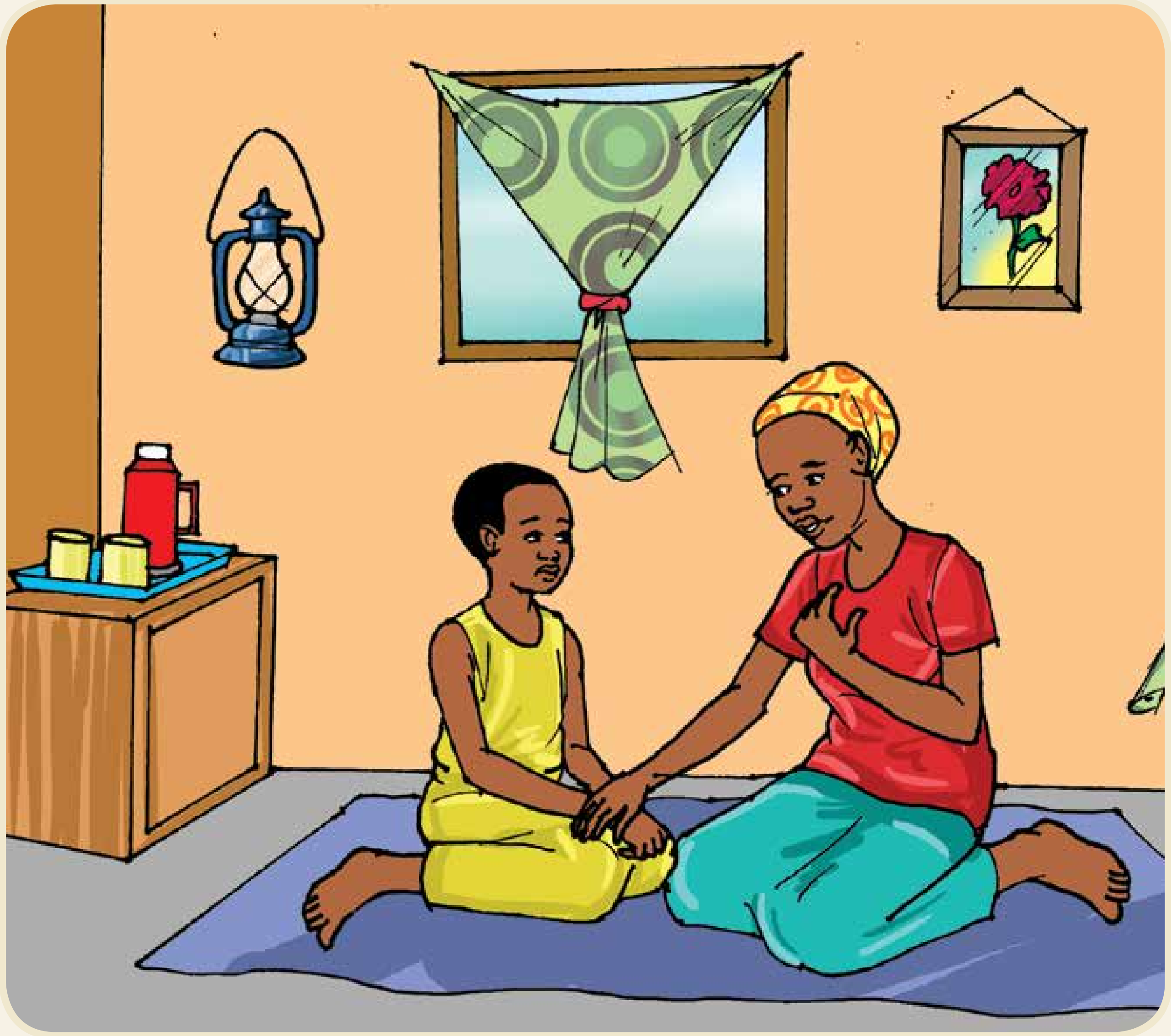

Team Building Exercises

Trust Fall

1. One person stands with their back facing the group and everyone else gathers behind the person.
2. The person falls backwards with their eyes closed and arms crossed over their chest.
3. The other group members catch the person falling backwards.
4. When the group catches the falling person everyone shouts “Wan Kanyakla!!!”

Key message:

- It is easy to support someone when you work together!

HIV Status Disclosure

Team Building Exercises

- Trust Fall

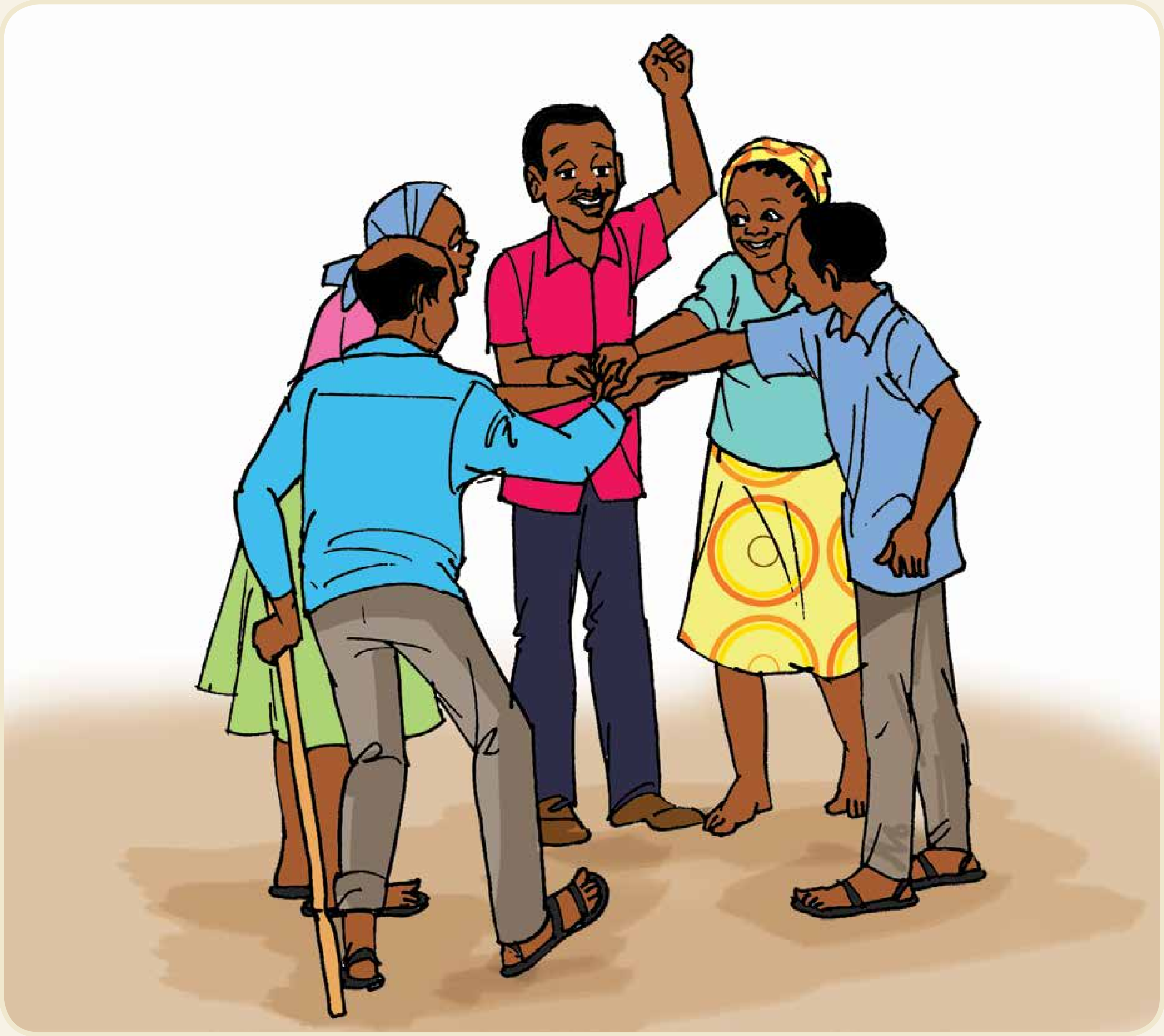

Facilitator's Guide

Confidentiality

Discuss the following questions with the group. There are no right or wrong answers. Encourage all group members to speak up.

1. What is confidentiality?
2. How do we practice and enforce confidentiality?
3. How can you be a good confidant?

Group Activity:

Ask the group to make a commitment to one another that they will practice confidentiality with things that are learnt in the group.

Have each member of the group in turn say to everyone else "I am a good confidant. I will keep things you tell me confidential."

Confidentiality

Group Discussion

1. What is confidentiality?
2. How do we practice and enforce confidentiality?
3. How can you be a good confidant?

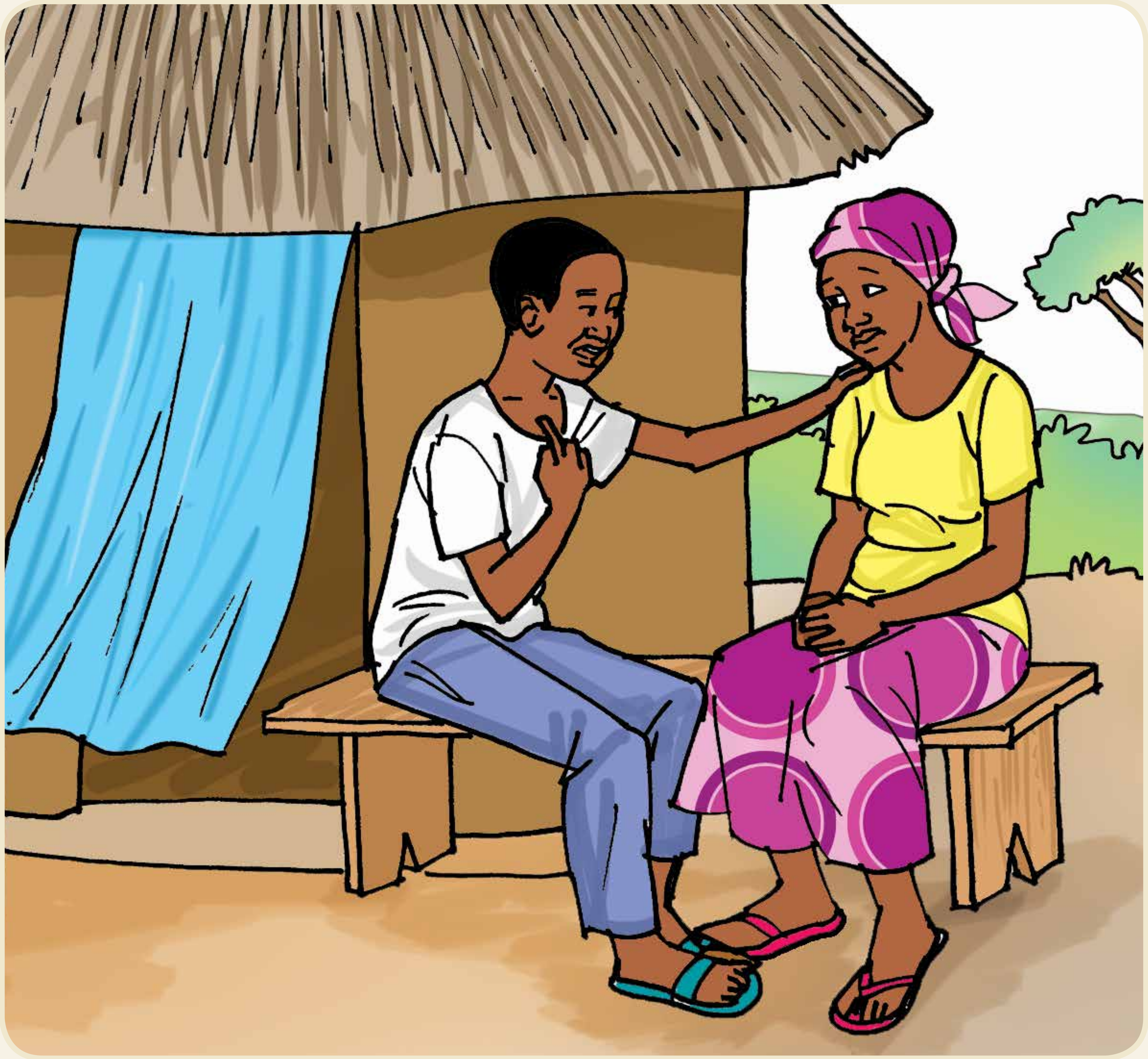

Facilitator's Guide

What to Expect

Ask the group the following questions:

1. What do you expect to hear?
2. What are your fears and expectations?
3. How will you react if you are HIV+?
4. How will you support others in your Kanyakla?
5. How will you support others not in a Kanyakla?

If some group members are talking while others remain silent, go around in a circle and ask each group member to answer at least one of these questions to make sure everyone has a chance to share.

1. At the end of today's session, we will schedule a group HIV testing session with a VCT counsellor.
2. On the specified day, we will meet with the VCT counsellor. He or she will first explain how group testing and disclosure works and will answer questions.
3. Everyone will then be tested at the same time. You will each pair up with a partner and first disclose your test results to your partner.
4. Then the whole group will get together and your partner will disclose your test results to the group. (For example, "Today I learnt that John is positive")

Ask the group if they have any questions or concerns. Take time to discuss these.

What to Expect

Group Disclosure:

- Group counselling
- Disclosure testimonial
- Disclosure to Kanyakla group

?

1. What do you expect to hear?
2. What are your fears and expectations?
3. How will you react if you are positive?
4. How will you support others in your Kanyakla?
5. How will you support others not in a Kanyakla?

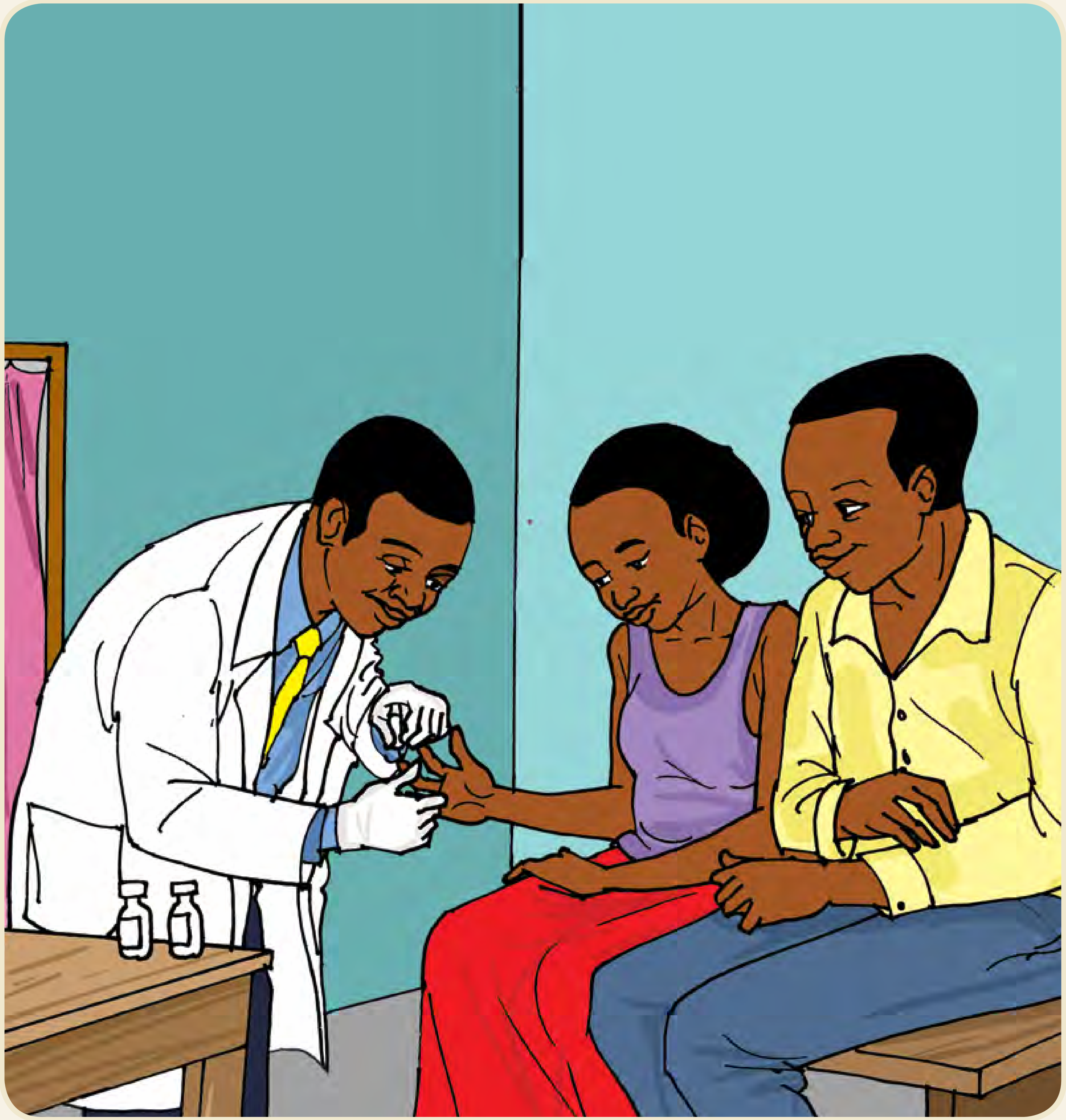

Facilitator's Guide

The Way Forward

Key message:

- Disclosing your HIV status may be difficult, but it can also be beneficial.
- Confidentiality is an important part of healthy, trusting, relationships.
- Breach of confidentiality causes stigma. This leads to poor medication adherence.
- Group testing and disclosure will follow this session.

Challenge: Find someone who is not in the Kanyakla that you want to disclose to. If there is anyone who is HIV+ in the Kanyakla who does not have care, help him or her get care.

Next Session: The next session will be about the future of the Kanyakla group after group disclosure.

Note: Remember to tell the group the time and place of the next session.

Remember:

I know something about HIV, I can do something about it, and I can do something for someone else affected by HIV and AIDS!

The Way Forward

Key message:

- Disclosing your HIV status may be difficult, but it can also be beneficial.
- Confidentiality is an important part of healthy, trusting, relationships.
- Breach of confidentiality causes stigma. This leads to poor medication adherence.
- Group testing and disclosure will follow this session.

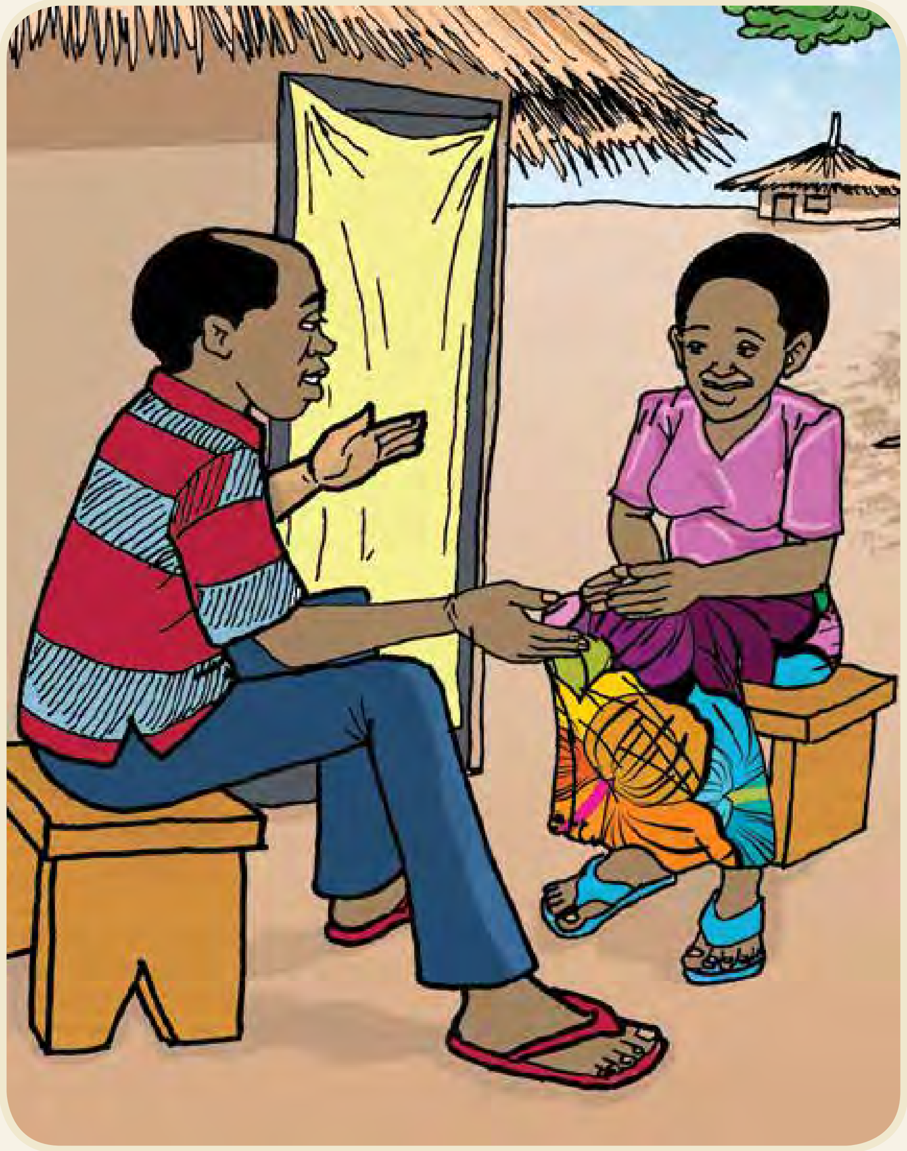

Challenge

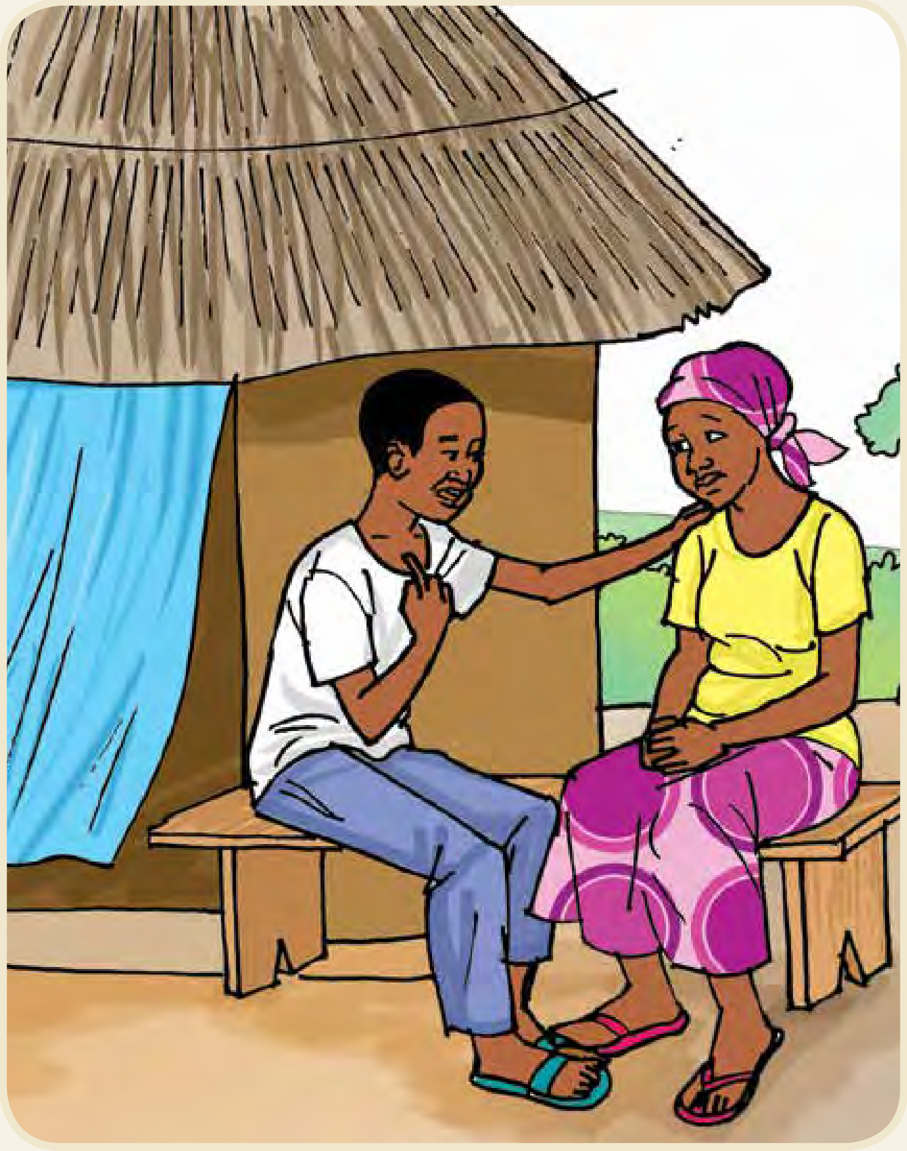

Next Session

I know something about HIV, I can do something about it, and I can do something for someone else affected by HIV and AIDS!

Facilitator's Guide

The Way Forward

Objectives:

- Discuss the group disclosure process and challenges.
- Discuss the future of the Kanyakla group.

Session overview (90 minutes)		
**Activity**	**Time (minutes)**	**Objectives**
Prayer and meditation	20	• Gather the group together and pray for a good session.<br>• Give the group some meditations to reflect upon.
Review previous session	15	• Review the material from the previous session.<br>• Use the questions and key messages to guide the review.
Group discussion	20	• Discuss the disclosure session.<br>• Discuss how the Kanyakla can support those who are HIV-positive.
Class- Teach from the flipbook	20	• Teach about how to remain healthy.
Wisdom circle	15	• Review the objectives of the session. What was learnt?<br>• Distribute a handout for the Kanyakla if provided.<br>• Discuss how the Kanyakla can continue to meet.

Meditations:

"We have flown the air like birds and swum the sea like fishes, but have yet to learn the simple act of walking the earth like brothers." – *Martin Luther King, Jr.*

"There is nothing more beautiful than someone who goes out of their way to make life beautiful for others." – *Mandy Hale*

Session Eight: The Way Forward

1 2 3 4 5 6 7 **8**

Objectives:

- Discuss the group disclosure process and challenges.
- Discuss the future of the Kanyakla group.

I know something about HIV, I can do something about it, and I can do something for someone else affected by HIV and AIDS.

Facilitator's Guide

HIV Status Disclosure:

Use this time to check in on how people felt after disclosing their HIV status. Identify any problems that may have come up and discuss how the Kanyakla can help them.

Use the questions below to guide the discussion.

?

1. How did you feel during group disclosure?
2. What has changed since disclosure?
3. For those who are HIV-positive, are you getting the care you need?
4. How can the Kanyakla support those who are HIV-positive?
5. How can the Kanyakla support those who are HIV-negative?

HIV Status Disclosure

?

1. How did you feel during group disclosure?
2. What has changed since disclosure?
3. For those who are HIV-positive, are you getting the care you need?
4. How can the Kanyakla support those who are HIV-positive?
5. How can the Kanyakla support those who are HIV-negative?

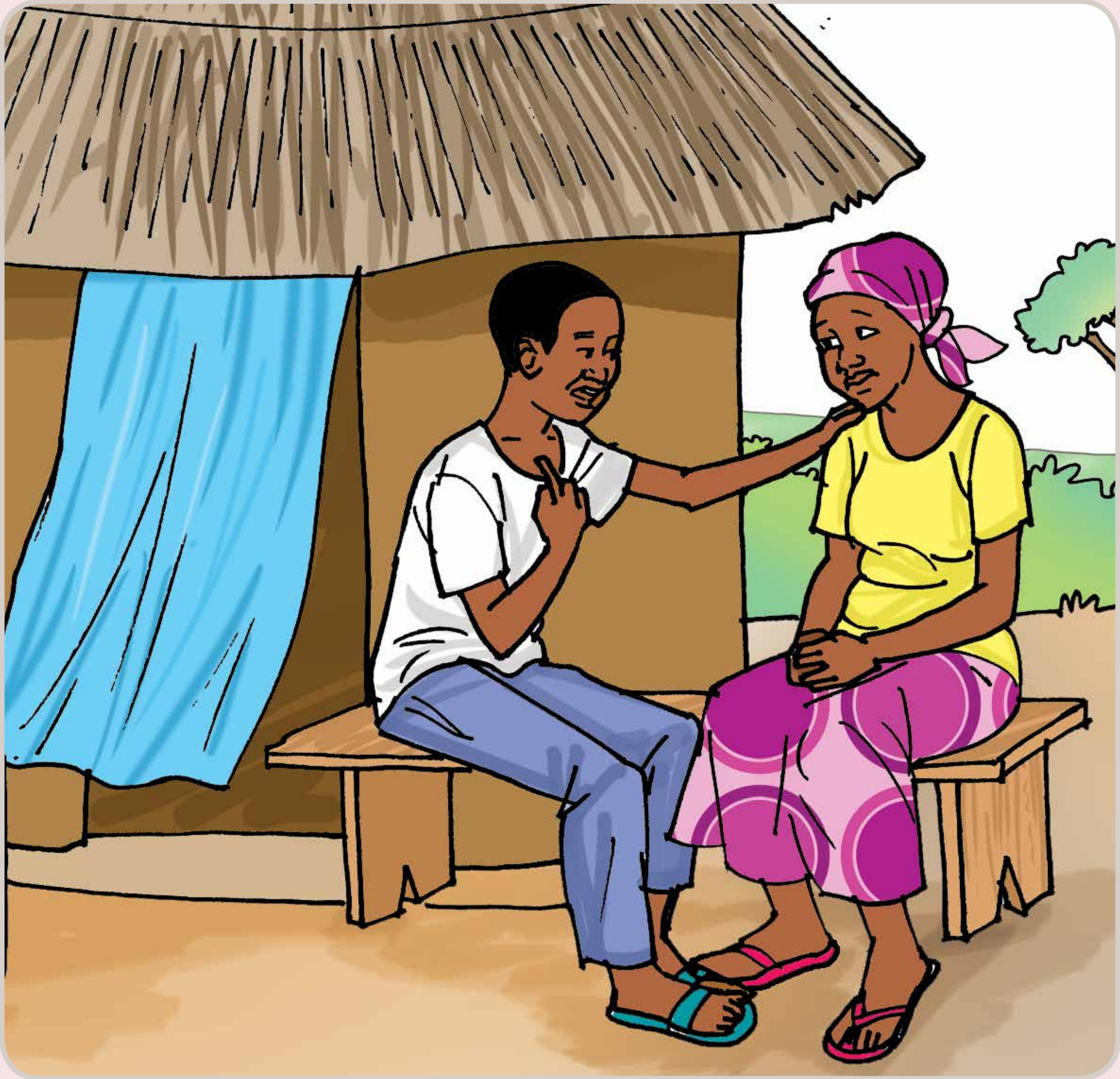

The Way Forward: Staying Healthy

- Use the page to write down the ways that Kanyakla members can help one another stay healthy.
- All group members should discuss, regardless of whether they are HIV-positive or HIV-negative.
- Write down ideas for BEHAVIOURS that the group can support and SOCIAL SUPPORT that the group can provide. There are no right or wrong answers!

Below are some ideas to discuss if they are not brought up by the group.

Behaviours

1. Practice safe sex
2. Take medications and seek health care
3. Partner testing
4. Voluntary Medical Male Circumcision
5. Talk to kids about sex
6. Proper nutrition

Social Support

1. Check in on community members
2. Teach your neighbours what you have learnt
3. Ask for help when you need it

The Way Forward: Staying Healthy

Behaviours

Social Support

Moving Forward

Discuss what the role of the Kanyakla will be moving forward.

1. Will the group continue to meet?
2. What activities will the Kanyakla participate in together? (For example, shall they make a garden? Form a food security plan? Open a bank account?)
3. Even if the Kanyakla doesn't meet, they can still check in and support each other.

?

1. What role can the Kanyakla have moving forward?
2. What could you do to remain active as a Kanyakla group?

Moving Forward

1. What role can the Kanyakla have moving forward?
2. What could you do to remain active as a Kanyakla group?

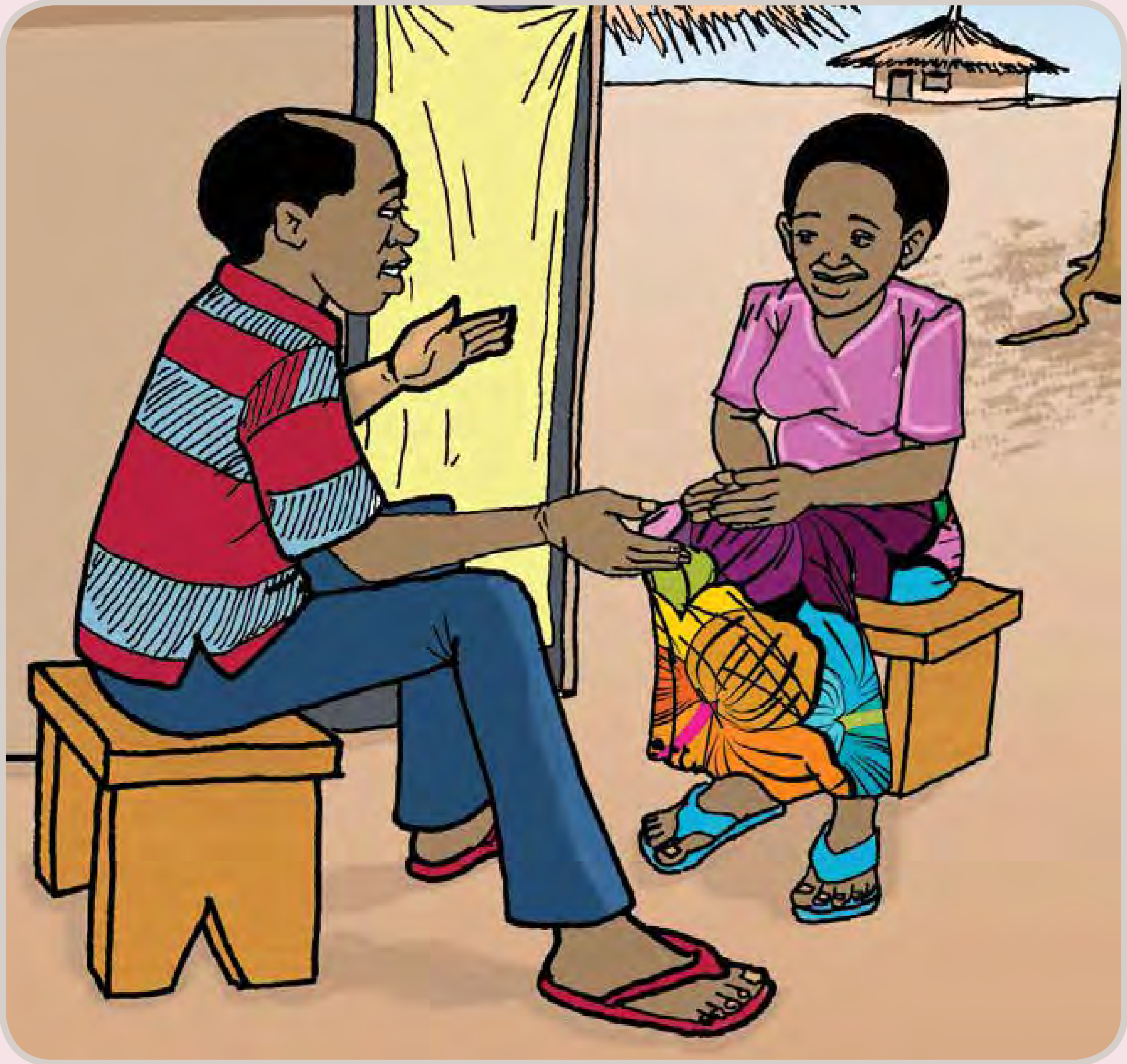

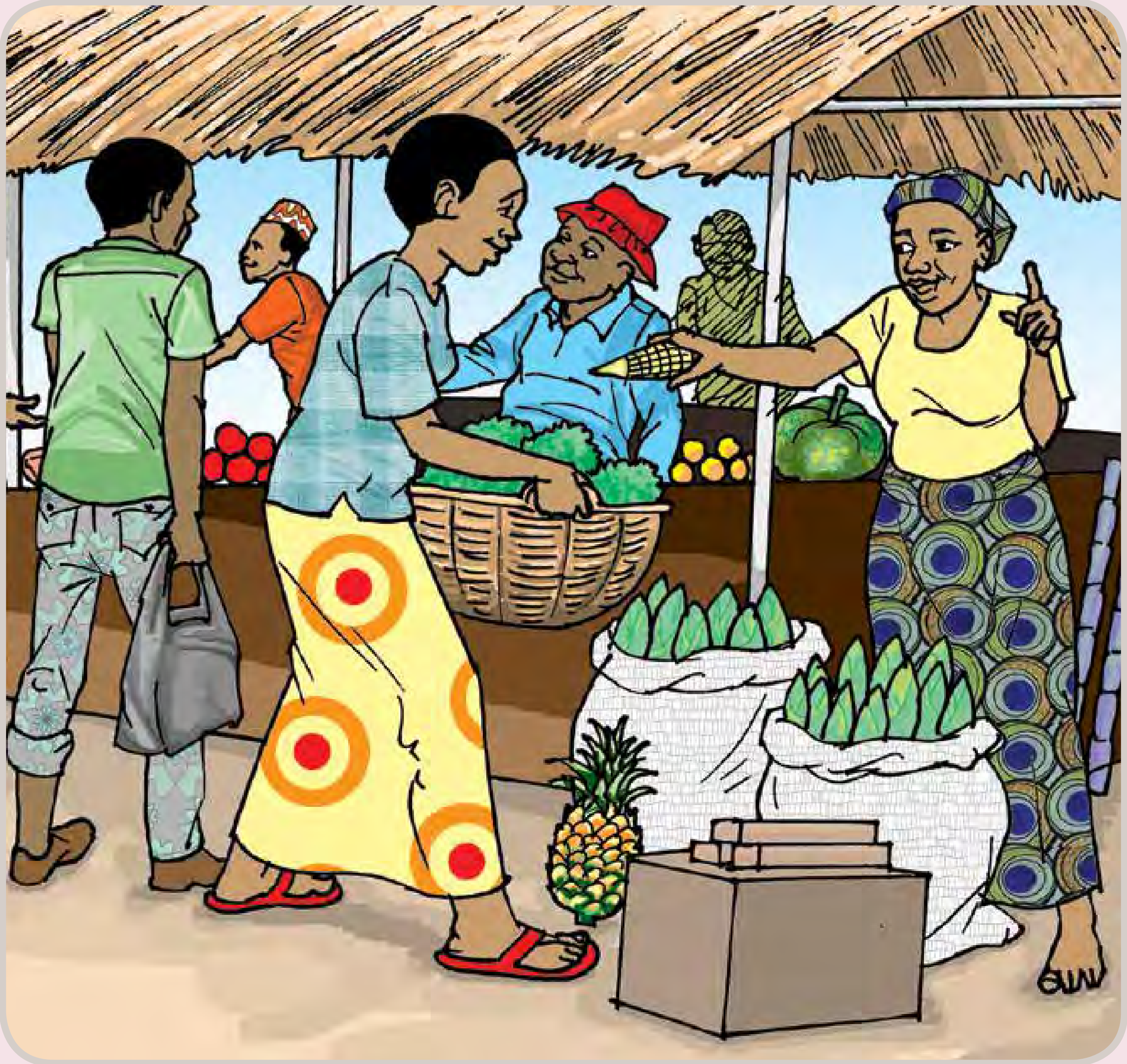

The Way Forward

Key message:

- Kanyakla groups can continue to be a form of support for each other and the community.
- Continue to educate yourself and your community about HIV.
- As a trained Kanyakla, be a role model for your community.
- Take steps to stay healthy and help others to stay healthy.

Challenge: Discuss with your Kanyakla how you can help each other stay healthy. Some examples are to encourage treatment adherence, visit each other, help with paying for medications/transport, and develop a food security plan.

Remember to thank the group for their participation in the Kanyakla training sessions.

Remember:

I know something about HIV, I can do something about it, and I can do something for someone else affected by HIV and AIDS!

The Way Forward

Key message:

- Kanyakla groups can continue to be a form of support for each other and the community.
- Continue to educate yourself and your community about HIV.
- As a trained Kanyakla, be a role model for your community.
- Take steps to stay healthy and help others to stay healthy.

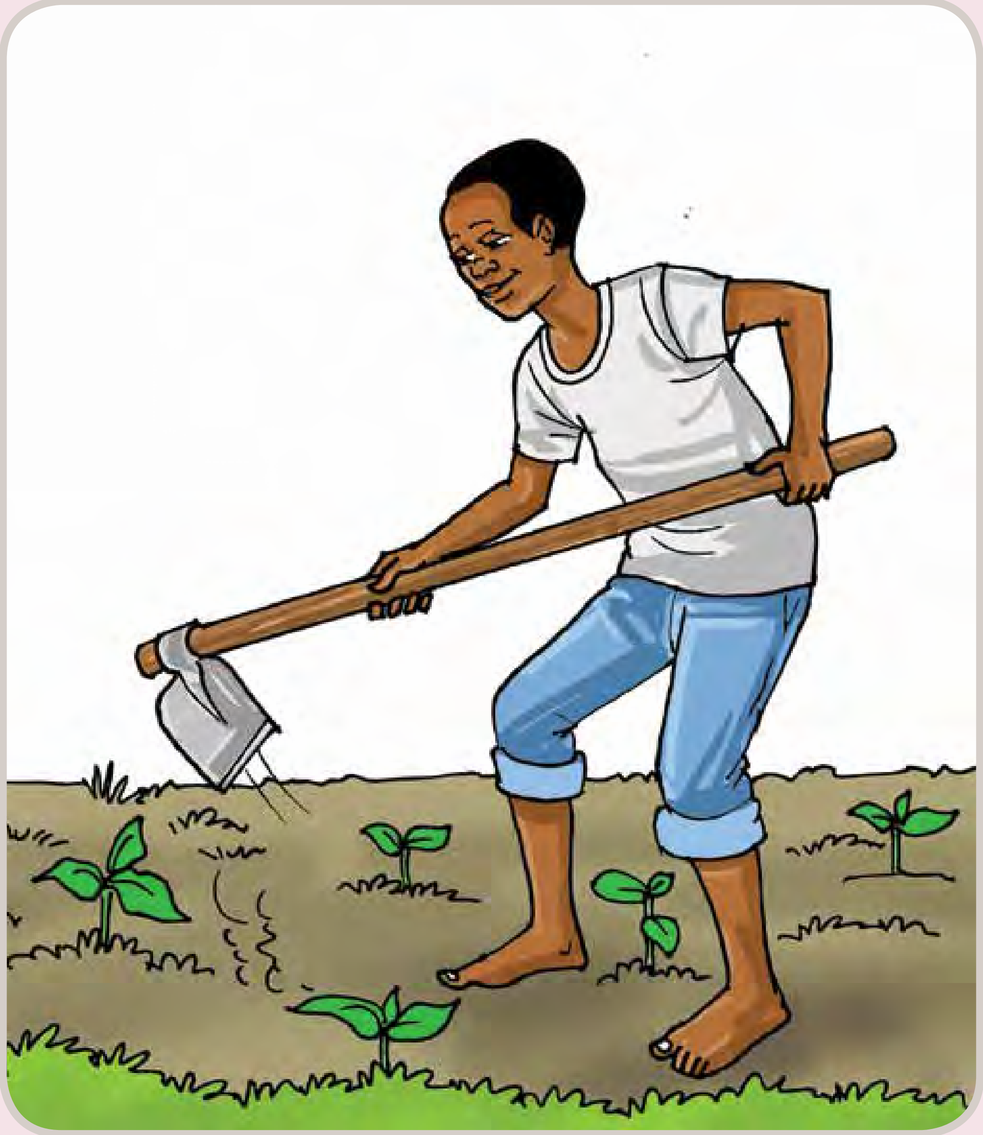

Challenge

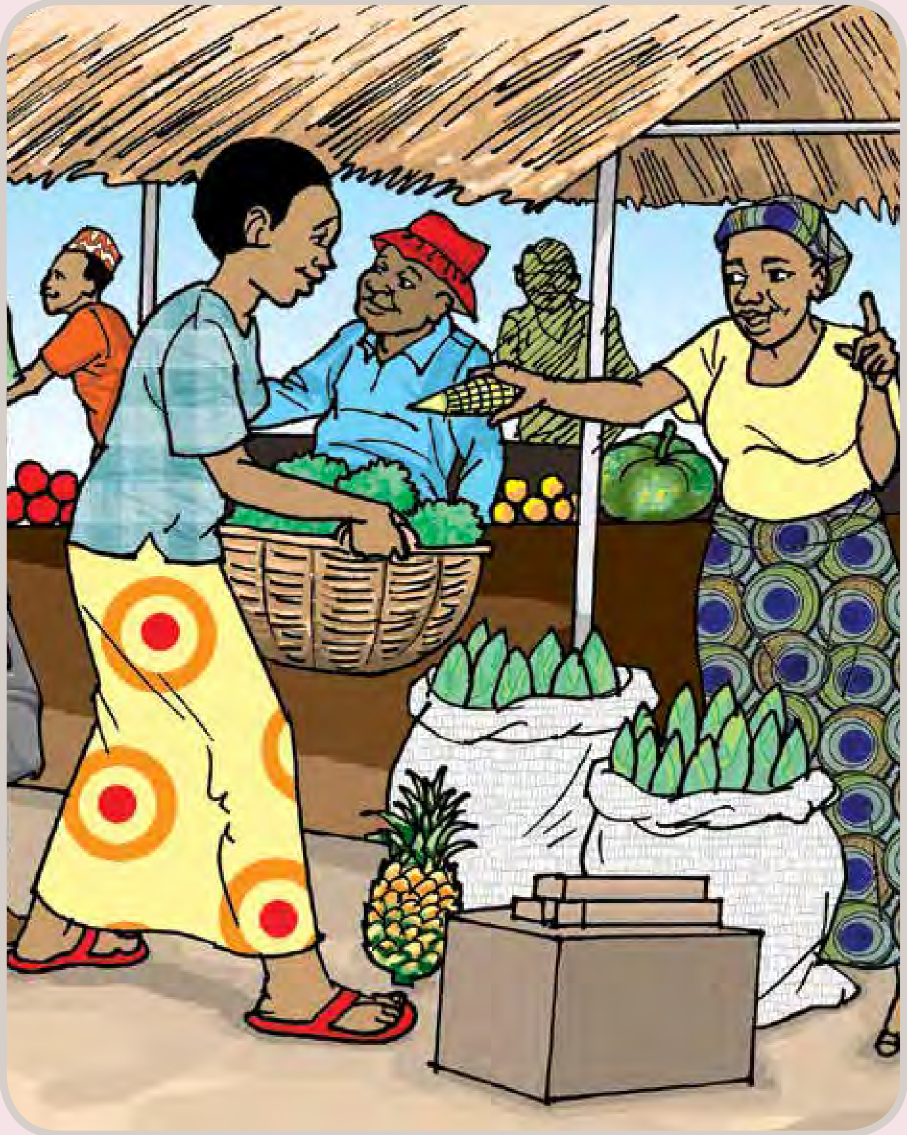

I know something about HIV, I can do something about it, and I can do something for someone else affected by HIV and AIDS.
