# Supplementary material for: The Kanyakla study: Randomized controlled trial of a microclinic social network intervention for promoting engagement and retention in HIV care in rural western Kenya
Source: PLoS One. 2021 Sep 13;16(9):e0255945. doi: 10.1371/journal.pone.0255945 (PMC8437299; doi:10.1371/journal.pone.0255945)
Supplement: S1 Table — (DOCX) [file pone.0255945.s002.docx]

**S1 Table. Reasons for non-enrollment (n=46)**

|  | **n** | **%** |
| --- | --- | --- |
| Plans to move | 11 | 24% |
| Too busy | 8 | 17% |
| Not interested in research | 5 | 11% |
| Does not want to participate in a microclinic group | 4 | 9% |
| Does not want Ekialo Kiona programs | 4 | 9% |
| No reason given | 2 | 9% |
| Concerned about disclosure | 2 | 4% |
| Lives outside islands | 2 | 4% |
| Refused to talk with study team | 2 | 4% |
| Died before study team could enroll | 2 | 4% |
| Travels too frequently to participate | 1 | 2% |
| No friends with whom to participate | 1 | 2% |
| Mobility problems due to age | 1 | 2% |
|  |  |  |
