# Supplementary material for: The Kanyakla study: Randomized controlled trial of a microclinic social network intervention for promoting engagement and retention in HIV care in rural western Kenya
Source: PLoS One. 2021 Sep 13;16(9):e0255945. doi: 10.1371/journal.pone.0255945 (PMC8437299; doi:10.1371/journal.pone.0255945)
Supplement: S2 Table — (DOCX) [file pone.0255945.s003.docx]

**S2 Table. Baseline characteristics of enrolled vs non-enrolled participants**

| **Characteristic** | **Enrolled (n=304)** | | **Not Enrolled (n=46)** | |
| --- | --- | --- | --- | --- |
| Men, n (%) | 97/304 | 32% | 9/46 | 20% |
| Women, n (%) | 207 | 68% | 37/46 | 80% |
| Age (yrs), median (IQR) | 34 | 29-43 | 35 | 28-41 |
| Age category (yrs), n (IQR) |  |  |  |  |
| 18-24 | 32/304 | 11% | 6/46 | 13% |
| 25-49 | 232/304 | 76% | 35/46 | 76% |
| ≥50 | 40/304 | 13% | 5/46 | 11% |
| HIV Status Disclosure |  |  |  |  |
| Disclosed HIV status to anyone else, n (%) | 285/304 | 94% | 3/46 | 7% |
| Number of people disclosed HIV status, median (IQR) | 4 | 2-8 | 0 | 0-0 |
| Clinical Characteristics |  |  |  |  |
| Time since clinic enrollment (yrs), median (IQR) | 4.0 | 1.9-6.8 | 2.0 | 0.4-4.9 |
| Initiated on ART by clinic records, n (%) | 275/304 | 90% | 41/46 | 89% |
| Taking ART by self-report, n (%) | 270/304 | 89% | 3/46 | 7% |
| Time since ART initiation by clinic records (yrs), median (IQR) | 2.9 | 1.1-4.7 | 1.4 | 0.4-4.4 |
