# Supplementary material for: The Kanyakla study: Randomized controlled trial of a microclinic social network intervention for promoting engagement and retention in HIV care in rural western Kenya
Source: PLoS One. 2021 Sep 13;16(9):e0255945. doi: 10.1371/journal.pone.0255945 (PMC8437299; doi:10.1371/journal.pone.0255945)
Supplement: S2 File — (DOC) [file pone.0255945.s005.doc]

**S2. Study Protocol**

The Kanyakla Study: a social network intervention for promoting engagement and retention in HIV care

**I.** **Project Title** [**2**](#__RefHeading___Toc49526429)

II. Investigator and Institutional Affiliations [2](#__RefHeading___Toc49526430)

III. Abstract / Summary [3](#__RefHeading___Toc49526431)

IV. Introduction/Background [3](#__RefHeading___Toc49526432)

V. Justification [5](#__RefHeading___Toc49526433)

VI. Research Questions [6](#__RefHeading___Toc49526434)

VII. Research Objectives [7](#__RefHeading___Toc49526435)

A. General Objectives [7](#__RefHeading___Toc49526436)

B. Specific Objectives [7](#__RefHeading___Toc49526437)

VIII. Design and Methodology [7](#__RefHeading___Toc49526438)

A. Study site [7](#__RefHeading___Toc49526439)

B. Study population [8](#__RefHeading___Toc49526440)

C. Sampling [8](#__RefHeading___Toc49526441)

D. Subject Recruitment and Enrollment (Figure 1) [10](#__RefHeading___Toc49526442)

E. Community Mobilization [12](#__RefHeading___Toc49526443)

F. Procedures [13](#__RefHeading___Toc49526444)

G. Data Analysis [19](#__RefHeading___Toc49526445)

H. Project Sustainability [20](#__RefHeading___Toc49526446)

IX. Ethical Considerations [21](#__RefHeading___Toc49526447)

X. Data Management [23](#__RefHeading___Toc49526448)

XI. Time Frame/Duration of the Project [24](#__RefHeading___Toc49526449)

XII. Expected Application of the Results [24](#__RefHeading___Toc49526450)

XIII. References [24](#__RefHeading___Toc49526451)

XIV. Budget [25](#__RefHeading___Toc49526452)

XV. Study Team Roles [26](#__RefHeading___Toc49526453)

XVI. List of Appendices [28](#__RefHeading___Toc49526454)

XVII. English Appendices [29](#__RefHeading___Toc49526455)

# Project Title

The Kanyakla Study: a social network intervention for promoting engagement and retention in HIV care

*Kanyakla* (definition) – team or together in Dholuo

# Investigator and Institutional Affiliations

| **Name** | **Organization** | **Role on Project** |
| --- | --- | --- |
| Elizabeth Bukusi, MB.ChB., M. Med, MPH, PhD, PGD (Research ethics) | Kenya Medical Research Institute (KEMRI) | KEMRI-Principal Investigator |
| Craig Cohen, MD, MPH | University of California, San Francisco (UCSF) | Co-Investigator |
| Matt Hickey, MD candidate | University of California, San Francisco (UCSF) | Co-Investigator |
| Charles Salmen, MD, MPhil (Oxon.) | The Organic Health Response (OHR) | Co-Investigator |
| Dan Omollo, BSc, MPH candidate | The Organic Health Response (OHR) | Co-Investigator |
| Katie Fiorella, MPH | The Organic Health Response (OHR) | Co-Investigator |
| Dr. Betty Njoroge | Kenya Medical Research Institute (KEMRI) | Co-Investigator |
| Lindah Otieno | Family AIDS Care and Education Services (FACES) | Co-Investigator |
| Richard Magerenge | The Organic Health Response (OHR) | Research Coordinator |
| Gor Benard Ouma | The Organic Health Response (OHR) | Research Coordinator |
| Catie Glatz | University of Minnesota (UMN) | Research Coordinator |
| Elvin Geng, MD MPH | University of California, San Francisco (UCSF) | Study Consultant |
| Dr. CT Muga | Kenya Medical Research Institute (KEMRI) | Study Consultant |
| Dr. Norton Sang | Kenya Medical Research Institute (KEMRI) | Study Consultant |
| Daniel Zoughbie, MSc (Oxon.), Dphil | Microclinic International (MCI) | Study Consultant |

**Collaborating Institutions**

Kenya Medical Research Institute (KEMRI)

University of California, San Francisco (UCSF): [www.ucsf.edu](http://www.ucsf.edu/)

The Organic Health Response (OHR): [www.organichealthresponse.org](http://www.organichealthresponse.org/)

The Microclinic International (MCI): [www.microclinics.org](http://www.gmcp.org/)

Family AIDS Care and Education Services (FACES): [www.faces-kenya.org](http://www.faces-kenya.org/)

# Abstract / Summary

Objective: The objective of this intervention is to improve engagement and retention in clinical care for HIV-infected individuals at high risk of disengagement from medical care on Mfangano, Remba and Ringiti Islands, Kenya.

Design: Individual parallel groups randomized controlled trial.

Subjects: *Selection Criteria:* Patients in HIV care at any of eight target clinics on Mfangano, Remba and Ringiti islands who miss a clinic appointment by >3 days. *Recruitment:* During the recruitment period, individuals meeting eligibility criteria will be enrolled in the study and will receive a brief counseling session identifying barriers to care and encouraging return to clinic. Index participants meeting above eligibility criteria will be randomized to either the microclinic or control arms. Those randomized to microclinic arm will be invited to recruit members of their social support network to form a ‘microclinic’ group of 5-10 individuals and will then proceed through the intervention. Control arm index participants will not form microclinic groups during the study, but will be invited to form microclinic groups after the completion of the study.

Intervention: *Intervention HIV Curriculum*: 7 bi-weekly group sessions on HIV-related topics incuding HIV biology, antiretroviral medications and group treatment support, in addition to a facilitated HIV status disclosure session.

Measurements: *Primary outcomes*: 90 day absence from clinical care; Engagement in HIV care over 12 months, as measured by proportion of time in clinical care. *Secondary outcomes*: Time to return to care; alive and retained in care at end of study; virologic suppression at 12 months.

# Introduction/Background

The global health community has made tremendous progress in the scale-up of antiretroviral therapy (ART) over the past decade, with 9.7 million people accessing ART worldwide by the end of 2012. Importantly, there is now substantial evidence supporting anti-retroviral treatment as prevention, demonstrating that reductions in community viral load and infectivity can contribute to a decrease in new HIV infections.[^3^](#_ENREF_3)

Despite this progress, retention in lifelong HIV care and treatment remains a major challenge. One estimate indicates that fewer than one third of patients diagnosed with HIV in sub-Saharan Africa are consistently retained in care until ART eligibility.[^4^](#_ENREF_4) Countless others continue to be lost to follow-up at various stages along the continuum of HIV care.[^5-7^](#_ENREF_5) This sub-optimal provision and maintenance of treatment severely curtails the potential impact of ART within high-prevalence populations.[^1^](#_ENREF_1) If ART is to be scaled and optimally implemented, comprehensive strategies to address these gaps along the continuum of HIV care need to be developed. We argue that such strategies must recognize the central role that social environment plays in an individual’s ability to engage with HIV testing and clinical care.

Given the magnitude of the retention challenge, there is considerable interest in understanding barriers and facilitators of engagement in care over time. Numerous studies have identified HIV-related stigma as a key barrier to engagement in long-term HIV care in sub-Saharan Africa.[^8^](#_ENREF_8) In a large ethnographic study across three sub-Saharan African countries, Ware et al found that access to social capital was among the most important facilitators of good adherence to therapy.[^9^](#_ENREF_9) Investigators in that study found that patient support networks provided critical psychosocial and material resources for maintaining engagement in HIV care and adherence to therapy and, in return, expected ‘good adherence’ from the patients they were supporting. This exchange of support for positive health-seeking behavior is an important function of social networks for maintaining good health.

However, our own ethnographic field work suggests that such social capital is often difficult to access when seeking support for HIV treatment.[^10^](#_ENREF_10) In large part, this is because HIV status disclosure to members of their social networks can be a difficult process for many newly diagnosed individuals to initiate.[^11^](#_ENREF_11) Consequently, HIV-infected individuals continue to endure intense stigma in isolation across rural Africa, often forced to seek care in secret, consume antiretroviral regimens covertly, and navigate challenging healthcare and economic decisions on their own.

Social interventions to promote the exchange of social capital for positive health behaviors have been previously developed. Some ART programs encourage patients to identify a ‘treatment supporter’ – a trusted individual who can provide assistance with clinic appointments and medications, as well as psychosocial support throughout the treatment process.[^14-19^](#_ENREF_14) Patient support groups allow patient peer-groups to exchange knowledge and experiences with the management of HIV and HIV care. These interventions are both made more powerful through facilitation of HIV status disclosure to close supporters or peers.[^22^](#_ENREF_22) However, by focusing on either a single treatment supporter or a group of patient peers, these interventions may not fully utilize the pre-existing social infrastructure within which patients exist on a day-to-day basis. This infrastructure is the source of social capital identified as so crucial by Ware and colleagues and may provide critical support for maintaining HIV treatment over lifetimes. We developed an intervention targeted at patient-defined social networks, intended improve support for HIV treatment among informal social groups. We seek to evaluate this intervention’s impact on engagement in HIV care among patients on ART at a rural health facility on Mfangano Island in rural western Kenya.

These at-risk social networks represent an important yet underutilized resource in sub-Saharan Africa for optimizing HIV & AIDS treatment across the entire “continuum of care,” from diagnosis to enrollment in care, initiation of ART and long-term health maintenance. To date, strategies that specifically engage social networks in HIV care remain scarce, cumbersome to implement at scale and poorly evaluated. On Mfangano Island, a remote island in the Kenyan waters of Lake Victoria, our team has developed a social network-based intervention known as the ‘microclinic’ model. Home to nearly 20,000 residents, Mfangano has limited electricity, no paved roads and a very limited health infrastructure. Clinical facilities are limited to a single Level 3 health center and several dispensaries located throughout the island.

A microclinic is not a small medical facility, but rather a therapy management collective comprised of a small group of 5-15 neighbors, relatives, and friends. Members of microclinics are trained together as a team over three to six months to provide psychosocial, nutritional, and treatment adherence support for network members who are struggling with HIV & AIDS. Microclinic group composition is intentionally not restricted by HIV-status and the microclinic’s explicit purpose is to learn how to better improve HIV care for all members of the broader community rather than focus on a particular individual. This network-oriented approach is a novel departure from disease-oriented models, such as patient support groups, composed of group members who are exclusively HIV-infected. Our model recognizes that engagement of natural social supports within existing networks is essential for maintaining lifelong HIV treatment.

A community health worker and health educator guide Microclinics through a series of discussion sessions over a period of several months. Topics range from the biology of HIV and ART, to stigma reduction, effective communication and community outreach strategies. Following completion of these sessions microclinic groups are invited to participate in a facilitated group counseling and testing session that allows participants to voluntarily disclose their HIV status to one another – a powerful way to solidify specific group support and establish an enduring social infrastructure for both treatment and prevention of HIV.

In 2012, we completed a successful proof-of-concept pilot consisting of 47 groups composed of nearly 550 community members on Mfangano Island in Kenya’s Lake Victoria. Participation included 70% of all patients on ART within pilot villages, and over 84% of all participants disclosed their HIV status to their group at the conclusion of the program. Early results indicate that the intervention resulted in a 50% reduction in patients disengaging from care (an absolute reduction from 20% in the control group down to 10% in the intervention group. We now seek to expand the intervention across all of Mfangano Island, and to neighboring Remba and Ringiti Islands, to include 300-400 patients at eight ART clinics. Half of these patients will be randomized to form microclinic groups with their social networks and the other half will be randomized to standard clinical care. Patients randomized to control conditions will be invited to recruit members of their social network to form microclinic groups and participate in the curriculum following completion of study follow-up.

# Justification

Group social support and social network interventions have great potential to improve engagement in lifelong HIV care in a cost-effective manner, however limited evidence is available to support roll-out of interventions of this type. Social support interventions often suffer from major selection bias in most research studies, leaving program implementers and policy makers with limited evidence to support decisionmaking around implementation of these programs. Social network interventions suffer from a different challenge. Though a literature is developing around interventions to promote HIV prevention among social networks, there is little to no evidence supporting social network interventions to promote engagement and retention in HIV *treatment*. By conducting a rigorous randomized trial of a social network intervention to optimize HIV treatment, we seek to address both of these challenges simultaneously. Our non-randomized pilot study conducted from 2011-2013 demostrated feasibility and suggested that the intervention substantially improves engagement in HIV care (publication in preparation). However, further evidence from a randomized trial is needed to support implementation on a wider scale. This study will provide the necessary evidence supporting both the efficacy of this intervention and the ability to implement it in ‘real world settings’. Implementation within FACES-supported Ministry of Health clinics will also enable more effective dissemination throughout other clinics in the Ministry of Health clinic network, should the intervention prove efficacious and effective.

# Research Questions

- 1. **Research Questions**

Among HIV-infected patients enrolled in HIV care at eight clinics on Mfangano, Remba or Ringiti Islands, Kenya who have missed a HIV clinic appointment by at more than 3 days:

- - 1. Does the Microclinic social network-based HIV intervention reduce gaps in clinical HIV care over 12 months of follow-up, as compared to standard of care?
    2. Does the Microclinic social network-based HIV intervention improve the proportion of time patients spend adhering to recommended clinic appointments over 12 months of follow-up, as compared to standard of care?
    3. Does the Microclinic social network-based HIV intervention reduce time to re-engagement in clinical HIV care, as compared to standard of care?
    4. Does the Microclinic social network-based HIV intervention improve retention in HIV care at 12 months of follow-up, as compared to standard of care?
    5. Does the Microclinic social network-based HIV intervention increase the proportion of patients who are virologically suppressed at 12 months of follow-up, as compared to standard of care?
  1. **Hypotheses**
     1. Participants randomized to the microclinic HIV intervention will have a lower incidence rate of 90-day absence from clinical HIV care over 12 months post-randomization than participants randomized to standard of care.
     2. Participants randomized to the microclinic HIV intervention will spend a larger proprotion of time ‘in care’ than those randomized to standard of care. Time in care is defined as the number of days a patient spends adherent to his or her clinic visit schedule divided by the total number of days the patient is observed. See measurement section below for more detail).
     3. Participants randomized to the microclinic HIV intervention will have a higher hazard of re-engagement in clinical care than those randomized to standard of care. Re-engagement is defined as attending an HIV clinic visit and receiving the patient’s ART prescription from the clinic pharmacy.
     4. A larger proportion of participants randomized to the microclinic HIV intervention will be retained in HIV care at 12 months than those randomized to standard of care. Retention is defined as being alive and not missing a clinic appointment by 90 days or more as of study closure at 12 months post-enrollment of the last study participant.
     5. A larger proprotion of participants randomized to the microclinic HIV intervention will be virologically suppressed at 12 months of follow-up than those randomized to standard of care.

# Research Objectives

## General Objectives

### To rigorously evaluate the efficacy of the microclinic social network intervention on the ‘kinetics’ of engagement in the HIV cascade of care, as well as other clinical outcomes, on Mfangano, Ringiti and Remba Islands.

### To evaluate essential implementation science parameters of the microclinic social network intervention.

## Specific Objectives

### To understand the efficacy of the microclinic social network intervention on:

### engagement in HIV care over time among patients who are at particularly high risk for disengaging from care.

### re-engagement in care following a missed clinic appointment.

### overall retention in care after one year of follow-up

### To understand implementation parameters of the microclinic social network intervention, in particular:

### Cost-effectiveness measures of the intervention per patient retained

### Fidelity of implementation of a complex social network intervention in remote island clinic settings

### Adoption of intervention among diverse populations on three different islands

# Design and Methodology

## Study site

The study will be conducted on Mfangano, Ringiti and Remba Islands, Homa Bay County, Kenya. This study will be coordinated out of the Ekialo Kiona Center on Kitawi Beach, Mfangano Island. Index participant enrollment and data collection will occur at any of the FACES-supported Ministry of Health clinics on the three islands (Table 1). Patients who receive HIV care and treatment at any of these clinics and who miss a clinic visit by >3 days will be eligible for participation in the study. Patients residing in the Mfangano East sub-location will be excluded from participation in this study as we previously conductd a pilot study offering all patients in that geographc area the opportunity to participate in the microclinic intervention. Data will be analyzed by investigators at KEMRI, UCSF, MCI, and OHR.

| **Health Center** | **Current adult patients** | **Current adult patients on ART** |
| --- | --- | --- |
| **Mfangano Island Total** | **1,380** | **856** |
| Sena HC | 549 | 427 |
| Ugina HC | 153 | 66 |
| Wakula HC | 397 | 185 |
| Yokia HC | 192 | 131 |
| Nyakweri HC | 49 | 33 |
| Soklo HC | 40 | 14 |
| **Ringiti Island Total** | **111** | **78** |
| Ringiti HC | 111 | 78 |
| **Remba Island Total** | **234** | **147** |
| Remba HC | 234 | 147 |
|  |  |  |
| **Total patient population** | **1,725** | **1,081** |
| **Table 1.** Health center adult patient populations on Mfangano, Remba and Ringiti Islands | | |

## Study population

### Subject Inclusion Criteria

- Must be currently receiving combination antiretroviral therapy (cART) or co-trimoxozole prophylaxis from one of the eligible study clinics:
  - Is currently in care at study baseline, defined as having ≥1 medical care visit to receive medications (cART or prophylaxis) during the six months preceeding initiation of study enrollment, OR
  - Initiates pre-ART or ART care for the first time at a study clinic during the six month study recruitment period, OR
  - Transfers from a clinic outside the study area and receives pre-ART or ART care for the first time from a study clinic during the six month recruitment period
- Participants become eligible for study inclusion on the 4^th^ day following a missed clinic appointment, provided the patient has not transferred care to another clinic prior to the 4^th^ day, moved outside the study area or died. Participants who miss a study visit by >3 days but return to clinic before recruitment takes place are still eligible for study enrollment.
- Must currently reside on Mfangano, Remba or Ringiti Island.
- Must be conversant with one of the commonly spoken languages within the study area (i.e. DhoLuo, Swahili or English; prior experience indicates that this represents >99% of the adult population).
- Must be ≥18 years of age as of the date of study eligibility.

### Subject Exclusion Criteria

- Eligible participants who reside in the Mfangano East sub-location will be excluded from participation in this study. Many of these patients have already participated in the microclinic intervention as part of the Mfangano Island Healthy Network Impact Study.
- Participants planning on moving permanently out of the study area within the next six months will be excluded.
- Participants who, should they be randomized to the intervention arm, would be unwilling to participate in a microclinic will be excluded from study enrollment.

## Sampling

*Sample Size Calculation*

Sample size calculations were generated based on preliminary data from the Mfangano Island Healthy Network Impact Study (MIHNIS). The MIHNIS study was a quasi-experimental study with the intervention delivered to one community and neighboring communities acting as control groups. Additionally, the proposed clinic sites were visited in September 2014 to assess rates of missed clinic appointments to help ground sample size estimates in the realities of each individual clinic.

During the six months prior to delivery of the intervention in the MIHNIS study, 268 of 410 (65%) patients on ART at the Sena Health Center missed a clinic visit by >3 days. Of these, 238 (89%) joined the study. Among the control group in the MIHNIS study, 36 of 145 (25%) participants who missed a clinic visit by more than 3 days during the 6 months enrollment period had a 90-day clinic absence within the following 12 months. Among patients eligible for the intervention and who missed a clinic visit by >3 days during the enrollment period, 72 of 93 (77%) actually participated in the microclinic intervention. In the MIHNIS study participants in the intervention community had a more than 50% lower rate of 90-day disengagement than those in the control community, regardless of intervention uptake.

Thus, we assume an event rate of 0.25 in the control group and 0.125 in the intervention group. Setting alpha to 0.05 and beta to 0.2 and assuming an average of 2 participants per group with a coefficient of variation of 0.25, we will need 156 index participants per study arm. This sample size accounts for non-independence of outcomes introduced by the clustered nature of the intervention. Allowing for 10% participant attrition, we would need to sample 175 participants per study arm. The MIHNIS study ascertained outcomes for 98.6% of study participants, thus the allowance for 10% attrition in the proposed study is conservative.

The accessible population for this study includes all patients in HIV care and treatment, regardless of ART status, at the Sena (excluding patients residing in the East sub-location), Yokia, Ugina, Nyakweri, Wakula, Soklo, Remba and Ringiti Health Centers. Excluding patients residing in the Mfangano East sublocation from the total in Table 1, results in approximately 1500 current adult patients in these clinics. Extrapolating data from the MIHNIS study would suggest that 65% of these patients would become eligible for the Kanyakla Study during study enrollment. However, based on more recent observations at the included study clinics, we expect that only about half of the expected number of patients will become eligible. If we assume that 32% will become eligible that 75% of those who are elibigle will be willing to participate in the study, we will have an accessible population of 365 or approximately 180 per arm. Thus, because the accessible population is larger than the estimated sample size, we will extend our sample size to include up to 180 participants in each arm to allow for errors in these sample size calculations.

*Sampling Procedure*

Sampling will take place at included study clinics on a bi-weekly basis. Study staff will use information from the clinic daily activity log, master facility log and defaulter logs to identify all eligible patients who have missed clinic visits by >3 days. All eligible patients will be sampled for the study.

## Subject Recruitment and Enrollment (Figure 1)

Following sampling at the clinic, study staff will coordinate with clinic staff to obtain locator information and attempt to locate the potential participant in the community. Once identified, study staff will work with clinic staff to locate the patient to both encourage return to the clinic and invite them to join the study.

After explaining the study and answering all questions, eligible participants will be invited to enroll. Participants will be read the study consent form and will be asked to sign. If patient is illiterate, the participant will be asked to identify a witness who can co-sign the consent and help ensure that the participant fully understands all risks and benefits of the study. Following enrollment, participants will be randomized to either the intervention or control arm. Randomization will be conducted using sealed envelopes stratified by clinic site. Staff will open the next sequentially numbered envelope and will notify participants of their randomization allocation.

Participants randomized to intervention will be invited to form ‘microclinic’ groups. Microclinic formation involves recruitment of five to ten members of their social networks who they commonly rely upon for support (ie family members, friends, neighbors, co-workers, church members, etc). Study staff will assist in group recruitment and formation according to the preferences of the participant (i.e. participant will be asked whether they want to approach potential group members themselves or whether they want study staff to confidentially approach potential group members to invite them to participate). This process mirrors that used in our previous Mfangano Island Healthy Network Impact study (MIHNIS).

Participants randomized to control will be told that they are eligible to form microclinic groups following the conclusion of the study. They will be counseled on the importance of social support in their HIV care and will be clearly advised that assignment to the control group should not prevent them from discussing their HIV care with those close to them. As is true for all participants who are recruited for the study, they will be advised to return to the clinic. Beyond the counseling to return to clinical HIV care provided by study staff, standard of care for patients who have missed a clinic visit is to receive a follow-up visit from clinic staff to facilitate return to care. Study staff will collaborate with clinic staff to ensure that all study participants receive a visit from study staff and clinic staff to encourage return to care.

Orientation meetings will be conducted on a bi-monthly (every other month) basis with all newly formed microclinic groups. This meeting will include the following:

- The microclinic intervention will be explained to all prospective group members and a brief orientation to the curriculum will be provided.
- Study enrollment of social network members who were not originally enrolled through eligibility determination at the clinic. These ‘social network members’ will be consented for study participation (See Appendix I, Consent document for Microclinic Group Members), but will not be included in primary study analysis. Data from these participants will only be used for purposes of understanding the characteristics of those who were selected for microclinic participation and how their characteristics impact outcomes among index participants. For ‘social network’ participants, study participation will involve survey administration and data collection on participant HIV and clinic status in order to understand microclinic group composition. Study enrollment is voluntary and refusal will not prevent social network group members from participating in the microclinic group meetings. Aside from reasons for refusal, no data will be collected for social network participants refusing study enrollment.
- All group members will undergo individual confidential HIV testing (group members who are known to be HIV-infected will attend a counseling session but will not be tested).


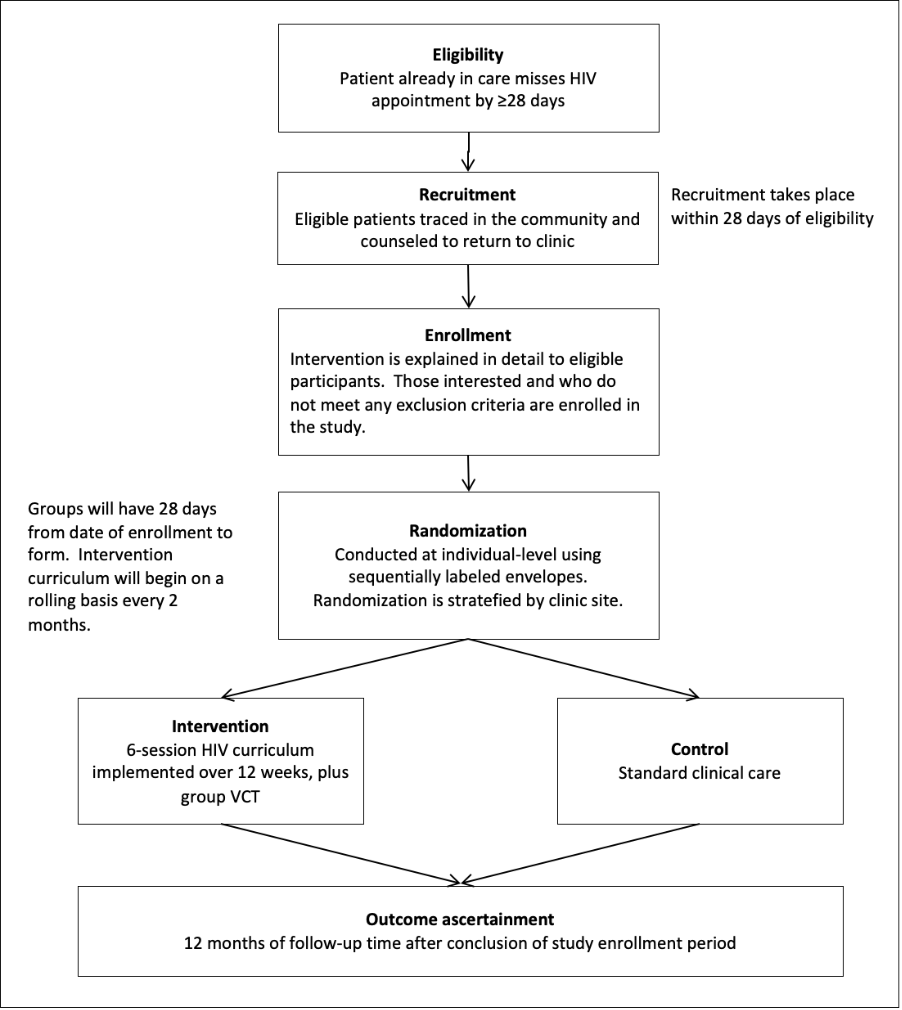


**Figure 1.** Participant flow diagram

## Community Mobilization

The objective of the study’s community mobilization strategy is to both communicate the purpose and nature of the proposed study to the community, and to also solicit community input on study activities on an ongoing basis. The following community groups are the primary target groups for this study’s mobilization activities:

1. Community Advisory Group
2. Provincial administration
3. Ministry of Health (County, Sub-County, individual clinics)
4. Local NGOs
5. Community Health units (Community Health Extension Workers, Community Health Workers)
6. Churches
7. Beach Management Unit
8. Communities within the study area

Beginning several months before commencement of participant recruitment, study staff will meet with representatives of each of the above groups in the communities included in the study to explain the nature of the study and solicit feedback. The study will also form a Community Advisory Group composed of community representatives. This group will meet monthly to hear updates on study progress and provide ongoing feedback from the community. After meeting with representatives from the above groups, mobilization will procede to community-wide mobilization. Chief’s barazas will be held in each sub-location included in the study to communicate the nature of the study to the community at large.

Given the nature of study recruitment and the phased roll-out of the study a carefully crafted message will be presented to the community regarding the way that microclinic groups are formed. Community members will be informed that we are expanding the microclinic program to all parts of Mfangano, Remba and Ringiti islands. To facilitate this expansion, we are starting with a small number of groups in the first phase in order to study how the program works at this larger scale. After completion of phase 1, those who were not able to join the program initially will then have an opportunity to join. This approach will allow for cover from stigma regarding the way that groups are initially formed, and will also help both control group index participants and other community members not feel left out of the program.

Following completion of the study and during the second phase of expansion where open microclinic enrollment is allowed (and where control group members will receive the intervention), study results will also be communicated back to participating communities. Chief’s barazas will be held to communicate findings back to the community at-large. Individual meetings with the above groups will also be held to further facilitate report-back of results.

## Procedures

### Study Design Overview

This is an individual randomized control trial evaluating the impact of the social network-based microclinic intervention on engagement and retention in HIV care over twelve months of follow up among individuals who have missed a clinic appointment by >3 days.

Study enrollment will occur over a six-month period. Upon meeting eligibility criteria, study staff will trace participants in the community to enroll them in the study. Eligibility will be determined using an Eligibility Determination Form, adapted from the FACES Loss to Follow-Up form (Appendix A). Study staff will counsel all eligible participants to return to clinic. After this counseling session, study staff will explain the purpose of the study and the nature of the intervention. After answering all participant questions, interested participants will be consented to participate (Section IX.c and Appendix H). Following study enrollment, participants will be randomized to intervention or control arm. Those randomized to intervention will be invited to recruit their social network members to form a microclinic. Study staff will provide confidential assistance to help discreetly identify and recruit family, friends, and neighbors to join the program based on previous protocols developed during the Mfangano Health Net Microclinic Pilot Program. Participants randomized to the control arm will be invited to form a microclinic group after the conclusion of the study.

Baseline measurements for index participants (i.e. participants who are eligible based on missed clinic visit) will be taken at study enrollment, with study staff administering a survey to collect data on baseline participant characteristics (Appendix C). Reasons for initial disengagement from care will also be recorded.

The microclinic intervention includes participation by members of the index patient’s social network. This may include other index patients, or community members who are HIV-infected, HIV-uninfected or of unknown HIV status. These non-index microclinic members, referred to as ‘social network’ participants, will be invited to join the study in addition to participation in a microclinic group. Microclinic participant is not contingent on study participation, however data will only be collected for microclinic group members who also consent to study participation.(Appendix I) For social network participants, study participation involves completion of a baseline survey to collect information on sociodemographic characteristics, HIV-related stigma, previous HIV testing, HIV status, and enrollment and engagement in HIV care (if HIV-infected).(Appendix D) For each microclinic group, a Microclinic Group Member Information form will also be completed to describe the nature of the realtionships between group members.(Appendix E) Only group members who consented to study participation will be included. At the time of group enrollment, all prospective social network study participants will be invited to enroll in the study. Following study enrollment, these surveys to collect information on sociodemographic characteristics and HIV-related stigma will be administered by trained study staff. Data on previous HIV testing, HIV status and enrollment and engagement in HIV care will be collected by a trained Voluntary Counseling and Testing (VCT) counselor as part of a baseline HIV counseling and testing session. Refusal to participate in the study will not preclude social network group members from participating in the microclinic group, but no study data will be collected on these participants.

A full clinic visit history will be obtained for all study participants on HIV care and treatment at any of the included study sites. Visit history data will be obtained from the FACES-MOH “Blue Cards”. For patients who made an official tranfer to another facilities within Nyanza Province, study staff will visit that facility to confirm the transfer and obtain a complete clinic visit history following the transfer. In addition to data on clinic visit attendance, patient enrollment date, ART initiation date, ART regimen, CD4 measurements, viral load measurements, pharmacy pick up, vital status and locator information will also be collected. All participants lost to follow-up from clinical care during the course of the study will be traced to ascertain vital and clinical status (i.e. disengagement from any clinical care, transfer to another facility, death while ‘in care’ or death after disengaging from care). For patients who made an unofficial transfer to another health facility in Nyanza Province, study staff will visit that facility to obtain visit history as was described for the official transfers. For patients who are disengaged from clinical care at the time of tracing, barriers to return to care will be identified and participants will be counseled to return to care. Clinic staff will be informed of any additional information gained in the field regarding the vital or clinical status of patients who were lost to follow up from clinical care.

### Intervention

Index participants who are randomized to the intervention arm will be invited to form a microclinic group. As described above, study staff will assist participants in microclinic group formation in a confidential manner. Though groups are formed from the social networks of index patients, group members will not be made aware of the specific process by which groups are formed or the identiy of the index patient or patients within any given group. This procedure protects against unintended breach of confidentiality and also allows groups to focus on the health of all members rather than specific index group members.

Following group recruitment, newly formed groups will attend an orientation meeting. This meeting will provide an overview of the microclinic intervention, as well as time for ‘social network’ participants to enroll in the study, complete baseline data collection and participate in baseline HIV voluntary counseling and testing (see enrollment procedures in Section VIII.d).

Following the orientation meeting, all microclinic groups will progress through a series of six microclinic sessions. (Appendix J) Sessions will take place every two weeks, will be led by trained community health workers and will take place at a time and location of the group’s choosing. Study staff will provide training for the community health worker facilitators and will supervise facilitation to ensure that the intervention is delivered as intended. Session topics for the seven sessions are as follows:

1. Overview of HIV biology and Scope of HIV on Lake Victoria
2. HIV medication overview – how medications work and why they are important
3. Antiretroviral therapy and herbal medicine
4. Destigmatization and group social support
5. Being an active patient
6. Confidentiality

[Group disclosure]

1. Post-group disclosure debriefing and the way forward

Following completion of the sixth group session, microclinic group members will be offered the opportunity to participate in Voluntary Counseling and Testing and group HIV status disclosure to other microclinic group members. Participation will be strictly voluntary and the sixth microclinic session will extensively cover the topic of group confidentiality and the importance of not pressuring or stigmatizing group members who do not want to participate in the process.

### Measurements

The predictor of interest is the baseline treatment assignment, as determined by randomization. All analysis will be conducted using an intention-to-treat analysis.

Covariates of interest will be collected through participant surveys at baseline and study closure, as well as routine clinic data collected at the participant’s original clinic and any other clinics from which he or she receives HIV care during the course of the study. Baseline surveys (Appendix C) will collect data related to the following:

1. Demographic characteristics
2. Contact and locator information
3. HIV-related stigma[^23^](#_ENREF_23)
4. HIV status disclosure
5. Social support
6. Clinic data (date first HIV positive, date of enrollment)
7. Reason for missed clinic appointment, including name of other facilities where care was received

At the end-of-study survey (Appendix G), additional data will be collected related to possible adverse events that may or may not be related to the intervention. In addition to specific questions in the survey, an open-ended question will be used to assess other adverse experiences related to issues such as stigma, unintended disclosure or poor treatment by health center staff. Participants will be asked whether they attribute any or all of these experiences to participation in the study. Additionally, participants in the intervention arm will specifically be asked about adverse experiences related to group status disclosure, again using both a checklist and an open-ended question. At the end-of-study survey, patients will be asked if they are currently in care and, if so, which clinic. Reasons for transfer or disengagement from the original clinic will be assessed. For patients who were lost-to-follow-up (missed a clinic visit by ≥90 days and did not return to clinic) or who transferred out from their original clinic, a patient tracing survey will also be conducted. (Appendix F) See description of clinic data capture below regarding determination of patient’s visit history and transfer status.

In addition to survey data, clinical data will also be collected for all study participants. Chart review will be conducted at participant’s clinic of origin to collect data on baseline disease status, and clinical and laboratory data over the course of the study (CD4 count, WHO stage, viral load). Other clinical data will include date of first HIV test, date of clinic enrollment, date of ART initiation (if applicable), complete visit history, patient tracing visits conducted by clinic staff, patient death and patient transfer. For patients who transfer to other sites, study staff will visit that site to confirm that transfer occurred and obtain the above clinic data available for the patient after the transfer (either through physical chart review or the OpenMRS electronic medical record). Data obtained from clinics will be limited to data that was collected during routine patient care.

Qualitative Data Collection

Focus Group Discussions:

Twelve (12) focus group discussions will be conducted at the conclusion of the intervention phase of the study. It will be reiterated to all participants that focus group participation is completely voluntary and that refusal to participate as well as any issues discussed within the focus group will have no effect whatsoever on relationships with FACES and OHR. All FG participants will complete a separate Qualitative Consent Form (Appendix K). Each Focus group will follow the discussion protocol outlined in Appendix L – Focus Group Discussion Guide.

- - - Eight (8) focus group discussions with study participants (5-15 people each). A convienience sample of participants will be identified and selected by research staff during enrollment, and follow-up, based on availability and interest in participating in Focus Group discussions.
    - Two (2) focus group discussions with CHW Facilitators who lead and conduct the micro-clinic training program.
    - Two (2) focus group discussions with the FACES/OHR staff implementing the Microclinic intervention.

Discussions will investigate issues of care and treatment utilization and HAART adherence, social support, local HIV attitudes, microclinic participation, and community mobilization strategies. The target size of the discussions will be 8-12 participants. Group discussions will be facilitated by a trained research team member and a note-taker/translator. The discussions will be audio-taped and transcribed. Finally, the collection of qualitative data may extend beyond these focus groups. If emerging data points to particular concerns or needs of individuals in the focus group, then we will conduct in-depth semi-structured interviews with particular focus group participants or schedule an additional focus group discussion. All data from FG discussions will be de-identified be kept anonymously to protect participant confidentiality.

Semi-Structured Interviews

A convenience sample of between 25 and 50 study participants to participate in informal, semi-structured interviews with trained research staff. Research staff will recruit an equal number of participants from intervention and non-intervention arms. Semi-structured interviews will be scheduled in a private location at the convenience of study participants, either at the clinic, community center or home. All interviews will be conducted by trained local research staff in Luo, Swahili or English, per subject preference, using an open-ended interview format. Participants will be given the opportunity to comment on three major topics 1) their experience regarding engagement with HIV care and treatment on Mfangano Island, 2) challenges faced, and 3) opportunities for improvement. Study subjects who participated in the microclinic intervention will be given an opportunity to provide critical, anonymous feedback. Notes and audio recordings will be maintained and secured at the local research facility. All data from semi-structured interviews will be de-identified be kept anonymously to protect participant confidentiality. Discussion guides for these semi-structured interviews can be found in Appendix M. Participants in semi-structured interviews will complete a separate informed consent document prior to initiating the interview (Appendix K).

Participant Observation

Trained social scientists from the University of California in San Francisco, along with qualified local research staff will also participate in MC training workshops throughout the island, living within the Mfangano Island community for the duration of the study. These research staff will work with FACES and OHR staff to implement the program and gather anonymous observations and feedback from participants and community members. Formal participant observations will be documented to aid in the interpretation and discussion of study results at the conclusion of the study.

### Data Verification and Security

All data will be collected by study staff trained in Good Clinical Practice procedures and Human Subjects Protection. Training will be conducted independently by an on-site co-investigator and through internet-based courses with the NIDA Clinical Trials Network and the National Institutes of Health. Training will be renewed on an annual basis during the duration of the trial.

All personal identifying information (name, date of birth, phone number, locator information, medical record number) will be collected on a hard copy form that will be separate from the rest of the survey data that will be collected on elecronic tablets. Identifying information will be linked to electronically-captured study data only through a unique identifier that is assigned by the study and carries no meaning outside the study. Indentifying information will be stored in separate locked file cabinets in the Ekialo Kiona Center Research Department. De-identified electronic data will be collected using password protected tablets and will be encrypted and uploaded to a secure server. Tablets will be stored in the study office, which will remain locked any time an authorized study staff member is not present. Furthermore, the Ekialo Kiona center is under survelliance 24 hours per day, 7 days per week by a trained security guard to ensure that there is no unauthorized access to the facilities or to confidential research documents.

Data collected at the health facilities will be entered into a password-protected Microsoft Access database on a password protected laptop. Where possible, clinical data will be cross-checked against electronic medical record data obtained from the FACES OpenMRS database.

### Adverse Events

All study participants in both study arms will be asked to complete an end-of-study survey. This survey will include an open-ended questions about any adverse events experienced over the course of thes study (Appendix G). Risk of any adverse event will be compared between index participants in the two study arms, and risk of adverse events will be described among ‘social network’ members who participated in the microclinic intervention. All participants reporting an adverse event will be offered counseling by a trained VCT counselor and/or a staff member at the participant’s preferred health facility.

In addition to self-reported adverse events, clinic staff will also review participant medical records on a monthly basis. Appendix N outlines study procedures for reporting adverse clinical events to the KEMRI Ethical Review Committee.

## Data Analysis

Primary analyses will be conducted using only the index study participants (i.e. participants who are HIV-infected and who missed a clinic visit by >3 days to become eligible for study participation).

Hypothesis 1

We will compare the indicence of 90-day absence from clinical HIV care between study arms using a Cox proportional hazards model. Additionally, we will depict the cumulative incidence of 90-day absence using the Cumulative Incidence Function, with death as a competing event.[^24^](#_ENREF_24) Primary analysis will be unadjusted, with standard errors adjusted for clustering of participants who are in the same microclinic group. Validity of the proportional hazards assumption will be assessed graphically and through formal testing using Schoenfeld residuals.

Hypothesis 2

‘Time in care’ will be calculated for each participant by subtracting the total days ‘out of care’ from the total length of follow-up and divided by the total length of follow-up. This will constitute a proportion depicting the proportion of time a given participant has spent adhering to his or her clinic appointment schedule. The number of days ‘out of care’ will be calculated by summing the total number of days between missed appointments and next clinic visits for each participant over the course of follow-up. Analysis will be conducted using linear regression to compare the mean proportion of ‘time in care’ between study arms. Standard errors will be adjusted for clustering, as in Hypothesis 1. Bootstrapping with cluster re-sampling will be conducted to address potential non-normality of residuals. Primary analysis will be conducted using unweighted time in care proportions for all participants, with sensitivity analysis conducted where each participant is weighted according to the length of follow-up.

Hypothesis 3

Because all eligible study participants will have recently missed an appointment, we are interested in the rate of reconnection to care. Reconection to care will be defined as return to any HIV clinic and receiving a refill of that patient’s ART or co-trimoxazole prophylaxis. We will depict time to reconnection to care by study arm using the Cumulative Incidence function, with death as a competing event.[^24^](#_ENREF_24) We will formally test the differece in the rate of reconnection to care using a Cox proportional hazards model. Standard errors will be adjusted for clustering, as in Hypothesis 1. Validity of the proportional hazards assumption will be assessed graphically and through formal testing using Schoenfeld residuals.

Hypothesis 4

The proportion of paritipcants alive and retained in care at the end of 12 months of follow-up will be compared between study arms using simple chi-squared analysis. Retention is defined as being alive and not missing a clinic appointment by 90 days or more as of study closure at 12 months post- study enrollment.

Hypothesis 5

The proportion of participants who are alive and virally suppressed at the end of the study will be compared between study arms using chi-squared testing. Viral load testing conducted within 180 days of the 12-month timepoint post study enrollment will be used for analysis. As above, viral loads utilized for analysis will be collected during routine patient care. Primary analysis will be conducted on an intention to treat basis, with missing values assumed to be virologically non-suppressed. Sensitivity analysis will be conducted using completer analysis, where only participants with measured viral loads will be included. In this sensitivity analysis, participants who died or who disengaged from care will be included as virologically non-suppressed.

## Project Sustainability

This project is an extension of the Mfangano Island Healthy Networks Impact Study, and thus directly a part of the sustainability of that project. As a certified Community Based Organization that has been working on Mfangano Island for the past six years, the Organic Health Response (OHR) has a longstanding commitment to the communities where this project will be implemented. Should the study demonstrate efficacy, OHR is committed to maintaining an active microclinic program on Mfangano, Remba and Ringiti islands. If efficacious, OHR plans to work with other regional and international partners to scale this intervention throughout Nyanza Province as a cost-effective and highly efficacious method for employing social networks to help maintain high levels of retention in HIV care throughout the region.

# Ethical Considerations

- 1. **Declaration of Helsinki**

The Investigators will ensure that this study is conducted in conformity to the Declaration of Helsinki and the International Conference on Harmonization.

- 1. **Institutional Review Board**

This protocol and the informed consent forms will be reviewed and approved by the ethical review bodies responsible for oversight of research conducted by the two participating institutions, KEMRI and UCSF. The IRBs in both the United States and in Kenya hold a current U.S. Federal-Wide Assurance issued by the Office for Human Research Protections (OHRP).

- 1. **Informed Consent Process**

After receiving Institutional Review Board (IRB) approval from KEMRI and UCSF and prior to the start of all study procedures, written informed consent will be obtained individually from each study participant in a private location. A written informed consent form will be provided to potential subjects in the language of his or her choice (English, Swahili or Luo; Appendex H), and its contents will be carefully and fully explained. During the written and verbal consent process, study staff will explain the purpose of the study, any anticipated consequences of the research, the anticipated uses of the data, possible benefits of the study and possible harm or discomfort that might affect participants, and the option to end the interview or study involvement at any time. In addition, it will explain the secure, confidential storage of the data, and explain that participants will receive access to the aggregate final results of the study. All participants will be over 18 years old and will have the capacity to consent for themselves. In the event that a potential participant is illiterate, the participant will mark his/her thumbprint and a witness will be asked to preside over the consenting process and co-sign the consent form.

- 1. **Subject Confidentiality**

Participant confidentiality is of paramount importance to the study investigators and staff and extreme caution will be taken to avoid any breaches of confidentiality. Study staff will be thoroughly trained in research practices and confidentiality, as well as proper handing of study documents. Data collected from participants will be de-identified at the time of data collection and will only be linked to identifying information through the unique study ID. As discussed above, identifying information and study datasheets will be kept in separate locked file cabinets located in a locked room dedicated only for research activities.

Participants will be advised that, by nature, group interventions such as the microclinic intervention have an inherant risk that confidentiality will be breached by a fellow group member. To minimize this risk, a substantial portion of the microclinic curriculum focuses on the importance of creating a safe space for all group members, emphasizing that all discussion that occurs within the setting of the group must not be shared with those outside the group.

- 1. **Study Discontinuation**

Inadaquate participant enrollment is the only reason why the study will be prematurely discontinued. Inadequate enrollment is defined as <50 participants enrolled in the study at the end of the 6 month enrollment period.

- 1. **Alternatives to Study Participation**

The alternative to study participation is not to participate. This will not in any way affect potential patients’ access to benefits from FACES or OHR services.

- 1. **Risks and Benefits of Study**

1. **Potential risks and discomforts**

The main risk of this study is breach of confidentiality or harm to reputation resulting from unintended HIV status disclosure.

1. **Steps being taken to minimize potential risks or discomforts**

Both study staff and microclinic group members will be trained on the importance of maintaining confidentiality. Additionally, all study documents will be de-identified at the time of data collection and stored separately from identifying information.

1. **Potential direct benefits to study subjects**

There is no direct benefit to study subjects. The intervention may provide benefit, but as there is currently equipoise regarding the efficacy of this intervention, this cannot be considered a benefit.

1. **Potential benefits to society**

This study evaluates a novel, sustainable, community-based intervention for improving clinical HIV care. If the intervention proves successful, the intervention will be expanded to include all patients meeting criteria at the study sites. If adopted by other clinical sites, it could also have broader impact on HIV service delivery throughout the region.

Furthermore, this intervention evaluates a method for improving clinical care that could be applied to other health problems beyond HIV. Our research group has evaluated this model for diabetes care in other parts of the world and, similarly, is interested in expanding the scope of its use for other chronic illnesses or health challenges that involve a behavioral component in their treatment.

1. **Risk/Benefit analysis**

The risks of this study are minimal, especially after careful attention to minimizing such risks taken by the study team. Conversely, the potential benefits to society are great. Should this intervention prove effective, it will pave the way for broad implementation across Mfangano, Ringiti and Remba islands, at minimum. If adopted by study team partners, it could also impact the way that HIV patients are supported throughout Nyanza. Our assessment is that the benefits outweigh the risks in this case.

1. **Payments to subjects**

Participants who have missed clinic visis at either baseline or end-of-study visits will receive 100 shillings to compensate participants for time spent responding to survey questions. Investigators have choosen to offer this small monitary reimbursement because it is consistent with what other studies in the region are also providing to participants (i.e. the SEARCH study). Subjects who participate in a microclinic group will receive refreshments at group meetings (tea and mandazi) as both an incentive to attend group meetings and as a symbol of group solidarity.

1. **Cost to subjects**

The only cost to subjects is their time. The pre- and post-intervention surveys will take approximately 30 minutes each.

# Data Management

- 1. **Data Collection and Storage**

Survey data will be collected by bilingual OHR research staff and recorded electronic tablets using Open Data Kit. Data entered into tablets will not contain any identifying information and tablets will be password protected. The limited personal identifying information collected from participants (name, date of birth, and a unique study identification number) will be written on a paper datasheet, which will be linked to data in the tablet only through the use of the unique study ID number. Identifier pages and tablets will be stored in separate locked containers immediately following data collection.

Data collected through the tablets will be encrypted and uploaded to a secure server. Identifying information will be entered into a separate Microsoft Access database and this ‘link log’ will be maintained on a password protected computer located in a locked room at the study site. Data collected from clinics included in the study will be entered into a password protected Microsoft Access database located on a dedicated, password-protected study computer. The study database will be backed up on the secure UCSF MyResearch portal.

- 1. **Data Analysis**

Study investigators will be responsible for all data analysis. Analysis will be conducted in Stata and will consist of intent-to-treat comparison of study outcomes between study arms.

# Time Frame/Duration of the Project

The total duration of this project will be 2.5 years (30 months):

- Months 1-2: Community mobilization
- Months 3-8: Participant recruitment and enrollment
- Months 3-11: 3-month intervention delivered to newly enrolled participants on a rolling basis
- Months 9-20: Follow-up phase: participant engagement in care assessed for at least 12 months post-enrollment
- Months 21-24: Data cleaning and analysis (onsite and offsite data compilation and regression).
- Months 24-30: Manuscript preparation and reporting of results back to community.

# Expected Application of the Results

All study results will be reported, with approval of KEMRI, in peer-reviewed publications. Study investigators will also report results in other relevant forums within Kenya and elsewhere (i.e. University of Nairobi STD/AIDS Collaborative Group Annual Meeting). Finally, if intervention proves successful, investigators plan to pursue funding for, at minimum, local implementation and hopefully broader implementation of the intervention throughout Homa Bay County or Nyanza Province.

# References

1. WHO. *Global Update on HIV Treatment 2013: Results, Impact and Opportunities.* Geneva, Switzerland: World Health Organization;2013.

2. Hirnschall G, Schwartländer B. Treatment 2.0: catalysing the next phase of scale-up. *Lancet.* 2011;378:209-211.

3. Cohen MS, Chen YQ, McCauley M, et al. Prevention of HIV-1 infection with early antiretroviral therapy. *N Engl J Med.* Aug 11 2011;365(6):493-505.

4. Rosen S, Fox MP. Retention in HIV care between testing and treatment in sub-Saharan Africa: a systematic review. *PLoS medicine.* 2011;8:e1001056.

5. Fox MP, Rosen S. Patient retention in antiretroviral therapy programs up to three years on treatment in sub-Saharan Africa, 2007-2009: systematic review. *Trop Med Int Health.* 2010;15 Suppl 1:1-15.

6. Geng EH, Bwana MB, Muyindike W, et al. Failure to Initiate Antiretroviral Therapy, Loss to Follow-up and Mortality among HIV-infected Patients during the Pre-ART period in Uganda. *J Acquir Immune Defic Syndr.* Feb 20 2013.

7. Geng EH, Glidden DV, Bwana MB, et al. Retention in care and connection to care among HIV-infected patients on antiretroviral therapy in Africa: estimation via a sampling-based approach. *PloS one.* 2011;6:e21797.

8. Mbonu NC, van den Borne B, De Vries NK. Stigma of People with HIV/AIDS in Sub-Saharan Africa: A Literature Review. *Journal of tropical medicine.* 2009;2009:145891.

9. Ware NC, Idoko J, Kaaya S, et al. Explaining adherence success in sub-Saharan Africa: an ethnographic study. *PLoS Med.* Jan 27 2009;6(1):e11.

10. Salmen C. *Towards an Anthropology of Organic Health: The Relational Fields of HIV/AIDS among the Suba of Lake Victoria*: Institute of Social and Cultural Anthropology, Oxford University; 2009.

11. Greeff M, Phetlhu R, Makoae LN, et al. Disclosure of HIV status: experiences and perceptions of persons living with HIV/AIDS and nurses involved in their care in Africa. *Qualitative health research.* Mar 2008;18(3):311-324.

12. Simbayi LC, Kalichman S, Strebel A, Cloete A, Henda N, Mqeketo A. Internalized stigma, discrimination, and depression among men and women living with HIV/AIDS in Cape Town, South Africa. *Soc Sci Med.* May 2007;64(9):1823-1831.

13. Turan JM, Hatcher AH, Medema-Wijnveen J, et al. The role of HIV-related stigma in utilization of skilled childbirth services in rural Kenya: a prospective mixed-methods study. *PLoS Med.* 2012;9(8):e1001295.

14. Stubbs BA, Micek MA, Pfeiffer JT, Montoya P, Gloyd S. Treatment partners and adherence to HAART in Central Mozambique. *AIDS Care.* 2009;21:1412-1419.

15. Idoko JA, Agbaji O, Agaba P, et al. Direct observation therapy-highly active antiretroviral therapy in a resource-limited setting: the use of community treatment support can be effective. *International journal of STD & AIDS.* Nov 2007;18(11):760-763.

16. Fatti G, Meintjes G, Shea J, Eley B, Grimwood A. Improved survival and antiretroviral treatment outcomes in adults receiving community-based adherence support: 5-year results from a multicentre cohort study in South Africa. *J Acquir Immune Defic Syndr.* Dec 1 2012;61(4):e50-58.

17. Nachega JB, Chaisson RE, Goliath R, et al. Randomized controlled trial of trained patient-nominated treatment supporters providing partial directly observed antiretroviral therapy. *AIDS.* Jun 1 2010;24(9):1273-1280.

18. Pearson CR, Micek MA, Simoni JM, et al. Randomized control trial of peer-delivered, modified directly observed therapy for HAART in Mozambique. *J Acquir Immune Defic Syndr.* Oct 1 2007;46(2):238-244.

19. Taiwo BO, Idoko JA, Welty LJ, et al. Assessing the viorologic and adherence benefits of patient-selected HIV treatment partners in a resource-limited setting. *J Acquir Immune Defic Syndr.* May 1 2010;54(1):85-92.

20. Luque-Fernandez MA, Van Cutsem G, Goemaere E, et al. Effectiveness of patient adherence groups as a model of care for stable patients on antiretroviral therapy in Khayelitsha, Cape Town, South Africa. *PLoS One.* 2013;8(2):e56088.

21. Wouters E, Van Damme W, Van Loon F, van Rensburg D, Meulemans H. Public-sector ART in the Free State Province, South Africa: community support as an important determinant of outcome. *Soc Sci Med.* Oct 2009;69(8):1177-1185.

22. O'Laughlin KN, Wyatt MA, Kaaya S, Bangsberg DR, Ware NC. How treatment partners help: social analysis of an African adherence support intervention. *AIDS Behav.* Jul 2012;16(5):1308-1315.

23. Visser MJ, Kershaw T, Makin JD, Forsyth BW. Development of parallel scales to measure HIV-related stigma. *AIDS Behav.* Sep 2008;12(5):759-771.

24. Satagopan JM, Ben-Porat L, Berwick M, Robson M, Kutler D, Auerbach AD. A note on competing risks in survival data analysis. *British journal of cancer.* Oct 4 2004;91(7):1229-1235.

# Budget

| **Category** | **Amount** |
| --- | --- |
| Personnel, salaries and benefits | $51,750 |
| Intervention materials and community health worker training (food, supplies, stipends) | $29,018 |
| Equipment | $2,000 |
| Supplies | $3,625 |
| Local transportation, vehicle maintence | $9,250 |
| Operating expenses | $13,805 |
| Contingency funds (15%) | $16,417 |
| **Total** | **$125,865** |
| Total (KES, 86.5KES to 1USD as of 37Mar2014) | 10,887,290 |

**Budget Justification**

This budget covers study costs estimated through the duration of the study and are based on current salaries and expenses for operation of the Ekialo Kiona Center Research Department, as well as both anticipated inflation, raises and unanticipated events outside the control of the research department or the study. Based on longstanding experience working on Mfangano, our team has established an efficient system that enables us to keep costs low. All salary expendatures will be spent on staff working directly within the study area and, to the extent possible, new hires will be from the local region.

# Study Team Roles

| **Elizabeth Bukusi, MB.ChB., M. Med, MPH, PhD, PGD (Research ethics):** Dr. Bukusi is the KEMRI PI for the investigation. Her role is to provide guidance and oversight of the research protocol development, study implementation, compliance with Good Clinical Practice and dissemination of research findings. |
| --- |
| **Craig Cohen, MD, MPH:** Dr. Cohen is Co-Investigator on the project, and will be advising on protocol development, study implementation, compliance with Good Clinical Practice and dissemination of research findings. He is also the director of Family AIDS Care and Education Services (FACES) which supports HIV care and treatment on the island. |
| **Matt Hickey, MD candidate, Certificate in Advanced Training in Clinical Research:** Mr. Hickey is a Co-investigator for the study. He is a medical student at the University of California in San Francisco and the Co-Director of Research at Organic Health Response. He brings to the team extensive experience working on Mfangano Island, through the Mfangano Health Baseline Study, the Mfangano Island Healthy Networks Impact Study and the Healthy Networks Water Treatment Study. Matt will lead the team in study design, implementation, evaluation and dissemination. He will additionally provide overall coordination of study activities, ensuring consistent engagement of all investigators in study decisions. |
| **Charles Salmen, MPhil (Oxon.), MD:** Dr. Salmen is Co-Investigator on the project, bringing to the project extensive anthropology fieldwork on Mfangano Island and experience developing and evaluating integrated HIV prevention projects that combine medical, behavioral, economic, and environmental sustainability strategies. Charles will provide oversight and technical expertise with study design, implementation, evaluation and dissemination. He graduated from the University of California, San Francisco School of Medicine in May 2014 and is currently a Family Medicine Resident at the University of Minnesota. Charles is also the US Director of the Organic Health Response. |
| **Dan Omollo, BSc, MPH candidate:** Mr. Omollo is a study Co-Investigator and the Research Department Manager at the Organic Health Response and an MPH candidate at Maseno University in Homa Bay. He brings to the team extensive experience with research study implementation on Mfangano and will lead field implementation of the study. In addition to field implementation and evaluation, Dan will also contribute extensively to study design, data analysis and dissemination of findings. |
| **Katie Fiorella, MPH, PhD candidate:** Ms. Fiorella is a study Co-Investigator and a Co-Director of Research at Organic Health Response. She brings to the study team extensive experience working on Mfangano Island to implement multiple longitudnal research studies, including the Research on Environmental and Community Health (RECH) study. She also contributes expertise in qualitative research methodology. Katie will contribute to study design, research department oversight, study implementation, data analysis and dissemination of findings. |
| **Betty Njoroge,** **MB.ChB., MPH:** Dr. Njoroge is a study Co-Investigator and a research scientist at KEMRI and will assist with IRB protocol preparation, study implementation, data analysis and interpretation, and dissemination of results. As a clinician and researcher, Dr. Njoroge will also provide important expertise in issues related to retention in care and HIV-related stigma, and will also assist in building collaboration between this study and KEMRI. |
| **Lindah Otieno, MPH Candidate:** Ms. Otieno is a study Co-Investigator and the Mbita District Coordinator for FACES. She brings to the team longstanding expertise in clinical HIV care in the Lake Victoria region and will guide the design and implementation of the study to ensure that it is relevant to clinical realities in the rural health centers where the study will take place. Her role involves contribution to study design, guidance of study activities at clinical sites, analysis and interpretation of study results and dissemination of study findings. |
| **Richard Magerenge:** Mr. Magerenge is the Executive Director of the Ekialo Kiona Center and will aid the study team in coordination of voluntary counseling and testing activities. He will lend his local expertise and knowledge of community issues in the villages of Mfangano East Sub-location, and his expertise as a trained VCT counselor. |
| **Gor Benard Ouma:** Mr. Ouma will serve as a study coordinator and will supervise implementation of all community-based study activities. He brings to the team extensive experience designing and implementing multiple longitudonal research studies on Mfangano and is an expert community mobilizer, educator and communicator. |
| **Catie Glatz, MD Candidate:** Ms. Glatz will serve as a study coordinator. She will be in-residence at the Ekialo Kiona Center during study implementation and will be responsible for ensuring that study protocol approvals and study Standard Operating Procedure documents are up to date and properly implemented at the study site. |
| **Elvin Geng, MD, MPH:** Dr. Geng will serve as a study consultant and will provide technical expertise on study methodology, retention and engagement in care measurement and implementation science. |
| **CT Muga, PhD:** Dr. Muga is a social scientist at KEMRI and will serve as a study consultant. Dr. Muga has extensive experience with social science, community engagement and qualitative research and will advise the study team on these areas. |
| **Norton Sang:** Mr. Sang will serve as a study consultant and will assist with study implementation and dissemination of results. He has extensive experience implementing large, complex studies in the Lake Victoria region and will be an important advisor for difficult study issues that arise. Mr. Sang will also provide important local expertise and assist in building collaboration between this study and KEMRI. |
| **Daniel Zoughbie, MSc (Oxon.), DPhil:** Dr. Zoughbie will be advising on research design and data analysis. He is also the Founder and CEO of the Global Micro-Clinic Project, which has led the development of the Micro-Clinic Pilot Program. |

# List of Appendices

1. Eligibility Determination Form
2. Participant Locator Information Form
3. Baseline Participant Survey Form
4. Microclinic Group Member Survey Form
5. Microclinic Group Information
6. Loss To Follow-Up Tracing Form
7. End of Study Participant Survey Form
8. Index Participant Consent Document
9. Microclinic Group Member Consent Document
10. Microclinic Curriculum Overview
11. Qualitative Consent Document
12. Focus Group Discussion Guide
13. Semi-Structured Interview Guide
14. Severe Adverse Event Reporting

# English Appendices

| 21-APR-2014 | | | **APPENDIX A. ELIGIBILITY DETERMINATION** | | | | | | | | | | | | | | | | Reviewer’s initials and date of review: | | |  | | | | | | | | | | |
| --- | --- | --- | --- | --- | --- | --- | --- | --- | --- | --- | --- | --- | --- | --- | --- | --- | --- | --- | --- | --- | --- | --- | --- | --- | --- | --- | --- | --- | --- | --- | --- | --- |
| **SECTION A: ADMINISTRATIVE** | | | | | | | | | | | | | | | | | | |  |  |  | - - | | | | | | | | | | |
|  |  |  |  |  |  |  |  |  |  |  |  |  |  |  |  |  |  |  |  |  |  | DAY(*dd*) | | | | MONTH (*mmm*) | | | | | | YEAR (*yy*) |
| A1. Patient name: | | |  | | | | | | | | | | | | A2. Patient age | |  | | | | | A3. Patient gender | | | | | | ⭘ Male  ⭘ Female | | | | |
| A4. Clinic name: | | | 1 ⭘ Sena  _2_ ⭘ Yokia  _3_ ⭘ Nyakweri  _4_ ⭘ Wakula  _5_ ⭘ Ugina | | | | | | 6 ⭘ Soklo  _7_ ⭘ Takawiri  _8_ ⭘ Ringiti  _9_ ⭘ Remba  _10_⭘ Other, specify  __________________ | | | | | A5. Patient clinic number: | | | | | A6. Date of initial enrollment in clinic | | | | | | | | | | | | | |
|  |  |  |  |  |  |  |  |  |  |  |  |  |  |  | | | | | - -  DAY(*dd*) MONTH (*mmm*) YEAR (*yy*) | | | | | | | | | | | | | |
| A6a. Date of ART initiation | | | | | - - | | | | | | | | | | A7. Date last seen in clinic: | | | | | - - | | | | | | | | | | | | |
|  |  |  |  |  | DAY(*dd*) | | | | | MONTH (*mmm*) | | YEAR (*yy*) | | |  |  |  |  |  | DAY(*dd*) | | | MONTH (*mmm*) | | | | YEAR (*yy*) | | | | | |
| A8. Staff initials: | |  | | | | | A9. First date of tracking: | | | | | | - - | | | | | | | A10. ART status of patient: | | | Not yet started ART  Has started ART | | | | | | | | | |
|  |  |  |  |  |  |  |  |  |  |  |  |  | DAY(*dd*) | | | MONTH (*mmm*) | | YEAR (*yy*) | |  |  |  |  |  |  |  |  |  |  |  |  |  |
| A11. Source of information | | ⭘ Communicated with patient 🡪 go to **SECTION B**  ⭘ Communicated with informant(s) who knows the patient and not directly with patient 🡪 go to **SECTION C**  ⭘ Attempted but unable to communicate with patient or informant 🡪 go to **SECTION D**  ⭘ Tracking not attempted 🡪 go to **A11a** | | | | | | | | | | | | | | | | | | | | | | | | | | | | | | |
| A11a. Why was tracking not attempted? | | ⭘ Contact information illegible  🡪 **END**  ⭘ Contact information missing  🡪 **END**  ⭘ Chart missing 🡪 **END**  ⭘ Patient permanently moved outside study area 🡪 **END** | | | | | | | | | | | ⭘ Location listed too general to make tracking possible  🡪 **END**  ⭘ Chart reviewed and patient not lost to follow up  **🡪 go to A11b**  ⭘ Other, specify:  🡪 **END** | | | | →A11b. What is patient outcome? | | | | ⭘ Has recent visit – doesn’t  meet definition of lost-to-follow-up  ⭘ Transferred to clinic within study area  ⭘ Transferred to clinic outside study area  ⭘ Dead  Date:- - | | | | | | | | | | | |
| A12. Source of locator information | | | | ⭘ Paper file ⭘ Electronic database  ⭘ No locator information in paper or electronic database ⭘ Other, specify:…………………………… | | | | | | | | | | | | | | | | | | | | | | | | | | | | |
| **SECTION B: TRACKER COMMUNICATES WITH PATIENT** | | | | | | | | | | | | | | | | | | | | | | | | | | | | | | | | |
| B1. Interview type. *Mark all that apply:* | | | | | | | | in-person phone | | | | | | | | B2. Interview date. | | | | | | - - | | | | | | | | | | |
|  |  |  |  |  |  |  |  |  |  |  |  |  |  |  |  |  |  |  |  |  |  | DAY(*dd*) | | | MONTH (*mmm*) | | | | | YEAR (*yy*) | | |
| B3. Confirm identity of patient using as many identifiers as possible. *Mark all that apply:* | | | | | | | | | | | | | | | | | | | | | | | | | | | | | | | | |
| 🞎 Name 🞎 Age 🞎 Sex 🞎 Height 🞎 Location or residence 🞎 Occupation 🞎 Clinic number or medical papers | | | | | | | | | | | | | | | | | | | | | | | | | | | | | | | | |
| B4. Have you seen a doctor or nurse for the care or treatment of HIV within the last four weeks? | | | | | | | | | | | | | | | | | | | | | | | | | | | | | | | | |
| ⭘Yes | →B4a. Where?  *Mark all that apply.* | | | | | 🞎 Original clinic  **→ Question patient about missed visit**  🞎 Different clinic  🞎 Other, specify: | | | | | | | →B4b. Name of clinic or other: | | | →B4c.  When did you first make a visit to **any** new clinic? | | | | | | - - | | | | | | | | | | |
|  |  |  |  |  |  |  |  |  |  |  |  |  |  |  |  |  |  |  |  |  |  |  |  |  |  |  |  |  |  |  |  |  |
|  |  |  |  |  |  |  |  |  |  |  |  |  |  |  |  |  |  |  |  |  |  | DAY  *dd*) | MONTH (*mmm*) | | | | | | | | YEAR(*yy*) | |
|  | →B4d. Why are you going to [new clinic] instead of your previous one? *Answer both coded and descriptive responses* | | | | | Coded responses: *Mark all that apply* | | | | | | | | | | | | | | | | | | | | | | | | | | |
|  |  |  |  |  |  | **Access to Care**  🞎 I moved - now transportation is easier or cheaper  to new clinic.  🞎 I have not moved, but transportation is easier or  cheaper to new clinic.  🞎 I spend less money at new clinic.  🞎 I spend less time at new clinic.  🞎 Starting ART is easier new clinic.  🞎 Treatment supporter not required at new clinic.  🞎 Fewer administrative requirements at new clinic.  🞎 Diagnosed with TB and sent to a TB clinic  **Work and Family**  🞎 New clinic is closer to work.  🞎 I had family obligations.  🞎 Family conflict prevented attending clinic.  🞎 My HIV status was less likely to be discovered by  my family at new clinic. | | | | | | | | | | | **Clinic Factor**  🞎 Old clinic ran out of medications (“stock-out”).  🞎 The staff is nicer at new clinic.  🞎 Care is better at new clinic.  🞎 The waiting area is more comfortable at new clinic.  🞎 My HIV status is less likely to be discovered by people I know at new clinic.  🞎 I was afraid old clinic would scold me for missing  appointment.  🞎 I was afraid of scolding at the old clinic in general, not  just for missing an appointment | | | | | | | | | | | | | | | |
|  |  |  |  |  |  |  |  |  |  |  |  |  |  |  |  |  | 🞎 I received more goods (i.e., food, bednets) at new  clinic  🞎 Declines to answer  🞎 Other, specify: | | | | | | | | | | | | | | | |
|  |  |  |  |  |  | Describe details of patient’s response: | | | | | | | | | | | | | | | | | | | | | | | | | | |
|  |  |  |  |  |  |  |  |  |  |  |  |  |  |  |  |  |  |  |  |  |  |  |  |  |  |  |  |  |  |  |  |  |
| ⭘No | →B4e. Why did you stop going to **any** clinic for your HIV care? *Answer both coded and descriptive responses* | | | | | Coded responses: *Mark all that apply* | | | | | | | | | | | | | | | | | | | | | | | | | | |
|  |  |  |  |  |  | **Access to Care**  🞎Transportation was too difficult or expensive.  🞎 I didn’t have enough money to access care.  🞎 I spent too much time at clinic.  🞎 It was taking too long to start ART.  🞎 I couldn’t find a treatment supporter that was  required.  🞎 Too many administrative requirements.  **Medical / Psychosocial**  🞎 I felt too sick to come to clinic.  🞎 The medicine was not helping me feel better.  🞎 I was afraid of side effects from the medicine.  🞎 I felt well and thought I didn’t need care.  🞎 I didn’t want to take drugs forever.  🞎 I was taking too many pills a day.  🞎 I didn’t have enough food.  🞎 I was drinking alcohol.  🞎 Depression (i.e. “I don’t care about living  anymore” or “I don’t feel like doing things for my  health anymore”)  🞎 I don’t believe that my HIV status was ever  positive  🞎 I was re-tested for HIV and am negative  🞎 I have been cured of HIV by a traditional/religious  healer, though I have not been retested | | | | | | | | | | | **Work and Family**  🞎 Work interfered with picking up medications or visiting  clinic.  🞎 Work interfered with taking medications.  🞎 I had family obligations.  🞎 Family conflict prevented attending clinic.  🞎 Attending clinic risked disclosure to my family that I had  HIV.  **Clinic Factor**  🞎 The clinic ran out of medications (“stock-out”).  🞎 The staff was not nice.  🞎 The care was not good.  🞎 The waiting area was not comfortable.  🞎 Attending clinic risked disclosure that I had HIV.  🞎 I was afraid clinic would scold me for missing  appointment.  🞎 I stopped receiving goods (i.e food, bednets) at clinic.  **Alternative Treatment and Advice**  🞎 A family member or other important person told me to  stop going to clinic.  🞎 My doctor or nurse told me to stop going to clinic.  🞎 Because I went to someone who tried / is trying to  cure me by prayer / religious rituals.  🞎 Because I saw / am seeing a traditional healer  instead.  🞎 Declines to answer  🞎 Other, specify: | | | | | | | | | | | | | | | |
|  |  |  |  |  |  | Describe details of patient’s response: | | | | | | | | | | | | | | | | | | | | | | | | | | |
|  |  | | | | | | | | | | | | | | | | | | | | | | | | | | | | | | | |
|  | →B4f. What things would make it easier to go back to the clinic?  *Answer both coded and descriptive responses* | | | | | Coded responses: *Mark all that apply* | | | | | | | | | | | | | | | | | | | | | | | | | | |
|  |  |  |  |  |  | **Access to Care**  🞎 Transportation money.  🞎 Shorter waiting times.  **Work and Family**  🞎 Better support from family/friends  🞎 Help with disclosure to family/friends  **Medical / Psychosocial**  🞎 I felt too sick to come to clinic / it would be easier  to come to clinic if I felt better | | | | | | | | | | | **Clinic Factor**  🞎 Nicer staff.  🞎 More privacy/respect for confidentiality at the clinic.  🞎 Goods provided by the clinic (i.e food, bednets).  **Alternative Treatment and Advice**  🞎 Better communication with / acceptance of traditional  healers by clinic staff.  🞎 Support/encouragement to attend clinic from my  pastor  🞎 Declines to answer  🞎 Other, specify: | | | | | | | | | | | | | | | |
|  |  |  |  |  |  | Describe details of patient’s response: | | | | | | | | | | | | | | | | | | | | | | | | | | |
|  |  |  |  |  |  |  |  |  |  |  |  |  |  |  |  |  |  |  |  |  |  |  |  |  |  |  |  |  |  |  |  |  |
| B5. ***This question is only for living patients not yet started on ART at original clinic. Others GO TO B6.***  Have you ever taken antiretroviral medications? | | | | | | | | | | | | | | | | | | | | | | | | | | | | | | | | |
| ⭘Yes | 🡪B5a. When did you start? | | | | | | | | | | | - -  DAY(dd) MONTH(mmm) YEAR(yy) | | | | | | | | | | | | | | | | | | | | |
| ⭘ No | **→ Go to Section E** | | | | | | | | | | | | | | | | | | | | | | | | | | | | | | | |
| B6. Have you taken antiretroviral medicine (defined as any ART) in the last 14 days? | | | | | | | | | | | | | | | | | | | | | | | | | | | | | | | | |
| ⭘Yes | → B6a. Where did you get the medicine?  *Mark all that apply* | | | | | | | | | | | 🞎 Original clinic 🞎 Friend or family member  🞎 Other clinic, specify: 🞎 Other, specify: | | | | | | | | | | | | | | | | | | | | |
|  | → B6b. Can you show me or name your medicines? *If phone interview ask “Can you name your medicines?”* | | | | | | | | | | ⭘ Yes, → *Record the medicines*  ⭘ No, patient unable to name or show medicines | | | | | | 1. | | | | | 2. | | 3. | | | | | 4. | | | |

| ⭘No | → B6c. When did you stop ART? | | - - | | | |
| --- | --- | --- | --- | --- | --- | --- |
|  |  |  | DAY(*dd*) | MONTH (*mmm*) | YEAR (*yy*) | |
|  | → B6d. Why did you stop ART? *Answer both coded and descriptive responses* | Coded responses: *Mark all that apply* | | | | |
|  |  | **Access to Care**  🞎 Transportation was too difficult or expensive.  🞎 I didn’t have enough money to access care.  🞎 I spent too much time at clinic.  🞎 Too many administrative requirements.  **Work and Family**  🞎 Work interfered with picking up medications or visiting  clinic for review.  🞎 Work interfered with taking medications.  🞎 I had family obligations.  🞎 Family conflict prevented attending clinic.  🞎 Attending clinic risked disclosure to my family that I  had HIV.  **Medical**  🞎 I felt too sick to take the medicines.  🞎 The medicine was not helping me feel better.  🞎 I was experiencing side effects from the medicine.  🞎 I felt too sick to come to clinic.  🞎 I felt well and thought I didn’t need care.  🞎 I didn’t want to take drugs forever.  🞎 I was taking too many pills a day.  🞎 I didn’t have enough food.  🞎 I was drinking alcohol.  🞎 Depression (i.e. “I don’t care about living  anymore” or “I don’t feel like doing things for my health anymore”)  🞎 I don’t believe that my HIV status was ever positive  🞎 I was re-tested for HIV and am negative  🞎 I have been cured of HIV by a traditional/religious  healer, though I have not been retested | | | | **Clinic Factor**  🞎 The clinic ran out of medications (“stock-out”).  🞎 The staff was not nice.  🞎 The care was not good.  🞎 The waiting area was not comfortable.  🞎 Attending clinic risked disclosure that I had HIV.  🞎 I stopped receiving goods (i.e food, bednets) at  clinic.  **Alternative Treatment and Advice**  🞎 A family member or other important person told me  to stop taking ART.  🞎 My doctor or nurse told me to stop taking ART.  🞎 Because I went to someone who tried / is trying to  cure me by prayer / religious rituals.  🞎 Because I saw / am seeing a traditional healer  instead.  🞎 Declines to answer  🞎 Other, specify:  _________________________________________  _________________________________________  _________________________________________  _________________________________________  _________________________________________ |
|  |  | Describe details of patient’s response: | | | | |
|  |  |  |  |  |  |  |
| **→ GO TO SECTION E** | | | | | | |

| **SECTION C: TRACKER COMMUNICATES WITH INFORMANT(S) WHO KNOWS THE PATIENT (NO COMMUNICATION WITH PATIENT)** | | | | | | | | | | | | | | | | | | | | | |
| --- | --- | --- | --- | --- | --- | --- | --- | --- | --- | --- | --- | --- | --- | --- | --- | --- | --- | --- | --- | --- | --- |
| C1. Interview type: *Mark all that apply for all of the informants.* | | | | | in-person phone | | | | | C2. Interview date. | | | - - | | | | | | | | |
|  |  |  |  |  |  |  |  |  |  |  |  |  | DAY(*dd*) | | | | MONTH (*mmm*) | | | | YEAR (*yy*) |
| C3. Confirm identity of the patient using as many identifiers as possible. *Mark all that apply.* | | | | | | | | | | | | | | | | | | | | | |
| 🞎 Name 🞎 Age 🞎 Gender 🞎 Height 🞎 Location or residence 🞎 Occupation 🞎 Clinic number or medical papers | | | | | | | | | | | | | | | | | | | | | |
| C4. What is your relationship to [state patient’s name]? *Mark all that apply for all of the informants who provided important information*. | | | | | | | | | | | | | | | | | | | | | |
| 🞎 Spouse | | 🞎 Grandparent | | 🞎 Traditional healer | | 🞎 Parent | | | | 🞎 Local leader | | | | 🞎 Doctor or nurse | | | | | | | |
| 🞎 Neighbor | | 🞎 Clergyman | | 🞎 Child | | 🞎 Friend | | | | 🞎 Brother or sister | | | | 🞎 Other relative | | | | | | | |
| 🞎 Other, specify: | | | | | | | | | | | | | | | | | | | | | |
| C5. As far as you are aware, is the patient alive? | | | | | | | | | | | | | | | | | | | | | |
| ⭘Yes | → C5a. When was the last time you or someone else you know had contact with the patient? | | | | | | | - - | | | | | | | | | |  | | | |
|  |  |  |  |  |  |  |  | DAY(*dd*) MONTH (*mmm*) YEAR(*yy*) | | | | | | |  | | | | | | |
|  | → C5b. Did he/she move away? | | | | | | | ⭘ Yes | | | ⭘ No | | | ⭘ Unknown | | | | | | | |
|  | → C5c. Has he/she been receiving medical services? | | | | | | | ⭘ Yes | | | ⭘ No | | | ⭘ Unknown | | | | | ⭘ Not asked | | |
|  | → C5d. Has he/she seen a doctor or nurse for the care or treatment of HIV within the last four weeks? | | | | | | | ⭘ Yes | | | ⭘ No | | | ⭘ Unknown | | | | | ⭘ Not asked | | |
|  | → C5e. Has he/she taken antiretroviral medicine (defined as any ART) in the last 14 days? | | | | | | | ⭘ Yes | | | ⭘ No | | | ⭘ Unknown | | | | | ⭘ Not asked | | |
|  | → ***Questions C5f is only for living patients not yet started on ART at original clinic.*** | | | | | | | | | | | | | | | | | | | | |
|  | → C5f. Has he/she started ART somewhere else besides [name of original clinic]? | | ⭘ Yes  ⭘ No→**Go to Section E**  ⭘ Not asked →**Go to Section E**  ⭘ Unknown→**Go to Section E** | | | | →C5f(i). When did he/she start ART? | | | | - - | | | | | | | | | ⭘ Unknown | |
|  |  |  |  |  |  |  |  |  |  |  | DAY(*dd*) | MONTH (*mmm*) | | | | YEAR(*yy*) | | | |  | |
|  | **→ GO TO SECTION E** | | | | | | | |  | | | | | | | | | | | | |

| ⭘ No, Patient has died | → C5g. When did the patient pass away? | | | | - - | | | | | | | | |
| --- | --- | --- | --- | --- | --- | --- | --- | --- | --- | --- | --- | --- | --- |
|  |  |  |  |  | DAY(*dd*) | | MONTH (*mmm*) YEAR(*yy*) | | | | | |  |
|  | → C5h. How did he/she die? *Mark one.* | | | |  | | | | | | | | |
|  | ⭘ Suicide ⭘ Disease or illness ⭘ Injury, accident, or trauma ⭘ Relating to childbirth ⭘ Don’t know/declines to answer  ⭘ Other, specify: | | | | | | | | | | | | |
|  | → C5i. Had he/she seen a doctor or nurse for the care or treatment of HIV within the last three months before death? | | | ⭘ Yes | | ⭘ No | | | ⭘ Unknown | | ⭘ Not asked | | |
|  | → C5j. Had he/she taken antiretroviral medicine (defined as any ART) in the last 14 days before death? | | | ⭘ Yes | | ⭘ No | | | ⭘ Unknown | | ⭘ Not asked | | |
|  | → ***Questions C5k is only for deceased patients not yet started on ART at original clinic.*** | | | | | | | | | | | | |
|  | → C5k. Had he/she started ART somewhere else besides [name of original clinic]? | ⭘ Yes  ⭘ No→**Go to Section E**  ⭘ Not asked →**Go to Section E**  ⭘ Unknown→**Go to Section E** | →C5k(i). When did he/she start ART? | | - - | | | | | | | ⭘ Unknown | |
|  | | | | | DAY(*dd*) | | | MONTH (*mmm*) | | YEAR(*yy*) | | | |

| **SECTION D: TRACKER UNABLE TO COMMUNICATE WITH PATIENT OR INFORMANT** Check local death registry: | | | | | | | | | |
| --- | --- | --- | --- | --- | --- | --- | --- | --- | --- |
| D1. Is the patient dead? | ⭘ Yes  ⭘ No evidence of death in registry (registry  consulted)  ⭘ Unknown (no registry data available) | | | | → D1a. When did the patient die? | | - -  DAY(dd) MONTH(mmm) YEAR(yy) | | |
| **→ GO TO SECTION E** | | | | | | | | | |
| **SECTION E: TRACKER FEEDBACK (complete after return to research center)** | | | | | | | | | |
| E1. How many persons were questioned by phone or in person while looking for this patient? | | | |  |  | | --- | --- | | E2. How many hours were spent by phone or in person looking for this patient? | | | | : | |
|  |  |  |  |  |  |  |  | Hours *(hh)* : Minutes *(mm*) | |
| E2b. How many trips were made looking for this patient? | | |  |  | | --- | --- | | ⭘ Not applicable, phone tracking only | | | | | | |
| E3. Does patient have a new site or residence? | | ⭘ Yes: Address known → *Update contact form*  ⭘ Yes: He/she has moved but do not know where  ⭘ No  ⭘ Don’t Know ⭘ Not applicable (died) | | | | E4. Does the patient have a new phone contact number? | | | ⭘ Yes: *Update contact form*  ⭘ No  ⭘ Don’t Know  ⭘ Not applicable (died) |

| **Skip to E6 if patient or key informant interviewed.** “Site” refers to smallest geographic unit available to tracker in clinic records. “Residence” is the actual building or dwelling where the patient stays or has stayed. | | | | | | |
| --- | --- | --- | --- | --- | --- | --- |
| E5. What was the tracking outcome? | | | | | | |
| ⭘ Site found and residence found | | **→**E5a. Why was no further information about the patient obtained? | | ⭘ No one available to speak to  ⭘ None of the available persons were willing to talk  ⭘ None of the available persons knew the patient  ⭘ No one willing to talk about the patient even though they know him/her  ⭘ No one knows the patient’s current vital status or care status even though they know  him/her  ⭘ Other, specify: | | |
| ⭘ Site found but residence not found | | **→**E5b. Why were you unable to find the residence? | | ⭘ Site refers to an area that is too big to be able to narrow search for patient  ⭘ No one available to speak to  ⭘ None of the available persons was willing to talk  ⭘ None of the available persons knew the patient  ⭘ No one willing to talk about residence even though they know him/her  ⭘ No one knows about residence even though they know him/her  ⭘ Other, specify: | | |
| ⭘ Site not found and therefore residence of patient was not found | | **→**E5c. Why were you unable to find the site given by the clinic? | | ⭘ Site does not exist  ⭘ Site inaccessible (e.g.,flood)  ⭘ Site data not legible enough to understand  ⭘ Other, specify: | | |
| ⭘ Phone tracking only; no geographic information available for tracking | | **→**E5d. Why was phone tracking unsuccessful? | | ⭘ The phone number is invalid  ⭘ No one answered the phone  ⭘ The person answering the phone does not know the patient  ⭘ The person answering the phone refused to provide information about the patient  ⭘ Other, specify: | | |
| E6. Describe any unusual events that took place during the tracking | | | 33 | | | |
| E7. Describe any “lessons learned” in tracking this patient. | | |  | | | |
| **SECTION F: ELIGIBILITY** | | | | | | |
| F1. Is patient eligible for study? | | | | | | |
| ⭘ Yes | F1a. Does participant agree to participate in the study? | | | | F1b. Reasons for refusal | |
|  | ⭘ Yes **🡪 move to consenting process**  ⭘ No **🡪 go to F1b** | | | | ⭘ Too busy  ⭘ Not interested in participating in research  ⭘ Concerned that others will learn his/her HIV status if he/she participates  ⭘ Does not want to participate in kanyakla  ⭘ Does not want to participate in EK programs in general  ⭘ Other, specify: | |
| ⭘ No | F1.c Reason for ineligibility (check all that apply) | | | | | |
|  | **Patient contacted directly**  ⭘ In care at original facility – missed visit was an error in the  clinic record  ⭘ In care at a new facility within the study area  ⭘ In care at a new facility outside the study area  ⭘ Not in care, but permanently moved outside study area | | | | | **Patient not found, informant interviewed**  ⭘ In care at a new facility within the study area  ⭘ In care at a new facility outside the study area  ⭘ Patient transferred to another facility, but location is unknown  ⭘ Patient moved outside study area, not in care  ⭘ Patient moved outside study area, in care at another clinic  ⭘ Patient moved outside study area, unknown whether they are  in care  ⭘ Patient died  **Neither patient, nor informant was located**  ⭘ Patient whereabouts unknown  ⭘ Death registry indicates that patient is dead |

| Kanyakla Study  Date of Interview: //  Day Month Year | | | Participant ID:  Interviewer Initials: | | | | | |
| --- | --- | --- | --- | --- | --- | --- | --- | --- |
| ***ADMINISTRATIVE*** | | | | | | | | |
|  | Has participant signed consent form? | 1 ⭘ No 🡪 **complete consent form before proceeding**  _2_ ⭘ Yes | | | | | | |
| A.1 | Participant name (from official age verification document) |  | | | | | | |
| A.2 | Common name(s) |  | | | | | | |
| A.3 | Record participant’s gender | 1 ⭘ Male  _2_ ⭘ Female | | | | | |  |
| A.4 | What is your age? | years | | | | | | 99 ⭘ Don’t know |
| A.5 | Date of birth (from official age verification document) | //  Day Month Year | | | | | | 99 ⭘ Don’t know |
| A.6 | Where do you live? | 1 ⭘ Mfangano East  _2_ ⭘ Mfangano North  _3_ ⭘ Mfangano West  _4_ ⭘ Mfangano South | | 5 ⭘ Ringiti  _6_ ⭘ Remba  _7_ ⭘ Takawiri  _8_ ⭘ Mainland - specify _____________________ | | | | |
| A.7 | Village name |  | | | |  |  | |
| A.8 | Detailed locator information |  | | | |  |  | |
| A.9 | Do you plan to move, either temporarily or permanently, in the next 12 months? | 1 ⭘ No 🡪 **Skip to A.11**  _2_ ⭘ Yes 🡪 (Consider not eligible if planning to move out of the study area permanently within the next 12 months). | | | | |  | |
| A.10 | Where do you plan to move to and how can we contact you after your planned move? |  | | | | |  | |
| A.11 | Participant phone number(s) |  | | | | |  | |
| A.12 | Alternate contact person #1 (if staff is unable to locate participant | Name:  Locator information: | | | Relationship: | | Phone: | |
| A.13 | Alternate contact person #2 (if staff is unable to locate participant | Name:  Locator information: | | | Relationship: | | Phone: | |
| A.14 | Draw the map showing direction to the participant’s home from the research center. Record any additional notes. | | | | | | | |

| Kanyakla Study  Date of Interview: //  Day Month Year | | | | | | | | Participant ID:  Interviewer Initials: | | | | |
| --- | --- | --- | --- | --- | --- | --- | --- | --- | --- | --- | --- | --- |
| ***SECTION A. KANYAKLA INFORMATION*** | | | | | | | | | | | | |
| A1. | What is your marital status? | | | 1 ⭘ never married  _2_ ⭘ married, living together  _3_ ⭘ divorced  _4_ ⭘ remarried | | | | 5 ⭘ widowed  _6_ ⭘ separated  _7_ ⭘ married, living apart  _8_ ⭘ other, specify ______ | | |  | |
| A2. | What is the highest level of school you completed? | | | 1 ⭘ none  _2_ ⭘ some primary  _3_ ⭘ primary  _4_ ⭘ some secondary  _5_ ⭘ secondary | | | | 6 ⭘ some college  _7_ ⭘ certificate  _8_ ⭘ diploma  _8_ ⭘ degree  _9_ ⭘ postgraduate | A3. | Have you completed any other type of vocational school?  _0_ ⭘ No  _1_ ⭘ Yes | | |
| A4. | What is your primary occupation? | | 1 ⭘ Farmer  _2_ ⭘ Fishing/Fishmonger  _3_ ⭘ Shopkeeper/Market vendor  _4_ ⭘ Bar owner/worker  _5_ ⭘ Hotel owner/worker  _6_ ⭘ Motorbike driver  _7_ ⭘ Boat driver/worker | | | | | 8 ⭘ Tourism  _9_ ⭘ Teacher  _10_ ⭘ Student  _11_ ⭘ Government worker  _12_ ⭘ Military  _13_ ⭘ Healthcare worker  _14_ ⭘ Construction worker/fundi | | | 15 ⭘ Disabled  _16_ ⭘ No job  _17_ ⭘ Other  Specify______________  _88_ ⭘ Decline to answer | |
| A5. | Does your family own your home or do you pay rent? | | | 1 ⭘ Rent  _2_ ⭘ Own | | | | | | | | |
| A6. | How many people usually live in your household (including yourself)? By household we mean all of the people who usually stay in the same house and eat some of their meals together. This may include husband/wife/partner, children, grandchildren, parents, grandparents, siblings, nieces/nephews, etc. | | | | | | | | | | | **Number of**  **people** |
| A7. | **OTHER INCOME INFO: ASSET INDEX (check if yes)**  _1_ ⭘ Clock or watch  _2_ ⭘ Electricity  _3_ ⭘ Radio  _4_ ⭘ Television  _5_ ⭘ Mobile phone | | | | | | | 6 ⭘ Solar panel  _7_  ⭘ Laptop or computer  _8_ ⭘ Bed  _9_ ⭘ Wall unit or cupboard  _10_ ⭘ Sofa set with removable cushions  _11_ ⭘ Sofa set with permanent cushions | | | | |
| **SECTION B. DISCLOSURE** | | | | | | | | | | | | |
| B1. | When were you diagnosed with HIV? | -- | | | B1b. | When did you first attend an HIV clinic? | | | -- | | | |
| B1b. | Are you taking ARVs?  (Not including Septra) | | | | | | 1 ⭘ No  _2_ ⭘ Yes: Start Date? -- | | | | | |
| B2. | Not counting healthcare workers, have you disclosed your HIV status to anyone? | | | | | | 0 ⭘ No **🡪 Skip to SECTION C.**  _1_ ⭘ Yes | | | | | |
| B2a. | Not counting healthcare workers, how many people have you disclosed your status to? | | | | | | Number of people  ⭘ Too many to count  ⭘ Decline to answer | | | | | |
| B2b. | Which groups of people have you disclosed to?  (check all that apply) | | | | | | 1 ⭘ Sexual partner  _2_ ⭘ Other family  _3_ ⭘ Friends  _4_ ⭘ Coworkers  _5_ ⭘ Members of a social group (e.g. support group, church, etc)  _6_ ⭘ Other, specify: | | | | | |

| **SECTION C. HIV-RELATED STIGMA** | | | | | |
| --- | --- | --- | --- | --- | --- |
| **How do you feel about being HIV positive?** | **Strongly disagree** | **Disagree** | **Neutral** | **Agree** | **Strongly Agree** |
| C1. Having HIV makes me feel like I’m a bad person | ⭘ | ⭘ | ⭘ | ⭘ | ⭘ |
| C2. I feel I’m not as good as others because I have HIV | ⭘ | ⭘ | ⭘ | ⭘ | ⭘ |
| C3. I feel ashamed of having HIV | ⭘ | ⭘ | ⭘ | ⭘ | ⭘ |
| C4. I think less of myself because I have HIV | ⭘ | ⭘ | ⭘ | ⭘ | ⭘ |
| C5. Having HIV makes me feel unclean | ⭘ | ⭘ | ⭘ | ⭘ | ⭘ |
| C6. Having HIV is disgusting to me | ⭘ | ⭘ | ⭘ | ⭘ | ⭘ |
| **How likely is it that people will treat you in the following ways in the future because of your HIV status?** | **Very unlikely** | **Unlikely** | **Neither unlikely nor likely** | **Likely** | **Very likely** |
| C7. Family members will avoid me | ⭘ | ⭘ | ⭘ | ⭘ | ⭘ |
| C8. Family members will look down on me | ⭘ | ⭘ | ⭘ | ⭘ | ⭘ |
| C9. Family members will treat me differently | ⭘ | ⭘ | ⭘ | ⭘ | ⭘ |
| C10. Community health workers won’t take my needs seriously | ⭘ | ⭘ | ⭘ | ⭘ | ⭘ |
| C11. Community health workers will discriminate against me | ⭘ | ⭘ | ⭘ | ⭘ | ⭘ |
| C12. Community health workers will deny me services | ⭘ | ⭘ | ⭘ | ⭘ | ⭘ |
| C13. Healthcare workers will not listen to my concerns | ⭘ | ⭘ | ⭘ | ⭘ | ⭘ |
| C14. Healthcare workers will avoid touching me | ⭘ | ⭘ | ⭘ | ⭘ | ⭘ |
| C15. Healthcare workers will treat me with less respect | ⭘ | ⭘ | ⭘ | ⭘ | ⭘ |
| **How often have people treated you this way in the past because of your HIV status?** | **Never** | **Not often** | **Somewhat often** | **Often** | **Very Often** |
| C16. Family members have avoided me | ⭘ | ⭘ | ⭘ | ⭘ | ⭘ |
| C17. Family members have looked down on me | ⭘ | ⭘ | ⭘ | ⭘ | ⭘ |
| C18. Family members have treated me differently | ⭘ | ⭘ | ⭘ | ⭘ | ⭘ |
| C19. Community health workers have not taken my needs seriously | ⭘ | ⭘ | ⭘ | ⭘ | ⭘ |
| C20. Community health workers have discriminated against me | ⭘ | ⭘ | ⭘ | ⭘ | ⭘ |
| C21. Community health workers have denied me services | ⭘ | ⭘ | ⭘ | ⭘ | ⭘ |
| C22. Healthcare workers have not listened to my concerns | ⭘ | ⭘ | ⭘ | ⭘ | ⭘ |
| C23. Healthcare workers have avoided touching me | ⭘ | ⭘ | ⭘ | ⭘ | ⭘ |
| C24. Healthcare workers have treated me with less respect | ⭘ | ⭘ | ⭘ | ⭘ | ⭘ |

| **SECTION D. SOCIAL NETWORK**  *I now want to ask a few questions about important people in our life. Please list each significant person in your life.*  *Consider all the persons who provide personal support for you or who are important to you now.* | | | | | | | | | | | |
| --- | --- | --- | --- | --- | --- | --- | --- | --- | --- | --- | --- |
| Person Code | a. Name | *b.* M*ale or female?*  1 = Male  2 = Female | *c. How old is he/she?*  |__|__|  Years | *d. What is this person’s relationship with you?*  2 = WIFE/HUSBAND/LIVE-IN  PARTNER  3 = SON OR DAUGHTER  4 = SON/DAUGHTER-IN-LAW  5 = GRANDCHILD  6 = PARENT  7 = PARENT-IN-LAW  8 = BROTHER OR SISTER  9 = NIECE/NEPHEW  10 = AUNT/UNCLE (brother/sister to your parent)  11 = CLOSE FRIEND  12 = BUSINESS ASSOCIATE  13 = AQUAINTANCE  15 = OTHER specify: | *e. If you were to form a kanyakla, would you want this person in your group?*  *0 – No*  *1 – Yes*  *2 – Unsure* | *f. Without telling me whether they are negative or positive, do you know this person’s HIV status?*  *0 – No*  *1 – Yes*  *88 – Decline to answer* | *g. Does this person know your HIV status?*  *0 – No*  *1 – Yes*  *88 – Decline to answer* | h. How much material support does this person offer you? (including financial assistance, food, school fees, etc.)  0 – None  1 – a little  2 – moderate  3 – quite a bit  4 – a great deal | i. How much emotional support does this person offer you? (including love/friendship, comfort, advice, and counseling)  0 – None  1 – a little  2 – moderate  3 – quite a bit  4 – a great deal | j. How much does support from this person help you attend you clinic appointments?  0-None  1-Not often  2-Somewhat often  3-Often  4-Everytime | k. How much does support from this person help you to take your HIV medication?  0-None  1-Not often  2-Somewhat often  3-Often  4-Every dose |
| 01 |  | 1 2 | |__|__|  ⭘ DK |  | 0 1 2 | 0 1 88 | 0 1 88 |  |  |  |  |
| 02 |  | 1 2 | |__|__|  ⭘ DK |  | 0 1 2 | 0 1 88 | 0 1 88 |  |  |  |  |
| 03 |  | 1 2 | |__|__|  ⭘ DK |  | 0 1 2 | 0 1 88 | 0 1 88 |  |  |  |  |
| 04 |  | 1 2 | |__|__|  ⭘ DK |  | 0 1 2 | 0 1 88 | 0 1 88 |  |  |  |  |
| 05 |  | 1 2 | |__|__|  ⭘ DK |  | 0 1 2 | 0 1 88 | 0 1 88 |  |  |  |  |
| 06 |  | 1 2 | |__|__|  ⭘ DK |  | 0 1 2 | 0 1 88 | 0 1 88 |  |  |  |  |
| 07 |  | 1 2 | |__|__|  ⭘ DK |  | 0 1 2 | 0 1 88 | 0 1 88 |  |  |  |  |
| 08 |  | 1 2 | |__|__|  ⭘ DK |  | 0 1 2 | 0 1 88 | 0 1 88 |  |  |  |  |
| 09 |  | 1 2 | |__|__|  ⭘ DK |  | 0 1 2 | 0 1 88 | 0 1 88 |  |  |  |  |
| 10 |  | 1 2 | |__|__|  ⭘ DK |  | 0 1 2 | 0 1 88 | 0 1 88 |  |  |  |  |

| **SECTION E. SUPPORT FOR HIV CARE** | | | | | | |
| --- | --- | --- | --- | --- | --- | --- |
| **Please evaluate the following statements:** | | | | | | |
|  | **Never** | **Not often** | **Somewhat often** | | **Often** | **Every time/ every dose** |
| E1. In the last 6 months, I received assistance/support from any friends or family in attending my HIV clinic appointments | ⭘ | ⭘ | ⭘ | | ⭘ | ⭘ |
| E1b. Please check all types of support received: | 🞎 Encouragement  🞎 Reminders  🞎 Financial assistance  🞎 Transportation (non-financial) | | | 🞎 Help with duties at home (i.e. childcare, chores)  🞎 Help with duties at work  🞎 Accompanyment (i.e. someone goes with me to my appointments) | | |
| E2. In the last 6 months, I received assistance/support from any friends or family in taking my HIV medications | ⭘ | ⭘ | ⭘ | | ⭘ | ⭘ |
| E2b. Please check all types of support received: | 🞎 Encouragement  🞎 Reminders  🞎 Gives me food to take with medications  🞎 Advice about side effects | | | 🞎 Observation (someone watches me take my medications)  🞎 Accompanyment (someone takes their medications with me at the same time) | | |

| Kanyakla Study  Date of Interview: //  Day Month Year | | | | | | | | | Participant ID:  Interviewer Initials: | | | | | |
| --- | --- | --- | --- | --- | --- | --- | --- | --- | --- | --- | --- | --- | --- | --- |
| ***TO BE COMPLETED BY VCT COUNSELOR*** | | | | | | | | | | | | | | |
| **SECTION A. HIV TEST** | | | | | | | | | | | | | | |
| A1. | Age: |  | A2. | Gender ⭘ Male  ⭘ Female | | | | | | | | | |  |
| A3. | Have you been tested for HIV before? | | 0 ⭘ No 🡪 Skip to A8.  _1_ ⭘ Yes 🡪 A3a. and A3b. | | | | A3a. | What was the result?  ⭘ Negative  ⭘ Positive (A3b.) | | | | A3b. | What date did you find out you were HIV positive?  : //  Day Month Year | |
| A4. | Have you ever enrolled in HIV care? | | 0 ⭘ No 🡪 reasons why not enrolled, then skip to **section B**  _1_ ⭘ Yes | | | | | | | Reasons why not enrolled: | | | | |
| A5. | Which clinic? | | | ⭘ Sena  ⭘ Yokla  ⭘ Nyakweri  ⭘ Wakula  ⭘ Ugina  ⭘ Soklo  ⭘ Takawiri  ⭘ Ringiti  ⭘ Remba  ⭘ Other - specify ______________ | | | | | | A6. | Enrollment date  //  Day Month Year | | | |
|  |  |  |  |  |  |  |  |  |  | A7. | On ART?  ⭘ No  ⭘ Yes (record date of initiation)  //  Day Month Year | | | |
| A8. | What is the result of the test today? | | | | ⭘ Negative  ⭘ Positive  ⭘ Indeterminant | | | | | | | | | |
| **SECTION B. DISCLOSURE** | | | | | | | | | | | | | | |
| B1. | Not counting healthcare workers, have you disclosed your HIV status to anyone? | | | | | 0 ⭘ No **🡪 Skip to SECTION C.**  _1_ ⭘ Yes | | | | | | | | |
| B1a. | Not counting healthcare workers, how many people have you disclosed your status to? | | | | | Number of people  ⭘ Too many to count  ⭘ Decline to answer | | | | | | | | |
| B1b. | Which groups of people have you disclosed to?  (check all that apply) | | | | | 1 ⭘ Sexual partner  _2_ ⭘ Other family  _3_ ⭘ Friends  _4_ ⭘ Coworkers  _5_ ⭘ Members of a social group (e.g. support group, church, etc)  _6_ ⭘ Other, specify: _____________________ | | | | | | | | |
| **SECTION C. KANYAKLA INFORMATION** | | | | | | | | | | | | | | |
| C1. | What was the reason you decided to join kanyakla? | | | | | | | | 1 ⭘ Friend recruited me  _2_ ⭘ Thought group would help me  _3_ ⭘ Thought I could help group  _4_ ⭘ Thought there would be free stuff  _5_ ⭘ Other: Specify: _____________ | | | | | |

| Kanyakla name | |  | | | | | | Meeting location | |  | | | |  | |  | |  | |  | |  | |  |
| --- | --- | --- | --- | --- | --- | --- | --- | --- | --- | --- | --- | --- | --- | --- | --- | --- | --- | --- | --- | --- | --- | --- | --- | --- |
| # | Name | | | Participant ID | Gender | Age | | Person  1 | Person  2 | | Person  3 | Person  4 | Person  5 | | Person  6 | | Person  7 | | Person  8 | | Person  9 | | Person  10 | |
| 1 |  | | |  | ⭘ Male  ⭘ Female |  | | 1 |  | |  |  |  | |  | |  | |  | |  | |  | |
| 2 |  | | |  | ⭘ Male  ⭘ Female |  | |  | 1 | |  |  |  | |  | |  | |  | |  | |  | |
| 3 |  | | |  | ⭘ Male  ⭘ Female |  | |  |  | | 1 |  |  | |  | |  | |  | |  | |  | |
| 4 |  | | |  | ⭘ Male  ⭘ Female |  | |  |  | |  | 1 |  | |  | |  | |  | |  | |  | |
| 5 |  | | |  | ⭘ Male  ⭘ Female |  | |  |  | |  |  | 1 | |  | |  | |  | |  | |  | |
| 6 |  | | |  | ⭘ Male  ⭘ Female |  | |  |  | |  |  |  | | 1 | |  | |  | |  | |  | |
| 7 |  | | |  | ⭘ Male  ⭘ Female |  | |  |  | |  |  |  | |  | | 1 | |  | |  | |  | |
| 8 |  | | |  | ⭘ Male  ⭘ Female |  | |  |  | |  |  |  | |  | |  | | 1 | |  | |  | |
| 9 |  | | |  | ⭘ Male  ⭘ Female |  | |  |  | |  |  |  | |  | |  | |  | | 1 | |  | |
| 10 |  | | |  | ⭘ Male  ⭘ Female |  | |  |  | |  |  |  | |  | |  | |  | |  | | 1 | |
| **Relationship code**  1 = SELF  2 = WIFE/HUSBAND/LIVE-IN  PARTNER  3 = SON OR DAUGHTER  4 = SON/DAUGHTER-IN-LAW  5 = GRANDCHILD  6 = PARENT  7 = PARENT-IN-LAW | | | 8 = BROTHER OR SISTER  9 = NIECE/NEPHEW  10 = AUNT/UNCLE (brother/sister to your parent)  11 = CLOSE FRIEND  12 = BUSINESS ASSOCIATE  13 = AQUAINTANCE  14 = MEETING FOR THE FIRST TIME  15 = OTHER specify: | | | | **Notes** | | | | | | | | | | | | | | | | | |

| 21-APR-2014 | | **LOST TO FOLLOW-UP TRACING FORM** | | | | | | | | | | Reviewer’s initials and date of review: | | | |  | | |
| --- | --- | --- | --- | --- | --- | --- | --- | --- | --- | --- | --- | --- | --- | --- | --- | --- | --- | --- |
| **SECTION A: ADMINISTRATIVE** | | | | | | | | | | | |  |  |  |  | - -  DAY(*dd*) MONTH (mmm) YEAR (yy) | | |
| A1. Patient name: | | |  | | | | | | | A2. Patient age | | | |  | | | A3. Patient gender | ⭘ Male  ⭘ Female |
| A4. Clinic name: | | |  | | | | | | | A5. Patient clinic number: | | | |  | | | | |
| A6. Date of initial enrollment in clinic: | | | | - -  DAY(*dd*) MONTH (mmm) YEAR (yy) | | | | | A7. Date last seen in clinic: | | | | - -  DAY(*dd*) MONTH (mmm) YEAR (yy) | | | | | |
| A8. Staff initials: | | |  | | | A9. First date of tracking: | | - -  DAY(*dd*) MONTH (mmm) YEAR (yy) | | | | | A10. ART status of patient: | | | | Not yet started ART  Has started ART | |
| A11. Source of information: | | | ⭘ Communicated with patient 🡪 go to **SECTION B**  ⭘ Communicated with informant(s) who knows the patient and not directly with patient 🡪 go to **SECTION C**  ⭘ Attempted but unable to communicate with patient or informant 🡪 go to **SECTION D**  ⭘ Tracking not attempted 🡪 go to **A11a** | | | | | | | | | | | | | | | |
| A11a. Why was tracking not attempted? | | | ⭘ Chart reviewed and patient not lost to follow up  **🡪 go to A11b**  ⭘ Other, specify:  🡪 **END** | | | | | | | | →A11b. What is patient outcome? | | | | ⭘ Has recent visit – doesn’t  meet definition of lost  ⭘ Patient died without missing a clinic visit  Date:  - -  DAY(*dd*) MONTH (mmm) YEAR (yy) | | | |
| **SECTION B: TRACKER COMMUNICATES WITH PATIENT** | | | | | | | | | | | | | | | | | | |
| B1. Interview type. *Mark all that apply:* | | | | | | | in-person phone | | | B2. Interview date. | | | | - -  DAY(*dd*) MONTH (mmm) YEAR (yy) | | | | |
| B3. Confirm identity of patient using as many identifiers as possible. *Mark all that apply:* | | | | | | | | | | | | | | | | | | |
| 🞎 Name 🞎 Age 🞎 Sex 🞎 Height 🞎 Location or residence 🞎 Occupation 🞎 Clinic number or medical papers | | | | | | | | | | | | | | | | | | |
| B4. Have you seen a doctor or nurse for the care or treatment of HIV within the last three months? | | | | | | | | | | | | | | | | | | |
| ⭘Yes | →B4a. Where?  *Mark all that apply.* | | | | 🞎 Original clinic  **→ Question patient about missed visit**  🞎 Different clinic  🞎 Other, specify: | | | →B4b. Name of clinic or other | | →B4c.  When did you first make a visit to **any** new clinic? | | | | | - -  DAY(*dd*) MONTH (mmm) YEAR (yy) | | | |
|  |  |  |  |  |  |  |  |  |  |  |  |  |  |  |  |  |  |  |
|  |  |  |  |  |  |  |  |  |  |  |  |  |  |  |  |  |  |  |
|  | →B4d. Why are you going to [new clinic] instead of your previous one? *Answer both coded and descriptive responses* | | | | Coded responses: *Mark all that apply* | | | | | | | | | | | | | |
|  |  |  |  |  | **Access to Care**  🞎 I moved - now transportation is easier or cheaper to  new clinic.  🞎 I have not moved, but transportation is easier or cheaper to  new clinic.  🞎 I spend less money at new clinic.  🞎 I spend less time at new clinic.  🞎 Starting ART is easier new clinic.  🞎 Treatment supporter not required at new clinic.  🞎 Fewer administrative requirements at new clinic.  🞎 Diagnosis of TB and sent to a TB clinic  **Work and Family**  🞎 New clinic is closer to work.  🞎 I had family obligations.  🞎 Family conflict prevented attending clinic.  🞎 My HIV status was less likely to be discovered by my  family at new clinic. | | | | | | | **Clinic Factor**  🞎 Old clinic ran out of medications (“stock-  out”).  🞎 The staff is nicer at new clinic.  🞎 Care is better at new clinic.  🞎 The waiting area is more comfortable at new  clinic.  🞎 My HIV status is less likely to be discovered  by people I know at new clinic.  🞎 I was afraid old clinic would scold me for  missing appointment.  🞎 I was afraid of scolding at the old clinic in  general, not just for missing an appointment | | | | | | |
|  |  |  |  |  |  |  |  |  |  |  |  | 🞎 I received more goods (i.e., food, bednets)  at new clinic  🞎 Declines to answer  🞎 Other, specify: | | | | | | |
|  |  |  |  |  | Describe details of patient’s response: | | | | | | | | | | | | | |
|  |  |  |  |  |  |  |  |  |  |  |  |  |  |  |  |  |  |  |

| ⭘No | →B4e. Why did you stop going to **any** clinic for your HIV care? *Answer both coded and descriptive responses* | Coded responses: *Mark all that apply* | | | |
| --- | --- | --- | --- | --- | --- |
|  |  | **Access to Care**  🞎Transportation was too difficult or expensive.  🞎 I didn’t have enough money to access care.  🞎 I spent too much time at clinic.  🞎 It was taking too long to start ART.  🞎 I couldn’t find a treatment supporter that was  required.  🞎 Too many administrative requirements.  **Medical / Psychosocial**  🞎 I felt too sick to come to clinic.  🞎 The medicine was not helping me feel better.  🞎 I was afraid of side effects from the medicine.  🞎 I felt well and thought I didn’t need care.  🞎 I didn’t want to take drugs forever.  🞎 I was taking too many pills a day.  🞎 I didn’t have enough food.  🞎 I was drinking alcohol.  🞎 Depression (i.e. “I don’t care about living anymore” or  “I don’t feel like doing things for my health anymore”)  🞎 I don’t believe that my HIV status was ever positive  🞎 I was re-tested for HIV and am negative  🞎 I have been cured of HIV by a traditional/religious  healer, though I have not been retested | | | **Work and Family**  🞎 Work interfered with picking up  medications or visiting clinic.  🞎 Work interfered with taking medications.  🞎 I had family obligations.  🞎 Family conflict prevented attending clinic.  🞎 Attending clinic risked disclosure to my  family that I had HIV.  **Clinic Factor**  🞎 The clinic ran out of medications (“stock-out”).  🞎 The staff was not nice.  🞎 The care was not good.  🞎 The waiting area was not comfortable.  🞎 Attending clinic risked disclosure that I had  HIV.  🞎 I was afraid clinic would scold me for  missing appointment.  🞎 I stopped receiving goods (i.e food,  bednets) at clinic.  **Alternative Treatment and Advice**  🞎 A family member or other important person  told me tostop going to clinic.  🞎 My doctor or nurse told me to stop going to  clinic.  🞎 Because I went to someone who tried / is  trying to cure me by prayer / religious rituals.  🞎 Because I saw / am seeing a traditional healer instead.  🞎 Declines to answer  🞎 Other, specify: |
|  |  | Describe details of patient’s response: | | | |
|  |  | | | | |
|  | →B4f. What things would make it easier to go back to the clinic?  *Answer both coded and descriptive responses* | | Coded responses: *Mark all that apply* | | |
|  |  |  | **Access to Care**  🞎 Transportation money.  🞎 Shorter waiting times.  **Work and Family**  🞎 Better support from family/friends  🞎 Help with disclosure to family/friends  **Medical / Psychosocial**  🞎 I felt too sick to come to clinic / it would be easier  to come to clinic if I felt better | | **Clinic Factor**  🞎 Nicer staff.  🞎 More privacy/respect for confidentiality at the clinic.  🞎 Goods provided by the clinic (i.e food, bednets).  **Alternative Treatment and Advice**  🞎 Better communication with / acceptance of traditional healers by clinic staff.  🞎 Support/encouragement to attend clinic from my pastor  🞎 Declines to answer  🞎 Other, specify: |
|  |  |  | Describe details of patient’s response: | | |
|  |  |  |  |  |  |
| B5. ***This question is only for living patients not yet started on ART at original clinic. Others GO TO B6.*** Have you ever taken antiretroviral medications? | | | | | |
| ⭘Yes | 🡪B5a. When did you start? | | | - -  DAY(dd) MONTH(mmm) YEAR(yy) | |
| ⭘ No | **→ Go to Section E** | | | | |

| B6. Have you taken antiretroviral medicine (defined as any ART) in the last 14 days? | | | | | | | | | | | | |
| --- | --- | --- | --- | --- | --- | --- | --- | --- | --- | --- | --- | --- |
| ⭘Yes | → B6a. Where did you get the medicine?  *Mark all that apply* | | | | 🞎 Original clinic 🞎 Friend or family member  🞎 Private physician 🞎 Other, specify: | | | | | | | |
|  | → B6b. Can you show me or name your medicines? *If phone interview ask “Can you name your medicines?”* | | | ⭘ Yes, → *Record the medicines*  ⭘ No, patient unable to name or show medicines | | 1. | | | 2. | | 3. | 4. |
| ⭘No | → B6c. When did you stop ART? | | | | - -  DAY(*dd*) MONTH (mmm) YEAR **(yy)** | | | | | | | |
|  | → B6d. Why did you stop ART? *Answer both coded and descriptive responses* | Coded responses: *Mark all that apply* | | | | | | | | | | |
|  |  | **Access to Care**  🞎 Transportation was too difficult or expensive.  🞎 I didn’t have enough money to access care.  🞎 I spent too much time at clinic.  🞎 Too many administrative requirements.  **Work and Family**  🞎 Work interfered with picking up medications or visiting  clinic for review.  🞎 Work interfered with taking medications.  🞎 I had family obligations.  🞎 Family conflict prevented attending clinic.  🞎 Attending clinic risked disclosure to my family that I had  HIV.  **Medical**  🞎 I felt too sick to take the medicines.  🞎 The medicine was not helping me feel better.  🞎 I was experiencing side effects from the medicine.  🞎 I felt too sick to come to clinic.  🞎 I felt well and thought I didn’t need care.  🞎 I didn’t want to take drugs forever.  🞎 I was taking too many pills a day.  🞎 I didn’t have enough food.  🞎 I was drinking alcohol. | | | | | **Clinic Factor**  🞎 The clinic ran out of medications (“stock-out”).  🞎 The staff was not nice.  🞎 The care was not good.  🞎 The waiting area was not comfortable.  🞎 Attending clinic risked disclosure that I had HIV.  🞎 I stopped receiving goods (i.e food, bednets) at clinic.  **Alternative Treatment and Advice**  🞎 A family member or other important person told me to  stop taking ART.  🞎 My doctor or nurse told me to stop taking ART.  🞎 Because I went to someone who tried / is trying to cure  me by prayer / religious rituals.  🞎 Because I saw / am seeing a traditional healer instead.  🞎 Declines to answer  🞎 Other, specify: | | | | | |
|  |  | Describe details of patient’s response: | | | | | | | | | | |
|  |  |  |  |  |  |  |  |  |  |  |  |  |
| **→ GO TO SECTION E** | | | | | | | | | | | | |
| **SECTION C: TRACKER COMMUNICATES WITH INFORMANT(S) WHO KNOWS THE PATIENT (NO COMMUNICATION WITH PATIENT)** | | | | | | | | | | | | |
| C1. Interview type: *Mark all that apply for all of the informants.* | | | | | in-person  phone | C2. Interview date. | | - -  DAY(*dd*) MONTH (mmm) YEAR **(yy)** | | | | |
| C3. Confirm identity of the patient using as many identifiers as possible. *Mark all that apply.* | | | | | | | | | | | | |
| 🞎 Name 🞎 Age 🞎 Gender 🞎 Height 🞎 Location or residence 🞎 Occupation 🞎 Clinic number or medical papers | | | | | | | | | | | | |
| C4. What is your relationship to [state patient’s name]? *Mark all that apply for all of the informants who provided important information*. | | | | | | | | | | | | |
| 🞎 Spouse | 🞎 Grandparent | | 🞎 Traditional healer | | 🞎 Parent | 🞎 Local leader | | | | 🞎 Doctor or nurse | | |
| 🞎 Neighbor | 🞎 Clergyman | | 🞎 Child | | 🞎 Friend | 🞎 Brother or sister | | | | 🞎 Other relative | | |
| 🞎 Other, specify: | | | | | | | | | | | | |

| C5. As far as you are aware, is the patient alive? | | | | | | | | | | | | | | | | | | | |
| --- | --- | --- | --- | --- | --- | --- | --- | --- | --- | --- | --- | --- | --- | --- | --- | --- | --- | --- | --- |
| ⭘Yes | → C5a. When was the last time you or someone else you know had contact with the patient? | | | | | - -  DAY(*dd*) MONTH (mmm) YEAR **(yy)** | | | | | | | | | | ⭘ Unknown | | | |
|  | → C5b. Did he/she move away? | | | | | ⭘ Yes | | | | | | ⭘ No | | ⭘ Unknown | | | | | |
|  | → C5c. Has he/she been receiving medical services? | | | | | ⭘ Yes | | | | | | ⭘ No | | ⭘ Unknown | | | ⭘ Not asked | | |
|  | → C5d. Has he/she seen a doctor or nurse for the care or treatment of HIV within the last three months? | | | | | ⭘ Yes | | | | | | ⭘ No | | ⭘ Unknown | | | ⭘ Not asked | | |
|  | → C5e. Has he/she taken antiretroviral medicine (defined as any ART) in the last 14 days? | | | | | ⭘ Yes | | | | | | ⭘ No | | ⭘ Unknown | | | ⭘ Not asked | | |
|  | → ***Questions C5f is only for living patients not yet started on ART at original clinic.*** | | | | | | | | | | | | | | | | | | |
|  | → C5f. Has he/she started ART somewhere else besides [name of original clinic]? | ⭘ Yes  ⭘ No→**Go to Section E**  ⭘ Not asked →**Go to Section E**  ⭘ Unknown→**Go to Section E** | | →C5f(i). When did he/she start ART? | | | | | | - -  DAY(*dd*) MONTH (mmm) YEAR **(yy)** | | | | | | | | ⭘ Unknown | |
|  | **→ GO TO SECTION E** | | | | | | | |  | | | | | | | | | | |
| ⭘ No, Patient has died | → C5g. When did the patient pass away? | | | | | | - -  DAY(*dd*) MONTH (mmm) YEAR **(yy)** | | | | | | | | | | | | |
|  | → C5h. How did he/she die? *Mark one.* | | | | | |  | | | | | | | | | | | | |
|  | ⭘ Suicide ⭘ Disease or illness ⭘ Injury, accident, or trauma ⭘ Relating to childbirth  ⭘ Don’t know/declines to answer ⭘ Other, specify: | | | | | | | | | | | | | | | | | | |
|  | → C5i. Had he/she seen a doctor or nurse for the care or treatment of HIV within the last three months before death? | | | | | | | ⭘ Yes | | | | | ⭘ No | | ⭘ Unknown | | ⭘ Not asked | | |
|  | → C5j. Had he/she taken antiretroviral medicine (defined as any ART) in the last 14 days before death? | | | | | | | ⭘ Yes | | | | | ⭘ No | | ⭘ Unknown | | ⭘ Not asked | | |
|  | → ***Questions C5k is only for deceased patients not yet started on ART at original clinic.*** | | | | | | | | | | | | | | | | | | |
|  | → C5k. Had he/she started ART somewhere else besides [name of original clinic]? | | ⭘ Yes  ⭘ No→**Go to Section E**  ⭘ Not asked →**Go to Section E**  ⭘ Unknown→**Go to Section E** | | →C5k(i). When did he/she start ART? | | | | | | - -  DAY(*dd*) MONTH (mmm) YEAR **(yy)** | | | | | | | | ⭘ Unknown |

| **SECTION D: TRACKER UNABLE TO COMMUNICATE WITH PATIENT OR INFORMANT** Check local death registry: | | | | | | | |
| --- | --- | --- | --- | --- | --- | --- | --- |
| D1. Is the patient dead? | | ⭘ Yes  ⭘ No evidence of death in registry  (registry consulted)  ⭘ Unknown (no registry data  available) | | → D1a. When did the patient die? | | | - -  DAY(*dd*) MONTH (mmm) YEAR **(yy)** |
| **→ GO TO SECTION E** | | | | | | | |
| **SECTION E: TRANSFER CLINIC DATA** | | | | | | | |
| F1. Did participant receive care from another health care facility at any time during the study? | | | | | | | |
| ⭘ Yes,  *list names and date of first attendance* | F1a. Facility name | |  | | F1b. Date first received services | - -  DAY(*dd*) MONTH (mmm) YEAR **(yy)** | |
|  | F1c. Facility name | |  | | F1d. Date first received services | - -  DAY(*dd*) MONTH (mmm) YEAR **(yy)** | |
|  | F1e. Facility name | |  | | F1f. Date first received services | - -  DAY(*dd*) MONTH (mmm) YEAR **(yy)** | |
|  | **🡪 Collect visit history from transfer clinics using VISIT HISTORY FORM** | | | | | | |
| ⭘ No | **🡪 END** | | | | | | |

| **SECTION F: TRACKER FEEDBACK** | | | | | | | | | |
| --- | --- | --- | --- | --- | --- | --- | --- | --- | --- |
| F1. How many persons were questioned by phone or in person while looking for this patient? | | | | |  |  | | --- | --- | | | F2. How many hours were spent by phone or in person looking for this patient? | | : | |
|  |  |  |  |  |  |  |  | Hours *(hh)* : Minutes *(mm*) | |
| F2b. How many trips were made looking for this patient? | | | |  |  | | --- | --- | | ⭘ Not applicable, phone tracking only | | | | | |
| F3. Does patient have a new site or residence? | | | ⭘ Yes: Address known → *Update contact form*  ⭘ Yes: He/she has moved but do not know where  ⭘ No  ⭘ Don’t Know ⭘ Not applicable (died) | | | | F4. Does the patient have a new phone contact number? | | ⭘ Yes: *Update contact form*  ⭘ No  ⭘ Don’t Know  ⭘ Not applicable (died) |
| **Skip to F6 if patient or key informant interviewed.** “Site” refers to smallest geographic unit available to tracker in clinic records. “Residence” is the actual building or dwelling where the patient stays or has stayed. | | | | | | | | | |
| F5. What was the tracking outcome? | | | | | | | | | |
| ⭘ Site found and residence found | **→**F5a. Why was no further information about the patient obtained? | | | | ⭘ No one available to speak to  ⭘ None of the available persons were willing to talk  ⭘ None of the available persons knew the patient  ⭘ No one willing to talk about the patient even though they know him/her  ⭘ No one knows the patient’s current vital status or care status even though they know him/her  ⭘ Other, specify: | | | | |
| ⭘ Site found but residence not found | **→**F5b. Why were you unable to find the residence? | | | | ⭘ Site refers to an area that is too big to be able to narrow search for patient  ⭘ No one available to speak to  ⭘ None of the available persons was willing to talk  ⭘ None of the available persons knew the patient  ⭘ No one willing to talk about residence even though they know him/her  ⭘ No one knows about residence even though they know him/her  ⭘ Other, specify: | | | | |
| ⭘ Site not found and therefore residence of patient was not found | **→**F5c. Why were you unable to find the site given by the clinic? | | | | ⭘ Site does not exist  ⭘ Site inaccessible (e.g.,flood)  ⭘ Site data not legible enough to understand  ⭘ Other, specify: | | | | |
| ⭘ Phone tracking only; no geographic information available for tracking | **→**F5d. Why was phone tracking unsuccessful? | | | | ⭘ The phone number is invalid  ⭘ No one answered the phone  ⭘ The person answering the phone does not know the patient  ⭘ The person answering the phone refused to provide information about the patient  ⭘ Other, specify: | | | | |
| F6. Describe any unusual events that took place during the tracking | |  | | | | | | | |
| F7. Describe any “lessons learned” in tracking this patient. | |  | | | | | | | |

| Kanyakla Study  Date of Interview: //  Day Month Year | | | | | | | Participant ID: Interviewer Initials: | |
| --- | --- | --- | --- | --- | --- | --- | --- | --- |
| **SECTION A. ADMINISTRATIVE [TO BE COMPLETED FROM CLINIC DATA]** | | | | | | | | |
| A1. At study closure, was patient actively in care at original clinic? | | | | | ⭘ Yes  ⭘ No **🡪 complete LTFU form** | | | |
| **SECTION B. MISSED VISITS**  ***Only for patients who miss a visit by 30-days and then come back to the same clinic. All other types of care gap will be addressed by LTFU form.*** | | | | | | | | |
| B1. During the past 12 months, did you receive more than 2-weeks supply of HIV medications from any source other than your regular clinic? | | | | | | | | |
| ⭘ Yes | B1a. What sources did you receive medication from? | | | B1b. What months did you receive medication from each of these sources? | | | | B1c. What type of medication? *(check all that apply)* |
|  | ⭘ Another clinic, name: | | |  | | | | ⭘ CTX  ⭘ ARVs |
|  | ⭘ Family/friends | | |  | | | | ⭘ CTX  ⭘ ARVs |
|  | ⭘ Other, specify: | | |  | | | | ⭘ CTX  ⭘ ARVs |
|  | **🡪 For any additional clinics patient visited, collect visit history from transfer clinics using VISIT HISTORY FORM** | | | | | | | |
| ⭘ No | **🡪 go to SECTION C** | | | | | | | |
| **SECTION C. DISCLOSURE** | | | | | | | | |
| C1. Not counting healthcare workers, have you disclosed your HIV status to anyone? | | | | | | | | |
| ⭘ Yes | | C1a. Not counting healthcare workers, how many people have you disclosed your status to? | | | | C1b. Which groups of people have you disclosed to?  (check all that apply) | | |
|  |  | Number of people | ⭘ Too many to count  ⭘ Refuse to answer | | | 1 ⭘ Sexual partner  _2_ ⭘ Other family  _3_ ⭘ Friends  _4_ ⭘ Coworkers  _5_ ⭘ Members of a group (e.g. support group, church, etc)  _6_ ⭘ Other, specify: | | |
| ⭘ No | | **🡪 skip to SECTION D** | | | | | | |

| **SECTION D. HIV-RELATED STIGMA** | | | | | |
| --- | --- | --- | --- | --- | --- |
| **How do you feel about being HIV positive?** | **Strongly disagree** | **Disagree** | **Neutral** | **Agree** | **Strongly Agree** |
| D1. Having HIV makes me feel like I’m a bad person | ⭘ | ⭘ | ⭘ | ⭘ | ⭘ |
| D2. I feel I’m not as good as others because I have HIV | ⭘ | ⭘ | ⭘ | ⭘ | ⭘ |
| D3. I feel ashamed of having HIV | ⭘ | ⭘ | ⭘ | ⭘ | ⭘ |
| D4. I think less of myself because I have HIV | ⭘ | ⭘ | ⭘ | ⭘ | ⭘ |
| D5. Having HIV makes me feel unclean | ⭘ | ⭘ | ⭘ | ⭘ | ⭘ |
| D6. Having HIV is disgusting to me | ⭘ | ⭘ | ⭘ | ⭘ | ⭘ |
| **How likely is it that people will treat you in the following ways in the future because of your HIV status?** | **Very unlikely** | **Unlikely** | **Neither unlikely nor likely** | **Likely** | **Very likely** |
| D7. Family members will avoid me | ⭘ | ⭘ | ⭘ | ⭘ | ⭘ |
| D8. Family members will look down on me | ⭘ | ⭘ | ⭘ | ⭘ | ⭘ |
| D9. Family members will treat me differently | ⭘ | ⭘ | ⭘ | ⭘ | ⭘ |
| D10. Community health workers won’t take my needs seriously | ⭘ | ⭘ | ⭘ | ⭘ | ⭘ |
| D11. Community health workers will discriminate against me | ⭘ | ⭘ | ⭘ | ⭘ | ⭘ |
| D12. Community health workers will deny me services | ⭘ | ⭘ | ⭘ | ⭘ | ⭘ |
| D13. Healthcare workers will not listen to my concerns | ⭘ | ⭘ | ⭘ | ⭘ | ⭘ |
| D14. Healthcare workers will avoid touching me | ⭘ | ⭘ | ⭘ | ⭘ | ⭘ |
| D15. Healthcare workers will treat me with less respect | ⭘ | ⭘ | ⭘ | ⭘ | ⭘ |
| **How often have people treated you this way in the past because of your HIV status?** | **Never** | **Not often** | **Somewhat often** | **Often** | **Very Often** |
| D16. Family members have avoided me | ⭘ | ⭘ | ⭘ | ⭘ | ⭘ |
| D17. Family members have looked down on me | ⭘ | ⭘ | ⭘ | ⭘ | ⭘ |
| D18. Family members have treated me differently | ⭘ | ⭘ | ⭘ | ⭘ | ⭘ |
| D19. Community health workers have not taken my needs seriously | ⭘ | ⭘ | ⭘ | ⭘ | ⭘ |
| D20. Community health workers have discriminated against me | ⭘ | ⭘ | ⭘ | ⭘ | ⭘ |
| D21. Community health workers have denied me services | ⭘ | ⭘ | ⭘ | ⭘ | ⭘ |
| D22. Healthcare workers have not listened to my concerns | ⭘ | ⭘ | ⭘ | ⭘ | ⭘ |
| D23. Healthcare workers have avoided touching me | ⭘ | ⭘ | ⭘ | ⭘ | ⭘ |
| D24. Healthcare workers have treated me with less respect | ⭘ | ⭘ | ⭘ | ⭘ | ⭘ |

| **SECTION E. SOCIAL NETWORK**  *I now want to ask a few questions about important people in our life. Please list each significant person in your life.*  *Consider all the persons who provide personal support for you or who are important to you now.* | | | | | | | | | | | |
| --- | --- | --- | --- | --- | --- | --- | --- | --- | --- | --- | --- |
| Person Code | a. Name | *b.* M*ale or female?*  1 = Male  2 = Female | *c. How old is he/she?*  |__|__|  Years | *d. What is this person’s relationship with you?*  2 = WIFE/HUSBAND/LIVE-IN PARTNER  3 = SON OR DAUGHTER  4 = SON/DAUGHTER-IN-LAW  5 = GRANDCHILD  6 = PARENT  7 = PARENT-IN-LAW  8 = BROTHER OR SISTER  9 = NIECE/NEPHEW  10 = AUNT/UNCLE (brother/sister to your parent)  11 = CLOSE FRIEND  12 = BUSINESS ASSOCIATE  13 = AQUAINTANCE  15 = OTHER specify: | *e. Was this person in your Kanyakla?*  *0 – No*  *1 – Yes*  *2 – I am not in a kanyakla* | *f. Without telling me whether they are negative or positive, do you know this person’s HIV status?*  *0 – No*  *1 – Yes*  *88 – Decline to answer* | *g. Does this person know your HIV status?*  *0 – No*  *1 – Yes*  *88 – Decline to answer* | h. How much material support does this person offer you? (including financial assistance, food, school fees, e.t.c.)  0 – None  1 – a little  2 – moderate  3 – quite a bit  4 – a great deal | i. How much emotional support does this person offer you? (including love/friendship, comfort, advice, and counseling)  0 – None  1 – a little  2 – moderate  3 – quite a bit  4 – a great deal | j. How much does does support from this person help you attend you clinic appointments?  0-None  1-Not often  2-Somewhat often  3-Often  4-Everytime | k. How much does does support from this person help you to take your HIV medication?  0-None  1-Not often  2-Somewhat often  3-Often  4-Every dose |
| 01 |  | 1 2 | |__|__|  ⭘ DK |  | 0 1 2 | 0 1 88 | 0 1 88 |  |  |  |  |
| 02 |  | 1 2 | |__|__|  ⭘ DK |  | 0 1 2 | 0 1 88 | 0 1 88 |  |  |  |  |
| 03 |  | 1 2 | |__|__|  ⭘ DK |  | 0 1 2 | 0 1 88 | 0 1 88 |  |  |  |  |
| 04 |  | 1 2 | |__|__|  ⭘ DK |  | 0 1 2 | 0 1 88 | 0 1 88 |  |  |  |  |
| 05 |  | 1 2 | |__|__|  ⭘ DK |  | 0 1 2 | 0 1 88 | 0 1 88 |  |  |  |  |
| 06 |  | 1 2 | |__|__|  ⭘ DK |  | 0 1 2 | 0 1 88 | 0 1 88 |  |  |  |  |
| 07 |  | 1 2 | |__|__|  ⭘ DK |  | 0 1 2 | 0 1 88 | 0 1 88 |  |  |  |  |
| 08 |  | 1 2 | |__|__|  ⭘ DK |  | 0 1 2 | 0 1 88 | 0 1 88 |  |  |  |  |
| 09 |  | 1 2 | |__|__|  ⭘ DK |  | 0 1 2 | 0 1 88 | 0 1 88 |  |  |  |  |
| 10 |  | 1 2 | |__|__|  ⭘ DK |  | 0 1 2 | 0 1 88 | 0 1 88 |  |  |  |  |

| **SECTION F. SUPPORT FOR HIV CARE** | | | | | | |
| --- | --- | --- | --- | --- | --- | --- |
| Please evaluate the following statements: | | | | | | |
|  | Never | Not often | Somewhat often | | Often | Very Often |
| F1. In the last 6 months, I received assistance/support from friends or family in attending my HIV clinic appointments | ⭘ | ⭘ | ⭘ | | ⭘ | ⭘ |
| F1b. Please check all types of support received: | 🞎 Encouragement  🞎 Reminders  🞎 Financial assistance  🞎 Transportation (non-financial) | | | 🞎 Help with duties at home (i.e. childcare, chores)  🞎 Help with duties at work  🞎 Accompanyment (i.e. someone goes with me to my appointments) | | |
| F2. In the last 6 months, I received assistance/support from friends or family in taking my HIV medications | ⭘ | ⭘ | ⭘ | | ⭘ | ⭘ |
| F2b. Please check all types of support received: | 🞎 Encouragement  🞎 Reminders  🞎 Gives me food to take with medications  🞎 Advice about side effects | | | 🞎 Observation (someone watches me take my medications)  🞎 Accompanyment (someone takes their medications with me at the same time) | | |

| **SECTION G. KANYAKLA INFORMATION** | | | | |
| --- | --- | --- | --- | --- |
| G1. Did you participate in a kanyakla group? | | | | |
| ⭘ Yes | G1a. What is the name of your kanyakla? | | |  |
|  | G1b. Did you participate in group VCT? | | | |
|  | ⭘ Yes | G1c. What were the reasons why you decided to participate in group VCT? *(check all that apply)* | | |
|  |  | ⭘ I had not disclosed to my group before, but wanted them to know my status.  ⭘ Group VCT was not the first time I was disclosing my status - I have already shared my status within my group.  ⭘ Group VCT was not the first time I was disclosing my status - I have already shared my status to many people in the community.  ⭘ I did not want to participate in group VCT but my kanyakla pressured me to participate.  ⭘ Other, specify: | | |
|  |  | Describe details of response: | | |
|  | ⭘ No | G1d. What were the reasons why you decided NOT to participate in group VCT? *(check all that apply)* | | |
|  |  | ⭘ I did not want my group members to know my status.  ⭘ I was afraid my group would not keep my status confidential.  ⭘ I did not think group VCT was important to attend.  ⭘ Other, specify: | | |
|  |  | Describe details of response: | | |
| ⭘ No | G1f. Were you offered a chance to join a kanyakla? *If participant is confused by this question, explain that you have not been told who has and has not been offered a chance to join kanyakla. This helps you make sure to get people’s answers as accurately as possible without making any assumptions.* | | | |
|  | ⭘ Yes | G1g. What were the reasons that you decided not to join? | | |
|  |  | ⭘ I did not have time for kanyakla  ⭘ I did not want to discuss HIV with others  ⭘ I did not want anyone to know my status.  ⭘ I did not think kanyakla was important  ⭘ I have a problem with EK or EK programs  ⭘ I wanted to form a kanyakla, but none of my family or friends wanted to join  ⭘ Other, specify: | | |
|  |  | Describe details of response: | | |
|  | ⭘ No | G1h. When the new round of kanyakla starts in a few months, would you like to join? | | |
|  |  | ⭘ Yes | 🡪 **record interest on KANYAKLA ENROLLMENT FORM** | |
|  |  | ⭘ No | G1i. What are the reasons that you do not want to join? | |
|  |  |  | ⭘ I do not have time for kanyakla  ⭘ I do not want to discuss HIV with others  ⭘ I do not want anyone to know my status.  ⭘ I do not think kanyakla was important  ⭘ I have a problem with EK or EK programs  ⭘ I want to form a kanyakla, but don’t think my family or friends will want to join  ⭘ Other, specify: | |
|  |  |  | Describe details of response: | |

**Kanyakla Study**

*Informed Consent for Index participants*

Investigator and Institutional Affiliations:

| **Name** | **Organization** | **Role on Project** |
| --- | --- | --- |
| Elizabeth Bukusi, MB.ChB., M. Med, MPH, PhD, PGD (Research ethics) | Kenya Medical Research Institute (KEMRI) | KEMRI-Principal Investigator |
| Craig Cohen, MD, MPH | University of California, San Francisco (UCSF) | Co-Investigator |
| Matt Hickey, MD candidate | University of California, San Francisco (UCSF) | Co-Investigator |
| Charles Salmen, MD, MPhil (Oxon.) | The Organic Health Response (OHR) | Co-Investigator |
| Dan Omollo, BSc, MPH candidate | The Organic Health Response (OHR) | Co-Investigator |
| Katie Fiorella, MPH | The Organic Health Response (OHR) | Co-Investigator |
| Dr. Betty Njoroge | Kenya Medical Research Institute (KEMRI) | Co-Investigator |
| Lindah Otieno | Family AIDS Care and Education Services (FACES) | Co-Investigator |
| Richard Magerenge | The Organic Health Response (OHR) | Research Coordinator |
| Gor Benard Ouma | The Organic Health Response (OHR) | Research Coordinator |
| Catie Glatz | University of Minnesota (UMN) | Research Coordinator |
| Elvin Geng, MD MPH | University of California, San Francisco (UCSF) | Study Consultant |
| Dr. CT Muga | Kenya Medical Research Institute (KEMRI) | Study Consultant |
| Dr. Norton Sang | Kenya Medical Research Institute (KEMRI) | Study Consultant |
| Daniel Zoughbie, MSc (Oxon.), DPhil | Microclinic International (MCI) | Study Consultant |

Introduction and Purpose

Researchers from the Ekialo Kiona Center are conducting a study to understand the effect of the Kanyakla *kanyakla* program on HIV care on Mfangano, Remba, and Ringiti Islands. Our research team also includes researchers from Organic Health Response, Kenya Medical Research Institute (KEMRI), the Global Micro-Clinic Project (GMCP), the University of California San Francisco (UCSF), and the University of Minnesota (UMN). You are being invited to participate in this study because you attend one of the clinics included in the study on Mfangano, Remba or Ringiti.

This study is trying to understand whether the *kanyakla* program helps HIV patients to attend their clinic appointments regularly. We have already tested the *kanyakla* program on a small scale in Mfangano East, and are now bringing the program to more people throughout Mfangano, Remba and Ringiti islands. Through this study, we hope to learn more about the strengths and weaknesses of *kanyakla*, to improve the program for the future, and to learn about whether *kanyakla* could be expanded to include other locations. This research will benefit the people of Mfangano, Remba, and Ringiti islands by providing health education. The information gathered from this study will also help improve the program, enable future expansion projects, and guide development of similar programs to improve care for people who are directly or indirectly affected by HIV/AIDS throughout Kenya and in other parts of the world.

Procedures

The study participants will be recruited from among HIV-infected patients receiving care from a health center on Mfangano, Remba or Ringiti islands. If you agree to participate in the study, you will be randomly assigned to one of two groups. Participants in the first group will go through the 6-session *kanyakla* program starting now, and the second group will begin the program starting about one year from now. This random assignment of when people can start kanyakla is the best way to help us know whether *kanyakla* actually helps to improve HIV care.

When forming the *kanyakla* group, either now or in one year, each participant will be asked to recruit close family, friends or other people in their lives to form a *kanyakla* group. These individuals should be trusted people that you rely on for support and who get along well with one another. The program will begin with an entrance survey, and HIV testing and counseling for all group members. The 6-session program will be completed over 3-4 months. At the end of the program, follow-up surveys be given to aid in our understanding of the strengths and weaknesses of the program. Each *kanyakla* group will choose their own meeting time and place.

The *kanyakla* sessions will cover several areas related to health and well being for people living with HIV/AIDS, including HIV biology, the scope of HIV around Lake Victoria, HIV medications, herbal medications, group support, destigmatization and group confidentiality. It will include information about HIV including how it is spread, and the effects it has on the health of those in the community affected by the virus. The sessions will focus on reducing the stigma faced by those living with HIV/AIDS. After completion of all 6 sessions, your *kanyakla* will have the opportunity to participate in VCT and a group HIV status disclosure session, allowing group members to share their HIV status with one another. This group disclosure session is optional – you will not be pressured to participate if you do not feel comfortable and not participating will not affect your ability to continue participating in your *kanyakla* group.

Each *kanyakla* session will last about 2 hours, and the beginning and ending surveys will each take about 30 minutes. The surveys will cover information about stigma, HIV status disclosure and reasons for missed clinic appointments. You are free to leave blank any question you are not comfortable answering. Each participant will be assigned a random number so that no identifying information will be kept with the survey results. Only research staff will have access to this data. No individual identities will be used in any reports or publications that may result from this study.

Any additional information about the study that arises during the course of the research will be provided to you. Information about the final study results will be available through the Ekialo Kiona Center.

Risks or Discomforts

Your involvement in this study has few risks, if any, but since we will be discussing sensitive information we want you to be aware of how your information will be kept.

The main risk of participating in this study is a loss of confidentiality. Every effort will be made to maintain privacy of health information during this study. Only research staff will have access to your health information and no personal identifying information (such as your name, national ID number, phone number, or exact location of your residence) will be connected to the data we gather. Study staff will assign participants a number that is not connected to your personal information so that your privacy can be protected.

Benefits

Participants will benefit from education about living with HIV/AIDS.

The information we gather from this research project will help improve future programming on Mfangano, Remba and Ringiti islands by understanding what works well, and how to efficiently coordinate interventions on a bigger scale. This also help the Ekialo Kiona Center and its partners to train organizations in similar communities to help improve health education for other HIV-positive people and their social-support groups throughout the region, and the world.

Confidentiality

We will keep all the information shared with us confidential. Your records will be handled as confidentially as possible. Only research staff will have access to the information we use, and no identifying information will be kept with the data we gather. No individual identities will be used in any reports or publications that may result from this study.

Cost/Payment

The only cost to you for being in the study is your time. You will receive 100 shillings after completion of the baseline survey and again after completion of the end of study survey. This money is intended to reimburse you for your time spent answering survey questions as part of the study.

Right to Refuse or Withdraw

You are free to join this study or not. If you decide to join, you are also free to change your mind and refuse to be in the study at any time for any reason.

Choosing not to participate in this study will in no way affect your relationship with OHR or FACES or any other partner who is working with these organizations.

Persons to Contact

If at any time during the project you have questions about the study, think you have been harmed in this study, or have concerns about your rights as a subject in this study, you may contact the Secretary, KEMRI Ethics Review Committee, P. O. Box 54840-00200, Nairobi; Telephone numbers: 020-2722541, 0722205901, 0733400003; Email address: erc-secretariat@kemri.org or the *Committee on Human Research* at the University of California, San Francisco (000-1-415-476-1814). Or you may write to: Committee on Human Research, Box 0962, University of California, San Francisco (UCSF), San Francisco, CA 94143. These committees are concerned with protection of volunteers in research studies.

Consent signing

The consent form has been explained to me and I agree to take part in this program. I understand that I am free to choose not to take part in this study at any time and that saying “NO” will have no effect on the care I receive.

You will receive a copy of this consent form to take with you.

| **Participant** | **Name:**  **ID #_______________** | **Signature:**  **or Fingerprint_______** | **Date**_______________________ |
| --- | --- | --- | --- |
| **Witness** | **Name:** | **Signature:** | **Date**________________________ |
| **Person conducting consent discussion** | **Name:** | **Signature:** | **Date**________________________ |

**Kanyakla Study**

*Informed Consent for Microclinic group members*

Investigator and Institutional Affiliations:

| **Name** | **Organization** | **Role on Project** |
| --- | --- | --- |
| Elizabeth Bukusi, MB.ChB., M. Med, MPH, PhD, PGD (Research ethics) | Kenya Medical Research Institute (KEMRI) | KEMRI-Principal Investigator |
| Craig Cohen, MD, MPH | University of California, San Francisco (UCSF) | Co-Investigator |
| Matt Hickey, MD candidate | University of California, San Francisco (UCSF) | Co-Investigator |
| Charles Salmen, MD, MPhil (Oxon.) | The Organic Health Response (OHR) | Co-Investigator |
| Dan Omollo, BSc, MPH candidate | The Organic Health Response (OHR) | Co-Investigator |
| Katie Fiorella, MPH | The Organic Health Response (OHR) | Co-Investigator |
| Dr. Betty Njoroge | Kenya Medical Research Institute (KEMRI) | Co-Investigator |
| Lindah Otieno | Family AIDS Care and Education Services (FACES) | Co-Investigator |
| Richard Magerenge | The Organic Health Response (OHR) | Research Coordinator |
| Gor Benard Ouma | The Organic Health Response (OHR) | Research Coordinator |
| Catie Glatz | University of Minnesota (UMN) | Research Coordinator |
| Elvin Geng, MD MPH | University of California, San Francisco (UCSF) | Study Consultant |
| Dr. CT Muga | Kenya Medical Research Institute (KEMRI) | Study Consultant |
| Dr. Norton Sang | Kenya Medical Research Institute (KEMRI) | Study Consultant |
| Daniel Zoughbie, MSc (Oxon.), DPhil | Microclinic International (MCI) | Study Consultant |

Introduction and Purpose

Researchers from the Ekialo Kiona Center are conducting a study to understand the effect of the Kanyakla *kanyakla* program on HIV care on Mfangano, Remba, and Ringiti Islands.. Our research team also includes researchers from Organic Health Response, Kenya Medical Research Institute (KEMRI), the Global Micro-Clinic Project (GMCP), the University of California San Francisco (UCSF), and the University of Minnesota (UMN). You are being invited to participate in this study because you are participating in a *kanyakla* group.

This study is trying to understand whether the *kanyakla* program helps HIV patients to attend their clinic appointments regularly. We have already tested the *kanyakla* program on a small scale in Mfangano East, and are now bringing the program to more people throughout Mfangano, Remba and Ringiti islands. Through this study, we hope to learn more about the strengths and weaknesses of *kanyakla*, to improve the program for the future, and to learn about whether *kanyakla* could be expanded to include other locations. This research will benefit the people of Mfangano, Remba, and Ringiti islands by providing health education. The information gathered from this study will also help improve the program, enable future expansion projects, and guide development of similar programs to improve care for people who are directly or indirectly affected by HIV/AIDS throughout Kenya and in other parts of the world.

Procedures

The study participants will be recruited from among *kanyakla* participants.

Study participation will begin with an entrance survey, and HIV testing and counseling for all group members. The 6-session *kanyakla* program will be completed over 3-4 months. At the end of the program, follow-up surveys be given to aid in our understanding of the strengths and weaknesses of the program.

The *kanyakla* sessions will cover several areas related to health and well being for people living with HIV/AIDS, including HIV biology, the scope of HIV around Lake Victoria, HIV medications, herbal medications, group support, destigmatization and group confidentiality. It will include information about HIV including how it is spread, and the effects it has on the health of those in the community affected by the virus. The sessions will focus on reducing the stigma faced by those living with HIV/AIDS. After completion of all 6 sessions, your *kanyakla* will have the opportunity to participate in a group VCT session, allowing group members to share their HIV status with one another. This group VCT session is optional – you will not be pressured to participate if you do not feel comfortable.

Each session will last about 2 hours, and the beginning and ending surveys will each take about 30 minutes. The surveys will cover information about HIV-related stigma, prior HIV testing and current HIV status. You are free to leave blank any question you are not comfortable answering. Each participant will be assigned a random number so that no identifying information will be kept with the survey results. Only research staff will have access to this data. No individual identities will be used in any reports or publications that may result from this study.

Any additional information about the study that arises during the course of the research will be provided to you. Information about the final study results will be available through the Ekialo Kiona Center.

Risks or Discomforts

Your involvement in this study has few risks, if any, but since we will be discussing sensitive information we want you to be aware of how your information will be kept.

The main risk of participating in this study is a loss of confidentiality. Every effort will be made to maintain privacy of health information during this study. Only research staff will have access to your health information and no personal identifiers will be connected to the data we gather. Study staff will assign participants a number that is not connected to your personal information so that your privacy can be protected.

Benefits

Participants will benefit from education about health.

The information we gather from this research project will help improve future programming by understanding what works well, and how to efficiently coordinate interventions on a bigger scale. This can enable OHR to train organizations in similar communities to help improve health education for other HIV-positive people and their social-support groups throughout the region, and the world.

Confidentiality

We will keep all the information shared with us confidential. Your records will be handled as confidentially as possible. Only research staff will have access to the information we use, and no identifying information will be kept with the data we gather. No individual identities will be used in any reports or publications that may result from this study.

Cost/Payment

The only cost to you for being in the study is your time. There will be no direct payment given for your participation.

Right to Refuse or Withdraw

You are free to join this study or not. If you decide to join, you are also free to change your mind and refuse to be in the study at any time for any reason. If you do not want to join the study, it will not prevent you from participating in *kanyakla* group sessions.

Choosing not to participate in this study will in no way affect your relationship with OHR or FACES or any other partner who is working with these organizations.

Persons to Contact

If at any time during the project you have questions about the study, think you have been harmed in this study, or have concerns about your rights as a subject in this study, you may contact the Secretary, KEMRI Ethics Review Committee, P. O. Box 54840-00200, Nairobi; Telephone numbers: 020-2722541, 0722205901, 0733400003; Email address: erc-secretariat@kemri.org or the *Committee on Human Research* at the University of California, San Francisco (000-1-415-476-1814). Or you may write to: Committee on Human Research, Box 0962, University of California, San Francisco (UCSF), San Francisco, CA 94143. These committees are concerned with protection of volunteers in research studies.

Consent signing

The consent form has been explained to me and I agree to take part in this program. I understand that I am free to choose not to take part in this study at any time and that saying “NO” will have no effect on the care I receive.

You will receive a copy of this consent form to take with you.

| **Participant** | **Name:**  **ID #_______________** | **Signature:**  **or Fingerprint_______** | **Date**_______________________ |
| --- | --- | --- | --- |
| **Witness** | **Name:** | **Signature:** | **Date**________________________ |
| **Person conducting consent discussion** | **Name:** | **Signature:** | **Date**________________________ |

**Session**

**6**

**Session 1**

**Session**

**2**

**Session 4**

**HIV on Lake Victoria:**

**Biology and**

**Sociology**

**Combination Antiretroviral**

**Therapy (cART):**

**How and Why**

**ARV’s and You:**

**Nutrition and Herbal Medication**

**Chasing Stigma:**

**Group Support and**

**Communication**

**Engaging with Care:**

**Being an Active**

**Patient**

**Session**

**3**

**Session**

**5**

**Pulling the**

**Net Together:**

**Counseling and Confidentiality**

*

****Facilitated**

**Group VCT Session**

**(Optional)**

**Session 1: HIV on Lake Victoria**

| **Topic** | **Method** | **Min** | **Content Summary** |
| --- | --- | --- | --- |
| Prayer and Introductions | class | 5 | - Have opening prayer. - Introduce members/facilitators |
| The Kanyakla | Class | 15 | - Discuss the MHN Kanyakla Program Goals and Overview |
| Daily Meditations | class | 15 | - Refer to daily meditation sheet. |
| Knowns and Unknowns | teaching game | 15 | - Listing what the group “knows” about HIV - List group “unknowns” |
| HIV on Lake Victoria | discussion | 10 | - Discuss local HIV stats and facts using KAIS 2013 |
| HIV games | teaching games | 35 | 1. HIV body game 2. HIV hidden ball game 3. HIV Fluids game |
| HIV biology | discussion | 30 | - Overview of HIV/AIDS |
| Wisdom Circle | class | 10 | - Review Handout |
|  | Total time | 135 Minutes | |

**Session 2: Combination Antiretroviral Therapy**

| **Topic** | **Method** | **Min** | **Content Summary** |
| --- | --- | --- | --- |
| Prayer and introductions | class | 5 | - Have opening prayer. |
| Mindfulness and meditations | class | 10 | - Refer to meditation sheet. |
| Wheel of Hope | teaching game | 10 | - Review questions from Session 2. |
| Two HIV Games | teaching game | 35 | #1 Teaching Game: HIV takes over a CD4 cell  #2 Teaching Game: How ARVs block HIV |
| Viral resistance Role Play | Role Play | 20 | - Role play of how HIV gains Resistance to ARVs |
| Wisdom Circle | class | 15 | - Review Handouts |
| Total time | | 120 minutes | |

Session 3: Engaging with Care:

| **Topic** | **Method** | **Minutes** | **Content Summary** |
| --- | --- | --- | --- |
| Prayer and introductions | class | 5 | - Have opening prayer. |
| Mindfulness and meditations | class | 10 | - Refer to meditation sheet. |
| Wheel of Hope | teaching game | 15 | - Review questions from previous sessions. |
| Role Plays | groups | 30 | - The Disorganized Patient - The Organized Patient - The Angry Nurse at the Clinic |
| Getting Organized | class | 20 | - Organizing ARV’s for life |
| The 5 Questions | class | 30 | - Discuss the 5 Questions an Active Patient needs to ask at Every Visit |
| Lean on Me | class | 15 | - Use Whiteboard to discuss helpful and unhelpful roles of Supporters |
| Wisdom Circle | class | 15 | - Review Handout |
| Total Time | | 125 minutes | |

**Session 4: ARV’s and You:**

| **Topic** | **Method** | **Minutes** | **Content Summary** |
| --- | --- | --- | --- |
| Opening prayer and mindfulness | class | 15 | - Opening prayer - Discuss daily meditations |
| Wheel of Hope and Agenda | teaching game | 15 | - Play Wheel of Hope game. - Review session agenda. |
| Role plays | class | 30 | 1. HIV clinic visit 2. Visit to the herbalist 3. Two people living with HIV/AIDS |
| Local beliefs about ARVs and Herbs | class | 30 | - Discuss local beliefs on HIV including Chira - Discuss appropriate use of herbs and ARVs |
| Nutrition on ART | class | 30 | - Discuss balanced diet for people on Antiretroviral therapy |
| Wisdom Circle | class | 10 | - Review session Handout |
| Total Time | | 130 minutes | |

**Session 5: Chasing Stigma:**

| **Topic** | **Method** | **Minutes** | **Content Summary** |
| --- | --- | --- | --- |
| Prayer and introductions | class | 5 | - Have opening prayer. |
| Daily Meditations | class | 10 | - Refer to daily meditation sheet. |
| Wheel of Hope | teaching game | 15 | - Review questions from previous sessions. |
| Stigma Role Plays | groups | 15 | 1. Stigma at the Market 2. Stigma at a Funeral 3. Chasing Stigma |
| Kanyakla Support | class | 35 | - Discuss the 5 Pillars of Kanyakla Support (Emotional, Material, Confidential, Organizational, and Spiritual) |
| Communication | class | 20 | - Discuss Basic Principles of Non-Violent Communication |
| Wisdom Circle | class | 10 | - Review Session Hand out |
| Total Time | | 125 minutes | |

**Session 6: Pulling the Net Together**

| **Topic** | **Method** | **Minutes** | **Content Summary** |
| --- | --- | --- | --- |
| Opening prayer and mindfulness | class | 10 | - Opening prayer and mindfulness training - Daily Meditations |
| Wheel of Hope and agenda | teaching game | 15 | - Play Wheel of Hope game. - Review session agenda. |
| Trust Games | games | 25 | 1. Play Blanket Game 2. Play Human Knot Game 3. Trust Fall Exercise |
| Role Plays | class | 20 | 1. The Unintended Disclosure 2. The Voluntary Disclosure 3. The Good Confidant |
| Confidentiality Discussion | class | 20 | - What is Confidentiality? - How is do we practice and enforce confidentiality? - How to be a good confidant? |
| Wisdom Circle | class | 10 | - Review Handout and Scheduling for Group VCT |
| Total Time | | 120 minutes | |

***Voluntary Group Counseling and Testing Preparation (Optional Sessi**on):

| **Topic** | **Method** | **Minutes** | **Content Summary** |
| --- | --- | --- | --- |
| Opening prayer and mindfulness | class | 10 | - Opening prayer - Daily Meditations |
| Role Plays | class | 20 | 1. The Husband and Wife Conversation 2. The Sister to Sister Conversation |
| Confidentiality Review | class | 10 | - What is Confidentiality? - How is do we practice and enforce confidentiality? - How to be a good confidant? |
| Guest Speaker | class | 20 | - Status Disclosure Testimonials |
| Overview of Group VCT | teaching | 15 | - Review of Ministry of Health Guidelines for Group VCT - Review “Voluntary” nature of HIV testing |
| Schedule Group VCT | Scheduling | 5 | - Determine best time for members who want to complete Group VCT together |
| ***Following Group VCT Session” | | | |
| Review of Group VCT Experience | Class | 20 | - Discuss how group members felt before, during and after group VCT |
| Facilitated Disclosure | Group | 30 | - Space for members to share status with kanyakla members |
| Review of Group Support | Class | 10 | - Review Group Support Principles |
| Kanyakla Contract | Group | 30 | - Signing of Kanyakla Contracts |
| Total Time | | 120 minutes | |

**Informed Consent for Qualitative Interviews/Discussion Groups**

**Kanyakla Study**

Investigator and Institutional Affiliations:

| **Name** | **Organization** | **Role on Project** |
| --- | --- | --- |
| Elizabeth Bukusi, MB.ChB., M. Med, MPH, PhD, PGD (Research ethics) | Kenya Medical Research Institute (KEMRI) | KEMRI-Principal Investigator |
| Craig Cohen, MD, MPH | University of California, San Francisco (UCSF) | Co-Investigator |
| Matt Hickey, MD candidate | University of California, San Francisco (UCSF) | Co-Investigator |
| Charles Salmen, MD, MPhil (Oxon.) | The Organic Health Response (OHR) | Co-Investigator |
| Dan Omollo, BSc, MPH candidate | The Organic Health Response (OHR) | Co-Investigator |
| Katie Fiorella, MPH | The Organic Health Response (OHR) | Co-Investigator |
| Dr. Betty Njoroge | Kenya Medical Research Institute (KEMRI) | Co-Investigator |
| Lindah Otieno | Family AIDS Care and Education Services (FACES) | Co-Investigator |
| Richard Magerenge | The Organic Health Response (OHR) | Research Coordinator |
| Gor Benard Ouma | The Organic Health Response (OHR) | Research Coordinator |
| Catie Glatz | University of Minnesota (UMN) | Research Coordinator |
| Elvin Geng, MD MPH | University of California, San Francisco (UCSF) | Study Consultant |
| Dr. CT Muga | Kenya Medical Research Institute (KEMRI) | Study Consultant |
| Dr. Norton Sang | Kenya Medical Research Institute (KEMRI) | Study Consultant |
| Daniel Zoughbie, MSc (Oxon.), DPhil | Microclinic International (MCI) | Study Consultant |

Introduction and Purpose

In order to better understand health challenges facing this community, researchers from the Organic Health Response, the Kenya Medical Research Institute (KEMRI) and the University of California, San Francisco (UCSF) would like to discuss health issues that you face and your ideas for solutions. You are being contacted because of your participation in the Kanyakla Study currently taking place on Mfangano, Remba and Ringiti islands.

Procedures:

We would like to have the opportunity to talk directly to Kanyakla study participants about their experiences with HIV care. We may ask you to participate in a group discussion with your entire Kanyakla, or we may ask you questions individually. Questions will relate to your health, the health of fellow kanyakla members, and experiences with the health centers. We will also discuss specifically issues related to HIV care that you or members of your kanyakla face. This interview or group discussion will last no more than 2 hours.

Risks or Discomforts

Your involvement in these focus groups has few risks for you. You may experience loss of confidentiality while participating in this study, although we will minimize this risk through the procedures described under “confidentiality” below. During these group discussions and activities, other participants will hear what you have to say. At no time during the group discussion will your name be written down in connection with the information you have provided. We will also ask you and the other participants not to tell anyone outside of the group what any person said during the discussion. However, we cannot guarantee that everyone will keep the discussions private. Individual intervews will be kept private between you and the interviewer and your name will not be recorded in connection with your responses.

Within the focus groups we will be asking you questions about your experiences with the Kanyakla program. If any question makes you uncomfortable at any time, you can feel free not to participate or answer.

Benefits:

The results of the evaluation will help us make the Kanyakla program more successful and more sensitive to the needs of the community. It may also help to include the quality of services available at the health center where you access care.

Confidentiality:

We will keep all the information shared with us confidential. Your records will be handled as confidentially as possible. All tapes/transcripts of the discussions and consent and data forms will be kept in locked files, and only study staff will have access to them. No individual identities will be used in any reports or publications that may result from this study.

Cost/Payment:

The only cost to you for being in the study is your time. Refreshments such as tea and mandazi will be served during group discussions.

Right to Refuse or Withdraw:

You are free to join the interview and/or focus group discussions or not. If you decide to join, you are also free to change your mind and refuse to be in the study at any time for any reason. Not participating will in no way affect your relationship with OHR or FACES.

Persons to Contact:

If at any time during the project you have questions about the study, think you have been harmed in this study, or have concerns about your rights as a subject in this study, you may contact The Secretary of the Ethical Research Committee, Kenya Medical Research Institute at Tel. 020-2722501 or the *Committee on Human Research* at the University of California, San Francisco (+1-415-476-1814). Or you may write to: Committee on Human Research, Box 0962, University of California, San Francisco (UCSF), San Francisco, CA 94143. These committees are concerned with the protection of volunteers in research projects.

Consent signing:

| The consent form has been explained to me and I agree to take part in focus group discussions. I understand that I am free to choose not to take part in this study at any time and that saying “NO” will have no effect on me. |  |
| --- | --- |
|  |

| **Participant** | **Name:**  **ID #_______________** | **Signature:**  **or Fingerprint_______** | **Date**_______________________ |
| --- | --- | --- | --- |
| **Witness** | **Name:** | **Signature:** | **Date**________________________ |
| **Person conducting consent discussion** | **Name:** | **Signature:** | **Date**________________________ |

**FOCUS GROUP DISCUSSION GUIDE**

**Kanyakla Study**

Opening Statement

Good morning. We are so glad to have your participation in this focus group. We would like to use this two hour time period to learn from you as community members about (insert topic). I would like to emphasize that we are NOT here as teachers and in fact, we are here to learn from you. We are here only as facilitators to help guide the flow of this group discussion. The purpose of this group is to provide a safe space for you to share your experiences, opinions, concerns and ideas as community members of Mfangano on the issue of (insert topic). We are here to pose questions about (insert topic) and guide group discussion when necessary. This is a discussion, not a traditional interview. There is no right or wrong answer you can give and everyone has an equal voice. You are welcome to respond directly to anything that someone else in the group says. We are here to hear your perspective and what you know from being a member of this community so that we can better understand (insert topic) on Mfangano. We ensure you that all of your information will be kept confidential and as a group we will make a covenant to not talk about what others have shared. We will use the confidential information you provide to help create better trainings and better programs for the community to address any concerns that arise through this discussion. We also cannot promise that everyone in this room will directly benefit from the information obtained in this discussion, but it is our sincerest intention to use the information you provide us to benefit the community. That is our primary purpose for holding this discussion. The process of this Focus Group is that we will raise a question and anyone who would like to respond will have a chance to respond. You can comment on others responses and ideas as is the nature of any discussion. Once everyone feels the question has been answered we will continue raising questions as needed and other questions from your comments may arise as well. We are only here to keep the conversation moving until our time is up or everyone feels they have had a chance to say what they would like to say. If at any time you have questions or need clarification about the questions we are asking, please ask them. We will also remain after the formal discussion has ended if you have any other questions or things you wish to share. Are there any questions about the nature of this discussion before we start?

Guide for Discussion

Topic: Topics will be drawn from the list provided in the protocol

Opening: Facilitator will begin with the opening statement above to greet participants and explain the process of the group. The co-facilitator will take notes and record the discussion.

Leading Question: “I would like to hear from you all what you and/or your community thinks about (insert topic).”

Probing Questions: Discussion will ensue based on participant responses and the facilitator will direct or redirect as necessary.

Ending: Facilitator will thank the group for their participation and explain confidentiality and how the information will be used one last time.

**Semi-Structured Interview Guide**

**Kanyakla Study**

Opening Statement

Good morning. We are so glad to have your participation in this interview. We would like to use this period to learn from you as a community member about (insert topic). The purpose of this interview is to provide a safe space for you to share your experiences, opinions, concerns and ideas as a community member of Mfangano on the issue of (insert topic). We are here to pose questions about (insert topic) and hear your thoughts and opinions. There is no right or wrong answer you can give. You are welcome to respond directly or refuse to answer any questions. You are also welcome to share ideas that we may not have asked about. We are here to hear your perspective and what you know from being a member of this community so that we can better understand (insert topic) on Mfangano. We ensure you that all of your information will be kept confidential and we will make a covenant to not talk about what others have shared. We will use the confidential information you provide to help create better trainings and better programs for the community to address any concerns that arise through this discussion. It is our sincerest intention to use the information you provide us to benefit the community. That is our primary purpose for holding this discussion. If at any time you have questions or need clarification about the questions we are asking, please ask them.

Guide for Informal Interview

Topic: Topics will be drawn from the list provided in the protocol

Opening: Facilitator will begin with the opening statement above to greet participants and explain the process of the group.

Topic 1: Experiences in HIV Care and Treatment: “I would like to hear from you about your experiences with HIV care and treatment on Mfangano. Is there anything in particular that you would like to share on this topic?

Probing Questions: Discussion will ensue based on participant responses and the facilitator will direct or redirect as necessary.

Topic 2: Challenges Faced: “I would like to hear from you about any challenges that you have faced while enrolled in HIV care and treatment on Mfangano. Is there anything in particular that you would like to share on this topic?

Probing Questions: Discussion will ensue based on participant responses and the facilitator will direct or redirect as necessary.

Topic 3: Opportunities for Improvement “I would like to hear from you about any opportunities that you have identified where HIV care and treatment on Mfangano could be improved. Is there anything in particular that you would like to share on this topic?”

Probing Questions: Discussion will ensue based on participant responses and the facilitator will direct or redirect as necessary.

Ending: Facilitator will thank participant for their participation and explain confidentiality and how the information will be used one last time.

**Severe Adverse Event Reporting**

Severe adverse events, including death from any cause and hospitalization not related to trauma or pregnancy, occuring among study participants during study enrollment will be reported to the KEMRI Ethical Review Committee within stated KEMRI reporting guidelines.
